# Supplementary material for: Genomic study of the Ket: a Paleo-Eskimo-related ethnic group with significant ancient North Eurasian ancestry
Source: Sci Rep. 2016 Feb 11;6:20768. doi: 10.1038/srep20768 (PMC4750364; doi:10.1038/srep20768)
Supplement: Supplementary Information [file srep20768-s1.pdf]

Genomic study of the Ket: a Paleo-Eskimo-related ethnic group with significant ancient North Eurasian ancestry

Pavel Flegontov, Piya Changmai, Anastassiya Zidkova, Maria D. Logacheva, N. Ezgi Altınışık, Olga Flegontova, Mikhail S. Gelfand, Evgeny S. Gerasimov, Ekaterina E. Khrameeva, Olga P. Konovalova, Tatiana Neretina, Yuri V. Nikolsky, George Starostin, Vita V. Stepanova, Igor V. Travinsky, Martin Tříska, Petr Tříska, Tatiana V. Tatarinova

CONTENTS

1. Ket settlements along the Yenisei River 2
2. Linguistic affinities of Ket language 3
3. Summary of datasets and analyses performed 7
4. Identification of a non-admixed Ket genotype 13
5. ADMIXTURE analysis 19
6. Principal component analysis 29
7. f_3 statistic 39
8. f_4 statistic 73
9. TreeMix analysis 102
10. Mitochondrial and Y-chromosomal haplogroups 111
11. Neanderthal contribution to the Ket genomes 116

1. Ket settlements along the Yenisei River

There are currently six compact areas in Krasnoyarsk Krai where Ket settlements are located (Suppl. Fig. 1.1): four areas in the Turukhansk administrative district, one in the Yeniseisk district, and another one in Evenkia. Settlement areas in the Turukhansk district are the following: Yelogui (the Kellog village on the Yelogui River), Surgutikha (the Surgutikha village on the Surgutikha River), Pakulikha (the Baklanikha village on the Yenisei River), Kureika (the Serkovo village on the Kureika River and the Maduika village on Maduiskoe Lake). Ket people of the Sym group (approximately 40 Ket individuals in the Sym village located on the Sym River) live in the Yeniseisk district. Kets of the Podkamennaya Tunguska group (the Sulomai village on the Podkamennaya Tunguska River) live in Evenkia, now a district of the Krasnoyarsk region. All these villages mentioned above were established in the early Soviet period, with the exception of Baklanikha, that was established in 1810 by Russian settlers. There are documentary evidences about marriages between Kets and Selkups; the highest probability of such marriages is observed for Kets in the Kellog and Baikha villages, while non-admixed Kets are expected in Bakhta, Komsa, Verkhneimbatsk, Alinskoe, Maduika, and Sulomai (Krivonogov 1998, 2003). The northern areas of the Yenisei basin, such as Igarka, were settled in 19-20th centuries by Nenets, Evenks, Selkups and even Yakuts. Therefore, Kets living along the Northern Yenisei are more likely to be of mixed origin.

References

Krivonogov, V. P. *Kety na poroge III tysyacheletiya [The Ket on the threshold of the 3rd millennium]*. Krasnoyarsk: RIO KGPU (1998).

Krivonogov, V. P. *Kety: Desyat' Let Spustya [The Ket: Ten Years Later]*. Krasnoyarsk: RIO KGPU (2003).

1.1. Map of sampling locations was plotted using QGIS v.2.8.

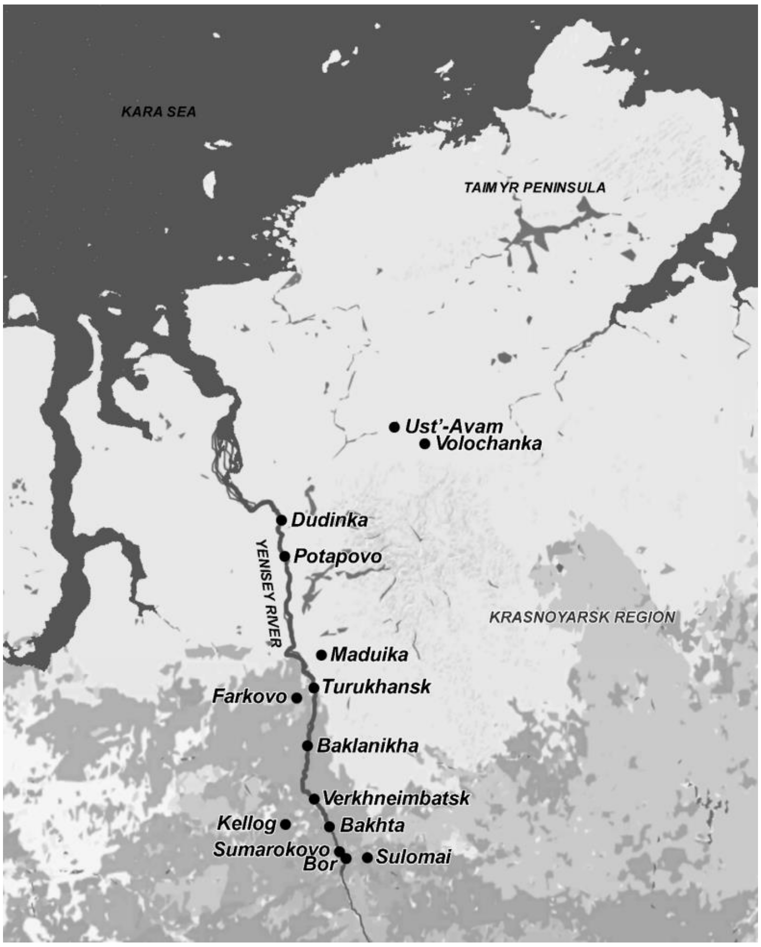

2. Linguistic affinities of Ket language

Linguistically, modern Ket language is the only remaining member of a formerly much larger linguistic family which, in addition to Ket, also contained the closely related Yugh (Sym) language that got extinct recently, as well as the more distantly related Kott, Arin, Assan, and Pumpokol languages. The latter four languages became extinct by the late 19th century and are only known today from sources recorded in the 18th-19th centuries (Werner 2005). Available data on these languages was, however, sufficient to allow the phonological and lexical reconstruction of their common ancestor, Proto-Yeniseian language (Starostin S. 1982, 1995), whose disintegration, according to glottochronological calculations, may have taken place approximately 2,500 years ago (Starostin G. 2013).

Deeper genetic-linguistic connections of Ket (and Yeniseian languages in general) remain controversial and far more problematic than their areal connections, i.e. elements of linguistic convergence acquired through various linguistic contacts with their geographic neighbors. Today, the main source of borrowings into Ket is, predictably, Russian. Several linguistic studies documented contacts with Siberian Turkic languages (this concerns especially those languages that were spoken south of the Ket areal, i.e. Kott and Arin), and with Uralic languages, particularly the Samoyed branch, and most especially the Selkup language, with which Ket, according to some descriptions, has had a "symbiotic" relationship (Khelimskiy 1982). These linguistic ties are of significant importance for ethno-cultural studies on the Ket people, but they shed no light on the actual origins of Proto-Yeniseian language, other than suggesting its formerly wide expansion over Central Siberia, a fact that is also indirectly confirmed by a large number of Siberian hydronyms that stretch as far south as Khakassia and northern Mongolia (Dul'zon 1959, 1962; reviewed in Vajda 2001).

Some authors suggested a possible connection between Proto-Yeniseians (or some early branch of Yeniseians) and certain nomadic tribes of Central Asia that appear in historical records, most notably the Xiongnu (Huns). Xiongnu language presumably contains certain elements that may be identified as Yeniseian in origin (Pulleyblank 1962; Vovin 2000, 2002). There is also a possible link to the Dinglings, a separate people known from Han-era Chinese chronicles, who are assumed to have migrated south from an area west of Lake Baikal (Werner 2004). Although some of the linguistic evidence in favor of such a link looks intriguing, very little is reliably known of the languages of these early tribes to judge these hypotheses as conclusive. However, these suggestions agree with a prominent presence of the Yeniseian ethnic component in East Asia, suggested by the hydronymic evidence and areal ties of known Yeniseian languages.

Soviet linguists included Yeniseian languages into a vague areal linguistic conglomeration called 'Paleo-Asiatic' or 'Paleo-Siberian'. This group included a large number of linguistic isolates or small language groups scattered across vast areas of Siberia and the Far East and showing no obvious historical ties with each other. In addition to Yeniseian, this amorphous grouping also included Chukchi-Kamchatkan, Yukaghir, Nivkh, sometimes also Eskimo-Aleut and/or Ainu. At the same time, numerous typological similarities, as well as some rather unsystematic phonetic resemblances between certain words, were identified between Yeniseian languages and certain other 'relict' linguistic units of the Old World outside Siberia (an overview of the main literature may be found in Werner 2004).

In the early 1980s, these hypotheses were generalized by Sergei Starostin (Starostin 1984). On the basis of comparison of the reconstructed phonology and lexicon of Proto-Yeniseian with the respective reconstructions for Proto-North Caucasian and Proto-Sino-Tibetan, Starostin claimed that the three protolanguages are, in their turn, linked together by a system of regular phonetic correspondences and should therefore be regarded as descendants of an even more remote protolanguage, which he called 'Sino-Caucasian'. A similar hypothesis was around the same time put forward by a number of American linguists, including Joseph Greenberg and John Bengtson, who proposed an even larger grouping which also included such Eurasian language isolates as Basque and Burushaski, as well as the Na-Dene (Tlingit-Eyak-Athabaskan) language family in North America. Additionally, Merritt Ruhlen (1994, 1998) has asserted, based on a number of lexical comparisons, that within this large 'macrofamily' Yeniseian is particularly closely affiliated with Na-Dene. The 'Sino-Caucasian' or 'Dene-Caucasian' hypothesis did not find widespread acceptance among linguists, as many have expressed dissatisfaction with the methodology of both its American proponents (commonly known as 'mass' or 'multilateral comparison') and the Russian scholars, criticizing Starostin's comparisons for their extreme complexity which has been described as typologically incredible (e.g. van Driem 2005).

In a separate line of research, a possible link between Yeniseian and Na-Dene languages has been thoroughly investigated by Edward Vajda (2010a). In his most recent works, Vajda does not negate the possibility of 'Dene-Yeniseian' as part of a larger 'Dene-Caucasian' entity, but prefers to remain agnostic on the issue (Vajda 2010b). Vajda offered his own model of regular phonetic correspondences between Proto-Yeniseian and Proto-Na-Dene, as well as complex morphological evidence that was interpreted as reflexion of a common system of verbal conjugation in their common linguistic ancestor. Unlike earlier proposals, Vajda's 'Dene-Yeniseian' hypothesis has been positively evaluated by several linguists (e.g. Comrie 2010; Hamp 2010; Nichols 2010), and has at the same time stirred up some interdisciplinary interest, leading to an increase in publications on possible historical, ethnographical, and genetic connections between Yeniseians (and the Siberian peoples in general) and native Americans of Na-Dene linguistic affiliation and beyond (e.g. Sicoli and Holton 2014).

On the other hand, Vajda's 'Dene-Yeniseian' has also been criticized both by those linguists who are skeptical of 'Sino-/Dene-/Caucasian' as a whole (Campbell 2011) and those who share a positive attitude towards the larger hypothesis, but do not agree with an especially tight link between Yeniseian and Na-Dene. Thus, Starostin (2010, 2012) critically analyzes and rejects a large part of Vajda's morphological and lexical argumentation, concluding that what they call strong evidence is most likely insufficient to support a 'Dene-Yeniseian' outside of a 'Dene-Caucasian'. He also argues that there is much stronger evidence for a special binary relation between Yeniseian and the isolated Burushaski language in the Pamir mountains – a point of view earlier defended by George van Driem (2001), which would also seem more rational from a purely geographical point of view.

Notwithstanding the controversy that continues to surround all of the listed hypotheses, it may be predicted that most of the research on the linguistic prehistory of Yeniseians in the near future will continue to be conducted from a 'Dene-Caucasian' perspective, either global or partial. Although a few scattered proposals have been made now and then about potential ties between Yeniseian and other

language families (e.g. Indo-European), they have not received any serious attention from the linguistic community for obvious paucity of evidence.

References

Campbell, L. Review of 'The Dene-Yeniseian Connection', ed. by James Kari and Ben A. Potter. *Int. J. Am. Linguistics* **77,** 445–451 (2011).

Comrie, B. The Dene-Yeniseian hypothesis: an introduction. *The Dene-Yeniseian Connection*, ed. Kari, J., Potter, B. A. *Anthropological Papers of the University of Alaska: New Series* **5,** 25–32 (2010).

van Driem, G. *Languages of the Himalayas: An Ethnolinguistic Handbook of the Greater Himalayan Region, Containing an Introduction to the Symbiotic Theory of Language (2 vols.)*. Leiden: Brill (2001).

van Driem, G. Sino-Austronesian vs. Sino-Caucasian, Sino-Bodic vs. Sino-Tibetan, and Tibeto-Burman as default theory. *Contemporary Issues in Nepalese Linguistics*, Yadava, Y. P., Bhattarai, G., Lohani, R. R., Prasain, B., Parajuli, K., eds. Kathmandu: Linguistic Society of Nepal, 285–338 (2005).

Dul'zon, A. P. Ketskie toponimy Zapadnoy Sibiri [Ket toponyms of Western Siberia]. *Uchenye Zapisky Tomskogo Gosudarstvennogo Pedagogicheskogo Instituta [Scholarly Proceedings of Tomsk State Pedagogical Institute]* **18,** 91–111 (1959).

Dul'zon, A. P. Byloe rasselenie Ketov po dannym toponimiki [The former settlement of the Kets according to the facts of toponymy]. *Voprosy Geografii* **68,** 50–84 (1962).

Hamp, E. P. On the first substantial trans-Bering language comparison. *The Dene-Yeniseian Connection*, ed. Kari, J., Potter, B. A. *Anthropological Papers of the University of Alaska: New Series* **5,** 285–298 (2010).

Khelimskiy, E. A. Keto-Uralica. *Ketskij sbornik. Antropologija, etnografija, mifologija, lingvistika*. Leningrad: Nauka, 238–250 (1982).

Nichols, J. Proving Dene-Yeniseian genealogical relatedness. *The Dene-Yeniseian Connection*, ed. Kari, J., Potter, B. A. *Anthropological Papers of the University of Alaska: New Series* **5,** 299–309 (2010).

Pulleyblank, E.G. The Consonantal System of Old Chinese. *Asia Major* **9,** 58–144, 206–265 (1962).

Ruhlen, M. Na-Dene etymologies. In: Ruhlen, M. *On the Origin of Languages*. Stanford University Press, 93–110 (1994).

Ruhlen M. The origin of the Na-Dene. *Proc. Natl. Acad. Sci. USA.* **95,** 13994–13996 (1998).

Sicoli, M. A., Holton, G. Linguistic phylogenies support back-migration from Beringia to Asia. *PLoS One* 9, e91722 (2014).

Starostin, G. Dene-Yeniseian and Dene-Caucasian: pronouns and other thoughts. *Working Papers in Athabaskan Languages 2009. Berkeley, California, July 10-12, 2009*. Alaska Native Language Center: Working Papers № 8, 107–117 (2010).

Starostin, G. Dene-Yeniseian: a critical assessment. *J. Language Relationship* **8,** 117–138 (2012).

Starostin, G. Annotated Swadesh wordlists for the Yeniseian group (Yeniseian family). On-line at: http://starling.rinet.ru/new100/yen.pdf (2013).

Starostin, S. A. Praenisejskaja rekonstruktsija i vneshnie sv'azi enisejskikh jazykov [Proto-Yeniseic reconstruction and the external relations of the Yeniseic languages]. *Ketskij sbornik. Antropologija, etnografija, mifologija, lingvistika*. Leningrad: Nauka, 144–237 (1982).

Starostin, S. A. Gipoteza o geneticheskikh sv'az'akh sinotibetskikh jazykov s enisejskimi i severnokavkazskimi jazykami [A hypothesis about the genetic connections of Sino-Tibetan, Yeniseic, and North Caucasian languages]. *Lingvisticheskaja rekonstruktsija i drevnejshaja istorija Vostoka, 4*. Moscow: Nauka, 19–38 (1984).

Starostin, S. A. Sravnitel'nyj slovar' enisejskikh jazykov [Comparative dictionary of the Yeniseic languages]. *Ketskij sbornik. Lingvistika*. Moscow: Jazyki russkoj kul'tury, 176–315 (1995).

Starostin, S. A. *Sino-Caucasian (phonology, glossary)*. Ms., online at: http://starling.rinet.ru/Texts/scc.pdf (2005).

Vajda, E. J. *Yeniseian Peoples and Languages: a History of Their Study with an Annotated Bibliography and a Source Guide.* Surrey, England: Curzon Press, 389 p. (2001).

Vajda E. J. A Siberian link with Na-Dene languages. *The Dene-Yeniseian Connection*, ed. Kari, J., Potter, B. A. *Anthropological Papers of the University of Alaska: New Series* **5,** 33–99 (2010a).

Vajda E. J. Yeniseian, Na-Dene, and Historical Linguistics. *The Dene-Yeniseian Connection*, ed. Kari, J., Potter, B. A. *Anthropological Papers of the University of Alaska: New Series* **5,** 100–118 (2010b).

Vovin, A. Did the Xiong-nu speak a Yeniseian language? *Central Asiatic J.* **44,** 87–104 (2000).

Vovin, A. Did the Xiongnu Speak a Yeniseian Language? Part 2: Vocabulary. *Central Asiatic J.* **46**, 389–394 (2002).

Werner, H. *Zur jenissejisch-indianischen Urverwandtschaft*. Wiesbaden: Harrassowitz (2004).

Werner, H. *Die Jenissej-Sprachen des 18. Jahrhunderts [Yeniseian Languages of the 18th Century].* Wiesbaden: Harrassowitz (2005).

3. Summary of datasets and analyses performed

Suppl. Table 1. Datasets and analyses performed. Analyses shown in the main text are highlighted in violet.

dataset name	**GenoChip + Illumina arrays**	**Ket genomes + Illumina arrays**	**Ket genomes + HumanOrigins array**	**Ket genomes + HumanOrigins array**	**Ket genomes + HumanOrigins array + Verdu et al. 2014**	**Ket genomes + reference genomes**	**Ket genomes + reference genomes / transversions**	**Ket genomes + Raghavan et al. 2015**	**Ket genomes + Raghavan et al. 2015 / transversions**
dataset basis	GenoChip	2 Ket genomes	2 Ket genomes	2 Ket genomes	2 Ket genomes	2 Ket genomes	2 Ket genomes	4 Ket genomes	4 Ket genomes
reference data	Illumina arrays	Illumina arrays	Lazaridis et al. 2014	Lazaridis et al. 2014	Lazaridis et al. 2014, Verdu et al. 2014	reference genomes	reference genomes	reference genomes	reference genomes
Ket884 removed	no	no	no	yes	no	no	no	no	no
transitions excluded	no	no	no	no	no	no	yes	no	yes
SNP count	32,189	103,495	195,918	195,918	68,625	398,163	189,964	225,010	104,727
populations*	90	105	139	139	145	36	36	43	43
individuals*	1624	2549	1786	1785	1868	64	64	79	79
r^2 LD threshold	0.4	0.5	0.5	0.5	0.5	0.5	0.5	0.5	0.5
minor allele frequency threshold	0.05	N/A	N/A	N/A	0.05	N/A	N/A	N/A	N/A
missing rate per SNP threshold	0.03	0.05	0.05	0.05	0.03	0.11	0.11	0.09	0.09
max. missing rate per individual	0.85	0.04	0.286	0.286	0.29	0.685	0.703	0.684	0.695
individual with the max. missing rate	Saqqaq		Mal'ta	Mal'ta	Mal'ta	Mari	Mari	Mari	Mari
average genotyping rate	0.99267	0.99906	0.99568	0.99568	0.99557	0.93649	0.93559	0.94093	0.94068
f_3 permutations, all vs. all	352,440	562,380	1,462,032	1,462,032	1,653,900	21,420	21,420	37,023	37,023
f_3 permutations, 1 pop. fixed	11,748	16,068	30,459	30,459	33,078	1,785	1,785	2,583	2,583
f_4 permutations, all vs. all	7,665,570	14,340,690	51,536,628	51,536,628	60,780,825	176,715	176,715	370,230	370,230
f_4 permutations, 1 pop. pair fixed	3,828	5,253	10,011	10,011	10,878	561	561	820	820
SNP window for f_3 and f_4 standard error computation	10	N/A	50	50	10	50	50	50	50
PCA, PC1 vs PC2									
PCA, PC3 vs PC4									
ADMIXTURE									

correlation of admixture components with mt/Y-chr haplogroups									
f_3 (Yoruba; Ket, X)									
f_3 (Yoruba; Selkup/Nganasan/Enets, X)									
f_3 (Yoruba; Na-Dene-speaking, X)	Athabaskans				Chipewyans, Tlingit	Athabaskans	Athabaskans		
f_3 (Yoruba; Haida, X)									
f_3 (Yoruba; Mal'ta, X)									
f_3 (Yoruba; Saqqaq, X)									
f_3 (Yoruba; Karasuk, X)									
admixture f_3 (Test; X, Y)									
all vs. all f_3(Yoruba; Test, X) correlation									
f_4 (X, Chimp; Mal'ta, Stuttgart)†						**	**		
f_4 (X, Chimp; Mal'ta, Loschbour)†						**	**		
f_4 (X, Chimp; Loschbour, Stuttgart)†						**	**		
f_4 (X, Papuan; Sardinian, Mal'ta)‡									
f_4 (Mal'ta, Yoruba; Y, X)									
f_4 (Saqqaq, Yoruba; Y, X)									
f_4 (Ket, Chimp; Haida, X)									
f_4 (Ket, Yoruba; Y, X)									
f_4 (Karasuk, Yoruba; Y, X)									
f_4 (Haida, Chimp; Ket, X)									
f_4 (Athabaskan, Yoruba; Y, X)									
f_4-ratios, Mal'ta ancestry in Kets									
f_4-ratios, Mal'ta ancestry in Native Americans									
f_4-ratios, Siberian ancestry in Saqqaq									
f_4-ratios, Saqqaq ancestry in Na-Dene			Chipewyans		Chipewyans		Athabaskans		
TreeMix									

* Chimp, Neanderthals and Denisovans not counted

** Yoruba used as an outgroup instead of Chimp

† the f_4 set-up follows Lazaridis et al. (2014)

‡ the f_4 set-up follows Seguin-Orlando et al. (2014)

Suppl. Table 2. Population composition of datasets.

Population	**GenoChip + Illumina arrays**	**Ket genomes + Illumina arrays**	**Ket genomes + HumanOrigins array**	**Ket genomes + HumanOrigins array + Verdu et al. 2014**	**Ket genomes + reference genomes**	**Ket genomes + Raghavan et al. 2015**
Abkhasian	24	23	9	9		
Adygei		17	17	17		
Afanasievo culture					1	1
Albanian			6	6		
Aleut		8	11	11	1	1
Algonquin			9	9		
Altaian	31	28	7	7		2
Andronovo culture					2	2
Armenian	35	35	10	10		
Athabaskan	21	21			2	2
Australian			3	3	3	3
Avar					1	1
Aymara	5	23	5	5		
Balkar	19	22	10	10		
Balochi		21	20	20		
Bantu Kenya		9				
Bantu South Africa		8				
Basque		24	29	29		
Belarusian	9	17	10	10		
Bengali			7	7		
Bergamo			12	12		
Biaka			20	20		
Bolivian			7	7		
Bougainville			10	10		
Brahui		23	21	21		
British						
Bulgarian	28	13	10	10		
Burusho		25	23	23		
Buryat	15	32				2
Cabecar	29	31	6	6		
Cambodian			8	8		
Chechen	24	20	9	9		
Chilote			4	4		
Chinese	12					
Chipewyan	3	15	30	30		
Chukchi	11	41	23	23		
Chuvash	17	19	10	10		
Clovis	1				1	1
Cree			13	13		
Croatian			10	10		
Cypriot			8	8		
Czech			10	10		
Dai			10	10	2	2
Danish	15					
Daur		9	9	9		
Dinka			7	7	2	2
Dolgan	4	8	3	3		
Druze			39	39		
East Greenland	4	7				

Egyptian	27	12	18	18		
Enets	3					
English			10	10		
Eskimo	16	16	22	22		2
Estonian	15	15	10	10		
Even	32	32	10	10		
Evenk	21	36				
Finnish	52		7	7		
French		28	25	25	2	2
French South			7	7		
Georgian	20	30	10	10		
German	15					
Greek	15	20	20	20		
Greenlander Inuit					2	2
Guarani			5	5		
Gujarati	12	82				
Hadza			22	22		
Haida				10		
Han		127	33	33	2	2
Han North			10	10		
Hazara		17	14	14		
Hezhen		8	8	8		
Huichol						1
Hungarian	20	20	20	20		
Iberian	12					
Icelandic			12	12		
Indian	62				1	1
Ingush	4					
Iranian	36	20	8	8		
Iron Age Altai					2	2
Iron Age Russia					1	1
Italian	15	23				
Itelmen			6	6		
Japanese	12	113	29	29		
Jordan		20				
Kabardin		3				
Kalash		23	18	18		
Kalmyk			10	10		
Kaqchikel			5	5		
Karasuk culture					6	6
Karitiana	13	13	12	12	3	3
Kazakh	18	18				
Kenyan	12					
Ket	2	4				2
Ket (this study)	46	2	2	2	2	2
Khakas	17	17				
Khanty		35				
Kharia			12	12		
Kinh			8	8	2	2
Korean			6	6		
Koryak	17	27	9	9		2
Kumyk	14	17	8	8		
Kurd		6				

Kusunda			10	10		
Kuwaiti	18					
Kyrgyz	19	21	9	9		
La Brana	1		1	1		
Late Dorset					1	1
Lebanese	29	8				
Lezgin	18	21	9	9		
Lithuanian	10	10	10	10		
Lodhi			13	13		
Loschbour			1	1	1	1
Luhya		73				
Madagascan	21					
Makrani		20	20	20		
Mal'ta			1	1	1	1
Mala			13	13		
Maltese			8	8		
Mandenka			17	17	2	2
Mansi			8	8		
Mari	15	15			1	1
Mayan		49	18	18	1	2
Mbuti			10	10	2	2
Melanesian		10				
Miao			10	10		
Mixe	17	17	10	10	1	1
Mixtec			10	10		
Mongolian	11		6	6		
Mordovian	15	15	10	10		
Motala12			1	1	1	1
Namibian	20					
Naukan	16	16				
Naxi			9	9		
Nenets		3				
Nganasan	22	22	11	11		
Nganasan (this study)	24					
Nisga'a				8		
Nivkh	3	3			2	2
Nogai	16	16	9	9		
North East Finn	41					
North Ossetian	15	18	10	10		
Norwegian			11	11		
Ojibwa			19	19		
Onge			11	11		
Orcadian			13	13		
Oroqen		9	9	9		
Palestinian		48	38	38		
Papuan	27	16	14	14	2	2
Pathan		22	19	19		
Piapoco			4	4		
Pima	29	33	14	14		
Punjabi			8	8		
Quechua	11	40	7	7		
Romanian	31	16				

Russian	18	50	22	22		
Saami WGA			1	1		
San					2	2
Saqqaq	1		1	1	1	1
Sardinian	15	28	27	27	2	2
Selkup	7	16	10	10		
Selkup (this study)	15					
She			10	10		
Shor	20	20				
Sicilian			11	11		
Sindhi		22	18	18		
South African	19					
Spanish		12	53	53		
Spanish North			5	5		
Splatsin				9		
Stswecem'c				13		
Stuttgart			1	1	1	1
Surui	24	24	8	8		
Swedish		18				
Tabasaran		3				
Tajik	28	15			1	1
Tajik Pamiri			8	8		
Tatar	15	20				
Teleut	10	10				
Tepehuano	22	25				
Thai			10	10		
Tiwari			15	15		
Tlingit				16		
Tsimshian				26		1
Tu		10	10	10		
Tubalar			22	22		
Tujia			10	10		
Tunisian	12		8	8		
Turkish		19	56	56		
Turkmen	15	15	7	7		
Tuscan		96	8	8		
Tuvinian		15	10	10		
Ukrainian	20	20	9	9		
Ulchi			25	25		
Uygur		10	10	10		
Uzbek	19	27	10	10		
Vanuatuan	10					
Vietnamese	29	17				
Vishwabrahmin			13	13		
West Greenland		8				
Xibo		9	7	7		
Yakut	17	51	20	20		2
Yi			10	10		
Yoruba	12	129	70	70	4	4
Yukaghir	11	13	19	19		
Zapotec	21	43	10	10		
**populations**	**90**	**105**	**139**	**145**	**36**	**43**
**individuals**	**1624**	**2549**	**1786**	**1868**	**64**	**79**

4. *Identification of a non-admixed Ket genotype*

Methods

Clustering

Within the Ket population, we have found a number of subpopulations using a combination of KMEANS clustering and Kullback-Leibler distance approach (Sahu and Cheng 2003). We used the KMEANS clustering routine in *R*. Let N be the number of individuals. We ran the KMEANS clustering for *k* ranging from the N to two, using the matrix of admixture proportions as input (the matrix was calculated with ADMIXTURE (Alexander et al. 2009) for the dataset GenoChip). At each iteration, we calculated the ratio of the sum of squares between groups and the total sum of squares. If this ratio was >0.9, then we accepted the *k*-component model. Since KMEANS clustering cannot be implemented for *k*=1, to decide between two clusters or a possible single cluster, we also calculated Kullback-Leibler distance (KLD) between the *k*=2 and *k*=1 models. If the KLD <0.1 and the ratio of the sum of squares between groups and the total sum of squares for two-component model was above 0.9, then the *k*=1 model was selected because, in such cases, there were no subgroups in the population.

GPS

An admixture-based Geographic Population Structure (GPS) method (Elhaik et al., 2014) was used for predicting the provenance of all genotyped individuals (including relatives). GPS finds a global position where the individuals with the genotype closest to the tested one live. GPS is not suitable to analyzed recently admixed individuals. GPS calculated the Euclidean distance between the sample's admixture proportions and the reference dataset. The matrix of admixture proportions was calculated with ADMIXTURE (Alexander et al. 2009) for dataset GenoChip. The shortest distance, representing the test sample's deviation from its nearest reference population, was subsequently converted into geographical distance using the linear relationship observed between genetic and geographic distances. The final position of the sample on the map was calculated by a linear combination of vectors, with the origin at the geographic center of the best matching population weighted by the distances to 10 nearest reference populations and further scaled to fit on a circle with a radius proportional to the geographical distance.

reAdmix

reAdmix (Kozlov et al. 2015) estimates individual mixture in terms of present-day populations and operates in unconditional and conditional modes. reAdmix models ancestry as a weighted sum of present-day populations (e.g. 50% British, 25% Russian, 25% Han Chinese) based on the individual's admixture components. In conditional mode, the user may specify one or more known ancestral populations, and in unconditional mode, no such information is provided. We used reAdmix for analysis of the Ket, Selkup, Nganasan, and Enets samples in unconditional mode, and the matrix of admixture proportions was calculated with ADMIXTURE (Alexander et al. 2009) for dataset GenoChip.

Results

For all 158 samples genotyped in this study we computed distance between individuals using the following formula: $D(A, B) = 1 - \frac{2 \times S(A,B)}{S(A,A)+S(B,B)}$, where $S(A, B)$is similarity between SNP profiles of individuals A and B, calculated as follows: $S(A, B) = \sum_{i=1}^{N} \quad s(a_i, b_i)$, where if both alleles are identical, $s(a_i, b_i) = 1$; if only one of the alleles matches, 0.5; and if none matches, 0. N is the total number of genotyped SNPs, equal to 150,541 for the full GenoChip array including X-, Y-chromosomal, and mitochondrial SNPs (Elhaik et al. 2013). The resulting distance matrix was used as an input for the hclust routine in R and displayed using package *ape* (Suppl. Fig. 4.1). Separation of the Ket population into five distinct clusters can be possibly explained by several factors: geography, family structure (see percentage of relatedness in Suppl. file S1), and admixture from other ethnic groups.

Then we applied GPS (Elhaik et al., 2014) and reAdmix (Kozlov et al. 2015) algorithms to infer provenance of the samples and confirm self-reported ethnic origin. For that purpose we compared the GenoChip SNP array data for the Ket, Selkup, Nganasan, and Enets populations (Suppl. file S1) to the worldwide collection of populations (Elhaik et al., 2014) based on 130K ancestry-informative markers (Elhaik et al. 2013). According to the GPS analysis, 46 of 57 (80%) self-reported Kets were identified as Kets, 9 (16%) as Selkups, one as a Khakas, and one as a Dolgan (Turkic speakers from the Taymyr Peninsula). In addition to the proposed population and geographic location, GPS also reports prediction uncertainty (the smallest distance to the nearest reference population) (Suppl. Fig. 4.2). The average prediction uncertainty was 2.5% for those Ket individuals identified as Kets; as Selkups, 4.4%; as Khakas, 5.6%; and 3.9% for the individual identified as a Dolgan. Prediction uncertainty over 4% indicates that the individual is of a mixed origin and the GPS algorithm is not applicable.

Using the reAdmix approach (in the unconditional mode) (Kozlov et al. 2015), we represented 57 Kets as weighted sums of modern reference populations (Suppl. Fig. 4.3, Suppl. Table 3). The median weight of the Ket ancestry in self-identified Kets was 94%; 39 (68%) of them had over 90% of the Ket ancestry (non-admixed Kets). Seven individuals with self-reported purely Ket origin appear to be closer to Selkups, with median 89% percent of Selkup ancestry. This closeness is not surprising, given the long shared history of Ket and Selkup people (Vajda 2004). Individuals with incorrect self-identification were randomly distributed across sixteen birthplaces along the Yenisei River (Suppl. Table 3). 86% of GPS predictions agree with the major ancestry prediction by reAdmix (Suppl. Table 3). The Pearson's correlation between percentage of major ancestry and GPS uncertainty is -0.42, meaning that the individuals predicted by reAdmix to be of non-admixed origin are likely to be predicted to be non-admixed by GPS as well. Hence, we identified a subset of non-admixed Kets among self-identified Ket individuals, and nominated individuals for whole-genome sequencing.

References

Alexander, D. H., Novembre, J., Lange, K. Fast model-based estimation of ancestry in unrelated individuals. *Genome Res.* **19,** 1655–1664 (2009).

Elhaik, E. *et al.* The GenoChip: a new tool for genetic anthropology. *Genome Biol. Evol.* **5,** 1021–1031 (2013).

Elhaik, E. *et al*. Geographic population structure analysis of worldwide human populations infers their biogeographical origins. *Nat. Commun.* **5,** 3513 (2014).

Kozlov, K. *et al*. Differential Evolution approach to detect recent admixture. *BMC Genomics* **16 Suppl 8,** S9 (2015).

Sahu, S., Cheng, R. A fast distance-based approach for determining the number of components in mixtures. *Can. J. Stat.* **31,** 3–22 (2003).

Vajda, E. J. Ket. *Languages of the World/Materials Volume 204.* Munich: Lincom Europa (2004).

Suppl. Fig. 4.1. Hierarchical clustering of 158 Siberian samples (labeled by sample ID), with ethnicity coded by color: dark-blue, Ket; orange, Selkup; brown, Nganasan; green: Enets, red, Evenk; pink, mixed. Unrelated individuals (88 in total) selected for downstream analyses are marked with red stars, and two sequenced Ket individuals with blue crosses. All individuals kept after removing cases of mixed ethnicity and proven relatives are separated by genetic distances larger than that marked by the dashed circle (except for the pair GRC13273898 and GRC13273899).

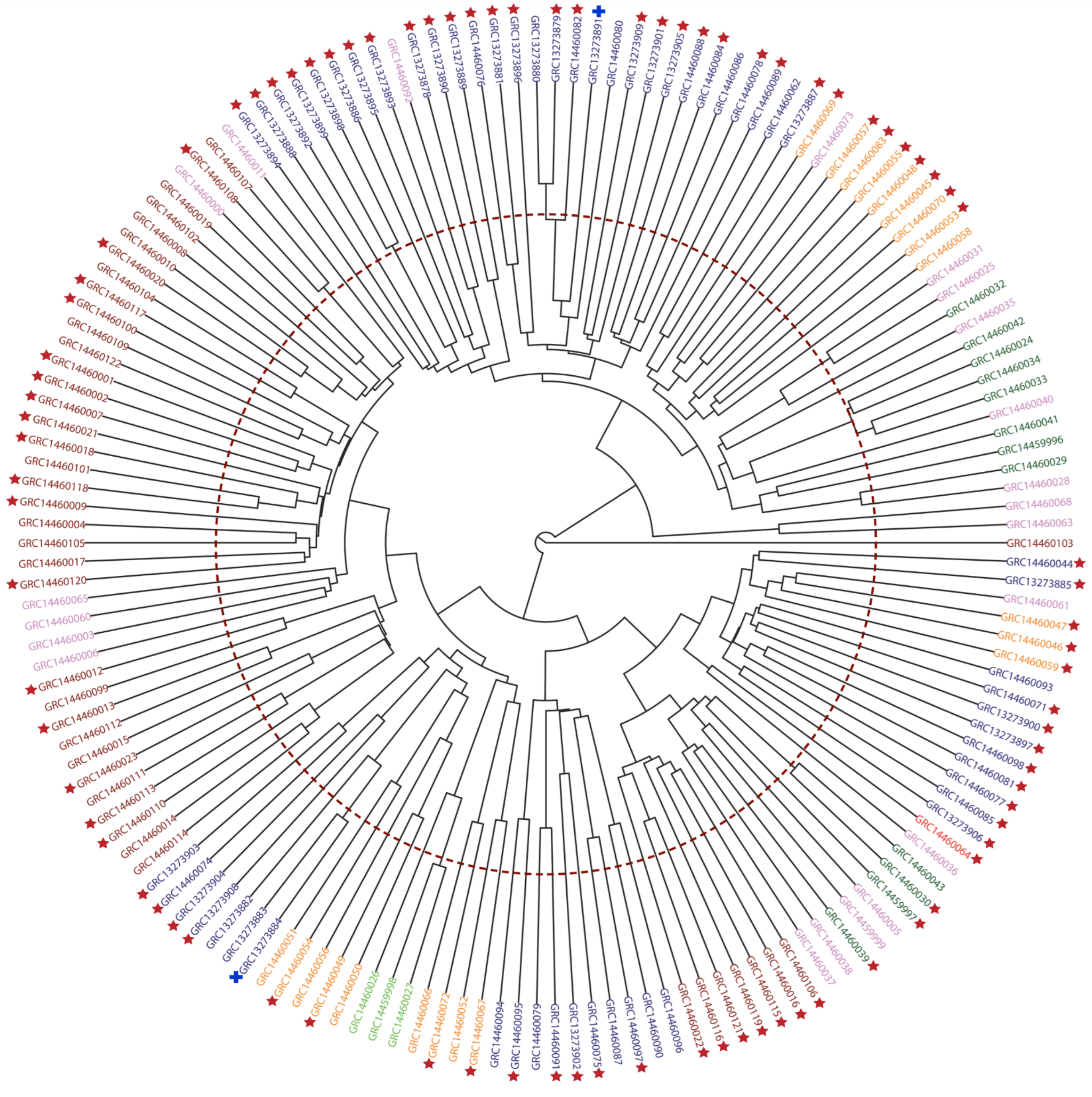

Suppl. Fig. 4.2. GPS predictions for individuals with self-reported ethnicities: Selkup (pink), Nganasan (cyan), Nenets (blue), Ket (green), Evenk (red), Enets (black). Size of the circle is proportional to the prediction uncertainty and points to individuals of mixed origin. The map was plotted using QGIS v.2.8.

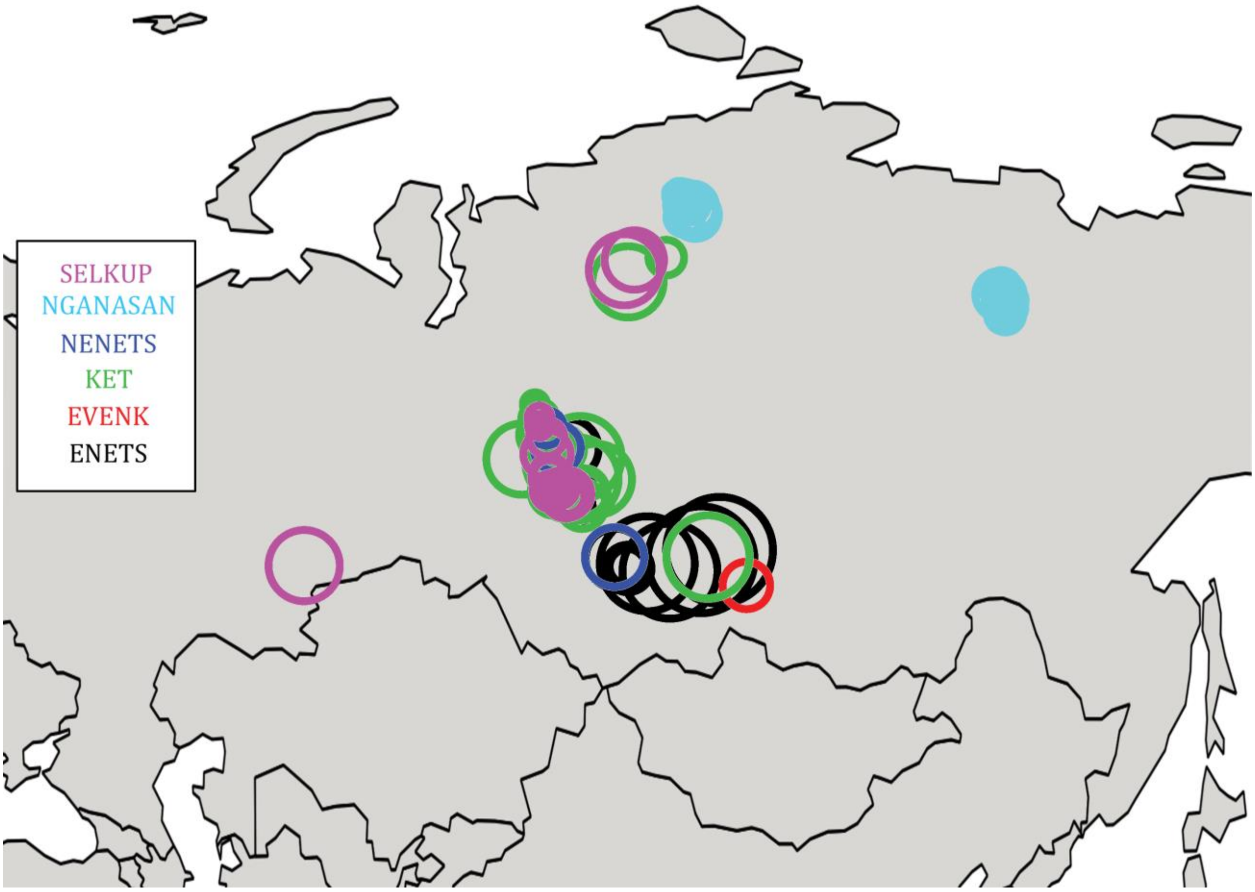

Suppl. Fig. 4.3. A. reAdmix analysis of individuals with self-reported Ket identity. The horizontal axis shows sample identifiers and the vertical axis shows a fraction of provenance attributed to a specific identity. All individuals except one were identified either as non-admixed or with unknown minor ancestry. 79% (45 out of 57) were identified as predominantly Ket. **B.** reAdmix analysis of individuals with self-reported Selkup and mixed Selkup identity. Only 57% of self-reported ethnic Selkups (12 out of 21) were identified as predominantly Selkup by reAdmix. Four individuals were identified as mixed. Individuals marked with the red arrow self-identified as the Ket-Selkup mix, and reAdmix identified them as Ket.

A.

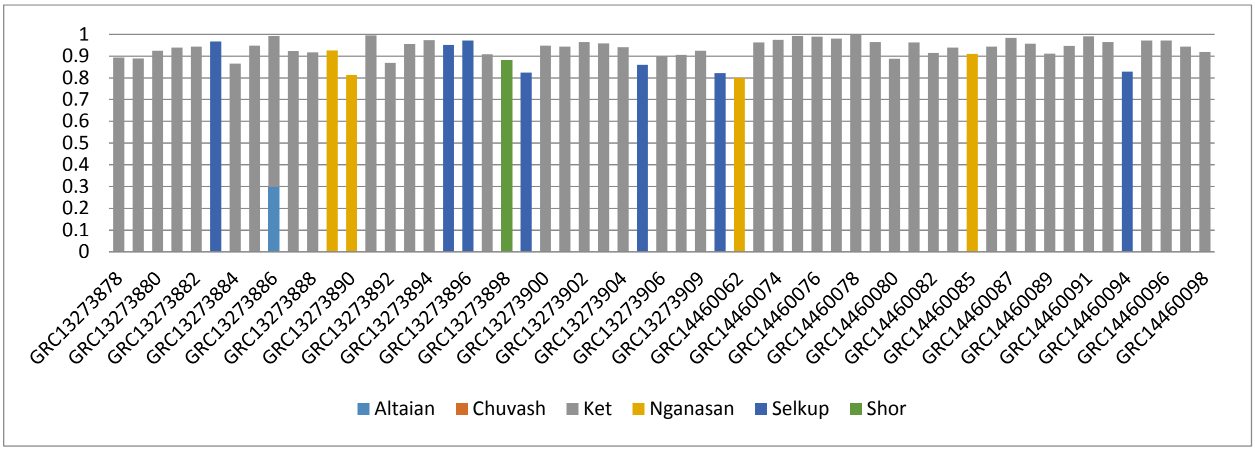

B.

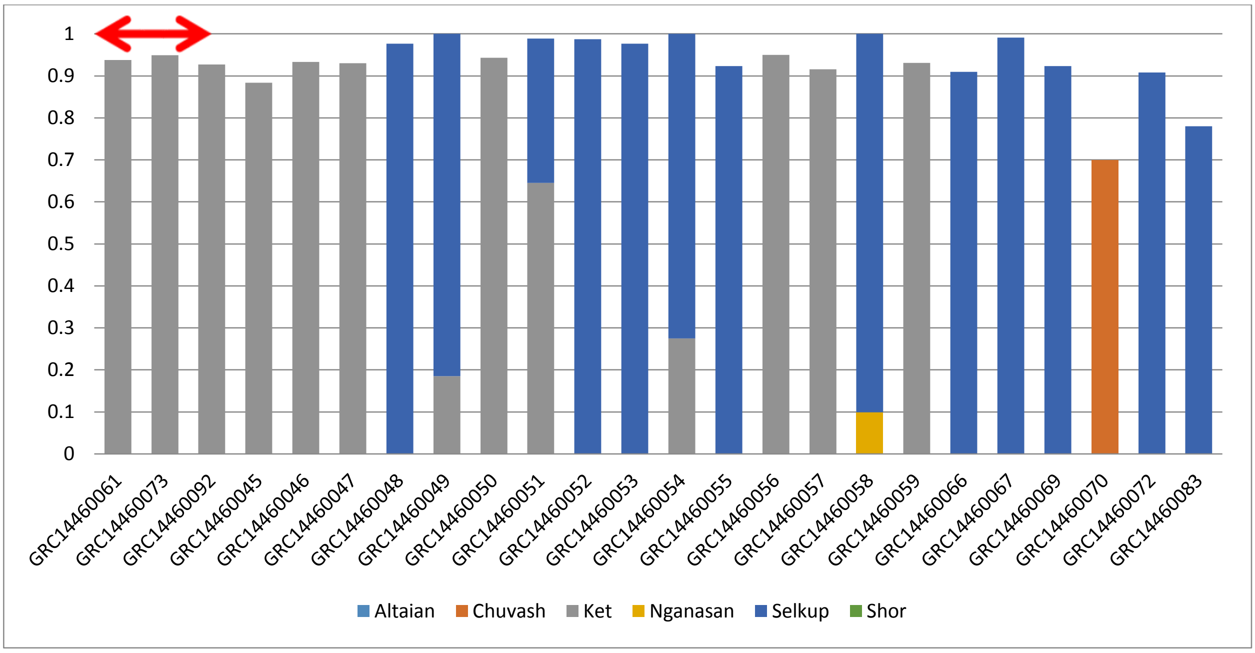

Suppl. Table 3. reAdmix and GPS predictions for individuals with self-reported Ket ancestry. Samples used for genome sequencing are marked in bold.

	reAdmix prediction						
**Sample ID**	**Altaian**	**Ket**	**Nganasan**	**Selkup**	**Shor**	**GPS prediction**	**GPS prediction accuracy, %**
GRC13273878	0	0.894	0	0	0	Ket	6.6
GRC13273879	0	0.889	0	0	0	Ket	3.0
GRC13273880	0	0.925	0	0	0	Ket	3.0
GRC13273881	0	0.94	0	0	0	Ket	2.6
GRC13273882	0	0.944	0	0	0	Ket	4.7
GRC13273883	0	0	0	0.967	0	Ket	2.6
**GRC13273884**	0	0.866	0	0	0	Ket	2.9
GRC13273885	0	0.948	0	0	0	Ket	2.7
GRC13273886	0.299	0.694	0	0	0	Ket	3.5
GRC13273887	0	0.923	0	0	0	Ket	2.3
GRC13273888	0	0.917	0	0	0	Ket	1.9
GRC13273889	0	0	0.926	0	0	Selkup	7.7
GRC13273890	0	0	0.813	0	0	Selkup	10.7
**GRC13273891**	0	0.995	0	0	0	Ket	2.5
GRC13273892	0	0.868	0	0	0	Selkup	4.2
GRC13273893	0	0.955	0	0	0	Ket	3.2
GRC13273894	0	0.973	0	0	0	Ket	1.9
GRC13273895	0	0	0	0.951	0	Selkup	3.8
GRC13273896	0	0	0	0.971	0	Selkup	3.0
GRC13273897	0	0.908	0	0	0	Ket	2.9
GRC13273898	0	0	0	0	0.88	Selkup	2.6
GRC13273899	0	0	0	0.825	0	Selkup	2.4
GRC13273900	0	0.948	0	0	0	Ket	1.2
GRC13273901	0	0.943	0	0	0	Ket	1.5
GRC13273902	0	0.965	0	0	0	Ket	1.8
GRC13273903	0	0.958	0	0	0	Ket	2.6
GRC13273904	0	0.941	0	0	0	Ket	3.0
GRC13273905	0	0	0	0.86	0	Ket	4.4
GRC13273906	0	0.903	0	0	0	Ket	2.7
GRC13273908	0	0.905	0	0	0	Ket	4.8
GRC13273909	0	0.925	0	0	0	Ket	2.3
GRC14460044	0	0	0	0.822	0	Selkup	1.8
GRC14460062	0	0	0.799	0	0	Dolgan	3.9
GRC14460071	0	0.963	0	0	0	Ket	1.5
GRC14460074	0	0.974	0	0	0	Ket	2.3
GRC14460075	0	0.993	0	0	0	Ket	1.7
GRC14460076	0	0.99	0	0	0	Ket	1.7
GRC14460077	0	0.98	0	0	0	Ket	1.0
GRC14460078	0	0.998	0	0	0	Ket	1.7
GRC14460079	0	0.964	0	0	0	Ket	2.0
GRC14460080	0	0.888	0	0	0	Ket	2.2
GRC14460081	0	0.963	0	0	0	Ket	1.4
GRC14460082	0	0.914	0	0	0	Ket	2.5
GRC14460084	0	0.939	0	0	0	Ket	3.2
GRC14460085	0	0	0.91	0	0	Khakas	5.6
GRC14460086	0	0.943	0	0	0	Ket	2.2
GRC14460087	0	0.984	0	0	0	Ket	1.9
GRC14460088	0	0.957	0	0	0	Ket	1.9
GRC14460089	0	0.912	0	0	0	Ket	2.1
GRC14460090	0	0.947	0	0	0	Ket	3.9
GRC14460091	0	0.991	0	0	0	Ket	1.7
GRC14460093	0	0.965	0	0	0	Ket	1.4
GRC14460094	0	0	0	0.829	0	Selkup	3.5
GRC14460095	0	0.971	0	0	0	Ket	2.0
GRC14460096	0	0.972	0	0	0	Ket	2.1
GRC14460097	0	0.944	0	0	0	Ket	3.1
GRC14460098	0	0.919	0	0	0	Ket	2.7

5. ADMIXTURE analysis

We combined the GenoChip array data with published SNP array datasets to produce a worldwide dataset of 90 populations and 1,624 individuals. The intersection dataset, containing 32,189 SNPs (Suppl. Table 1), was analyzed with the ADMIXTURE software (Alexander et al. 2009) (Fig. 1), selecting the best of 100 iterations and using 10-fold cross-validation criterion. In contrast to some of the previous studies of SNP array data (Rasmussen et al. 2010, Seguin-Orlando et al. 2014, Raghavan et al. 2015), a unique admixture component, characteristic of the Ket and Selkup individuals, appeared at K≥11 components, which is a low value for a worldwide dataset (Fig. 1A). That discrepancy can be explained by differences in marker selection. The GenoChip array includes a high percentage of ancestry-informative markers that were chosen to maximize F_{ST} (Elhaik 2012, Elhaik et al. 2013). Kets and Selkups were previously modeled on a worldwide dataset as a mixture of the North European and Siberian components at K up to 15 (Seguin-Orlando et al. 2014, Raghavan et al. 2015). Similarly, Selkups were modeled on a large worldwide dataset as a mixture of the North European and Siberian components at K up to 20 (Lazaridis et al. 2014, Haak et al. 2015), and Rasmussen et al. (2010) reached similar conclusions using the Eurasian-American dataset at K up to 10. However, in Fedorova et al. (2013) a unique 'Ket' admixture component appeared in Kets (4 individuals in the study) and in Selkups at K≥10 on a Eurasian-American dataset of 758 individuals from 55 populations. At the lower values of K, Kets and Selkups were again modeled as a mixture of the North European and Siberian components (Fedorova et al. 2013). And a similar result was obtained in Yunusbayev et al. (2015): an admixture component characteristic of Kets (6 individuals in the study), Nenets, and Nganasans appeared at K≥8 on a Eurasian dataset of 1,444 individuals from 93 populations.

In order to verify and explain the geographic distribution of the 'Ket' admixture component, we have performed ADMIXTURE (Alexander et al. 2009) analysis on two additional datasets, differing in populations (Suppl. Table 2) and marker selection (Suppl. Table 1). An admixture component with a geographical distribution closely resembling that discussed above was revealed in all datasets (Suppl. Table 4), however it reached its global peak either in Uralic-speaking Khanty or in Turkic-speaking Tubalars, populations not included into the GenoChip-based dataset. This component was also prominent in Selkups, Kets, in Turkic-speaking Altaians and Tuvinians, and in Uralic-speaking Nenets and Mansi.

A worldwide dataset based on the Ket genomes and Illumina SNP array data (103,495 SNPs, 105 populations and 2,552 individuals, Suppl. Table 1) contained data for Uralic-speaking Khanty and Nenets, omitted from the GenoChip-based dataset (Suppl. Table 2) due to a very low marker overlap. Probably due to the significantly increased dataset size (2,552 individuals vs 1,624 in the GenoChip-based dataset), we observed no minimum on the graph of cross-validation errors, and K=20 was chosen for the final analysis on this dataset since K=19 was used for the GenoChip-based dataset (Fig. 1). An admixture component with a geographical distribution closely resembling that of the 'Ket' component (in the GenoChip-based dataset) was revealed at K≥13, however it reached its global peak (~98%) in Khanty, being also prominent in Selkups, reference Ket individuals, Nenets, and Kets from

the present study (Suppl. Figs. 5.1-5.3). Similar to the GenoChip-based dataset, secondary peaks of the 'Khanty-Ket' component were observed in the Volgo-Ural region, in South Siberia (e.g., up to ~11% in Tuvinians not included in the GenoChip-based dataset), in East Siberia, in Central and South Asia (e.g., up to ~6% in Burusho not included in GenoChip-based dataset), but not in the North Caucasus. The Saqqaq genome was not included into this dataset.

The dataset based on the HumanOrigins SNP array (Lazaridis et al. 2014) overlapped with the Ket genomic data showed a somewhat different pattern in admixture analysis (Suppl. Figs. 5.4-5.6). The minimum of cross-validation errors was reached at K=17 (Suppl. Fig. 5.5). In agreement with the results by Lazaridis et al. (2014), Kets and Selkups were modeled as a mixture of Siberian and North European components up to K=22. At K=23 a component with the characteristic geographical distribution appeared, reaching a global maximum of ~100% in Tubalars, a population of the Altai region not included into the previous datasets (Suppl. Table 2). This component was also prominent (from ~25% to ~14%) in Altaians, Selkups, Kets, Tuvinians, and in Uralic-speaking Mansi. In addition to South Siberia, peaks of the 'Tubalar' component were observed in Central and South Asia, in East Siberia, in the Volgo-Ural region, in the North Caucasus, and, remarkably, in Aleutians and in the Mal'ta (9.4%) and Saqqaq (6.3%) ancient genomes (Suppl. Fig. 5.4).

The Siberian component reached its global peak (~100%) in Nganasans in all publications (Rasmussen et al. 2010, 2014, Fedorova et al. 2013, Lazaridis et al. 2014, Seguin-Orlando et al. 2014, Allentoft et al. 2015, Haak et al. 2015, Raghavan et al. 2015), and the North European component, in populations of the Baltic region and in ancient genomes of west European hunter-gatherers (Lazaridis et al. 2014, Seguin-Orlando et al. 2014, Allentoft et al. 2015, Haak et al. 2015,). It should be noted that Nganasans analyzed here, both from the present study and from previous publications (Rasmussen et al. 2010, Reich et al. 2012, Lazaridis et al. 2014), had up to ~100% of the Siberian admixture component in all datasets, demonstrating a remarkable consistency of results (however, see a unique component shared by Kets, Nenets and Nganasans at K from 8 to 10 in the analysis by Yunusbayev et al. 2015).

In summary, our ADMIXTURE analysis of three datasets and two previous studies using Illumina array datasets (Fedorova et al. 2013, Yunusbayev et al. 2015) have revealed an ancestral component characteristic of Uralic-speaking people of Western Siberia (Khanty, Nenets, Enets, Selkup) and of Kets, which we term here 'Ket-Uralic component'. This component occurs at lower levels (Suppl. Table 4) in other Uralic speakers of Russia (Komi, Mari, Mordovians, Udmurts, etc) or in populations known to be closely related to Uralic speakers (Chuvashes and Tatars) (Johanson 2010), in South Siberia (the Altai) and in Central Asia. High levels of the Ket-Uralic admixture component in South Siberia correlate with the former presence of extinct Yeniseian- and Samoyedic-speaking ethnic groups there (Vajda 2004). It remains to be elucidated whether the observed geographic distribution of this ancestral component was formed by population movements of forest and tundra hunter-gatherers and steppe nomadic groups within the last two millennia, or is a hallmark of far more ancient events. However, the most intriguing is the appearance of the Ket-Uralic component in the Saqqaq Paleo-Eskimo (~4,000 YBP): at a low level of 6.3-7.2%, but consistently in both datasets containing this individual (Suppl. Table 4).

Since the Ket-Uralic admixture component appears almost exclusively in populations having both the Mal'ta (ANE) ancestry and the Siberian ancestry (Suppl. Information, Section 7), it may be tentatively viewed as a correlate of these two ancestries combined. However, the HumanOrigins-based dataset showed that among all admixture components at K=23 in 102 Eurasian populations, the North European component (with a maximum in WHG individuals) correlated best with the statistic f_3(Yoruba; Mal'ta, X): the Pearson's correlation coefficient was 0.82 (*p*-value 5.7×10^{-26}) vs. 0.13 (*p*-value 0.193) and 0.15 (*p*-value 0.132) for the Ket-Uralic component. On the other hand, the HumanOrigins-based dataset is probably not the best model for the Ket-Uralic admixture component, in contrast to the GenoChip- and Illumina-based datasets (Fig 1, Fedorova et al. 2013, Yunusbayev et al. 2015): i/ the Ket-Uralic component appears at K=23 only; ii/ its geographic distribution is skewed towards the Altai region; iii/ it demonstrates the worst correlation with haplogroups U4 and Q (Suppl. Table 8). Thus, the conclusion that there is no correlation between ANE ancestry and the Ket-Uralic component appears premature.

References

Alexander, D. H., Novembre, J., Lange, K. Fast model-based estimation of ancestry in unrelated individuals. *Genome Res.* **19,** 1655–1664 (2009).

Allentoft, M. E. *et al.* Population genomics of Bronze Age Eurasia. *Nature* **522,** 167–172 (2015).

Elhaik, E. Empirical distributions of F(ST) from large-scale human polymorphism data. *PLoS ONE* **7,** e49837 (2012).

Elhaik, E. *et al.* The GenoChip: a new tool for genetic anthropology. *Genome Biol. Evol.* **5,** 1021–1031 (2013).

Fedorova, S. A. *et al.* Autosomal and uniparental portraits of the native populations of Sakha (Yakutia): implications for the peopling of Northeast Eurasia. *BMC Evol. Biol.* **13,** 127 (2013).

Haak, W. *et al.* Massive migration from the steppe was a source for Indo-European languages in Europe. *Nature* **522,** 207–211 (2015).

Johanson, L. Turkic language contacts. *The Handbook of Language Contact*, ed. Hickey, R. Oxford: Wiley-Blackwell, 652–672 (2010).

Lazaridis, I. *et al.* Ancient human genomes suggest three ancestral populations for present-day Europeans. *Nature* **513,** 409–413 (2014).

Raghavan, M. *et al.* Genomic evidence for the Pleistocene and recent population history of Native Americans. *Science* doi: 10.1126/science.aab3884 (2015).

Rasmussen, M. *et al.* Ancient human genome sequence of an extinct Palaeo-Eskimo. *Nature* **463,** 757–762 (2010).

Rasmussen, M. *et al.* The genome of a Late Pleistocene human from a Clovis burial site in western Montana. *Nature* **506,** 225–229 (2014).

Reich, D. et al. Reconstructing Native American population history. Nature 488, 370–374 (2012).

Seguin-Orlando, A. *et al.* Genomic structure in Europeans dating back at least 36,200 years. *Science* **346,** 1113–1118 (2014).

Vajda, E. J. Ket. *Languages of the World/Materials Volume 204.* Munich: Lincom Europa (2004).

Yunusbayev, B. *et al.* The genetic legacy of the expansion of Turkic-speaking nomads across Eurasia. *PLoS Genet.* **11,** e1005068 (2015).

Suppl. Table 4. Populations with >5% of the Ket-Uralic admixture component in two datasets. Maximum values of the component encountered in each population are shown. Ten-fold cross-validation was performed, and average cross-validation (CV) errors and their standard deviations (SD) are shown for respective values of K.

dataset	**GenoChip + Illumina arrays**		**Ket genomes + Illumina arrays**		**Ket genomes + HumanOrigins array**	
K analyzed	19		20		23	
CV error ± SD	0.56462 ± 0.00035		0.56198 ± 0.00026		0.49029 ± 0.00082	
K, lowest CV error	20		?		17	
CV error ± SD	0.56444 ± 0.00036		N/A		0.48977 ± 0.00024	
	population	Ket-Uralic component, %	population	Ket-Uralic component, %	population	Ket-Uralic component, %
	Ket present	99.9	Khanty	98.2	Tubalar	100.0
	Ket	91.5	Selkup	58.4	Altaian	24.9
	Selkup present	81.5	Ket	46.1	Selkup	19.9
	Selkup	48.5	Nenets	41.0	Ket	18.1
	Enets	22.6	Ket present	37.5	Tuvinian	15.0
	Shor	21.6	Mari	22.0	Mansi	14.1
	Khakas	21.6	Shor	20.1	Kyrgyz	10.5
	Altaian	20.5	Chuvash	17.9	Yakut	9.9
	Teleut	15.4	Khakas	15.8	Mal'ta	9.4
	Mari	14.3	Altaian	15.8	Kalmyk	9.3
	Kazakh	14.0	Teleut	14.6	Uygur	7.5
	Kyrgyz	12.3	Tatar	11.6	Burusho	7.4
	Chuvash	12.2	Tuvinian	10.9	Hazara	7.4
	Yakut	10.1	Nganasan	10.8	Turkmen	7.1
	Dolgan	9.5	Uzbek	9.2	Uzbek	7.0
	Evenk	9.4	Russian	8.6	Tajik Pamiri	6.8
	Buryat	9.3	Kazakh	8.5	Aleut	6.5
	Mongol	8.6	Mordovian	7.4	Dolgan	6.4
	Nganasan present	8.4	Kyrgyz	7.0	Saqqaq	6.3
	Tatar	8.2	Burusho	5.8	Chuvash	5.5
	Uzbek	8.1	Tajik	5.6	Russian	5.5
	Nogai	8.0	Turkmen	5.6	Yukaghir	5.1
	Saqqaq	7.2	Buryat	5.5		
	Russian	7.1	Chukchi	5.3		
	Tajik	7.1	Hazara	5.2		
	Even	6.5	Lezgin	5.1		
	Mordovian	6.5				
	Ingush	6.2				
	Turkmen	5.4				
	Finnish	5.4				
	Balkar	5.4				
	Indian	5.1				

5.1. Admixture coefficients plotted for dataset 'Ket genomes + Illumina arrays'. Abbreviated names of admixture components are shown on the left as follows: SAM, South American; ESK, Eskimo (Beringian); SEA, South-East Asian; SIB, Siberian; NEU, North European; CAU, Caucasian; SEU, South European; SAS, South Asian; OCE, Oceanian; AFR, African. The Ket-Uralic ('Khanty-Ket') admixture component appears at K≥13, and admixture coefficients are plotted for K=4, 12, 13, and 20. Only populations containing at least one individual with >5% of the Ket-Uralic component at K=20 are plotted, and individuals are sorted according to values of the Ket-Uralic component. Admixture coefficients for four reference Kets and two Ket individuals from this study are shown separately on the left.

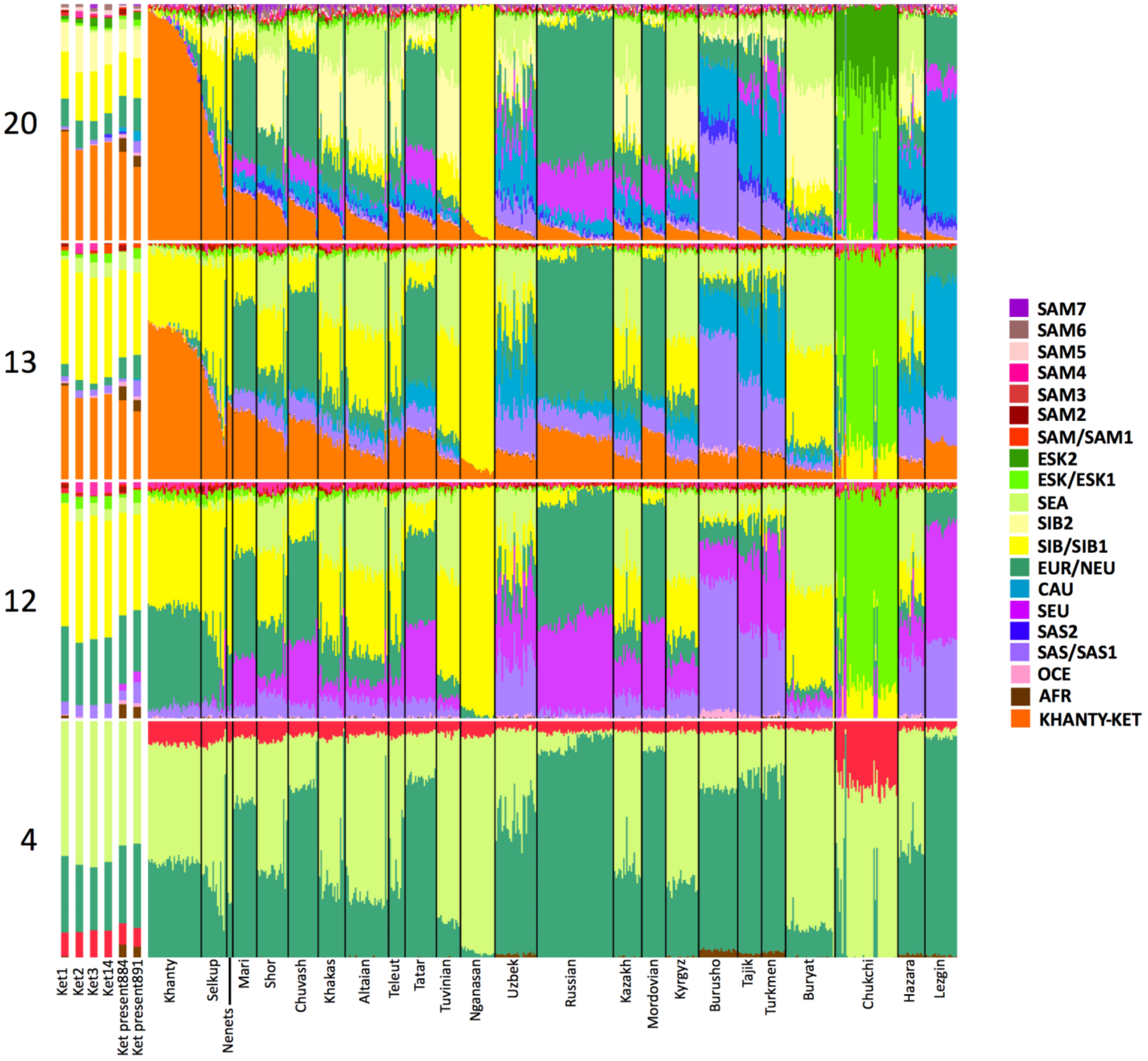

5.2. Average cross-validation (CV) error graph with standard deviations plotted, dataset 'Ket genomes + Illumina arrays'. Ten-fold cross-validation was performed.

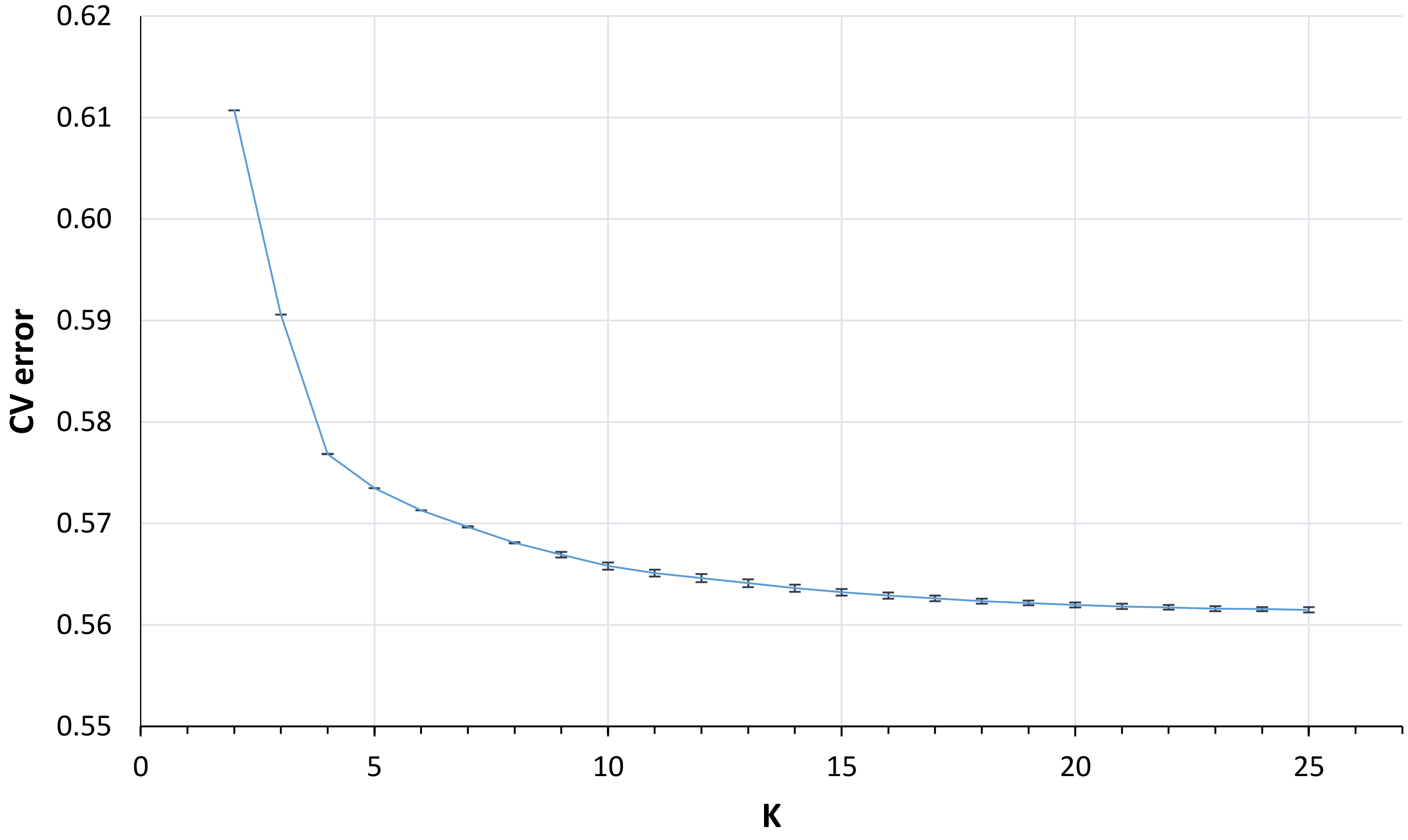

5.3. Color-coded values of the Ket-Uralic admixture component at K=20 plotted on the world map using QGIS v.2.8, dataset 'Ket genomes + Illumina arrays'. Maximum values in each population are taken, and only values >5% are plotted. Top five values of the component are shown in the bottom left corner.

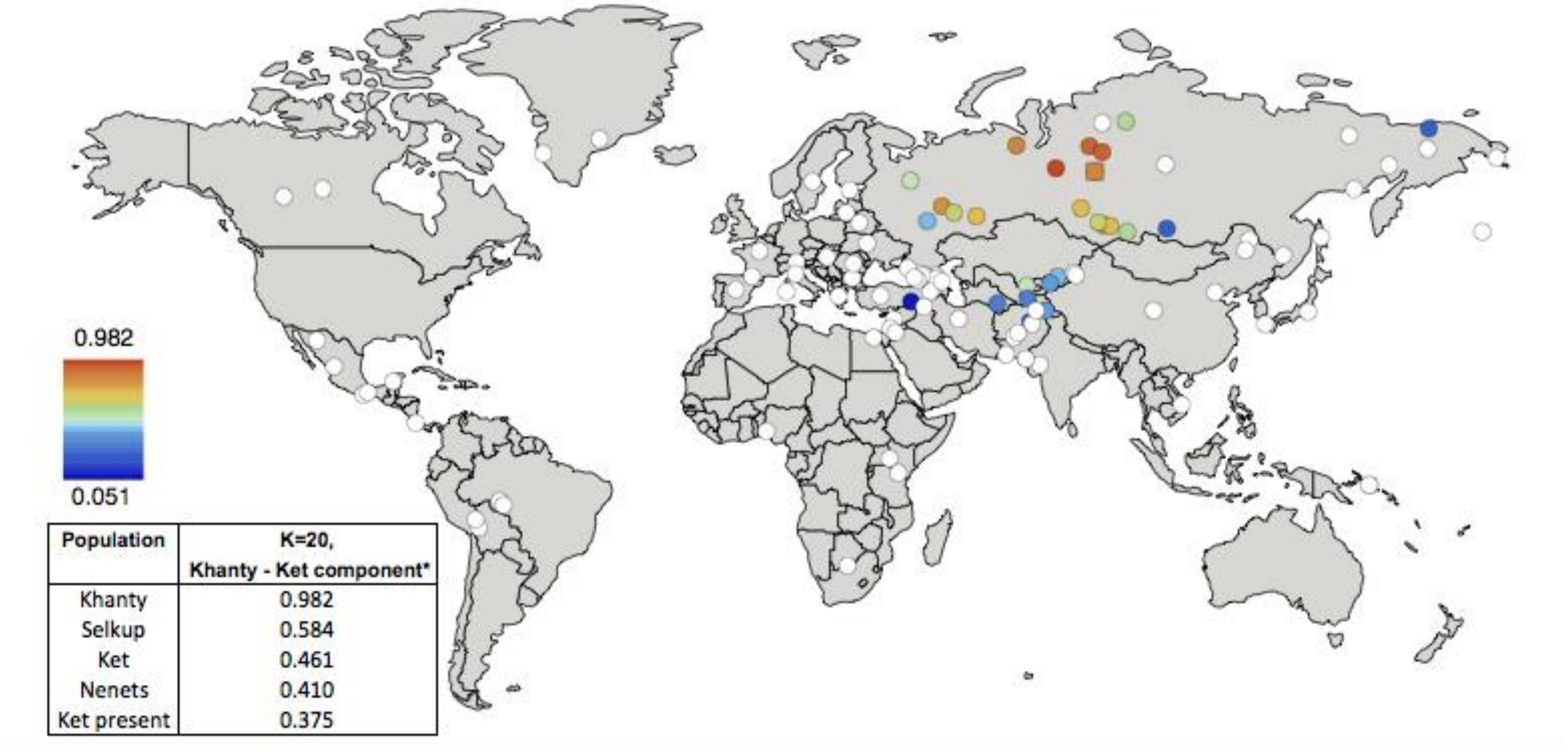

5.4. Admixture coefficients plotted for dataset 'Ket genomes + HumanOrigins array'. Abbreviated names of admixture components are shown on the left as follows: SAM, South American; NAM, North American; ESK, Eskimo (Beringian); SEA, South-East Asian; SIB, Siberian; NEU, North European; SEU, South European; ME, Middle Eastern; SAS, South Asian; OCE, Oceanian; AFR, African. The Ket-Uralic ('Tubalar') admixture component appears at K≥23, and admixture coefficients are plotted for K=4, 17 (demonstrating the lowest average cross-validation error), 22, and 23. Only populations containing at least one individual with >5% of the Ket-Uralic component at K=23 are plotted, and individuals are sorted according to values of the Ket-Uralic component. Admixture coefficients for the Mal'ta and Saqqaq ancient genomes are shown separately on the right, and for two Ket individuals from this study – on the left.

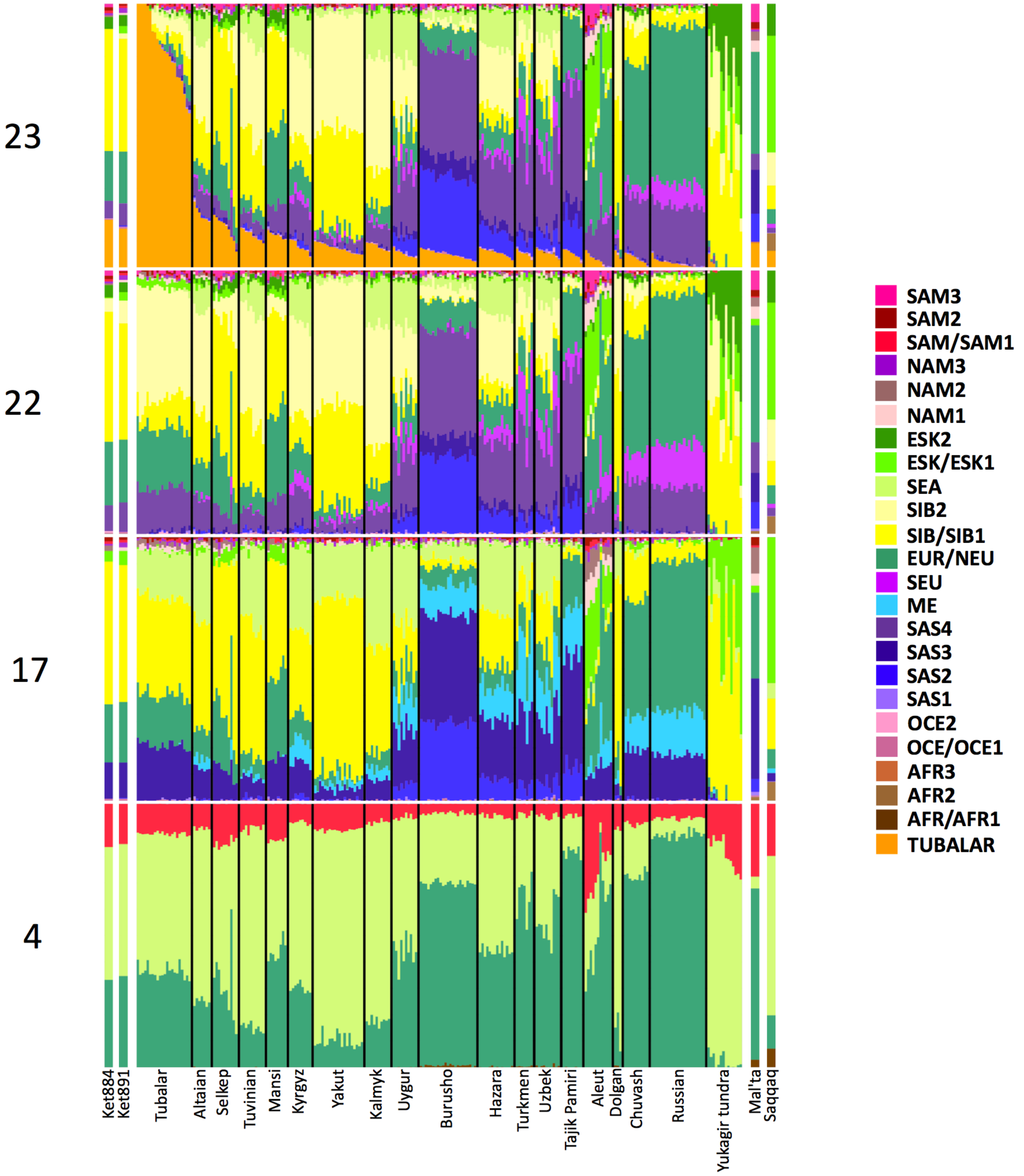

5.5. Average cross-validation (CV) error graph with standard deviations plotted, dataset 'Ket genomes + HumanOrigins array'. Ten-fold cross-validation was performed. The graph has a minimum at K=17.

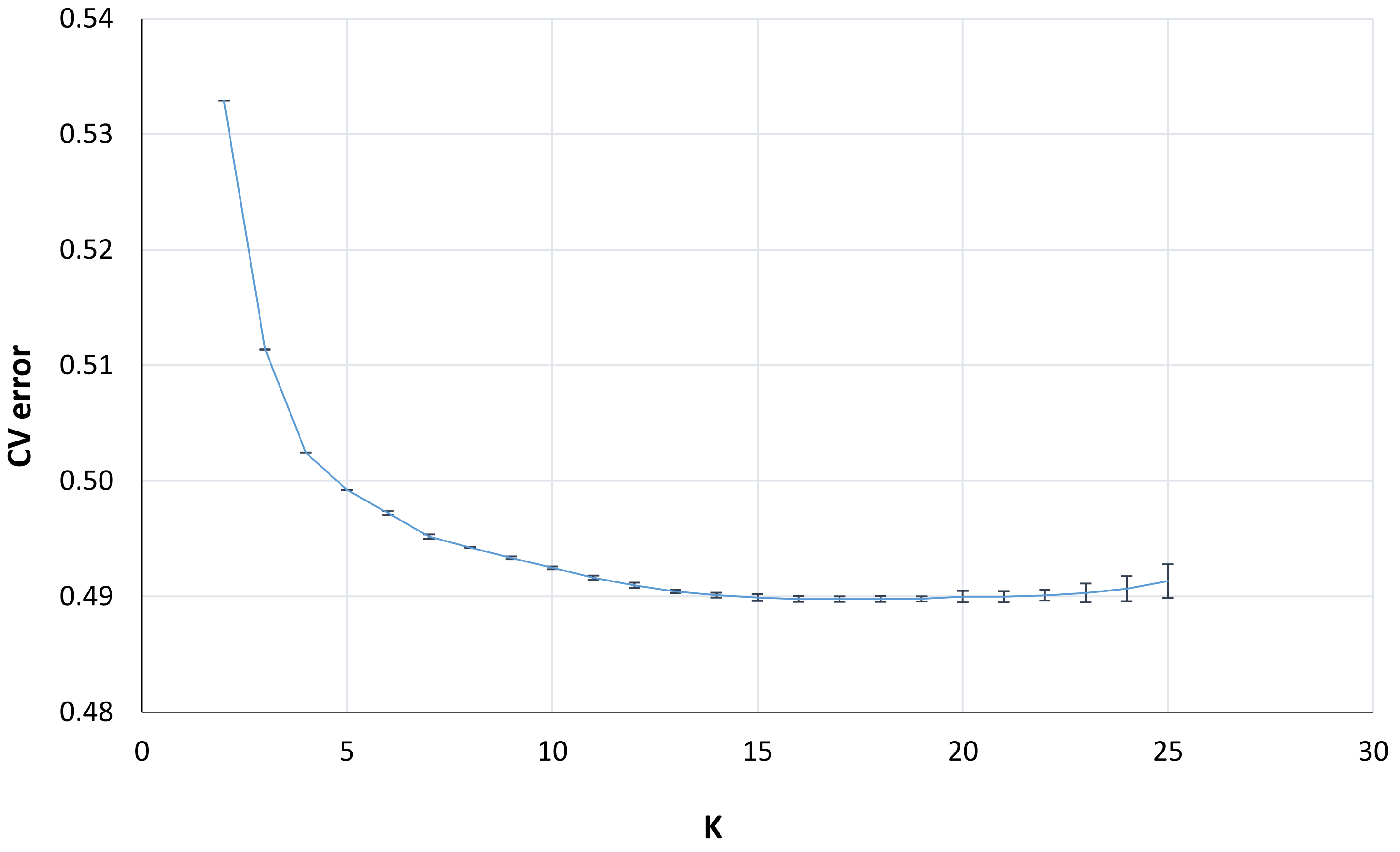

5.6. Color-coded values of the Ket-Uralic admixture component at K=23 plotted on the world map using QGIS v.2.8, dataset 'Ket genomes + HumanOrigins array'. Maximum values in each population are taken, and only values >5% are plotted. Top five values of the component are shown in the bottom left corner, and the value for Saqqaq is shown on the map.

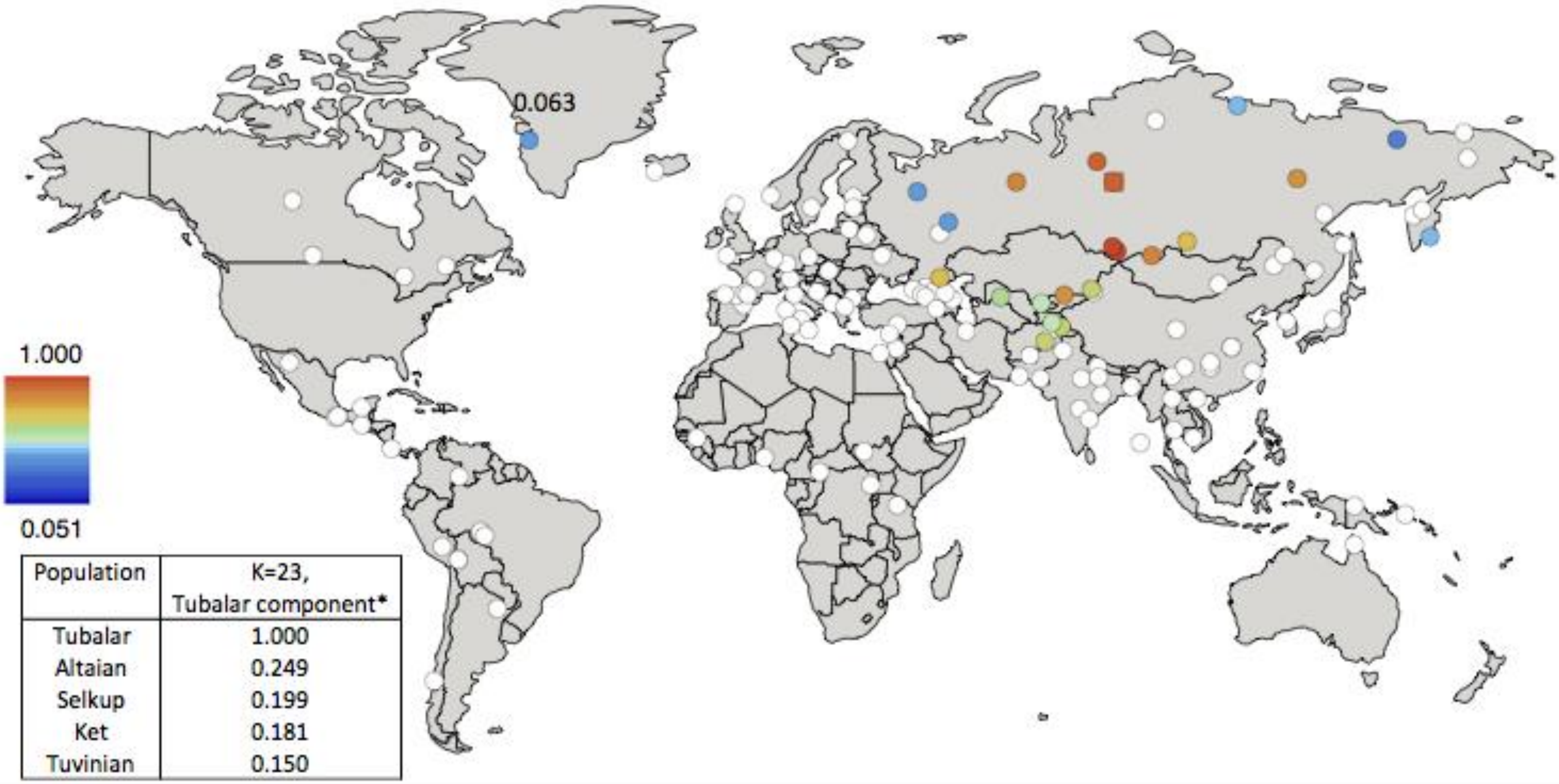

Population	K=23, Tubalar component*
Tubalar	1.000
Altaian	0.249
Selkup	0.199
Ket	0.181
Tuvinian	0.150

6. Principal component analysis

Principal component analysis (PCA) was performed on two datasets using SmartPCA. Results for the GenoChip-based SNP array dataset (~32,000 SNPs) are presented in Suppl. Figs. 6.1-6.6. In the PC1 vs PC2 plot (Suppl. Fig. 6.1), Ket, Selkup, and Enets individuals were reasonably positioned between the European and East Asian clusters, together with many Siberian and Central Asian populations. Nganasans formed a cluster with Evenks, Evens, Yakuts, Dolgans, Yukaghirs, Nivkhs, and Koryaks, located closer to the East Asian cluster. Most of the Ket individuals, i.e. two reference individuals and individuals from the present study, formed a tight cluster, however there were several outliers (see a zoomed-in version of the plot in Suppl. Fig. 6.2). Distributions for the Ket and Selkup populations were very similar. Turkic-speaking South Siberian and Central Asian populations, namely Khakases, Shors, Altaians, Teleuts, Kazakhs, and Kyrgyz, were located close to the Ket-Selkup cluster in the PC1 vs PC2 space, but did not overlap with it much. The same applies to Enets, one Koryak individual, one Evenk, and several Evens. Notably, Enets, Shors, Khakases, Altaians, Teleuts, Kazakhs, and Kyrgyz demonstrated a high percentage of the Ket-Uralic admixture component (see Results and Discussion), appearing in the GenoChip-based dataset at K≥11 and reaching its maximum percentage in Kets (Fig. 1A). These populations were ranked high, just below Kets and Selkups, in the list of populations sorted according to maximum Ket-Uralic component percentage in a given population (Suppl. Table 4), which shows that the ADMIXTURE and PCA results are in agreement. Notice, that Mari, having a comparable level of the Ket-Uralic component (up to 14.3%), were located closer to the European cluster on the PC plot.

Several Chukchi and one Eskimo individual were located on the other side of the Ket-Selkup cluster. Position of the ancient individuals (Clovis, Saqqaq, and La Braña) in this plot was probably affected by their high percentage of missing markers (Suppl. Table 1). The Ket-Selkup cluster and its neighbors remained generally the same in the PC3 vs PC4 space (Suppl. Figs. 6.4 and 6.5). However, in this case Koryaks and a few Even and Yukaghir individuals overlapped with the Ket-Selkup cluster.

Comparison of samples collected in this study and published samples of respective populations is shown in Suppl. Figs. 6.3 (PC1 vs PC2) and 6.6 (PC3 vs PC4). In both plots, distributions overlapped for: two reference Kets and 46 individuals from this study; seven reference Selkups and fifteen individuals from this study; 22 reference Nganasans and 24 individuals from this study, suggesting that population samples from the present study were generally similar to the published ones. However, in the PC3 vs PC4 plot five out of seven reference Selkup individuals lay outside the cluster of Selkups from this study. The difference of two Selkup population samples is further manifested in the ADMIXTURE analysis (Fig. 1A) and is probably explained by close proximity of our Selkup sampling area to the Ket settlements (see Results and Discussion)

PCA results based on the Ket genomes sequenced in this study and the HumanOrigins SNP array dataset (~196,000 SNPs) are presented in Suppl. Figs. 6.7-6.8 and Figs. 4A,B. In the PC1 vs PC2 plot Kets and 5 Selkup individuals were located between Altaians, Kyrgyz, Tubalars, and an Even individual on one side, and Aleutians, Chipewyan, Cree, and Ojibwa Native North American individuals on the

other side (Suppl. Fig. 6.8). The PC3 vs PC4 plot showed Kets in proximity to Selkups, Mansi, Tubalars, some Yukaghir and Even individuals (Fig. 5B). Tubalars, Selkups, Altaians, Kets, Mansi, and Kyrgyz share the Ket-Uralic admixture component, appearing in this dataset at $K \geq 23$ and reaching its maximum percentage in Tubalars (Suppl. Figs. 5.4-5.6). The Ket-Uralic component appeared at a level of >10% in at least one individual in each of these populations. The Saqqaq ancient genome was positioned between the above-mentioned samples and Yukaghirs, Koryaks, Itelmens, and Chukchi on the other side, that is between Siberian and Beringian (in this case Chukotkan and Kamchatkan) populations. According to the Euclidean distances calculated between individuals in the multi-dimensional space of ten principal components, Ket is the closest population to Saqqaq, followed by Nganasans, Selkups, Yukaghirs, Eskimos, and others (Fig. 4).

6.1. PCA, dataset 'GenoChip + Illumina arrays', PC1 vs PC2. African populations are not shown. Populations are color-coded by geographical region or language affiliation (in the case of Siberian and Central Asian populations), and most relevant populations are differentiated by marker shapes. Ancient genomes are shown in black.

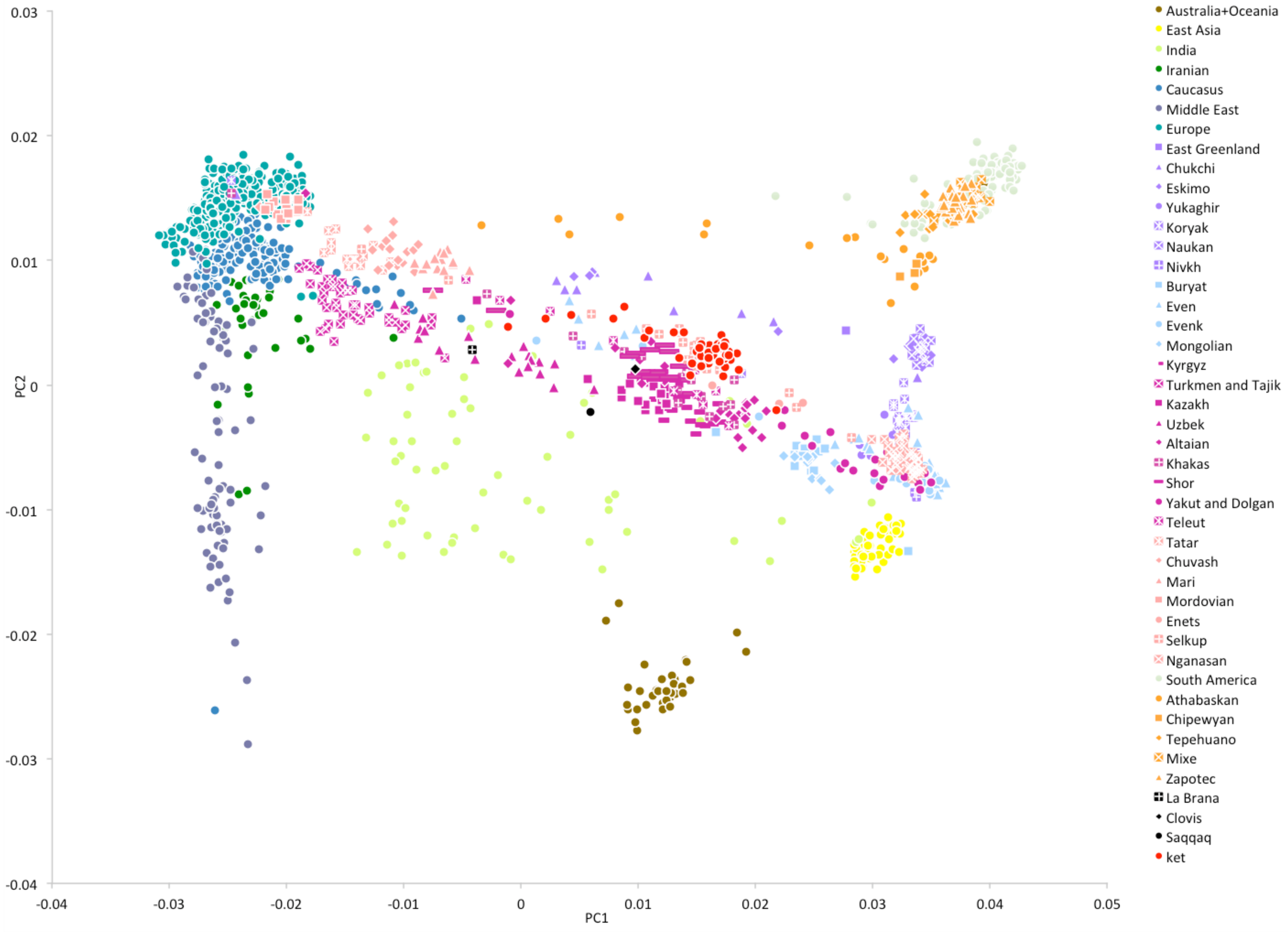

6.2. PCA, dataset 'GenoChip + Illumina arrays', PC1 vs PC2, zoom on the Ket population. Ancient genomes are shown in black.

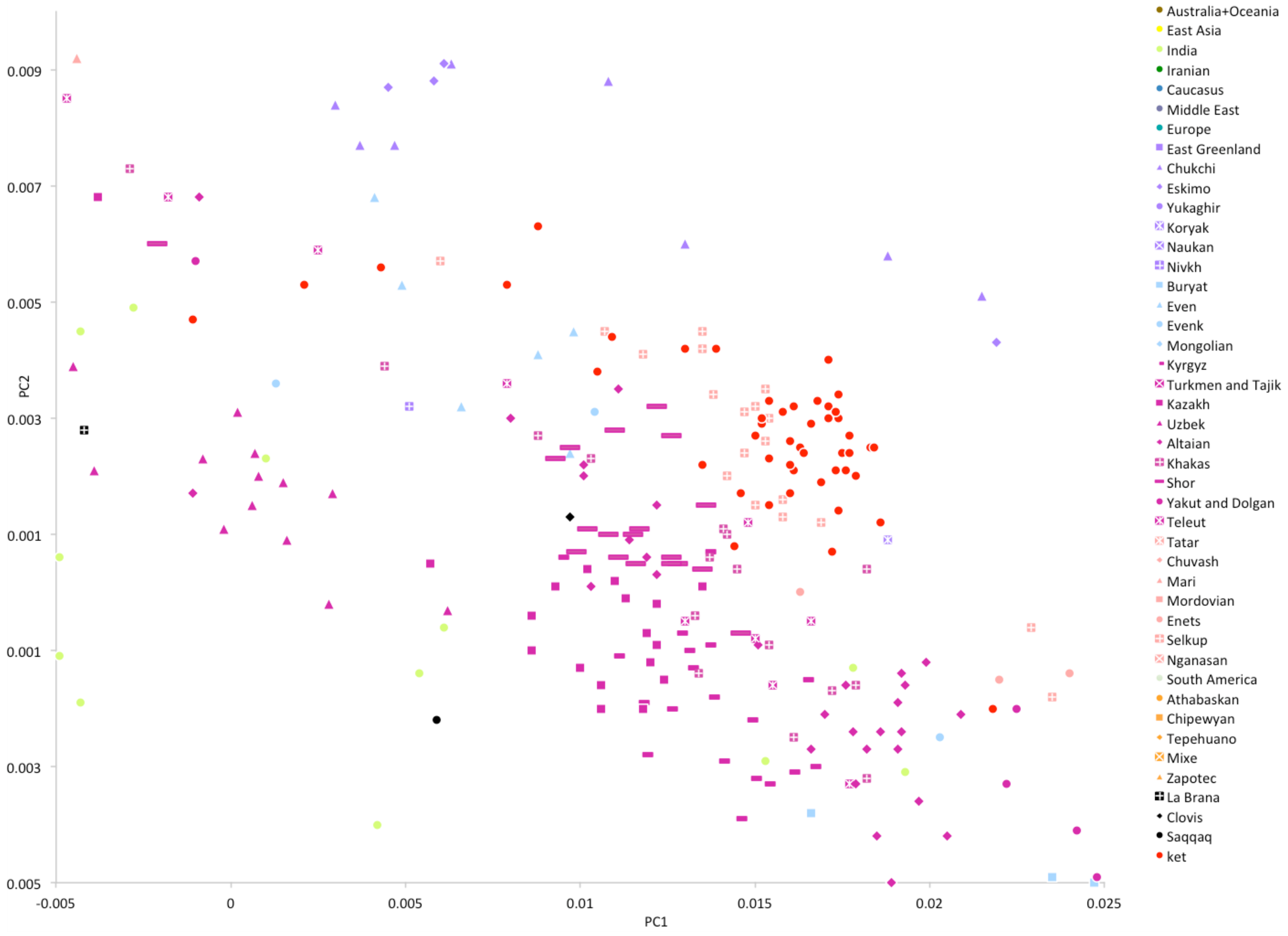

6.3. PCA, dataset 'GenoChip + Illumina arrays', PC1 vs PC2, only samples collected in the present study and published samples from respective populations are shown.

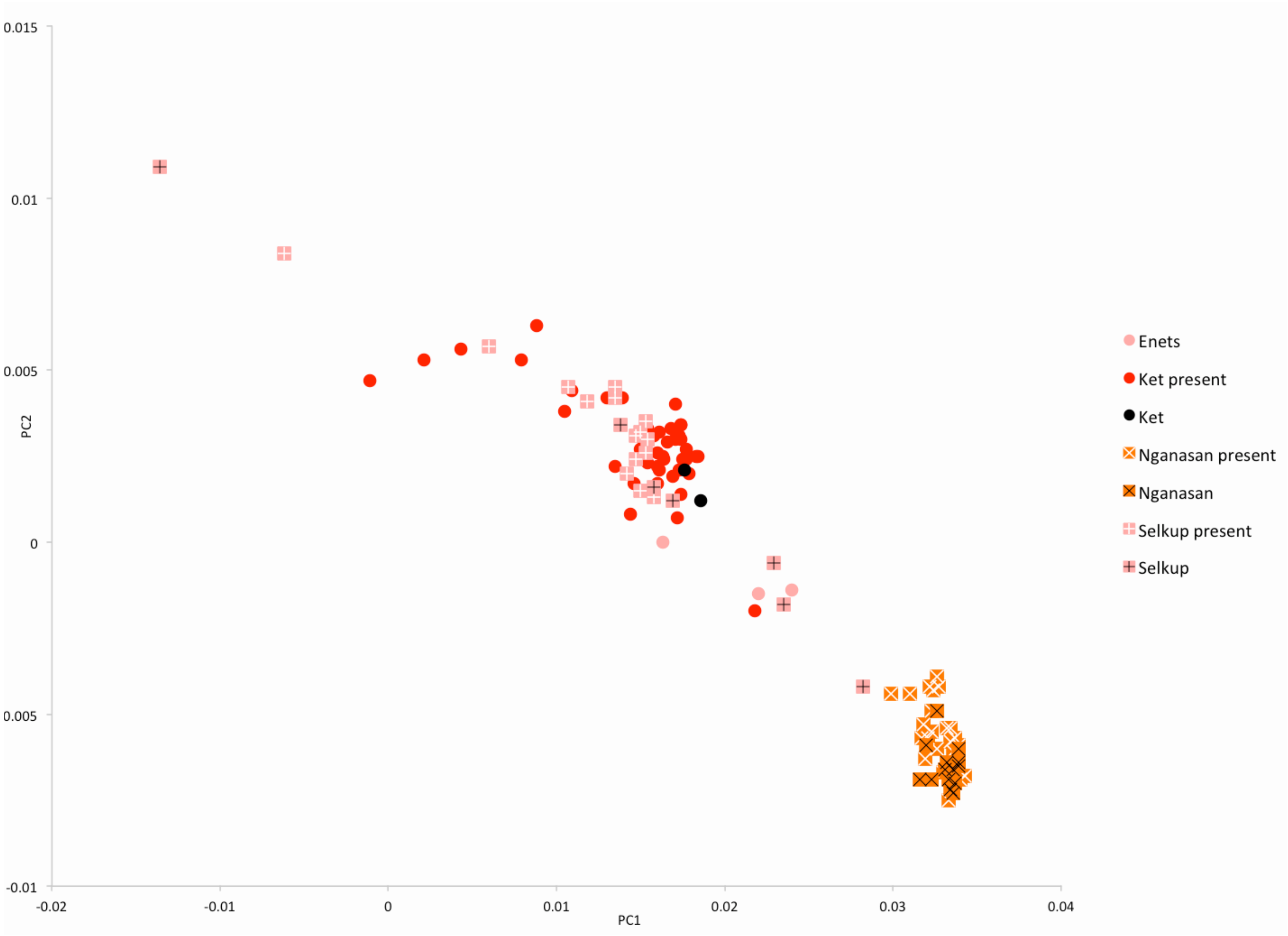

6.4. PCA, dataset 'GenoChip + Illumina arrays', PC3 vs PC4. African populations are not shown. Populations are color-coded by geographical region or language affiliation (in the case of Siberian and Central Asian populations), and most relevant populations are differentiated by marker shapes. Ancient genomes are shown in black.

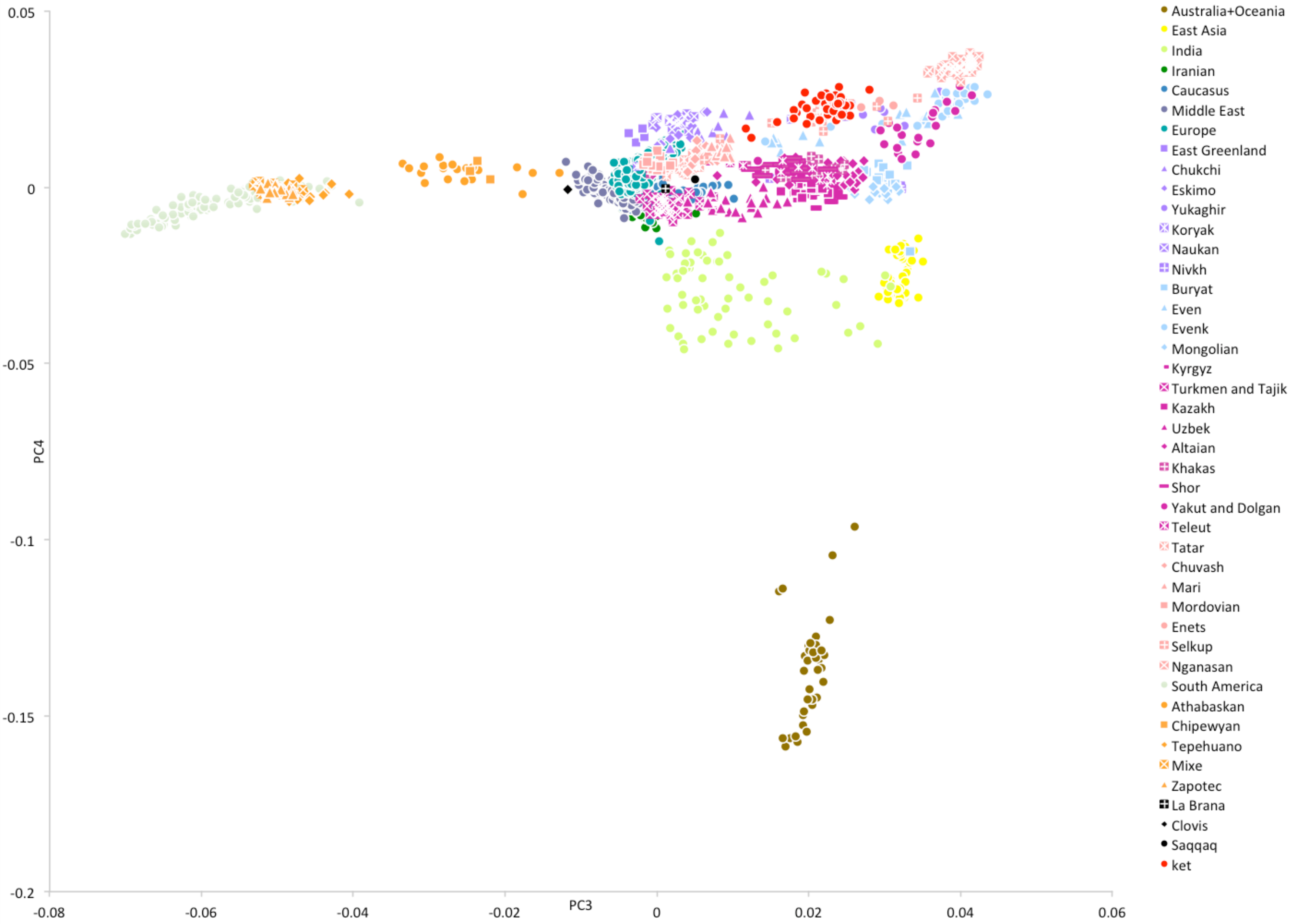

6.5. PCA, dataset 'GenoChip + Illumina arrays', PC3 vs PC4, zoom on the Ket population. Ancient genomes are shown in black.

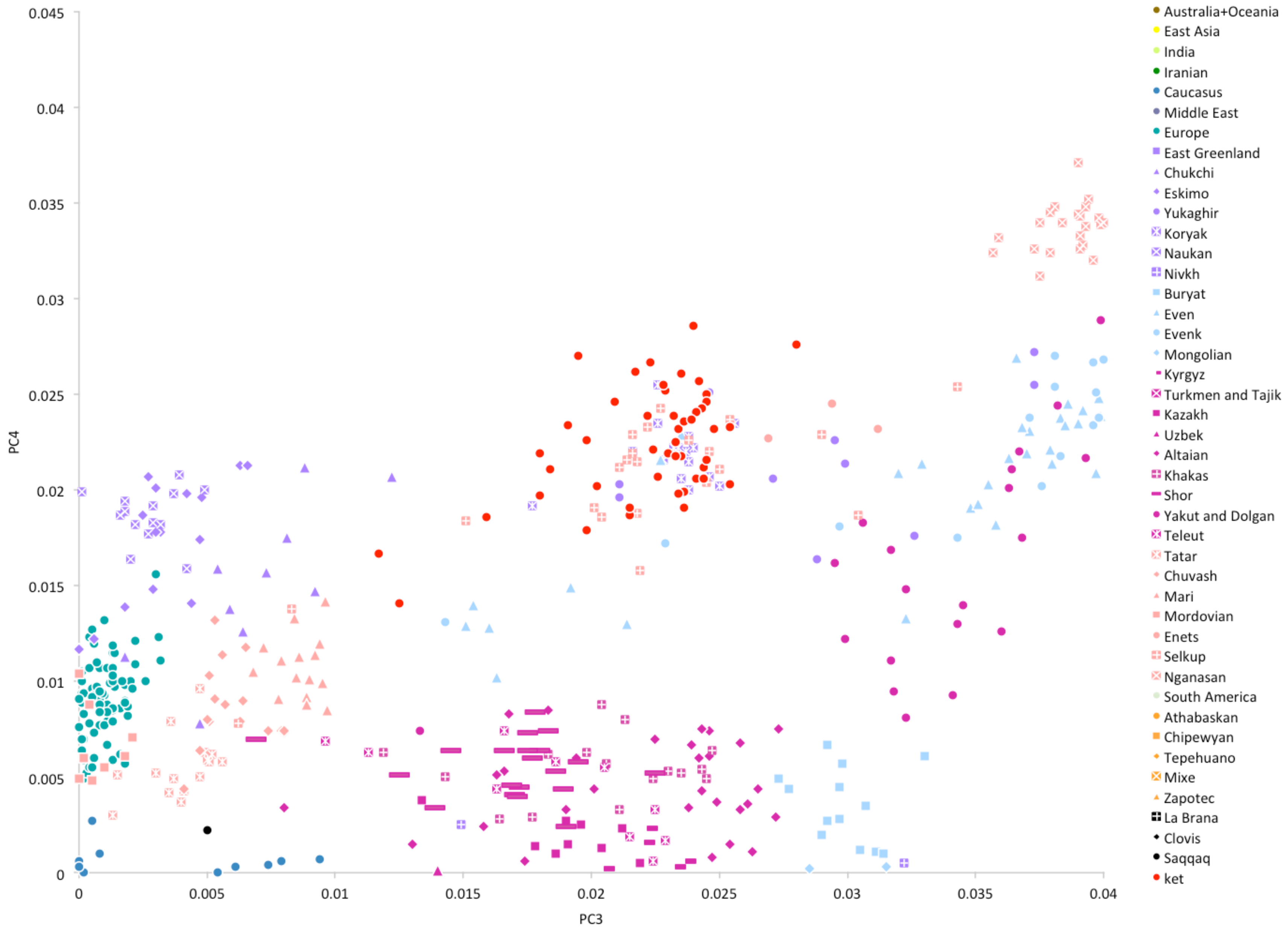

6.6. PCA, dataset 'GenoChip + Illumina arrays', PC3 vs PC4, only samples collected in the present study and published samples from respective populations are shown.

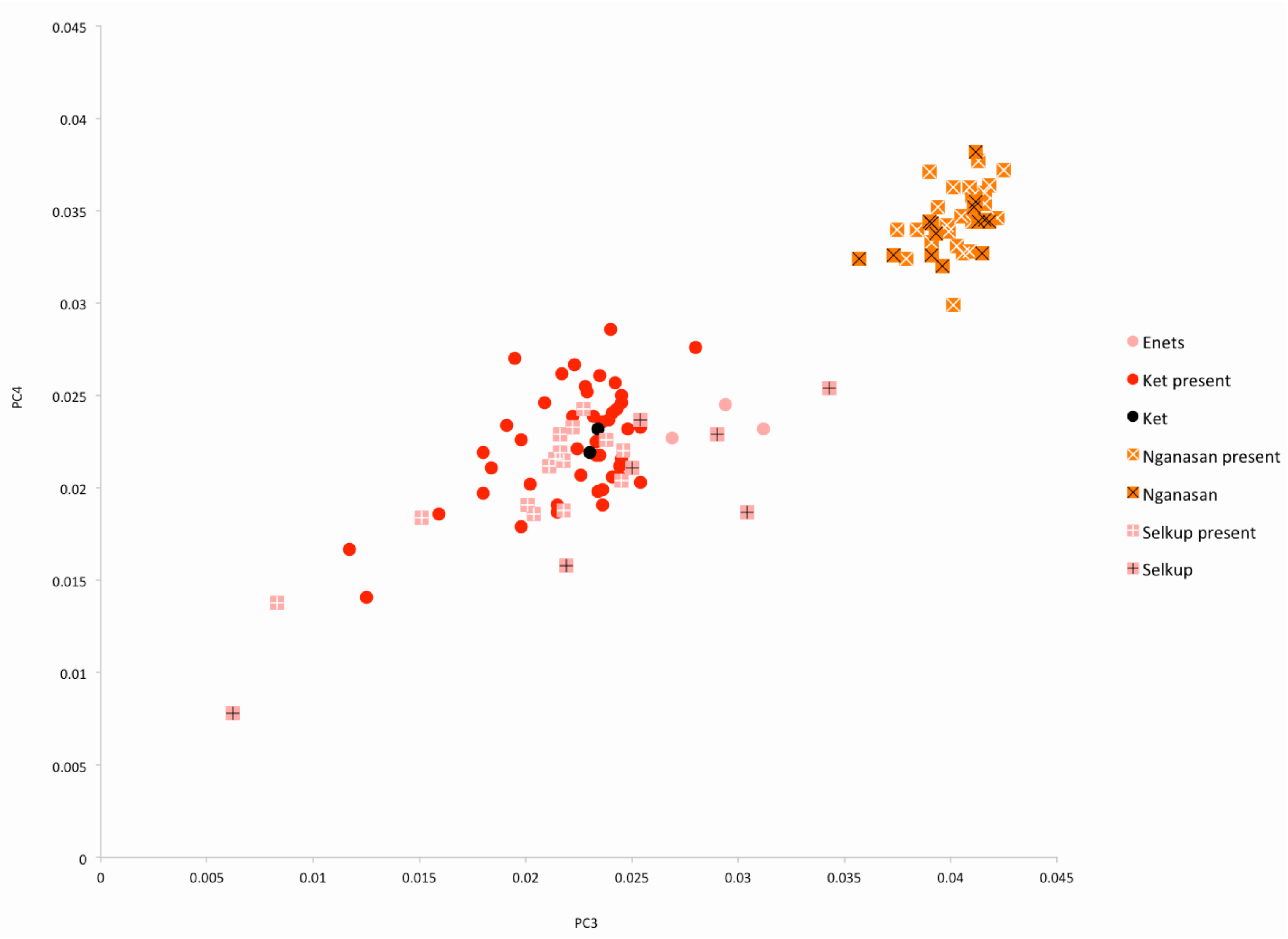

6.7. PCA, dataset 'Ket genomes + HumanOrigins array', PC1 vs PC2. African populations are not shown. Populations are color-coded by geographical region or language affiliation (in the case of Siberian and Central Asian populations), and most relevant populations are differentiated by marker shapes. Ancient genomes are shown in black.

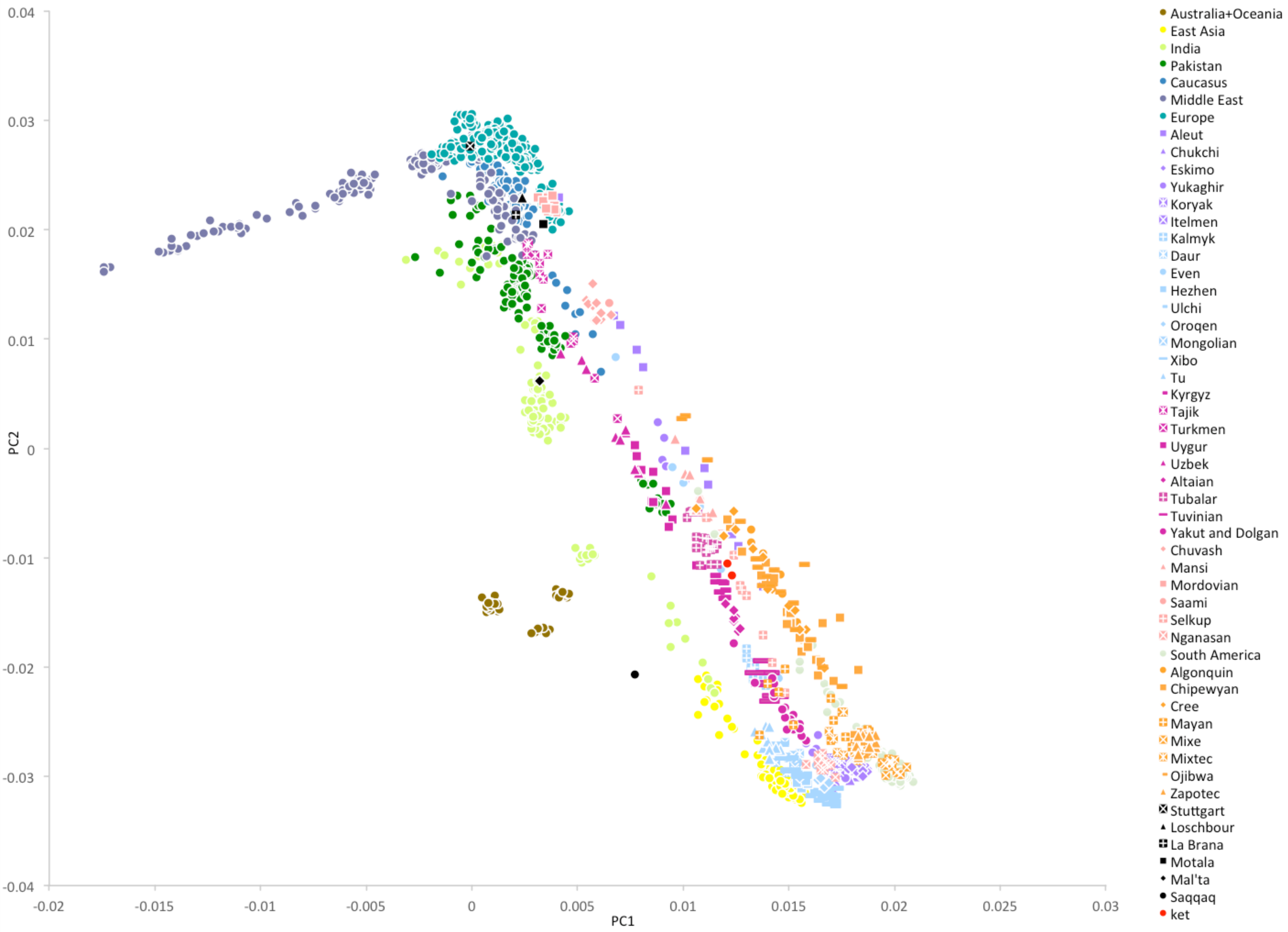

6.8. PCA, dataset 'Ket genomes + HumanOrigins array', PC1 vs PC2, zoom on the Ket population.

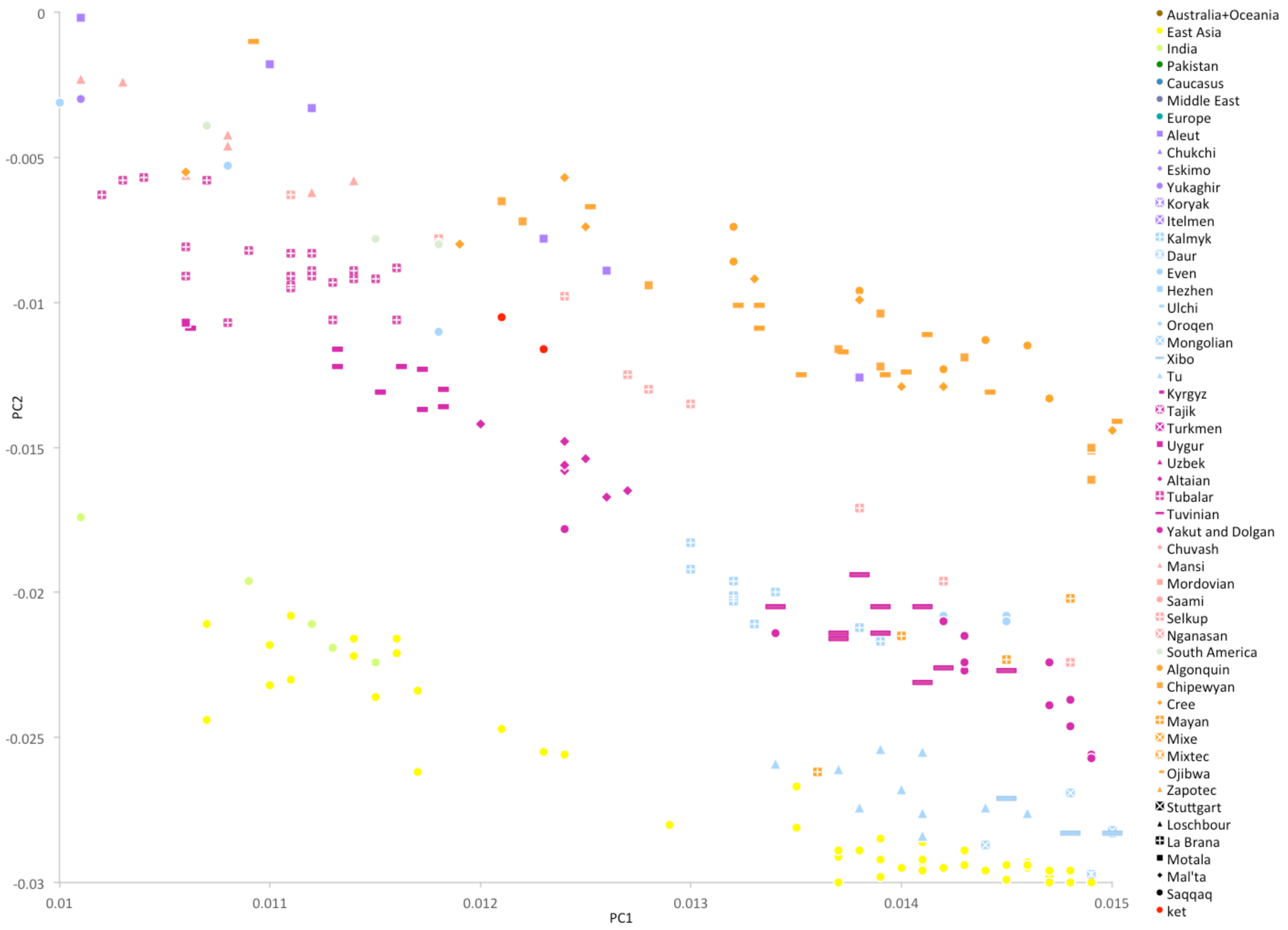

7. f_3 statistic

Due to the differences between experimental platforms, intersection between the GenoChip array data and all interesting datasets contains only a small number of SNPs. Therefore, we prepared four additional datasets based on two Ket genomes sequenced in this study, and including the Mal'ta and Saqqaq ancient genomes: two datasets of various population composition including the HumanOrigins SNP array data (69K and 196K SNPs), a genome-based dataset (398K SNPs), and its version with transition, i.e. CT and AG, polymorphisms removed (190K SNPs) (Suppl. Tables 1 and 2). Taking into account admixture coefficients for the two sequenced Ket individuals (Ket891 and Ket884, Fig. 1A), we selected Ket891 as an individual with lower values of the North European and Siberian admixture components (in the K=19 dimensional space). Ket891 was identified as non-admixed by the reAdmix and GPS analyses (Suppl. Table 3). Therefore, we also made a version of the 196K HumanOrigins-based dataset with the other Ket individual, Ket884, excluded (Suppl. Table 1). The results discussed below are predominantly based on these combined datasets. Finally, we prepared another version of the genome-based dataset with (225K SNPs) or without transitions (105K SNPs), including two additional Ket genomes from Raghavan et al. (2015), plus Siberian and selected Native American genomes from that study (Suppl. Tables 1 and 2). The latter dataset was used for analyzing f_3 vs. f_3 correlations and f_4-ratios only.

We calculated the outgroup f_3 statistic (Yoruba; Test, X) for the following 'Test' populations on all datasets containing a given population (except for the 69K dataset 'Ket genomes + HumanOrigins array +Verdu et al. 2014' used for investigating Na-Dene-speaking populations only): Kets, closely related Siberian populations sampled in this study and the respective published samples (Selkups, Nganasans, Enets), Na-Dene-speaking populations (Athabaskans, Chipewyans, Tlingit), populations tentatively included into the Na-Dene language family (Haida), and the Karasuk, Mal'ta, and Saqqaq ancient genomes. 'Admixture' f_3 statistic (Test; X, Y) was calculated for the same set of populations except for Mal'ta (Suppl. Table 5). The following results were consistent among all datasets: i/ Nganasans emerged as the best hit in outgroup f_3 statistic for Kets, Selkups, and Enets (Suppl. Figs 7.1, 7.2); ii/ a South European population and Nganasans made the best-scoring pair of admixture partners for Kets, Selkups and Enets according to 'admixture' f_3 statistic (Suppl. Table 5); iii/ no signature of admixture in reference Nganasans was revealed according to the same statistic, however Nganasans from this study were shown as admixed with a statistically significant Z-score (Suppl. Table 5). f_3(Yoruba; Test, X) results are not shown for Selkups, Nganasans, and Enets as they correlate well with f_3(Yoruba; Ket, X). Pearson correlation coefficients on the GenoChip-based dataset versus f_3(Yoruba; Ket, X) are as follows: 0.9959 for f_3(Yoruba; Enets, X), 0.9746 for f_3(Yoruba; Nganasan, X), 0.9699 for f_3(Yoruba; reference Nganasan, X), 0.9992 for f_3(Yoruba; Selkup, X), 0.9982 for f_3(Yoruba; reference Selkup, X). Pearson correlation coefficients on the HumanOrigins-based dataset versus f_3(Yoruba; Ket, X) are as follows: 0.9624 for f_3(Yoruba; reference Nganasan, X), and 0.9985 for f_3(Yoruba; reference Selkup, X) is the highest value among all populations. Thus, Yeniseian-speaking Kets and Uralic-speaking Selkups and Enets are grouped together not only in the ADMIXTURE analysis (through the Ket-Uralic admixture

component), but also share similar patterns in the analysis with the f_3 statistic. Correlation of outgroup f_3 statistics also showed that population samples obtained in this study are very much similar to the published ones. Pearson correlation coefficients on the GenoChip-based dataset are as follows: 0.9985 for f_3(Yoruba; Ket, X) vs. f_3(Yoruba; reference Ket, X), 0.9997 for f_3(Yoruba; Nganasan, X) vs. f_3(Yoruba; reference Nganasan, X), 0.9993 for f_3(Yoruba; Selkup, X) vs. f_3(Yoruba; reference Selkup, X).

Uralic-speaking Nganasans stood out in all ADMIXTURE analyses, demonstrating the global maximum of the Siberian admixture component, also having up to ~11% of the Ket-Uralic component according to some datasets ('GenoChip + Illumina arrays' and 'Ket genomes + Illumina arrays', see Suppl. Table 4). In outgroup f_3 set-ups best hits for Nganasans were East Siberian (Yukaghir, Even, Evenk, Dolgan, Ulchi, Oroqen) and Beringian populations (data not shown). As mentioned above, Nganasans of the published samples (Rasmussen et al. 2010; Reich et al. 2012; Lazaridis et al. 2014) had no negative Z-scores in the 'admixture' f_3 set-up (Suppl. Table 5). However, Nganasans from this study had statistically significant Z-scores down to -4.4 for pairs composed of reference Nganasans and a South European population (dataset 'GenoChip + Illumina arrays'). In the ADMIXTURE analysis with same dataset at K=20 (demonstrating the lowest cross-validation error), reference Nganasans (22 individuals) had 0% of North or South European admixture components, but Nganasans from this study (24 individuals) had marginal levels of South European (up to 1.4%) and North European components (up to 4.1%, data not shown). Hence results of both analyses demonstrate a low level of European admixture in the Nganasan population from this study. Similarly, Kets from this study, but not reference Kets, were revealed as admixed with highly significant Z-scores on the GenoChip-based dataset (Suppl. Table 5). A much broader sampling of Kets in this study (46 individuals) has apparently captured more variation as compared to a small reference sample in that dataset (2 individuals), which could possibly lead to different 'admixture' f_3 results. Both Ket populations, 2 individuals from this study and 4 reference individuals, were shown as admixed with highly significant Z-scores on the dataset 'Ket genomes + Illumina arrays' (Suppl. Table 5).

Outgroup f_3 of the form (Yoruba; Mal'ta, X) on the dataset obtained by merging both Ket genomes and the HumanOrigins array data (Lazaridis et al. 2014), and on the dataset version with Ket884 excluded, showed the highest degree of genetic drift shared with Mal'ta in a Saami individual and in Kets, among all modern Eurasian populations west of Chukotka and Kamchatka (Suppl. Figs. 7.13, 7.14). However, in both cases differences in f_3 statistics were negligible between Kets and some other North Eurasian populations: Estonians, Mansi, Lithuanians, Russians, as shown by $|Z_{diff}$ scores$| < 1$ (Suppl. Figs. 7.13, 7.14) . A similar result was reproduced with f_3(Yoruba; Mal'ta, X) on a genome-based-based dataset of 398,163 SNPs (64 individuals from 36 populations) and on its version without transitions (189,964 SNPs): the f_3(Yoruba; Mal'ta, Ket) values appeared within the range of f_3 statistics (Yoruba; Mal'ta, Native Americans) (Suppl. Figs. 7.15, 7.16). However, differences between f_3(Yoruba; Mal'ta, Ket) and some higher and lower statistic values were non-significant: $|Z_{diff}| < 3$ for Mayans, Motala, Afanasievo, Clovis, Karitiana, Mixe, Athabaskans, Greenlanders, Andronovo, Aleutian, Karasuk, Iron Age Russia, Saqqaq, Mari, French, Indians, Iron Age Altai, and Loschbour (Suppl. Fig. 15).

f_3(Yoruba; Ket, X) on the genome-based dataset with (Suppl. Fig. 7.3) or without (Suppl. Fig. 7.4) transitions recovered Saqqaq and Late Dorset as top hits for Kets, followed by Native American groups. The following populations had f_3 statistics not different significantly from f_3(Yoruba; Ket, Saqqaq): $|Z_{diff}|$ scores < 3 were obtained for Late Dorset, Mayans, Mixe, and Athabaskans (Suppl. Fig. 7.3). f_3(Yoruba; Saqqaq, X) on the same datasets recovered the following top hits: Late Dorset (by far the best Z-score, with the lowest Z_{diff} scores of 9.7 and 8.1), Greenlander Inuits, Nivkhs, Kets, Mayans, Athabaskans (Suppl. Figs. 7.18, 7.19). The following populations had f_3 statistics not different significantly from f_3(Yoruba; Saqqaq, Ket): $|Z_{diff}|$ scores < 3 were obtained for Nivkhs, Mayans, Mixe, Athabaskans, Han, Karitiana, Clovis, Kinh, and Dai (Suppl. Fig. 7.18). Unfortunately, other modern Siberian populations were lacking in this dataset with the exception of Nivkhs, and Beringian populations were represented by Greenland Inuits (Greenlanders) and an Aleutian individual only. 'Admixture' f_3(Ket; X, Y) on the original genome-based dataset and on its version without transitions recovered Motala12 and Saqqaq as a population pair with the lowest f_3 statistic and Z-scores of -3.3 (Suppl. Table 5). Although this Z-score is above the generally accepted significance threshold of $|Z| > 3$, Bonferroni-corrected threshold on this dataset equals 3.9. Motala12 has a certain degree of ANE ancestry (estimated at ~22% by Lazaridis et al. 2014), therefore modelling Kets as a mixture of Motala12 and Saqqaq, having considerable Siberian ancestry (Suppl. Table 6), appears reasonable. Statistic f_3(Saqqaq; X, Y) showed no statistically significant negative values, as ancient mixture partners that gave rise to the Saqqaq population were lacking in the dataset (Suppl. Table 5).

A very weak signal of the Ket - Na-Dene relationship was detected with the outgroup f_3 statistic f_3(Yoruba; Haida, X) on the HumanOrigins-based dataset (Suppl. Fig. 7.9): Kets emerged as the best hit to Haida in Eurasia, west of Chukotko-Kamchatkan (Beringian) populations, whereas Nganasans is the best Siberian hit to Chipewyans and Tlingit according to outgroup f_3 statistic (Suppl. Figs. 7.8, 7.10). However, f_4(Haida, Chimp; Ket, X) produced Z-scores for a number of Eurasian populations, e.g. Nganasans and Saami, close to zero (0.14 and 0.11, respectively, Suppl. Fig. 8.13), in line with only a marginal difference in statistics f_3(Yoruba; Haida, Ket) and f_3(Yoruba; Haida, Nganasan) estimated with Z_{diff} scores (Suppl. Fig. 7.9).

Correlation of outgroup f_3 statistics between a pair of populations was used as another approximate measure of population relatedness (see, e.g., Allentoft et al. 2015). Pearson coorelation coefficients were calculated for all possible pairs of statistics f_3(Yoruba; Test, X) on the genome-based dataset without transitions (190K SNPs) and on dataset 'Ket genomes + Raghavan et al. 2015' (225K SNPs), which combines two Ket genomes from this study with two Ket genomes published by Raghavan et al. (2015). For each Eurasian and American group, best hits with correlation coefficients > 0.8 are shown in Suppl. file S2. If less than five populations produced r > 0.8, the cut-off was relaxed to 0.6. In this analysis, 'core Siberian' populations, i.e. Siberian populations to the exclusion of Chukotkan and Kamchatkan groups, demonstrate highly correlated f_3 statistics: r > 0.98 for any pair within Altaians, Buryats, Nivkhs, and Yakuts. Best hits for Kets were (r > 0.95): Altaians, Koryaks, and Iron Age Russia. f_3 statistics for Iron Age Russia correlated almost equally well to all modern Siberian populations in the

dataset (r > 0.95): Altaians, Buryats, Kets, Nivkhs, Yakuts. f_3 statistics for Iron Age Altai and Karasuk correlated best between each other and with those of Kets (r > 0.8). These results suggest genetic continuity could have existed in South Siberia at least from the Iron Age (Allentoft et al. 2015). Best hits for Paleo-Eskimos (Late Dorset and Saqqaq) included Koryaks, Kets, and other Beringian and 'core Siberian' populations (r > 0.85, Suppl. file S2). Kets were a third-best for Late Dorset (after Saqqaq and Koryak) or a fourth-best hit for Saqqaq (after Late Dorset, Koryak, and Eskimo). It should be noticed that correlation cefficients for other hits were very much similar. Although lacking a formal test of statistical significance, the results discussed here are in good agreement with the results obtained with other methods: outgroup f_3 (see outgroup f_3 statistics for dataset 'Ket genomes + Raghavan et al. 2015' in Suppl. file S3) and f_4 statistics (Suppl. Information, Section 8), and TreeMix (Suppl. Information, Section 9).

References

Lazaridis, I. *et al.* Ancient human genomes suggest three ancestral populations for present-day Europeans. *Nature* **513,** 409–413 (2014).

Rasmussen, M. *et al.* Ancient human genome sequence of an extinct Palaeo-Eskimo. *Nature* **463,** 757–762 (2010).

Reich, D. *et al.* Reconstructing Native American population history. *Nature* **488,** 370–374 (2012).

Suppl. Table 5. Admixture f_3 statistics: up to five most negative statistics are shown for each reference population. Z-scores statistically significant on a given dataset according to Bonferroni correction for multiple testing are highlighted in bold.

Dataset	Reference	Test 1	Test 2	f_3	f_3 SE	Z	Z cutoff with Bonferroni correction
GenoChip + Illumina arrays	Ket (this study)	Greek	Nganasan	-0.0088	0.00101	**-8.75**	-4.37
		Danish	Nganasan	-0.0086	0.00096	**-8.87**	-4.37
		Lithuanian	Nganasan	-0.0082	0.00102	**-8.02**	-4.37
		Belarusian	Nganasan	-0.0081	0.00100	**-8.05**	-4.37
		Sardinian	Nganasan	-0.0079	0.00103	**-7.70**	-4.37
GenoChip + Illumina arrays	Ket	Italian	Nganasan	-0.0008	0.00265	-0.29	-4.37
		Danish	Nganasan	-0.0003	0.00269	-0.12	-4.37
		Sardinian	Nganasan	-0.0002	0.00269	-0.09	-4.37
		German	Nganasan	-0.0002	0.00270	-0.08	-4.37
		Greek	Nganasan	0.0000	0.00271	-0.01	-4.37
Ket genomes + Illumina arrays	Ket (this study)	Lithuanian	Nganasan	-0.0189	0.00045	**-42.11**	-4.43
		Basque	Nganasan	-0.0188	0.00045	**-42.16**	-4.43
		Spanish	Nganasan	-0.0187	0.00044	**-42.21**	-4.43
		Belarusian	Nganasan	-0.0187	0.00044	**-42.37**	-4.43
		French	Nganasan	-0.0187	0.00044	**-42.66**	-4.43
Ket genomes + Illumina arrays	Ket	Lithuanian	Nganasan	-0.0027	0.00028	**-9.60**	-4.43
		Belarusian	Nganasan	-0.0026	0.00027	**-9.41**	-4.43
		French	Nganasan	-0.0026	0.00027	**-9.46**	-4.43
		Swedish	Nganasan	-0.0026	0.00027	**-9.37**	-4.43
		Basque	Nganasan	-0.0025	0.00028	**-9.11**	-4.43
Ket genomes + HumanOrigins array	Ket (this study)	Motala12	Nganasan	-0.0060	0.00054	**-10.98**	-4.55
		Orcadian	Nganasan	-0.0049	0.00039	**-12.63**	-4.55
		English	Nganasan	-0.0049	0.00039	**-12.43**	-4.55
		Lithuanian	Nganasan	-0.0048	0.00040	**-12.20**	-4.55
		Czech	Nganasan	-0.0048	0.00039	**-12.33**	-4.55
genome-based dataset	Ket (this study)	Motala12	Saqqaq	-0.0050	0.00151	-3.34	-3.94
		French	Late Dorset	-0.0045	0.00137	-3.27	-3.94
		Motala12	Late Dorset	-0.0043	0.00178	-2.41	-3.94
		Sardinian	Saqqaq	-0.0041	0.00118	-3.52	-3.94
		Afanasievo	Late Dorset	-0.0040	0.00165	-2.45	-3.94
genome-based dataset without transitions	Ket (this study)	Motala12	Saqqaq	-0.0077	0.00231	-3.32	-3.94
		Avar	Saqqaq	-0.0060	0.00203	-2.97	-3.94
		Motala12	Late Dorset	-0.0059	0.00283	-2.08	-3.94
		French	Saqqaq	-0.0057	0.00183	-3.11	-3.94
		Sardinian	Saqqaq	-0.0054	0.00185	-2.92	-3.94
GenoChip + Illumina arrays	Selkup (this study)	Greek	Nganasan	-0.0071	0.00116	**-6.14**	-4.37
		Danish	Nganasan	-0.0069	0.00110	**-6.24**	-4.37
		Belarusian	Nganasan	-0.0066	0.00113	**-5.84**	-4.37

		Lithuanian	Nganasan	-0.0062	0.00119	**-5.24**	-4.37
		German	Nganasan	-0.0061	0.00113	**-5.40**	-4.37
GenoChip + Illumina arrays	Selkup	Greek	Nganasan	-0.0141	0.00118	**-11.94**	-4.37
		Italian	Nganasan	-0.0134	0.00118	**-11.31**	-4.37
		Sardinian	Nganasan	-0.0132	0.00122	**-10.79**	-4.37
		Belarusian	Nganasan	-0.0130	0.00119	**-10.87**	-4.37
		Danish	Nganasan	-0.0129	0.00116	**-11.12**	-4.37
Ket genomes + HumanOrigins array	Selkup	Motala12	Nganasan	-0.0059	0.00030	**-19.29**	-4.55
		English	Nganasan	-0.0053	0.00015	**-34.22**	-4.55
		Czech	Nganasan	-0.0052	0.00015	**-34.16**	-4.55
		Icelandic	Nganasan	-0.0052	0.00015	**-34.14**	-4.55
		Orcadian	Nganasan	-0.0052	0.00015	**-34.46**	-4.55
GenoChip + Illumina arrays	Nganasan (this study)	Greek	Nganasan	-0.0029	0.00067	**-4.36**	-4.37
		Italian	Nganasan	-0.0024	0.00067	-3.58	-4.37
		Romanian	Nganasan	-0.0023	0.00062	-3.66	-4.37
		Iberian	Nganasan	-0.0023	0.00065	-3.46	-4.37
		Armenian	Nganasan	-0.0023	0.00062	-3.63	-4.37
GenoChip + Illumina arrays	Nganasan	*no negative statistics*					
Ket genomes + HumanOrigins array	Nganasan	*no negative statistics*					
GenoChip + Illumina arrays	Enets (this study)	Italian	Nganasan	-0.0096	0.00175	**-5.50**	-4.37
		Greek	Nganasan	-0.0096	0.00183	**-5.25**	-4.37
		Sardinian	Nganasan	-0.0090	0.00180	**-4.96**	-4.37
		Bulgarian	Nganasan	-0.0088	0.00175	**-5.05**	-4.37
		Belarusian	Nganasan	-0.0087	0.00188	**-4.63**	-4.37
GenoChip + Illumina arrays	Athabaskan	Sardinian	Chipewyan	-0.0082	0.00151	**-5.46**	-4.37
		Chechen	Chipewyan	-0.0077	0.00135	**-5.73**	-4.37
		Russian	Chipewyan	-0.0077	0.00134	**-5.73**	-4.37
		Tunisia	Chipewyan	-0.0077	0.00135	**-5.68**	-4.37
		German	Chipewyan	-0.0075	0.00139	**-5.37**	-4.37
genome-based dataset	Athabaskan	*no negative statistics*					
genome-based dataset without transitions	Athabaskan	*no negative statistics*					
GenoChip + Illumina arrays	Chipewyan	*no negative statistics*					
Ket genomes + HumanOrigins array	Chipewyan	*no negative statistics*					
Ket genomes + HumanOrigins array + Verdu et al. 2014	Chipewyan	*no negative statistics*					
Ket genomes + HumanOrigins array + Verdu et al. 2014	Haida	Stuttgart	Piapoco	-0.0110	0.00058	**-19.07**	-4.57
		Loschbour	Piapoco	-0.0107	0.00061	**-17.42**	-4.57
		Sardinian	Piapoco	-0.0104	0.00031	**-33.49**	-4.57

		Sardinian	Cabecar	-0.0103	0.00034	**-30.23**	-4.57
		Stuttgart	Mixe	-0.0102	0.00051	**-20.19**	-4.57
Ket genomes + HumanOrigins array + Verdu et al. 2014	Tlingit	Stuttgart	Piapoco	-0.0093	0.00055	**-16.80**	-4.57
		Loschbour	Piapoco	-0.0090	0.00058	**-15.55**	-4.57
		Piapoco	Sardinian	-0.0086	0.00031	**-27.88**	-4.57
		Sardinian	Cabecar	-0.0085	0.00034	**-25.28**	-4.57
		Stuttgart	Mixe	-0.0085	0.00047	**-17.97**	-4.57
genome-based dataset	Karasuk	Afanasievo	Dai	-0.0039	0.00083	**-4.73**	-3.94
		Afanasievo	Late Dorset	-0.0036	0.00132	-2.71	-3.94
		French	Late Dorset	-0.0034	0.00101	-3.40	-3.94
		Mal'ta	Han	-0.0034	0.00098	-3.47	-3.94
		Loschbour	Late Dorset	-0.0032	0.00136	-2.31	-3.94
genome-based dataset without transitions	Karasuk	Afanasievo	Nivkh	-0.0052	0.00130	**-4.00**	-3.94
		French	Late Dorset	-0.0051	0.00156	-3.26	-3.94
		Loschbour	Late Dorset	-0.0049	0.00215	-2.28	-3.94
		Motala12	Saqqaq	-0.0049	0.00179	-2.72	-3.94
		Andronovo	Late Dorset	-0.0048	0.00161	-2.99	-3.94
Ket genomes + HumanOrigins array	Saqqaq	*no negative statistics*					
genome-based dataset	Saqqaq	Mixe	Late Dorset	-0.0003	0.00198	-0.17	-3.94
		Clovis	Late Dorset	-0.0002	0.00194	-0.09	-3.94
		Afanasievo	Late Dorset	-0.0001	0.00197	-0.03	-3.94
genome-based dataset without transitions	Saqqaq	Iron Age Russia	Late Dorset	-0.0042	0.00308	-1.37	-3.94
		Afanasievo	Late Dorset	-0.0031	0.00293	-1.06	-3.94
		Clovis	Late Dorset	-0.0026	0.00289	-0.89	-3.94
		Ket	Late Dorset	-0.0016	0.00256	-0.64	-3.94
		Dai	Late Dorset	-0.0016	0.00259	-0.62	-3.94

7.1. Statistics f_3(Yoruba; Ket, X) computed on the dataset 'GenoChip + Illumina arrays' for the Ket population from this study (46 individuals). **A.** Color-coded f_3(Yoruba; Ket, X) values plotted on the world map using QGIS v.2.8. Top five values are shown in the bottom left corner.

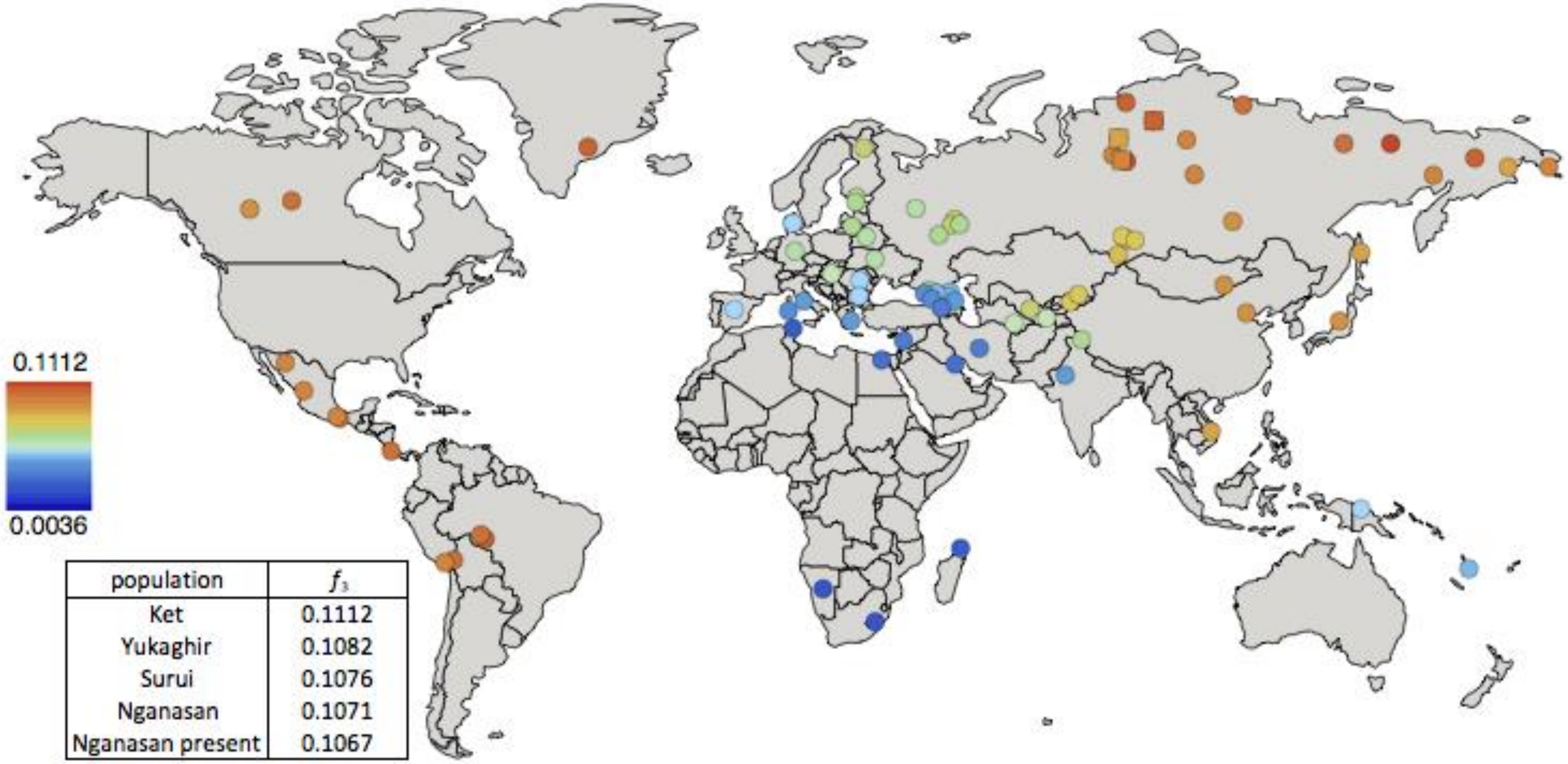

B. All f_3 values (green circles) sorted in descending order with their standard errors (single SE interval) shown by vertical lines. Absolute Z_{diff} scores >3.2 (plotted above the bars) show that a given f_3 statistic is significantly different from the lowest one, i.e. from that of the best hit. Bonferroni correction for 87 independent tests and a threshold *p*-value of 0.000575 were used. The Yukaghir population was considered the best hit in this figure, as the only lower f_3 statistic belonged to the reference Kets.

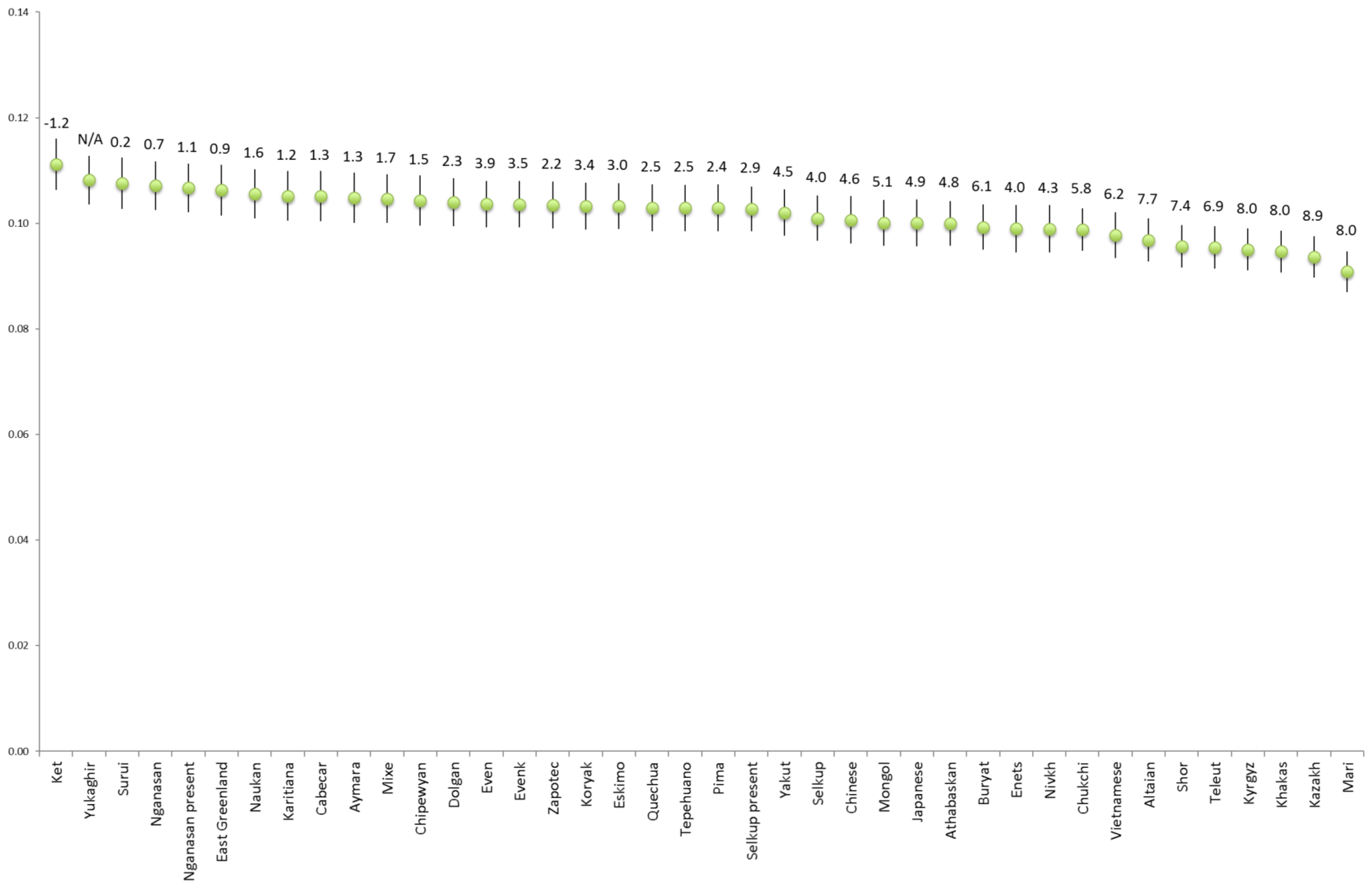

7.2. Statistics f_3(Yoruba; Ket, X) computed on the dataset 'Ket genomes + HumanOrigins array' for the Ket population from this study (2 individuals).

A. Color-coded f_3(Yoruba; Ket, X) values plotted on the world map using QGIS v.2.8. Top five values are shown in the bottom left corner.

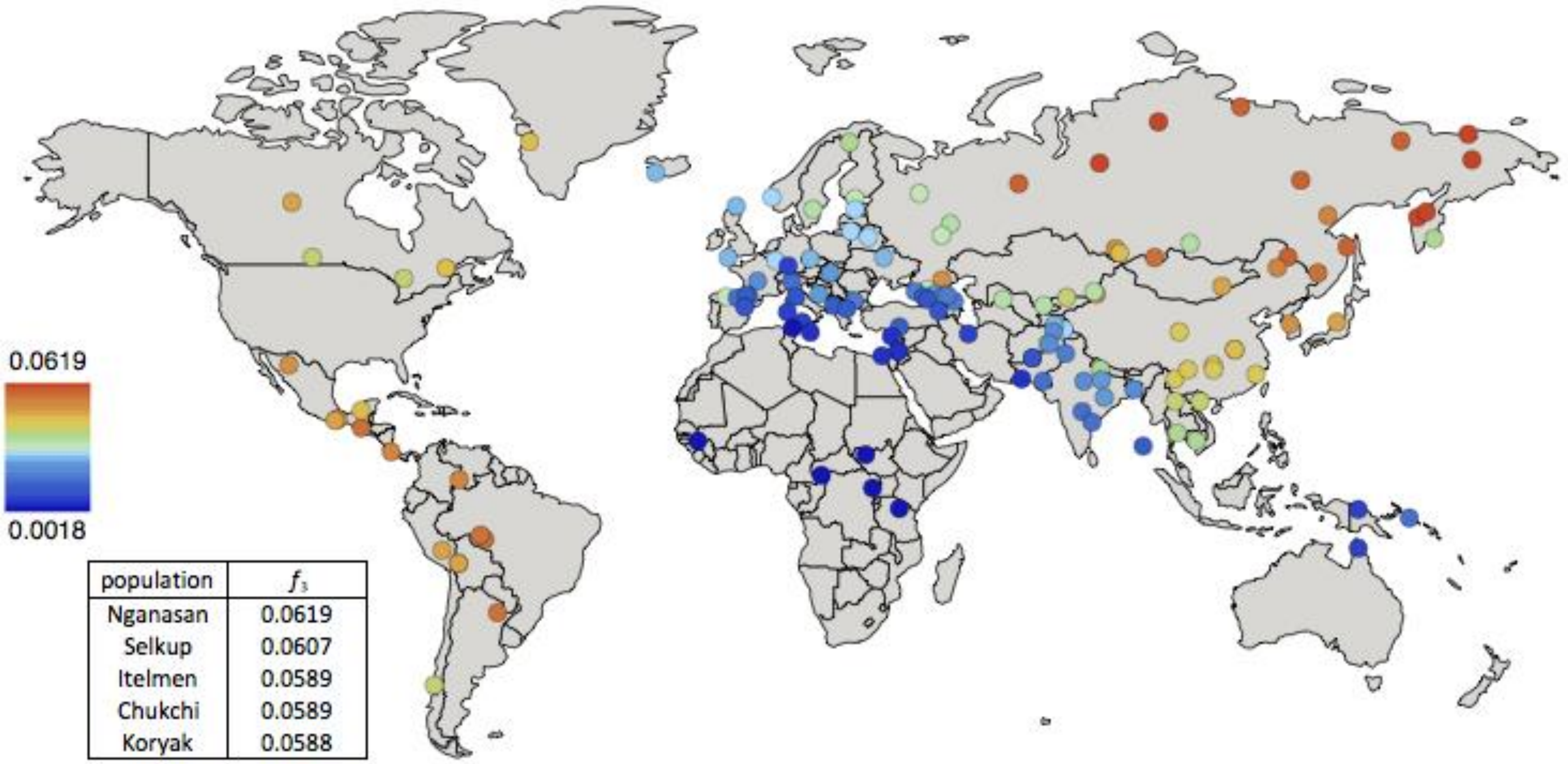

population	f_3
Nganasan	0.0619
Selkup	0.0607
Itelmen	0.0589
Chukchi	0.0589
Koryak	0.0588

B. Top f_3 values (green circles) sorted in descending order with their standard errors (single SE interval) shown by vertical lines. Absolute Z_{diff} scores >3.4 (plotted above the bars) show that a given f_3 statistic is significantly different from the lowest one, i.e. from that of the best hit. Bonferroni correction for 136 independent tests and a threshold p-value of 0.000368 were used.

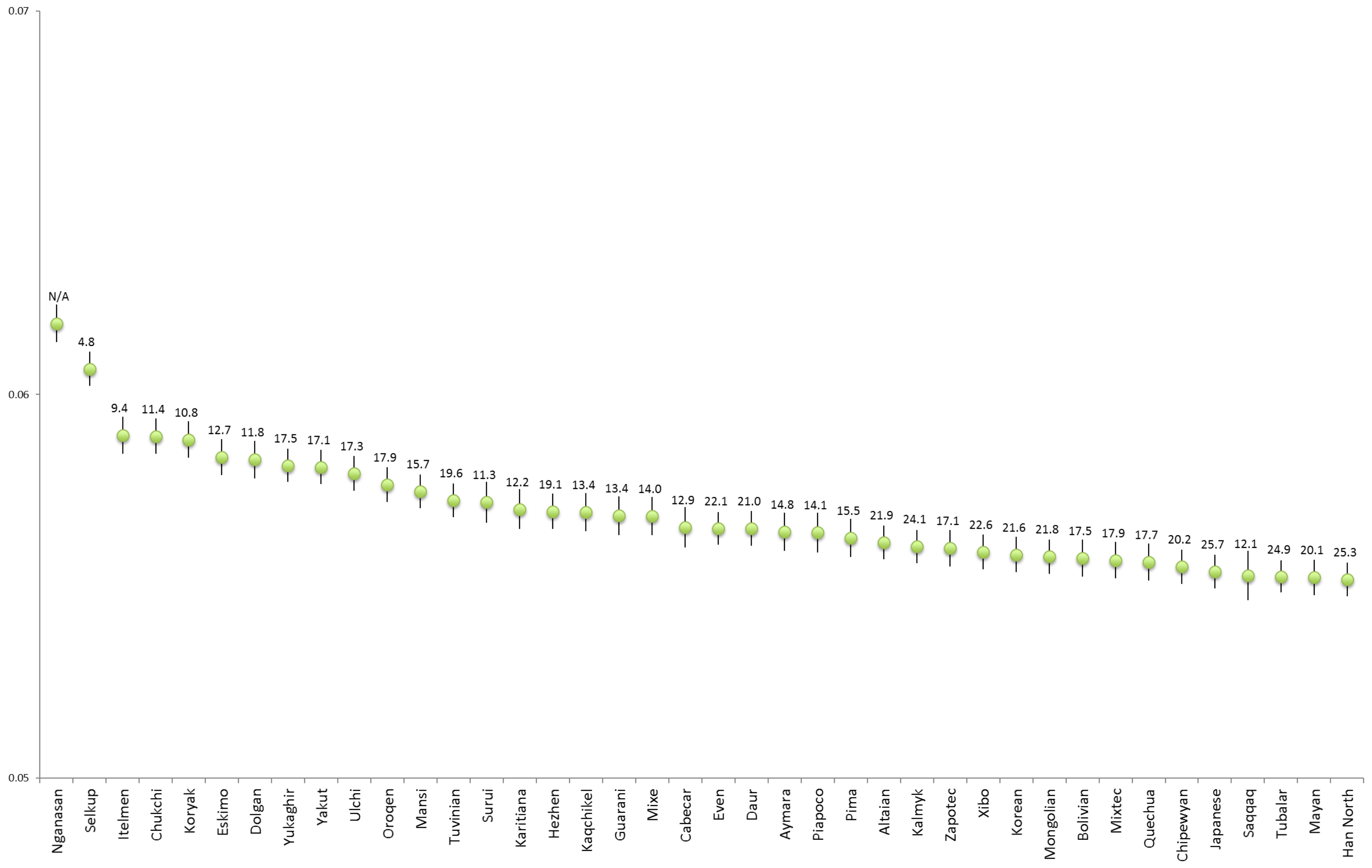

7.3. Statistics f_3(Yoruba; Ket, X) computed on the genome-based dataset (2 Ket individuals). All f_3 values (green circles) are in descending order with their standard errors (single SE interval) shown by vertical lines. Absolute Z_{diff} scores >3 (plotted above the bars) show that a given f_3 statistic is significantly different from the lowest one, i.e. from that of the best hit. Bonferroni correction for 33 independent tests and a threshold *p*-value of 0.0015 were used.

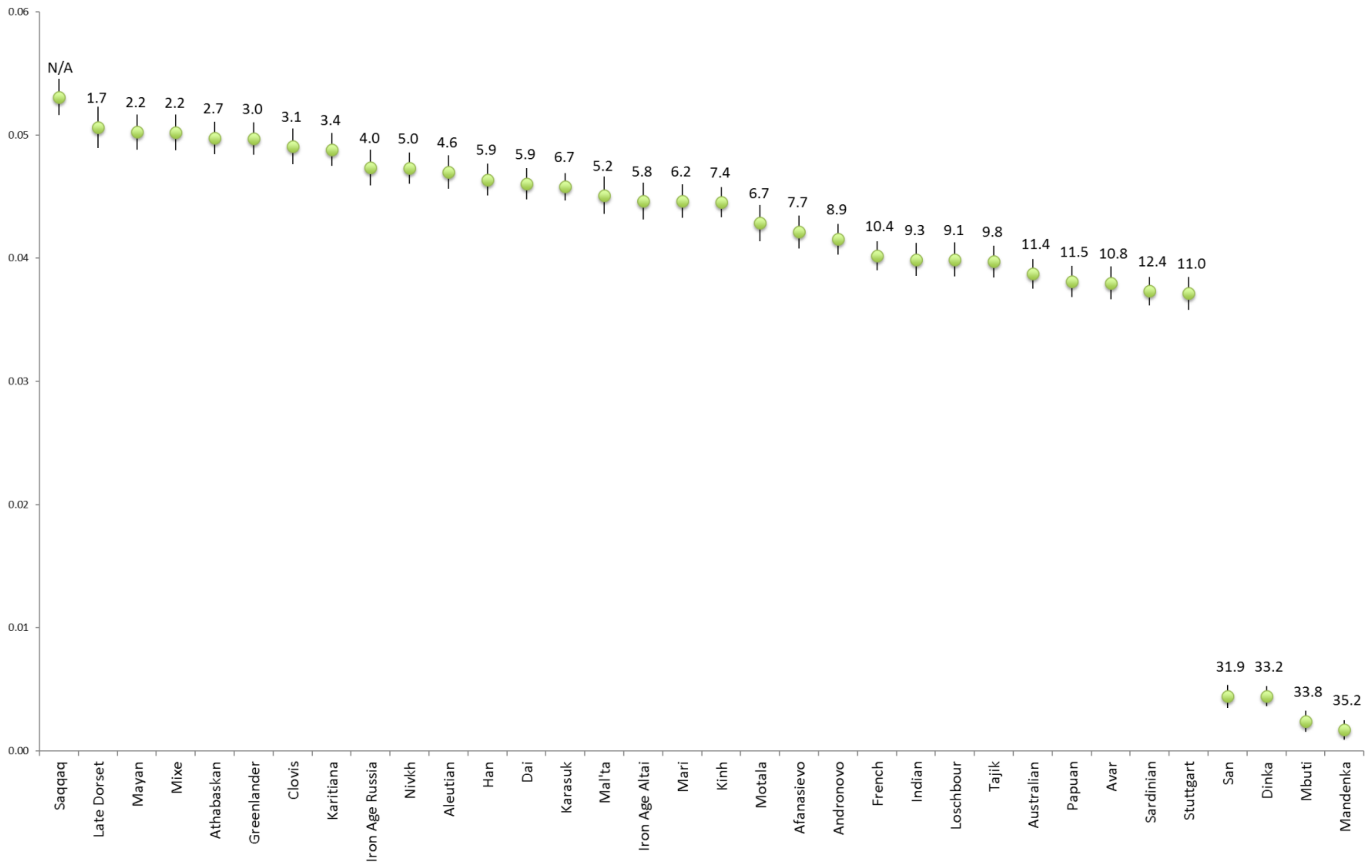

7.4. Statistics f_3(Yoruba; Ket, X) computed on the genome-based dataset without transitions (2 Ket individuals). All f_3 values (green circles) are sorted in descending order with their standard errors (single SE interval) shown by vertical lines. Absolute Z_{diff} scores >3 (plotted above the bars) show that a given f_3 statistic is significantly different from the lowest one, i.e. from that of the best hit. Bonferroni correction for 33 independent tests and a threshold *p*-value of 0.0015 were used.

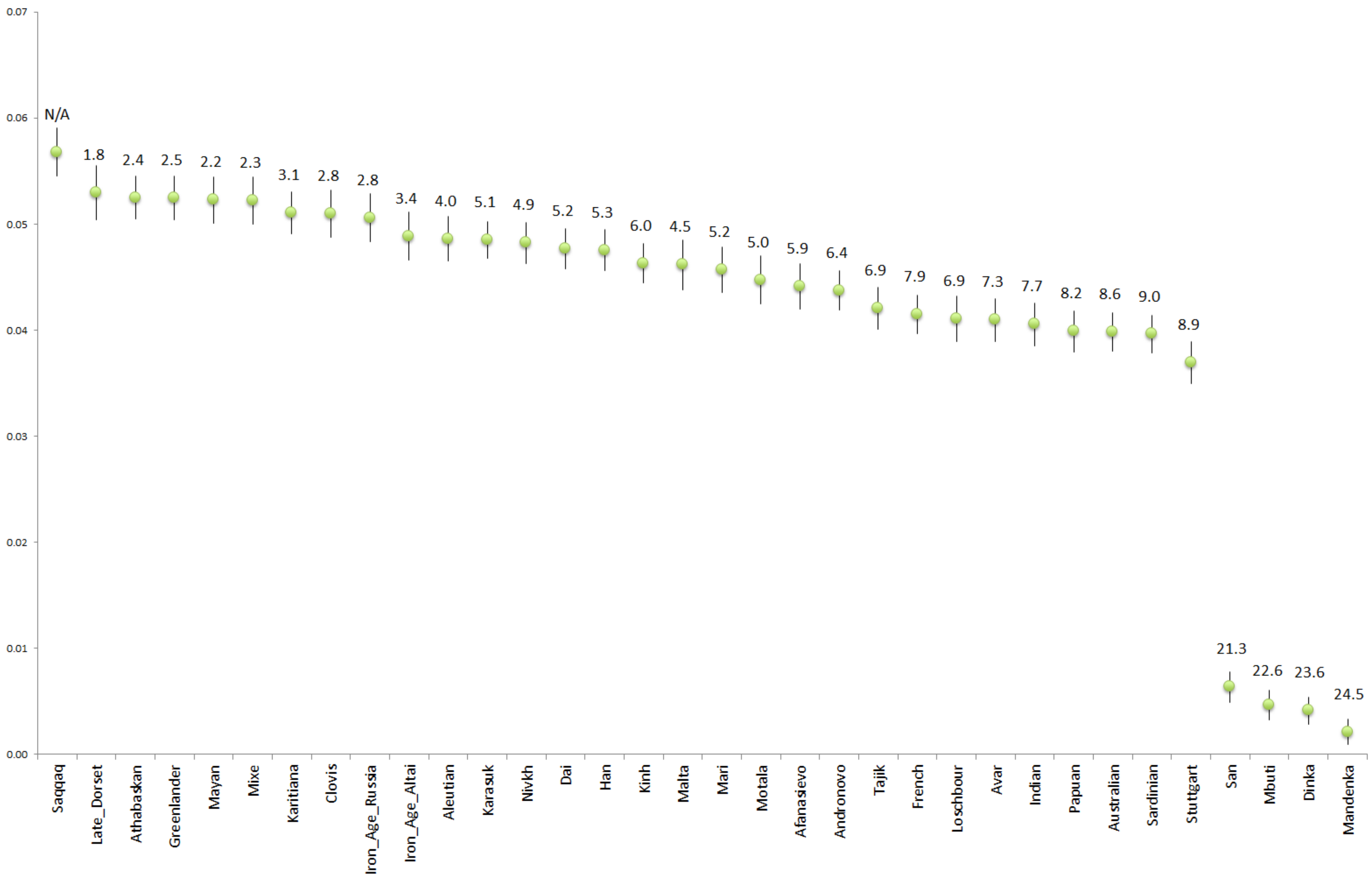

7.5. Statistics f_3(Yoruba; Karasuk, X) computed on the genome-based dataset (6 Karasuk individuals). All f_3 values (green circles) are sorted in descending order with their standard errors (single SE interval) shown by vertical lines. Absolute Z_{diff} scores >3 (plotted above the bars) show that a given f_3 statistic is significantly different from the lowest one, i.e. from that of the best hit. Bonferroni correction for 33 independent tests and a threshold *p*-value of 0.0015 were used.

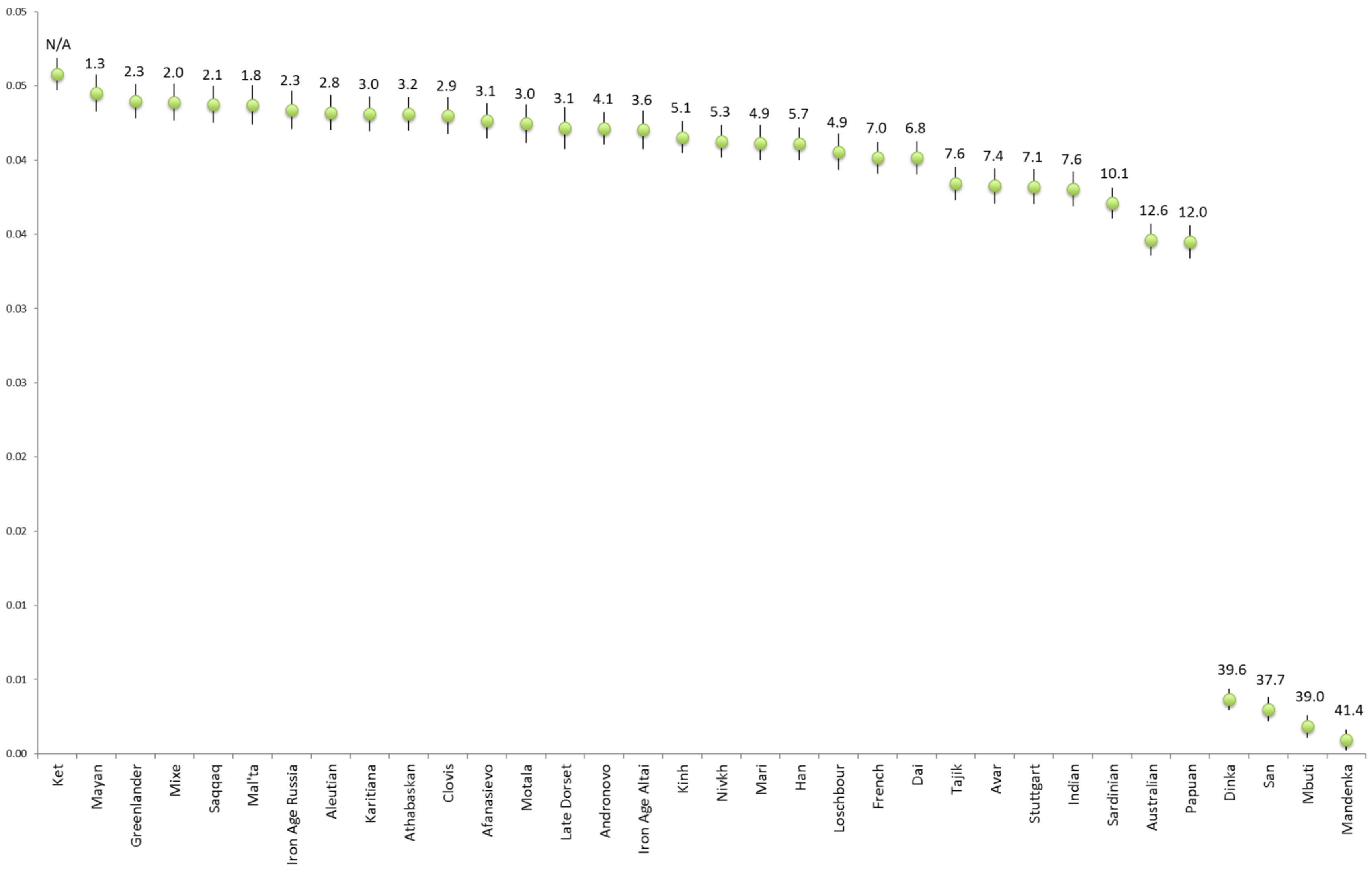

7.6. Statistics f_3(Yoruba; Karasuk, X) computed on the genome-based dataset without transitions (6 Karasuk individuals). All f_3 values (green circles) are sorted in descending order with their standard errors (single SE interval) shown by vertical lines. Absolute Z_{diff} scores >3 (plotted above the bars in red) show that a given f_3 statistic is significantly different from the lowest one, i.e. from that of the best hit. Bonferroni correction for 33 independent tests and a threshold p-value of 0.0015 were used. Z_{diff} scores in blue were calculated vs. the Ket population.

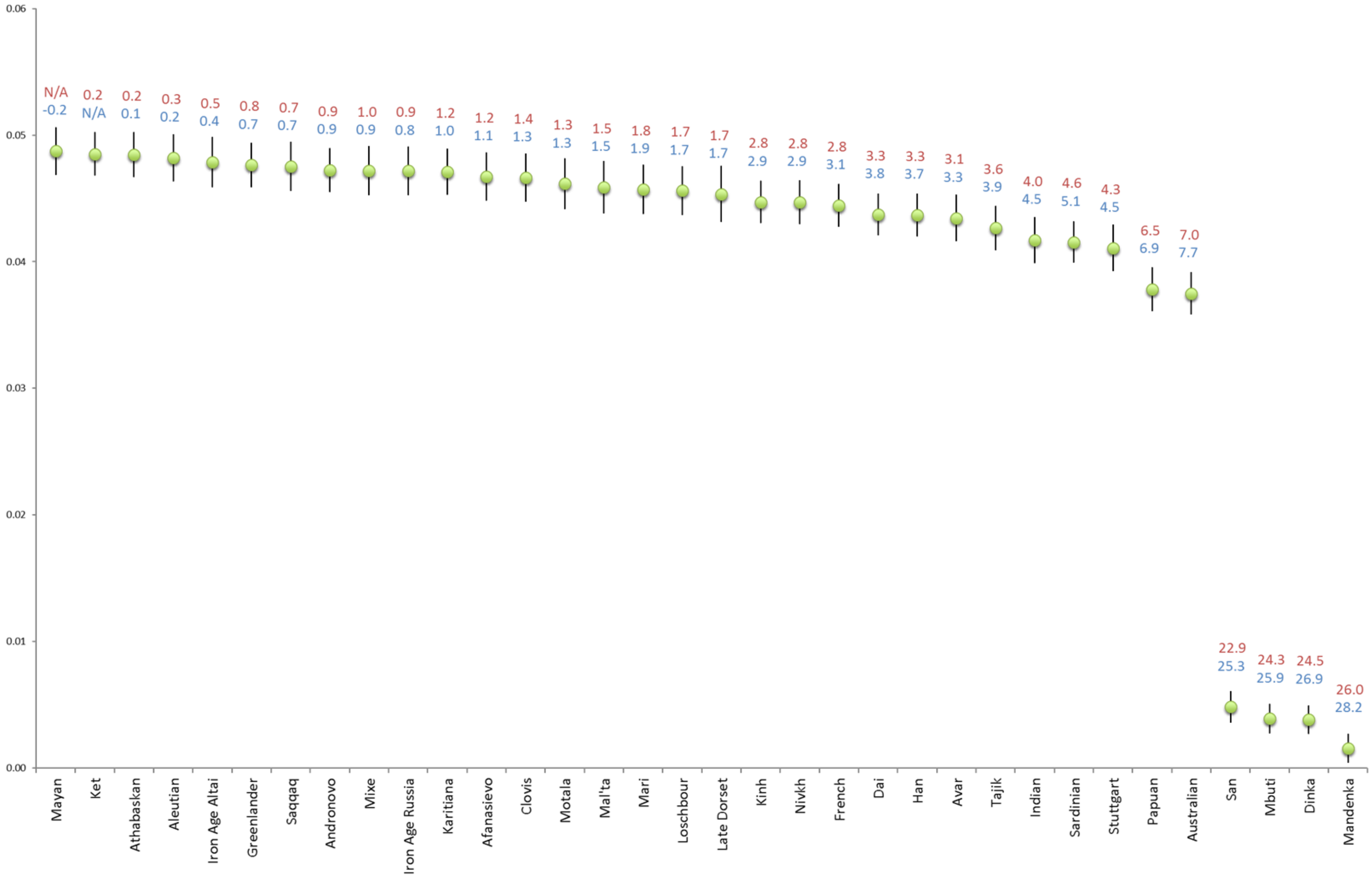

7.7. Statistics f_3(Yoruba; Athabaskan, X) computed on the dataset 'GenoChip + Illumina arrays' (21 Athabaskan individuals). **A.** Color-coded f_3(Yoruba; Athabaskan, X) values plotted on the world map using QGIS v.2.8. Top five values are shown in the bottom left corner.

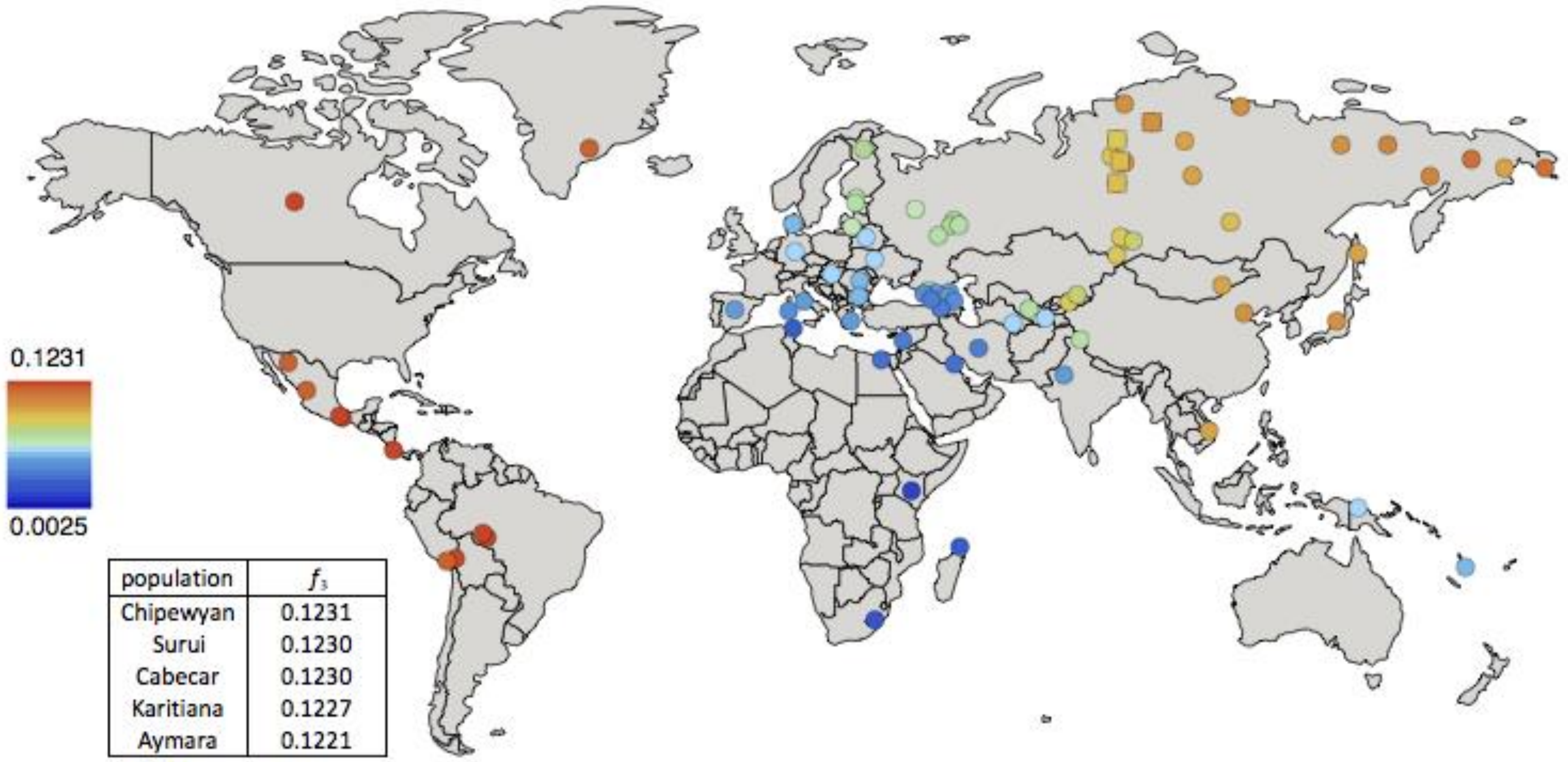

B. All f_3 values (green circles) sorted in descending order with their standard errors (single SE interval) shown by vertical lines. Absolute Z_{diff} scores >3.2 (plotted above the bars in red) show that a given f_3 statistic is significantly different from the lowest one, i.e. from that of the best hit. Bonferroni correction for 87 independent tests and a threshold p-value of 0.000575 were used. Z_{diff} scores in blue were calculated vs. the Ket population.

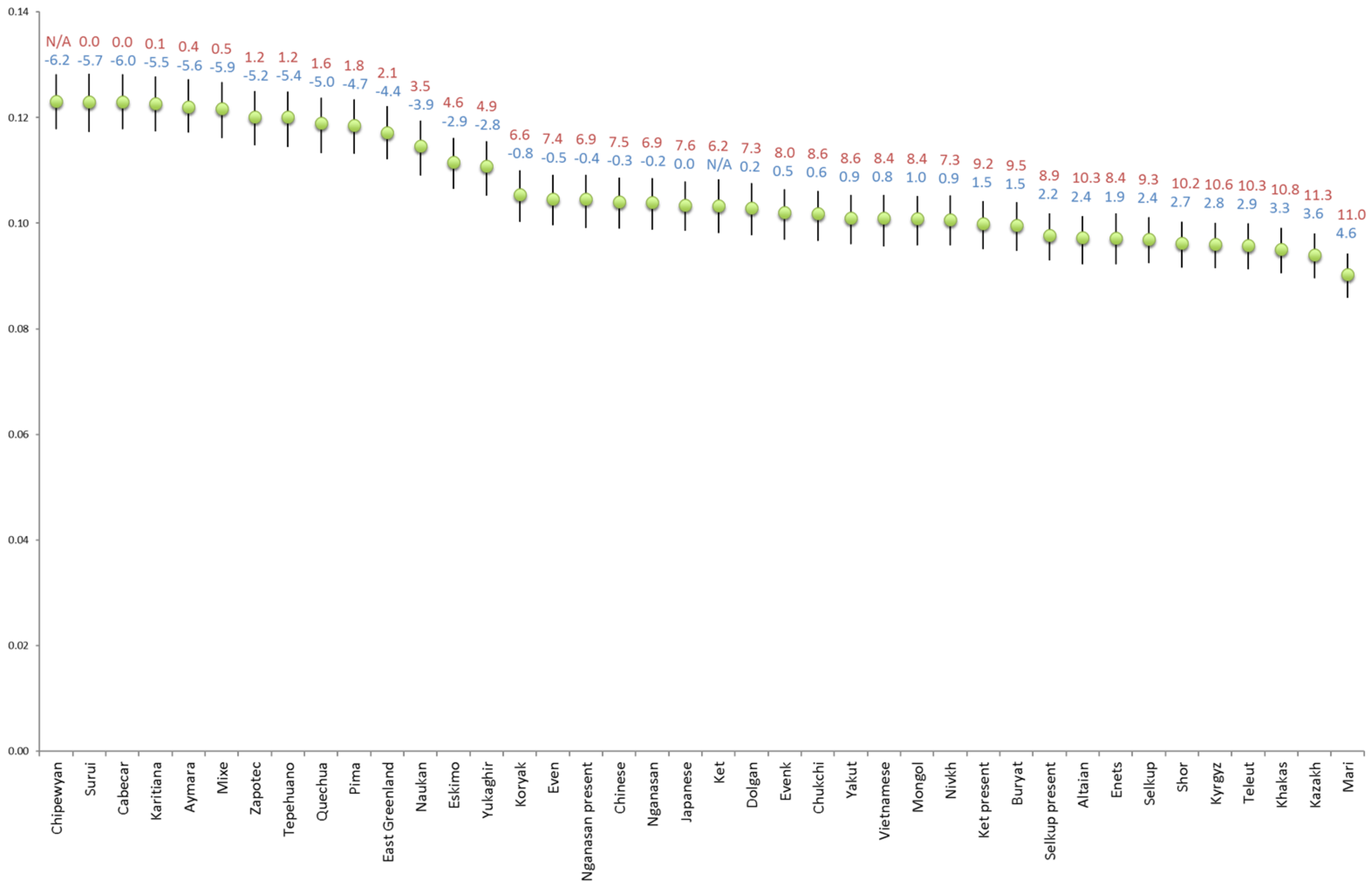

7.8. Statistics f_3(Yoruba; Chipewyan, X) computed on the dataset 'Ket genomes + HumanOrigins array + Verdu et al. 2014' (30 Chipewyan individuals). **A.** Color-coded f_3(Yoruba; Chipewyan, X) values plotted on the world map using QGIS v.2.8. Top five values are shown in the bottom left corner.

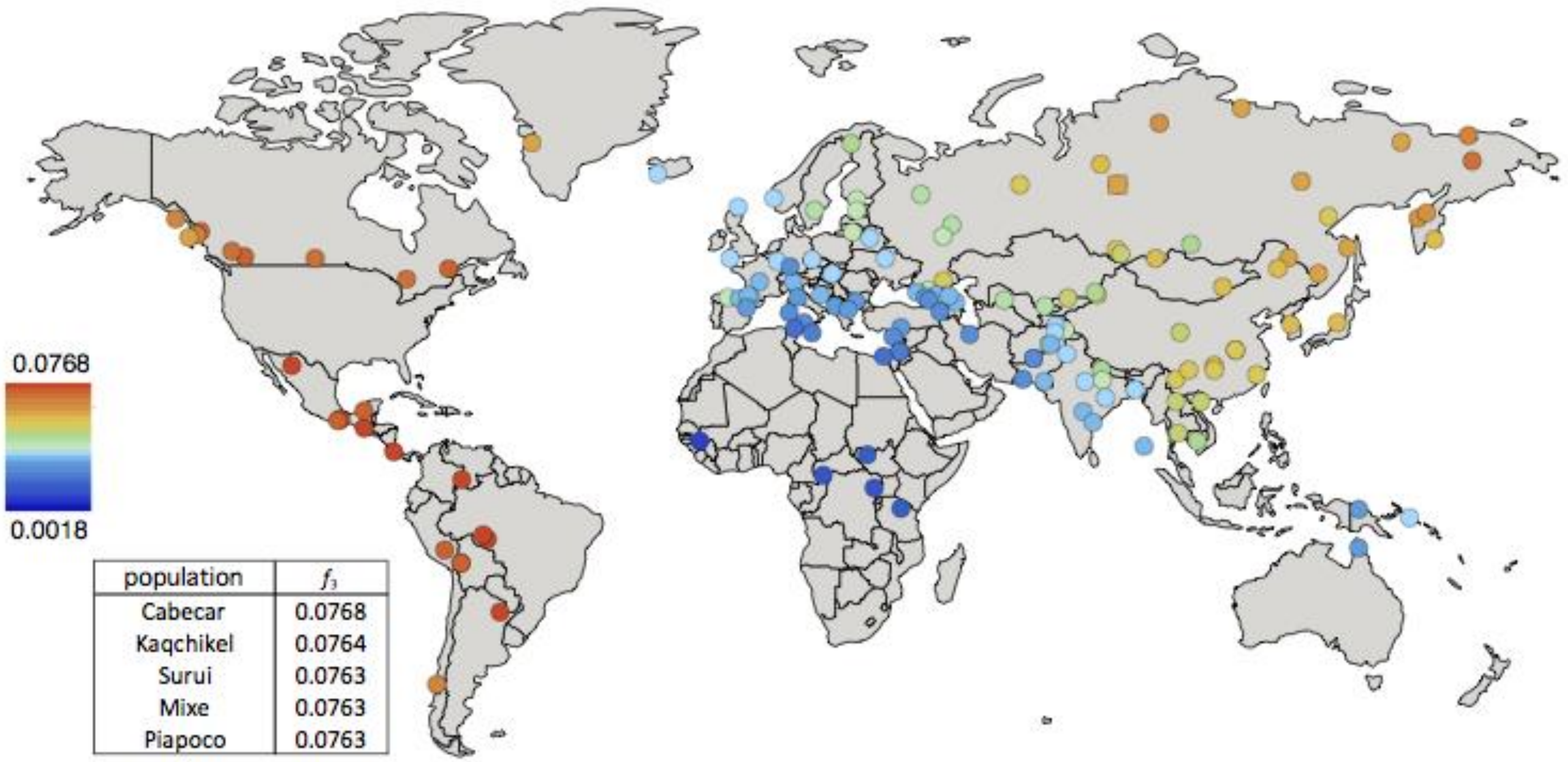

B. Top f_3 values (green circles) sorted in descending order with their standard errors (single SE interval) shown by vertical lines. Absolute Z_{diff} scores >3.4 (plotted above the bars in red) show that a given f_3 statistic is significantly different from the lowest one, i.e. from that of the best hit. Bonferroni correction for 142 independent tests and a threshold p-value of 0.000352 were used. Z_{diff} scores in blue were calculated vs. the Ket population.

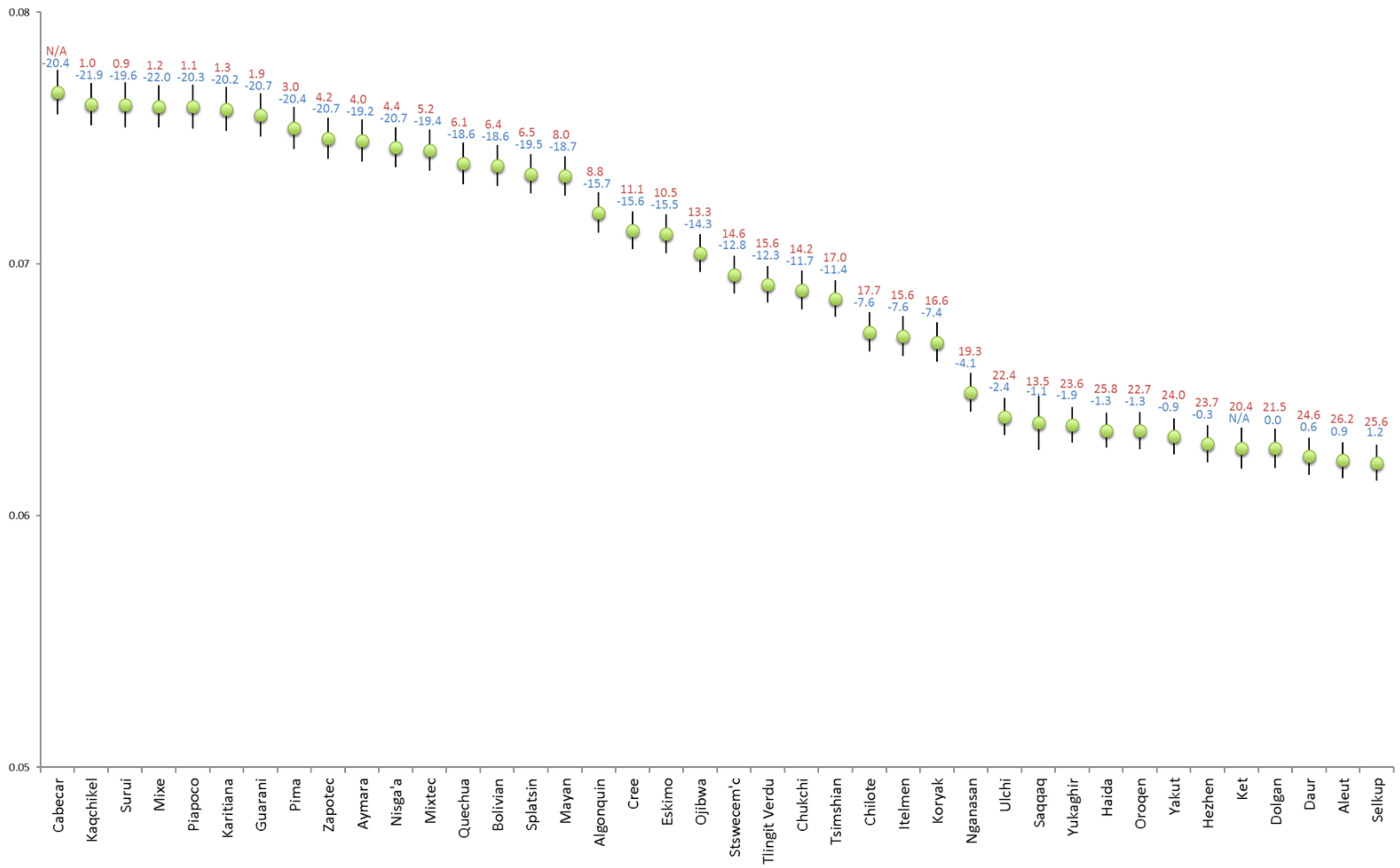

7.9. Statistics f_3(Yoruba; Haida, X) computed on the dataset 'Ket genomes + HumanOrigins array + Verdu et al. 2014' (10 Haida individuals). **A.** Color-coded f_3(Yoruba; Haida, X) values plotted on the world map using QGIS v.2.8. Top five values are shown in the bottom left corner.

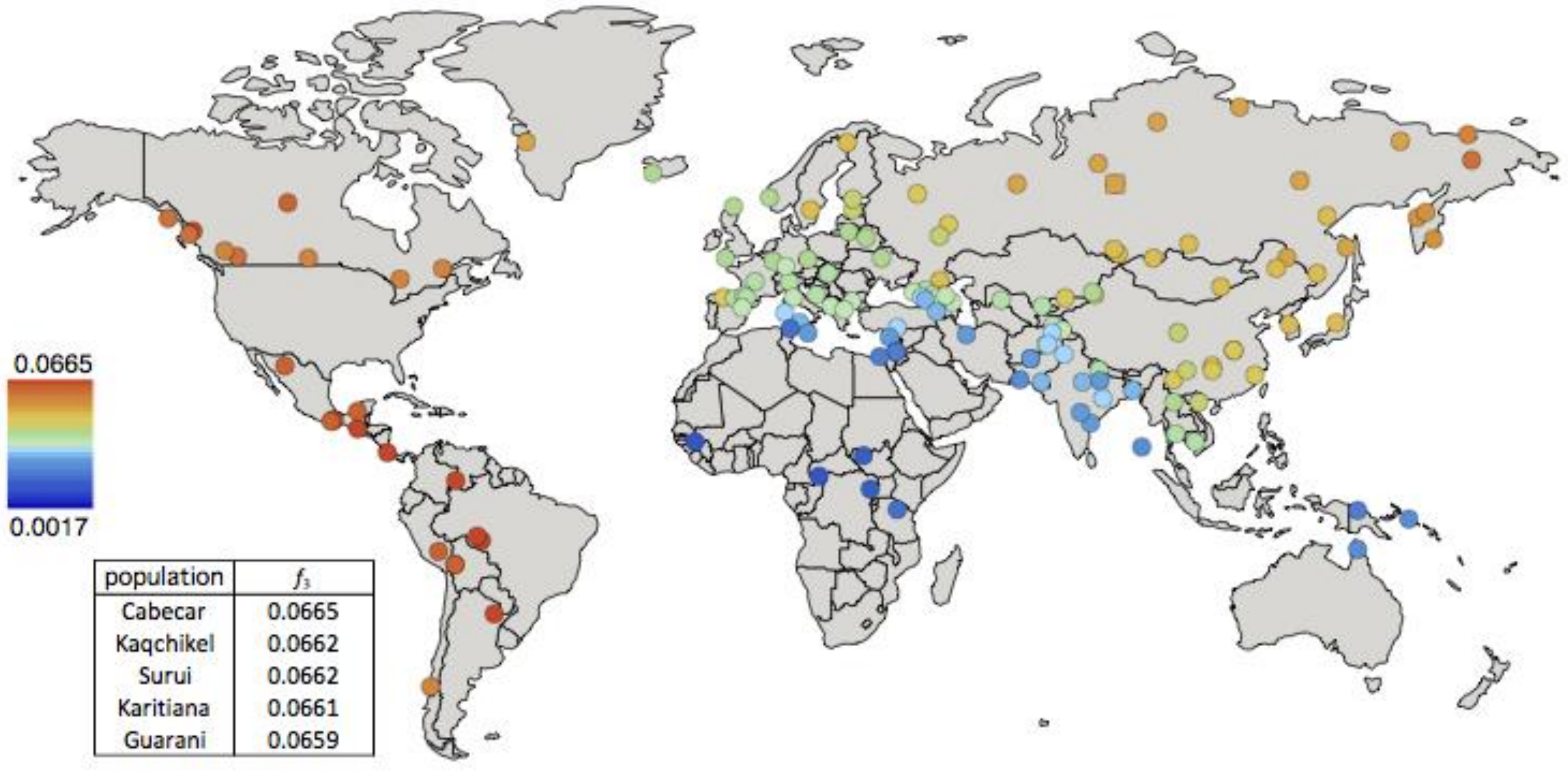

population	f_3
Cabecar	0.0665
Kaqchikel	0.0662
Surui	0.0662
Karitiana	0.0661
Guarani	0.0659

B. Top f_3 values (green circles) sorted in descending order with their standard errors (single SE interval) shown by vertical lines. Absolute Z_{diff} scores >3.4 (plotted above the bars in red) show that a given f_3 statistic is significantly different from the lowest one, i.e. from that of the best hit. Bonferroni correction for 142 independent tests and a threshold p-value of 0.000352 were used. Z_{diff} scores in blue were calculated vs. the Ket population.

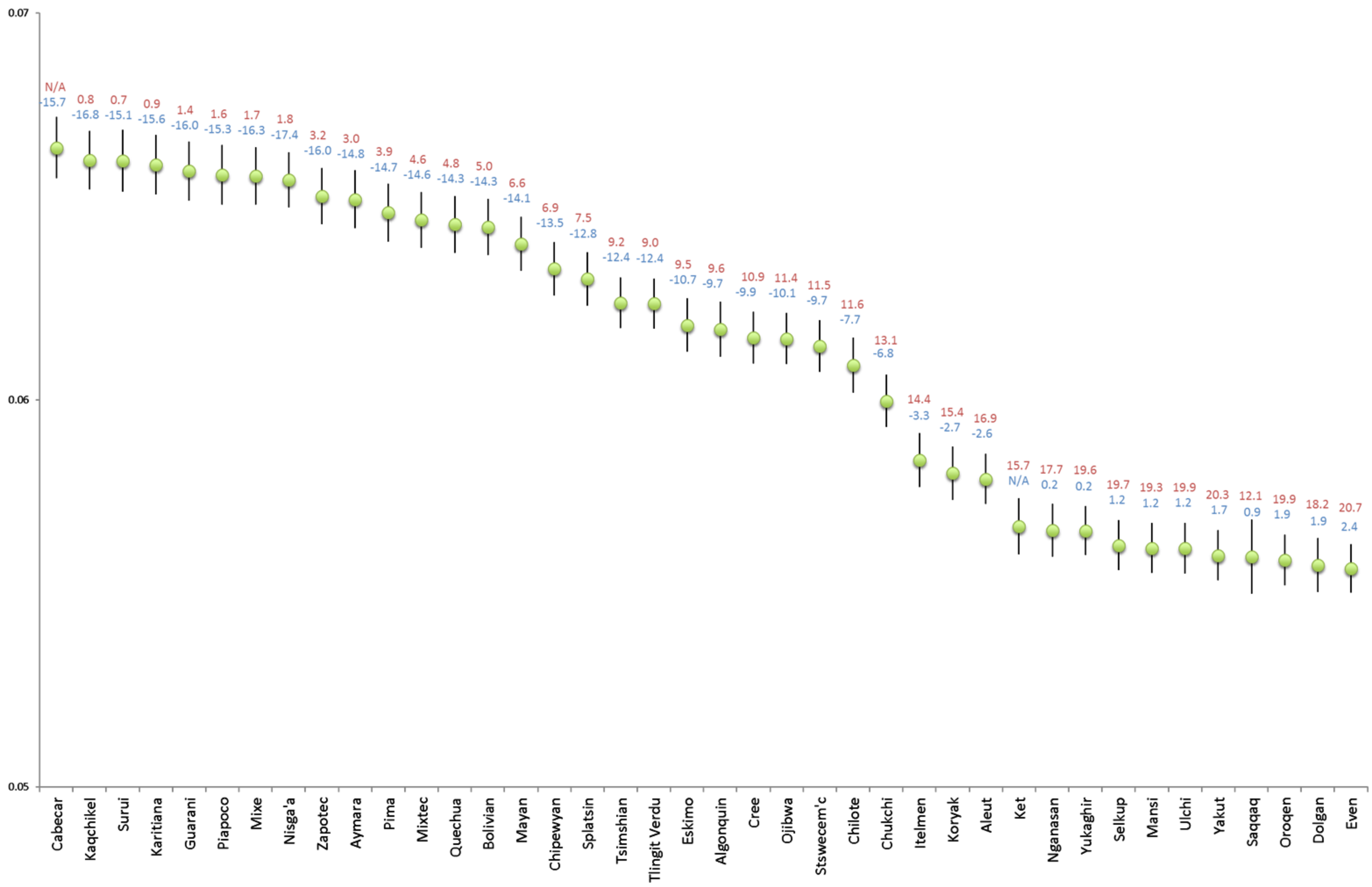

7.10. Statistics f_3(Yoruba; Tlingit, X) computed on the dataset 'Ket genomes + HumanOrigins array + Verdu et al. 2014' (16 Tlingit individuals). **A.** Color-coded f_3(Yoruba; Tlingit, X) values plotted on the world map using QGIS v.2.8. Top five values are shown in the bottom left corner.

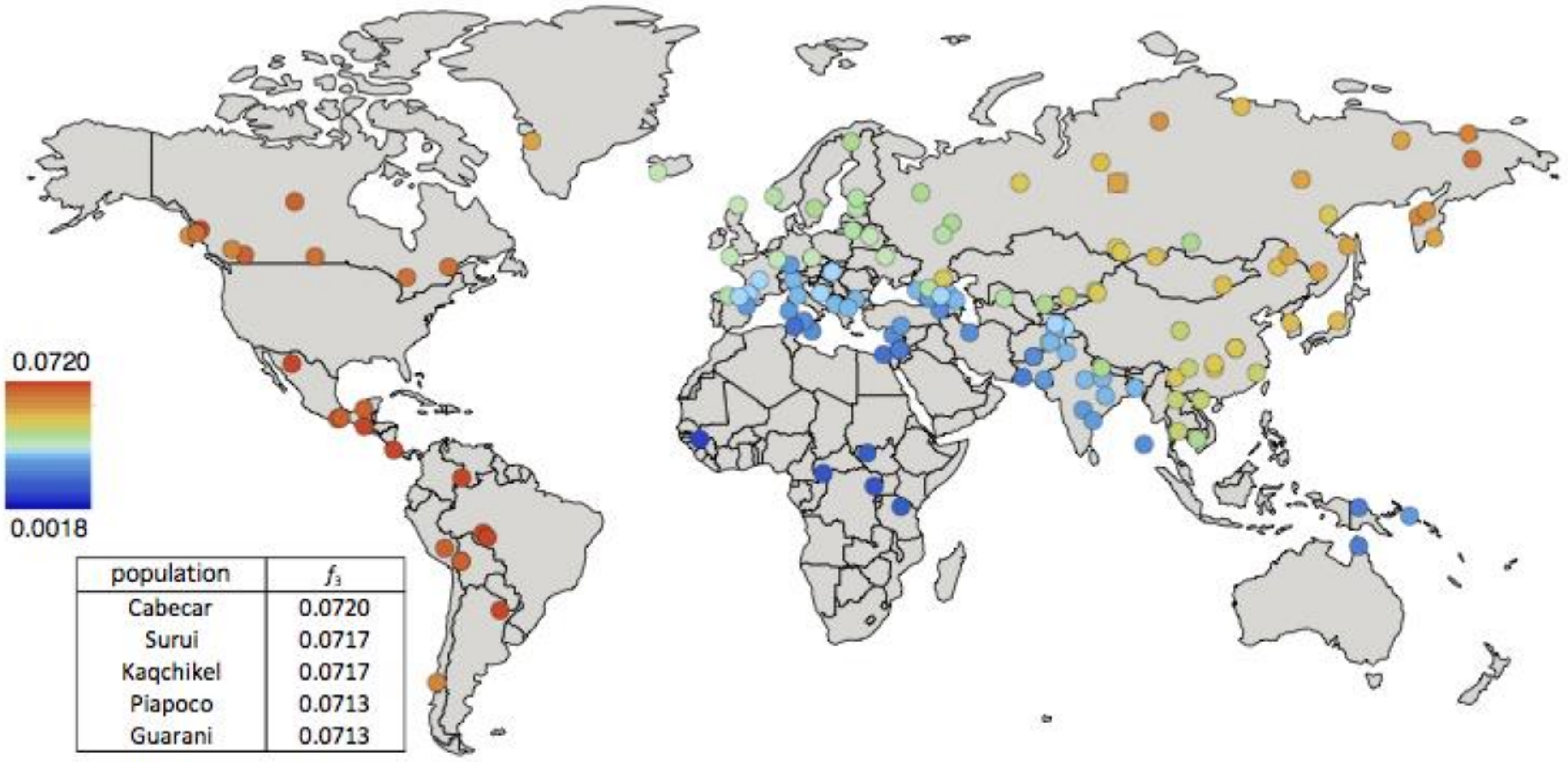

B. Top f_3 values (green circles) sorted in descending order with their standard errors (single SE interval) shown by vertical lines. Absolute Z_{diff} scores >3.4 (plotted above the bars in red) show that a given f_3 statistic is significantly different from the lowest one, i.e. from that of the best hit. Bonferroni correction for 142 independent tests and a threshold *p*-value of 0.000352 were used. Z_{diff} scores in blue were calculated vs. the Ket population.

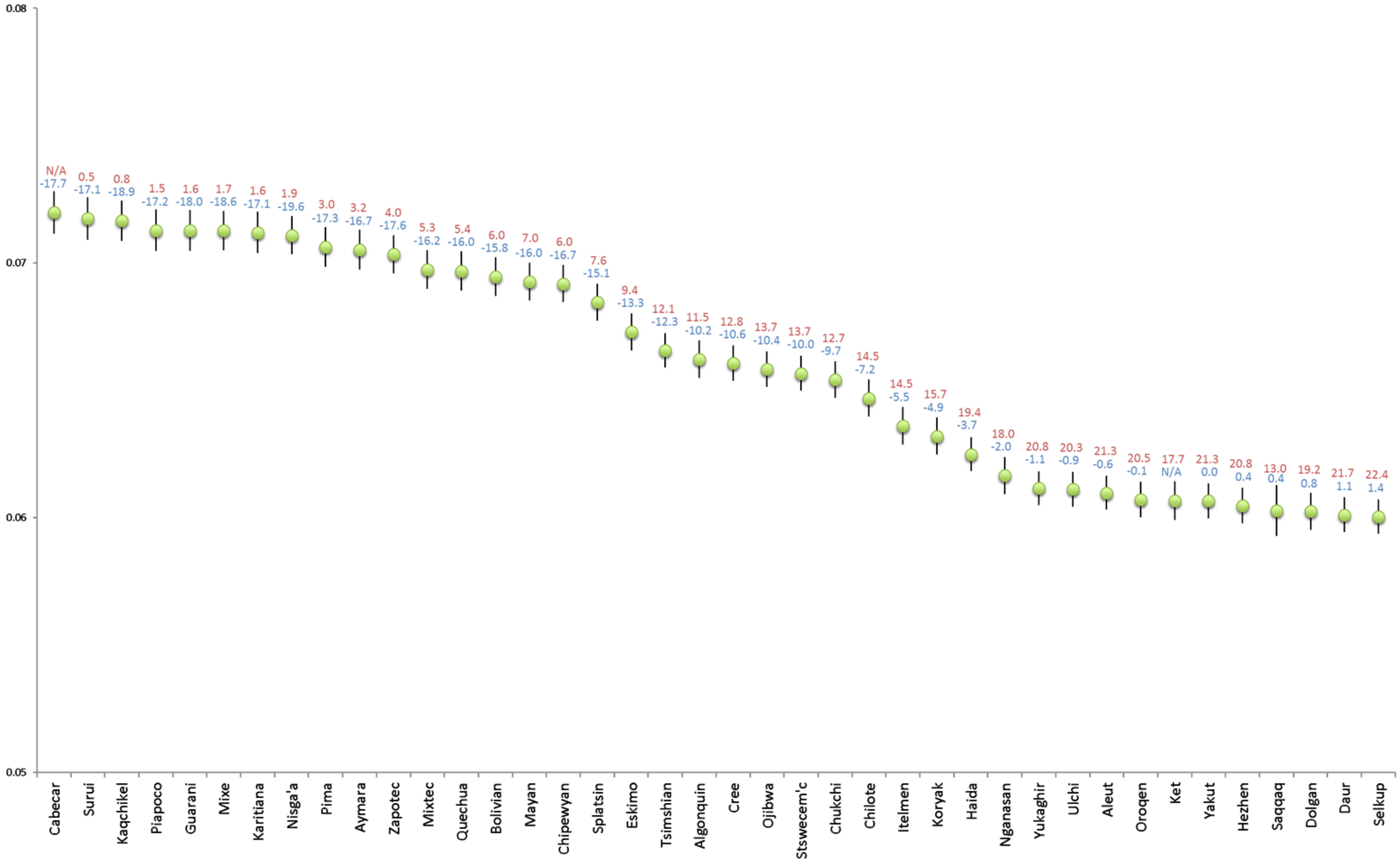

7.11. Statistics f_3(Yoruba; Athabaskan, X) computed on the genome-based dataset (2 Athabaskan individuals). All f_3 values (green circles) are sorted in descending order with their standard errors (single SE interval) shown by vertical lines. Absolute Z_{diff} scores >3 (plotted above the bars in red) show that a given f_3 statistic is significantly different from the lowest one, i.e. from that of the best hit. Bonferroni correction for 33 independent tests and a threshold p-value of 0.0015 were used. Z_{diff} scores in blue were calculated vs. the Ket population.

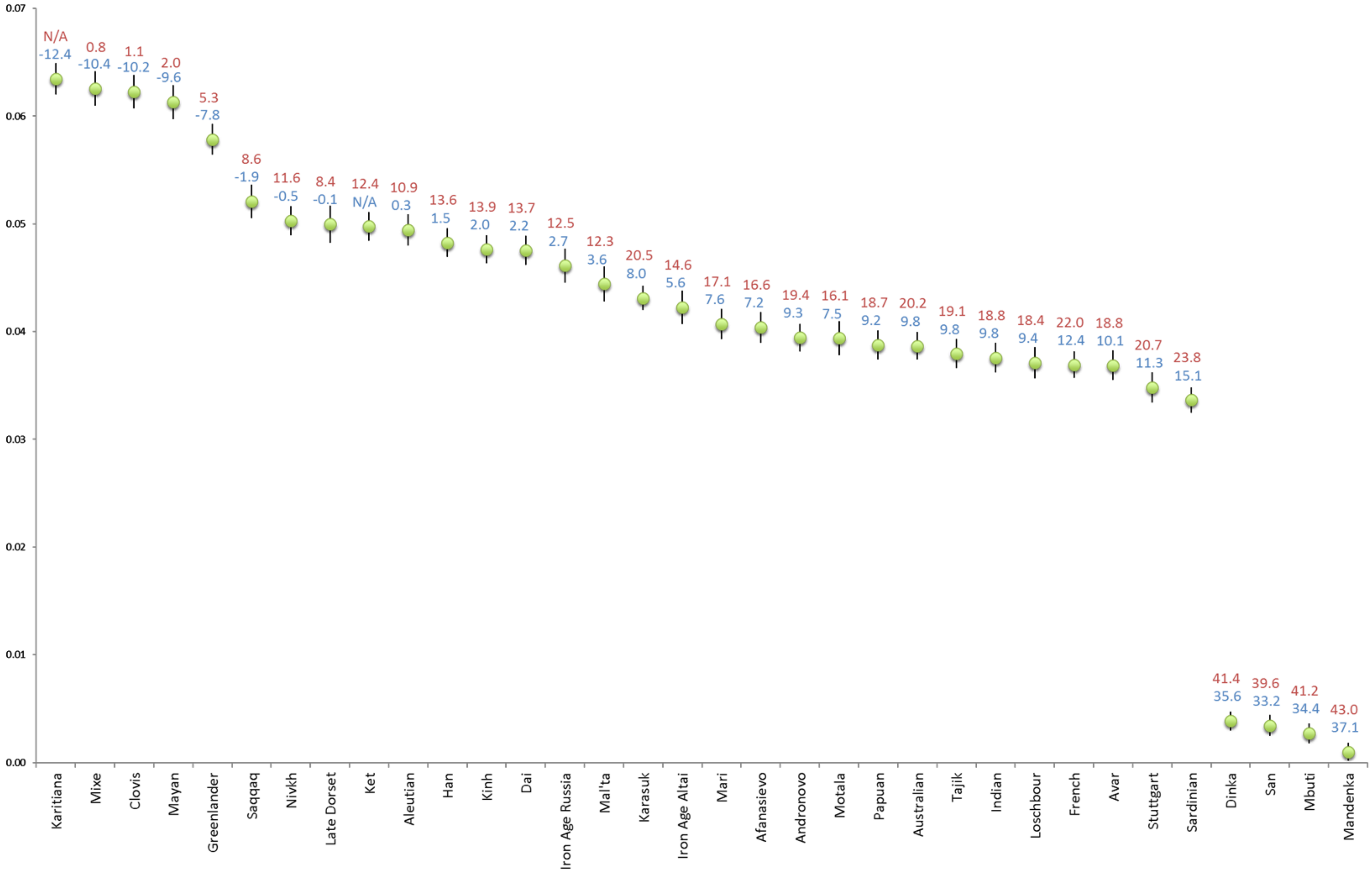

7.12. Statistics f_3(Yoruba; Athabaskan, X) computed on the genome-based dataset without transitions (2 Athabaskan individuals). All f_3 values (green circles) are sorted in descending order with their standard errors (single SE interval) shown by vertical lines. Absolute Z_{diff} scores >3 (plotted above the bars in red) show that a given f_3 statistic is significantly different from the lowest one, i.e. from that of the best hit. Bonferroni correction for 33 independent tests and a threshold p-value of 0.0015 were used. Z_{diff} scores in blue were calculated vs. the Ket population.

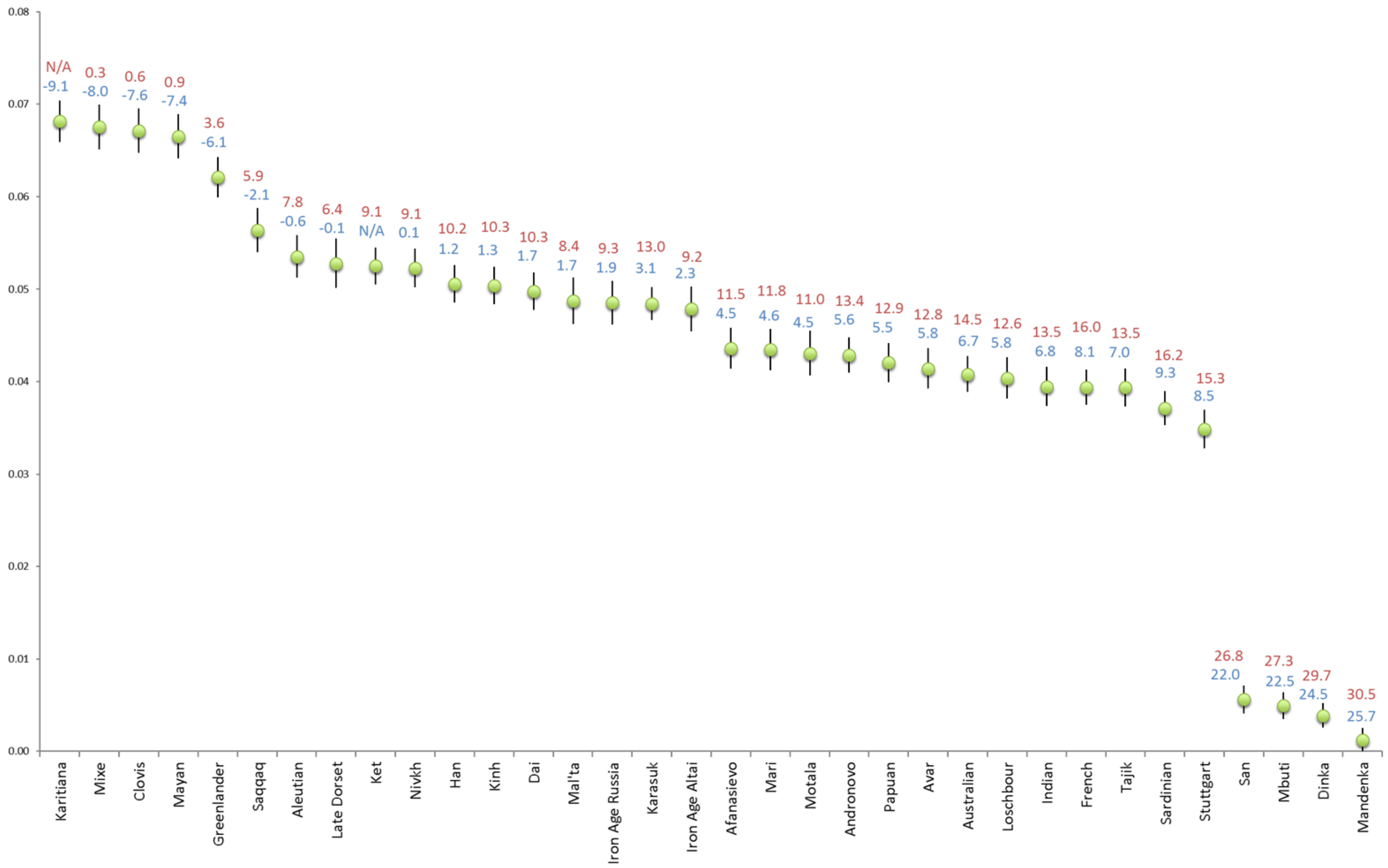

7.13. Statistics f_3(Yoruba; Mal'ta, X) computed on the dataset 'Ket genomes + HumanOrigins array'. **A.** Color-coded f_3 values plotted on the world map using QGIS v.2.8. Top five values are shown in the bottom left corner.

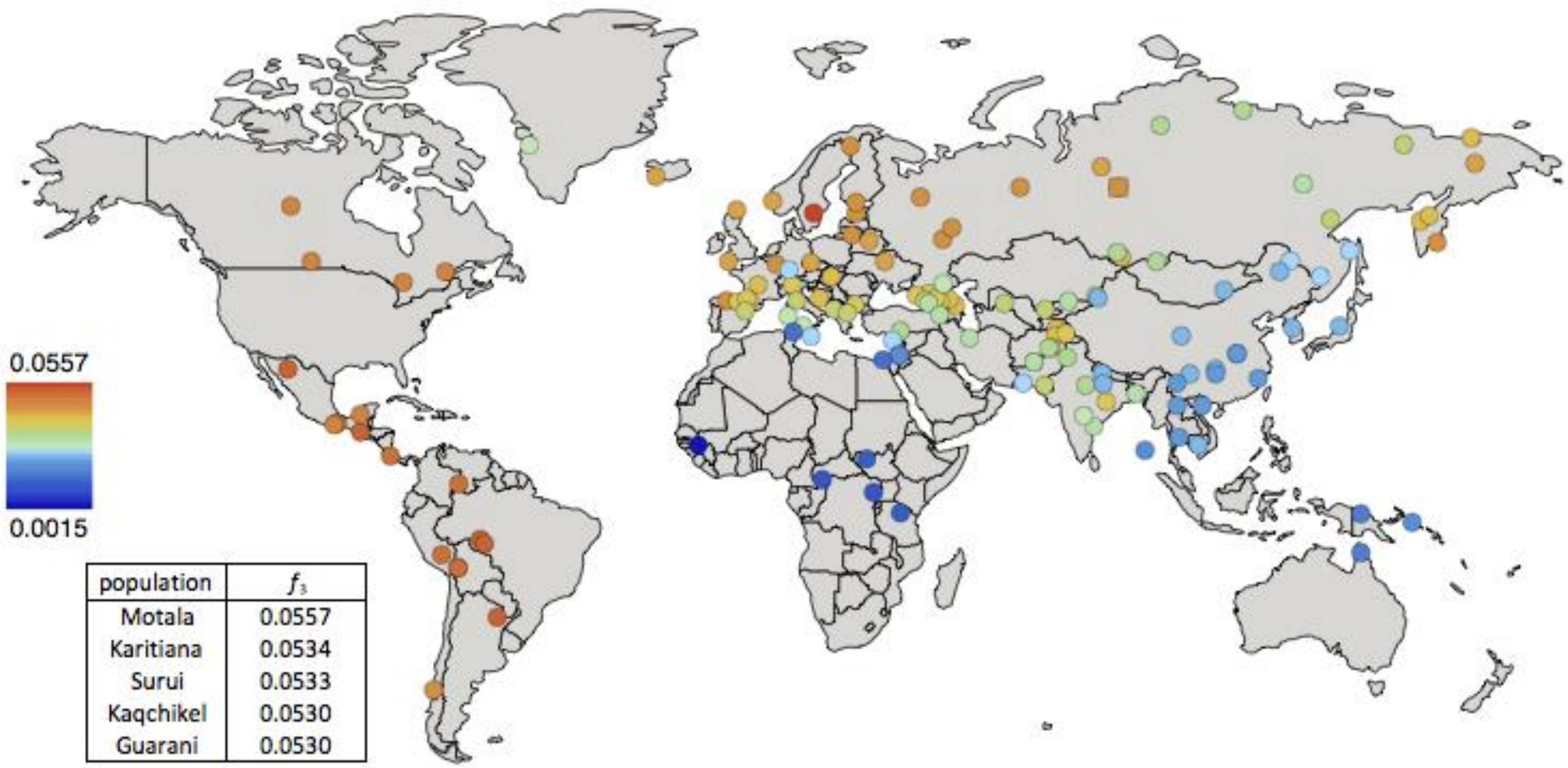

B. Top f_3 values (green circles) sorted in descending order with their standard errors (single SE interval) shown by vertical lines. Absolute Z_{diff} scores >3.4 (plotted above the bars in red) show that a given f_3 statistic is significantly different from the lowest one, i.e. from that of the best hit. Bonferroni correction for 136 independent tests and a threshold *p*-value of 0.000368 were used. Z_{diff} scores in blue were calculated vs. the Ket population.

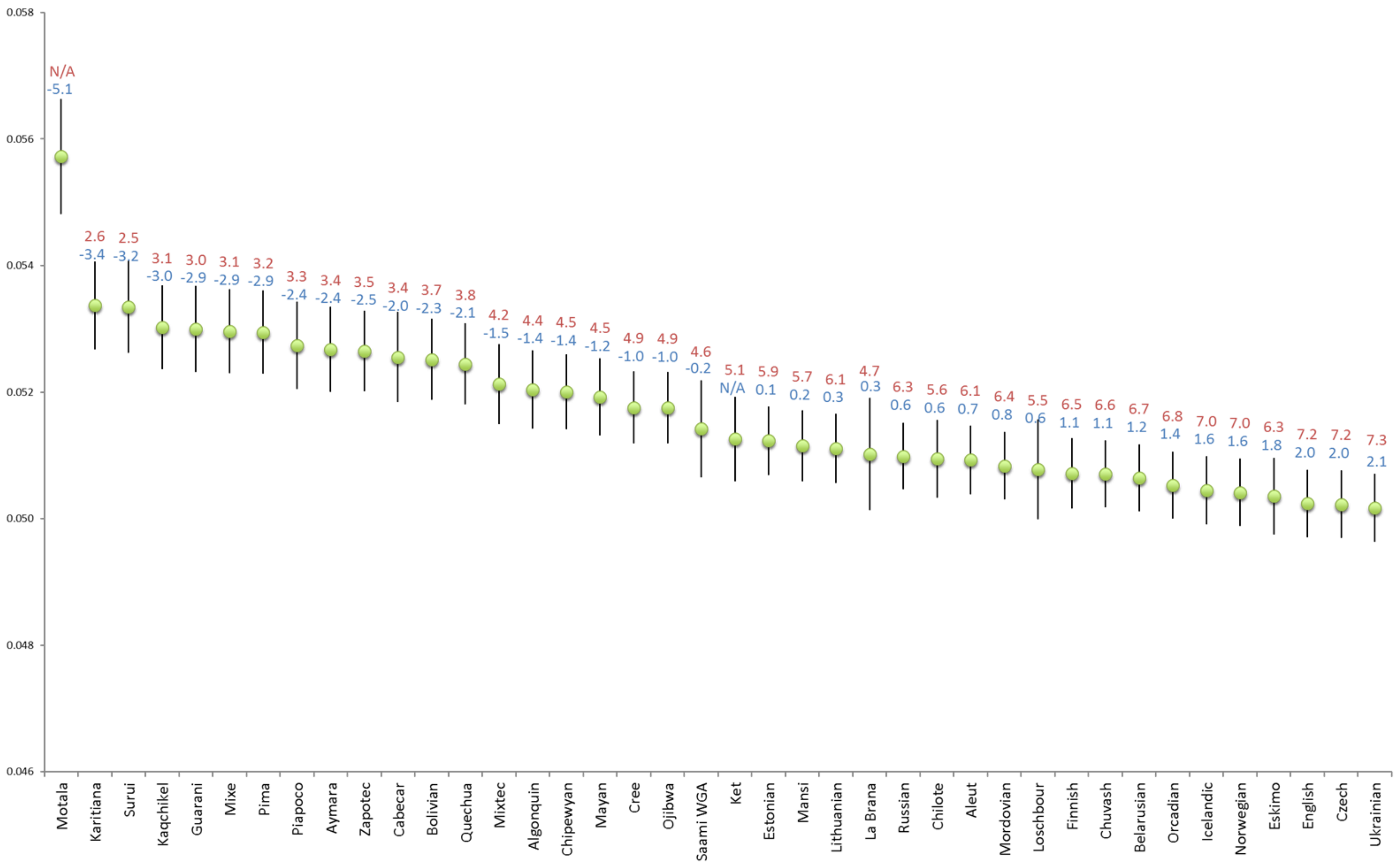

7.14. Statistics f_3(Yoruba; Mal'ta, X) computed on the dataset 'Ket genomes + HumanOrigins array' with individual Ket884 excluded. Top f_3 values (green circles) are sorted in descending order with their standard errors (single SE interval) shown by vertical lines. Absolute Z_{diff} scores >3.4 (plotted above the bars in red) show that a given f_3 statistic is significantly different from the lowest one, i.e. from that of the best hit. Bonferroni correction for 136 independent tests and a threshold p-value of 0.000368 were used. Z_{diff} scores in blue were calculated vs. the Ket population.

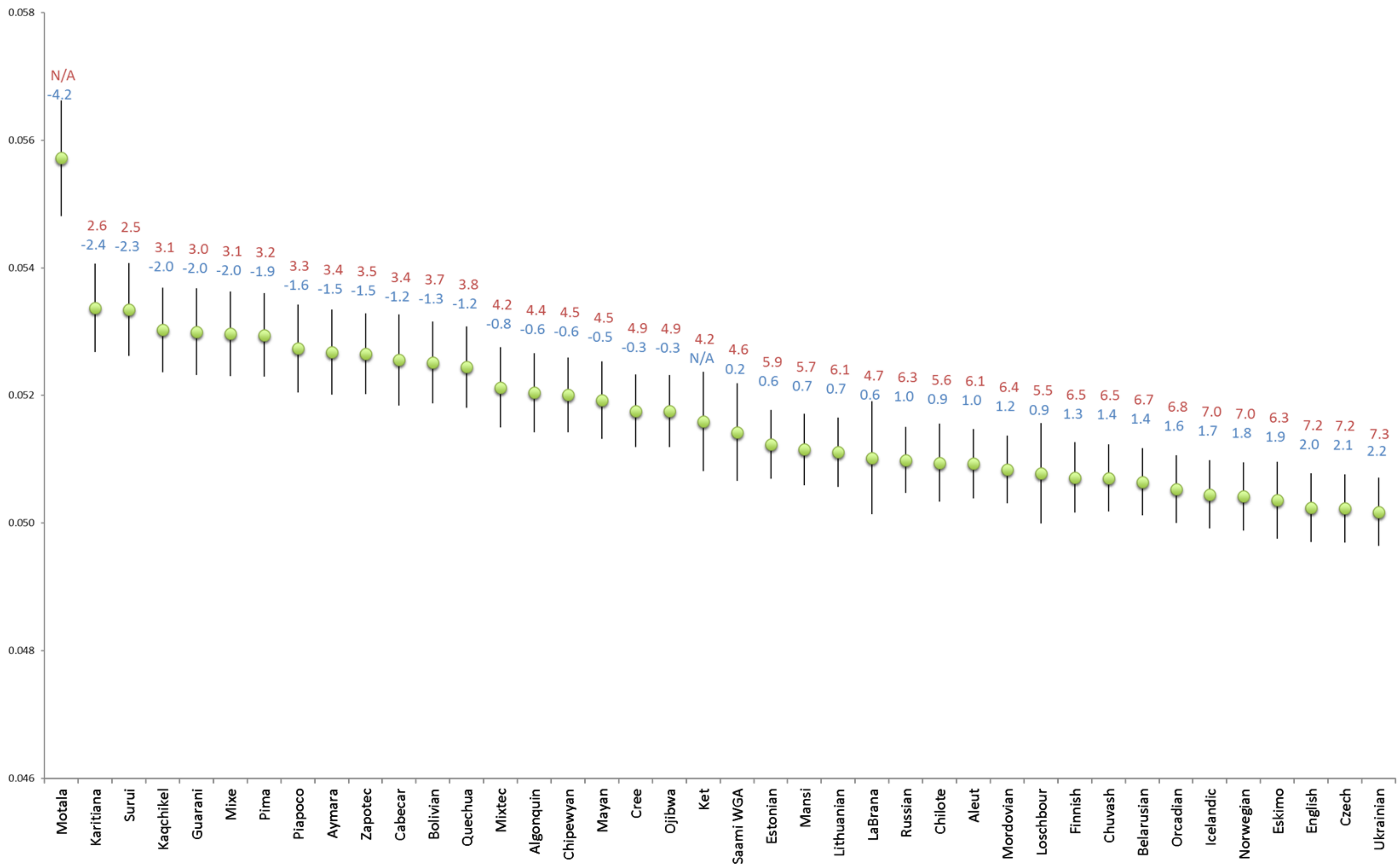

7.15 Statistics f_3(Yoruba; Mal'ta, X) computed on the genome-based dataset. f_3 values (green circles) are sorted in descending order with their standard errors (single SE interval) shown by vertical lines. Absolute Z_{diff} scores >3 (plotted above the bars in red) show that a given f_3 statistic is significantly different from the lowest one, i.e. from that of the best hit. Bonferroni correction for 33 independent tests and a threshold p-value of 0.0015 were used. Z_{diff} scores in blue were calculated vs. the Ket population.

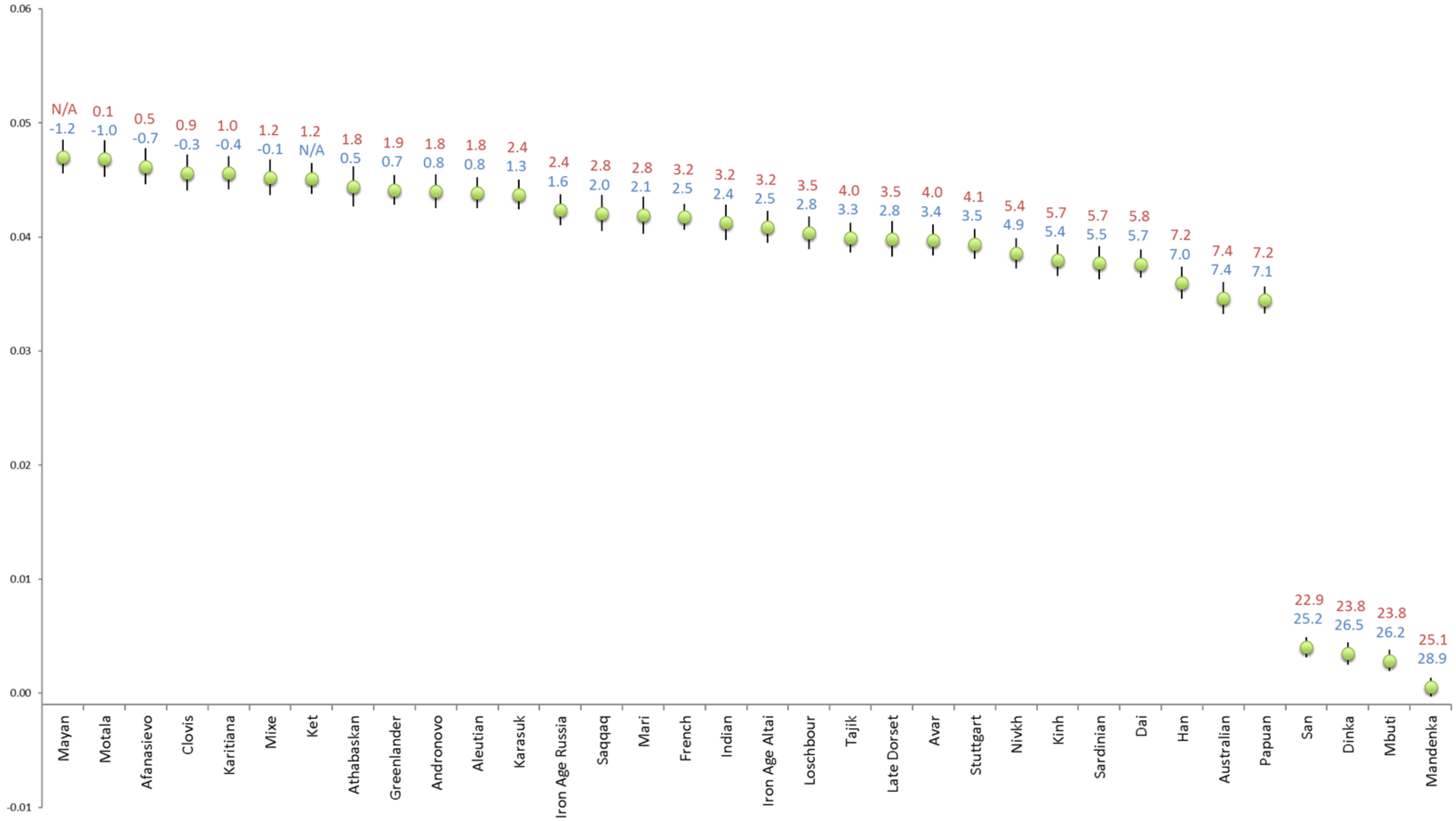

7.16. Statistics f_3(Yoruba; Mal'ta, X) computed on the genome-based dataset without transitions. All f_3 values (green circles) are sorted in descending order with their standard errors (single SE interval) shown by vertical lines. Absolute Z_{diff} scores >3 (plotted above the bars in red) show that a given f_3 statistic is significantly different from the lowest one, i.e. from that of the best hit. Bonferroni correction for 33 independent tests and a threshold *p*-value of 0.0015 were used. Z_{diff} scores in blue were calculated vs. the Ket population.

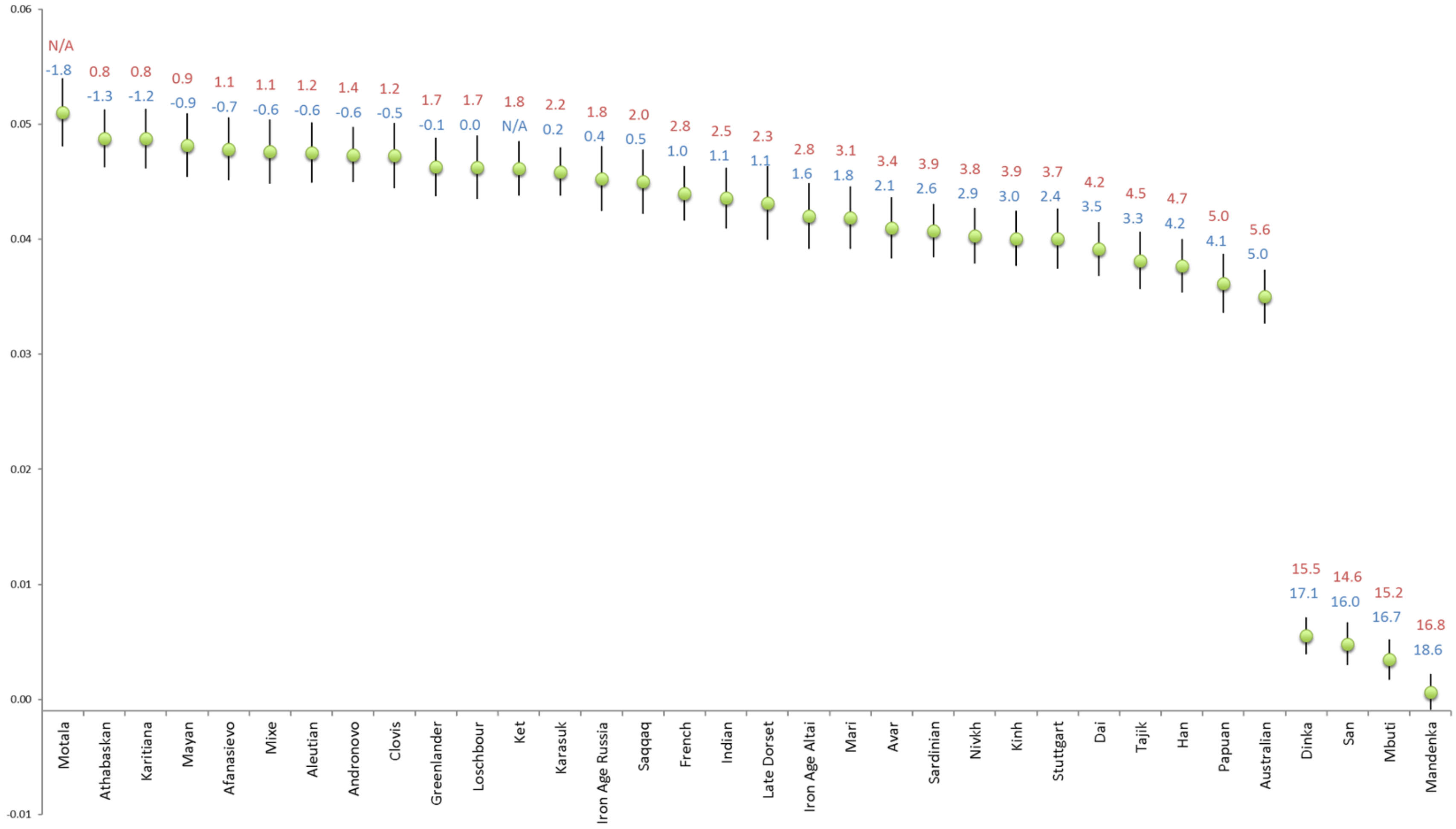

7.17. Statistics f_3(Yoruba; Saqqaq, X) computed on the dataset 'Ket genomes + HumanOrigins array'. **A.** Color-coded f_3(Yoruba; Saqqaq, X) values plotted on the world map using QGIS v.2.8. Top five values are shown in the bottom left corner.

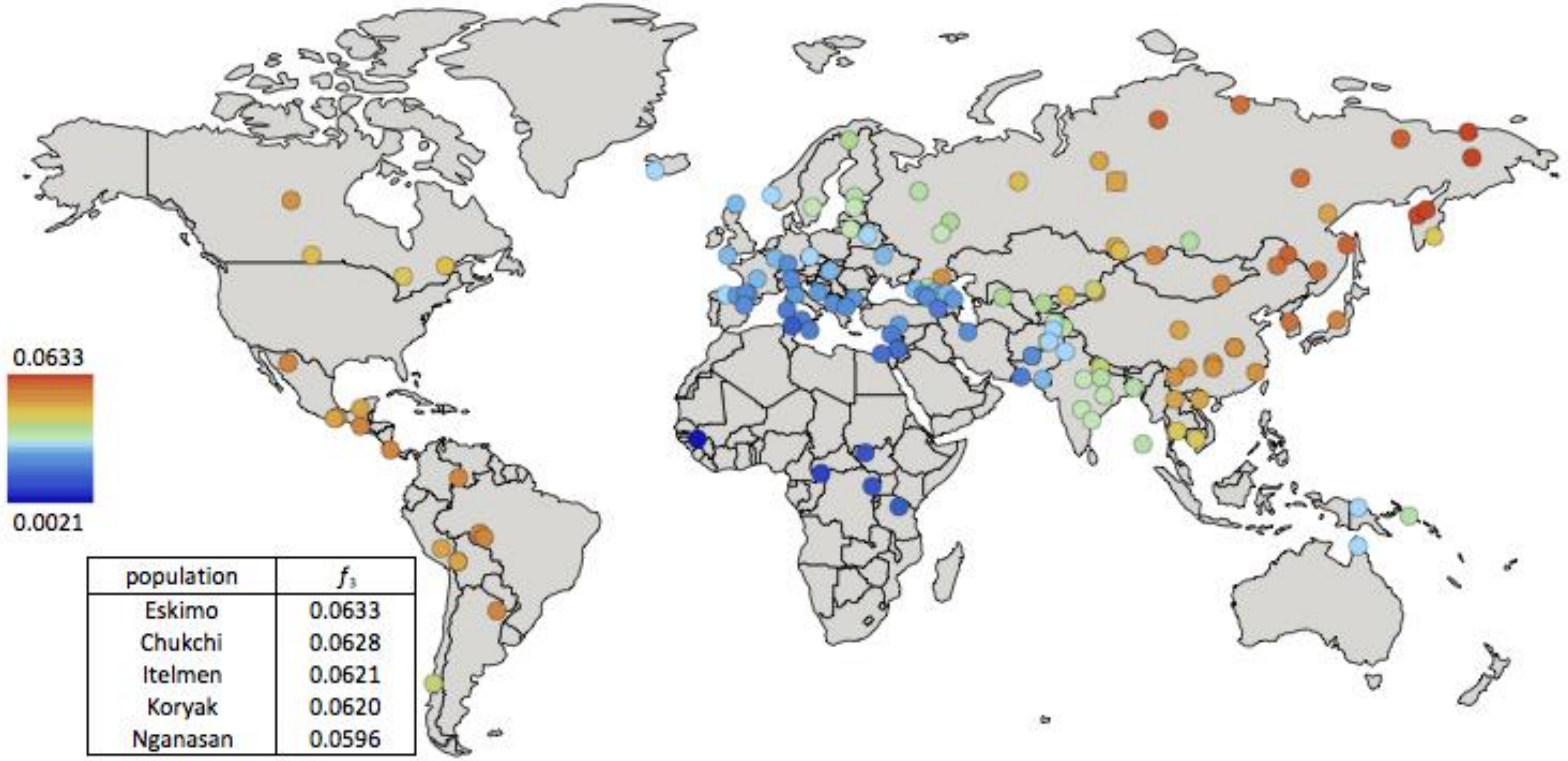

B. Top f_3 values (green circles) sorted in descending order with their standard errors (single SE interval) shown by vertical lines. Absolute Z_{diff} scores >3.4 (plotted above the bars in red) show that a given f_3 statistic is significantly different from the lowest one, i.e. from that of the best hit. Bonferroni correction for 136 independent tests and a threshold p-value of 0.000368 were used. Z_{diff} scores in blue were calculated vs. the Nganasan population.

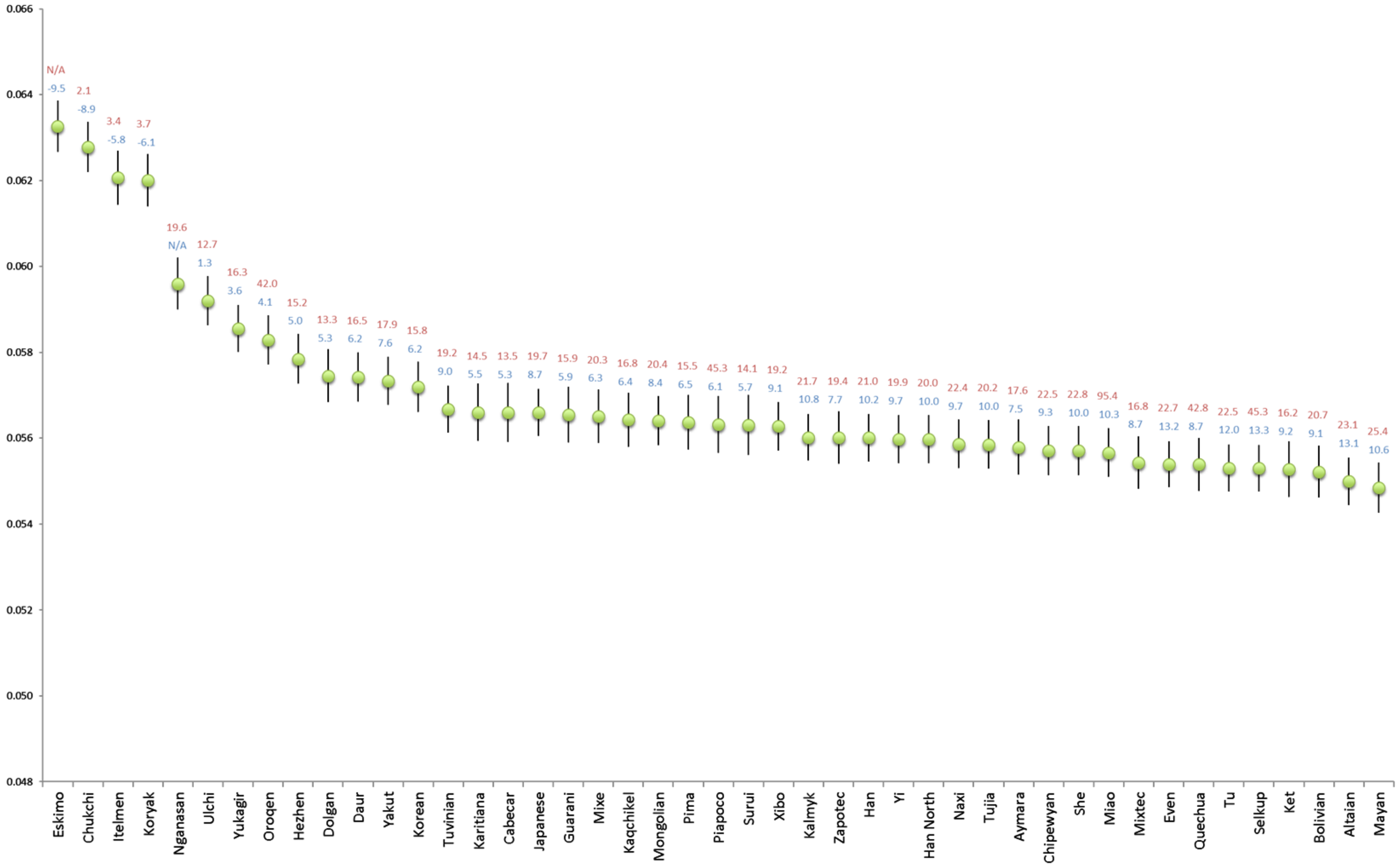

7.18. Statistics f_3(Yoruba; Saqqaq, X) computed on the genome-based dataset. All f_3 values (green circles) are sorted in descending order with their standard errors (single SE interval) shown by vertical lines. Absolute Z_{diff} scores >3 (plotted above the bars in red) show that a given f_3 statistic is significantly different from the lowest one, i.e. from that of the best hit. Bonferroni correction for 33 independent tests and a threshold *p*-value of 0.0015 were used. Z_{diff} scores in blue were calculated vs. the Ket population.

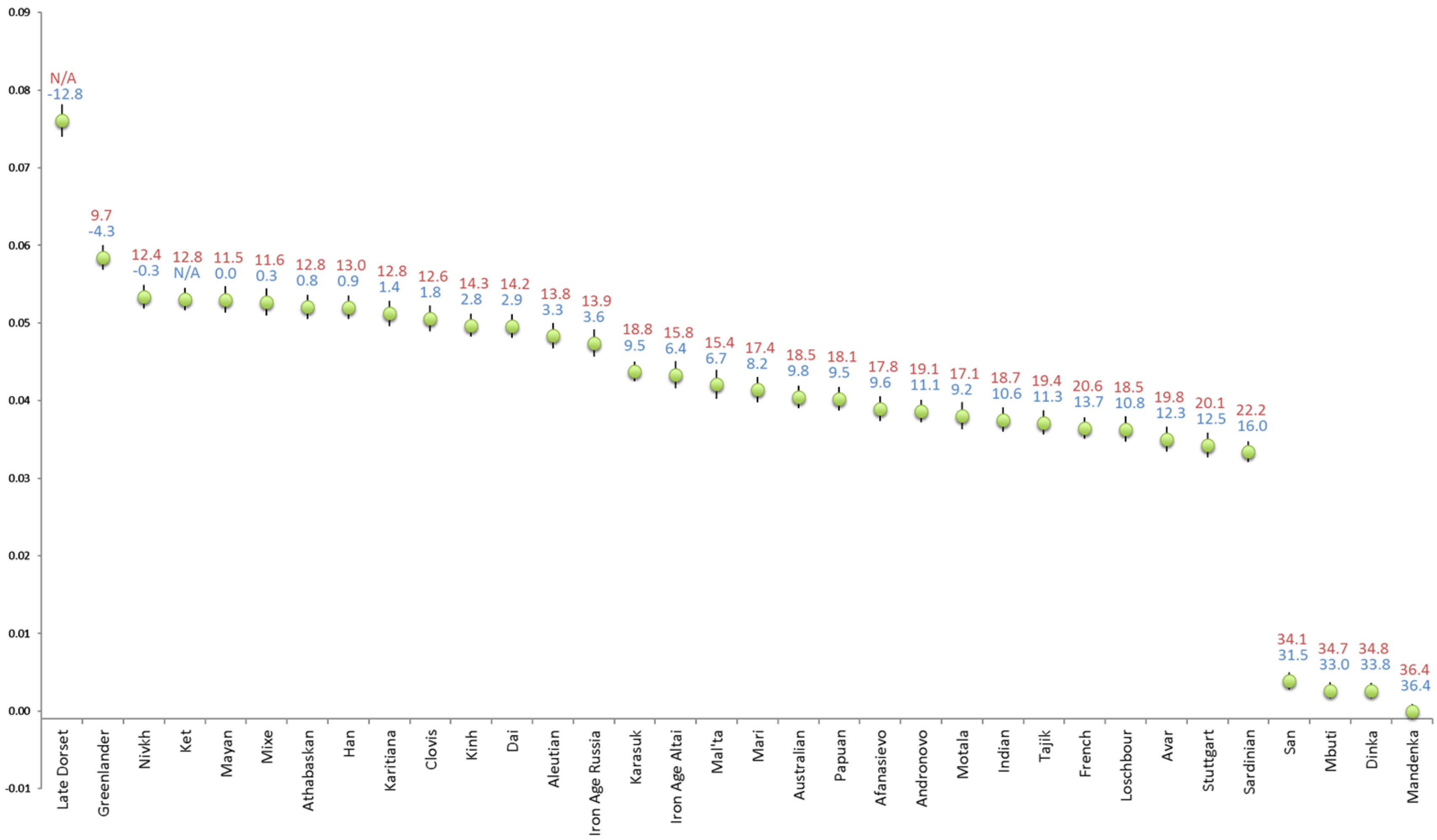

7.19. Statistics f_3(Yoruba; Saqqaq, X) computed on the genome-based dataset without transitions. All f_3 values (green circles) are sorted in descending order with their standard errors (single SE interval) shown by vertical lines. Absolute Z_{diff} scores >3 (plotted above the bars in red) show that a given f_3 statistic is significantly different from the lowest one, i.e. from that of the best hit. Bonferroni correction for 33 independent tests and a threshold *p*-value of 0.0015 were used. Z_{diff} scores in blue were calculated vs. the Ket population.

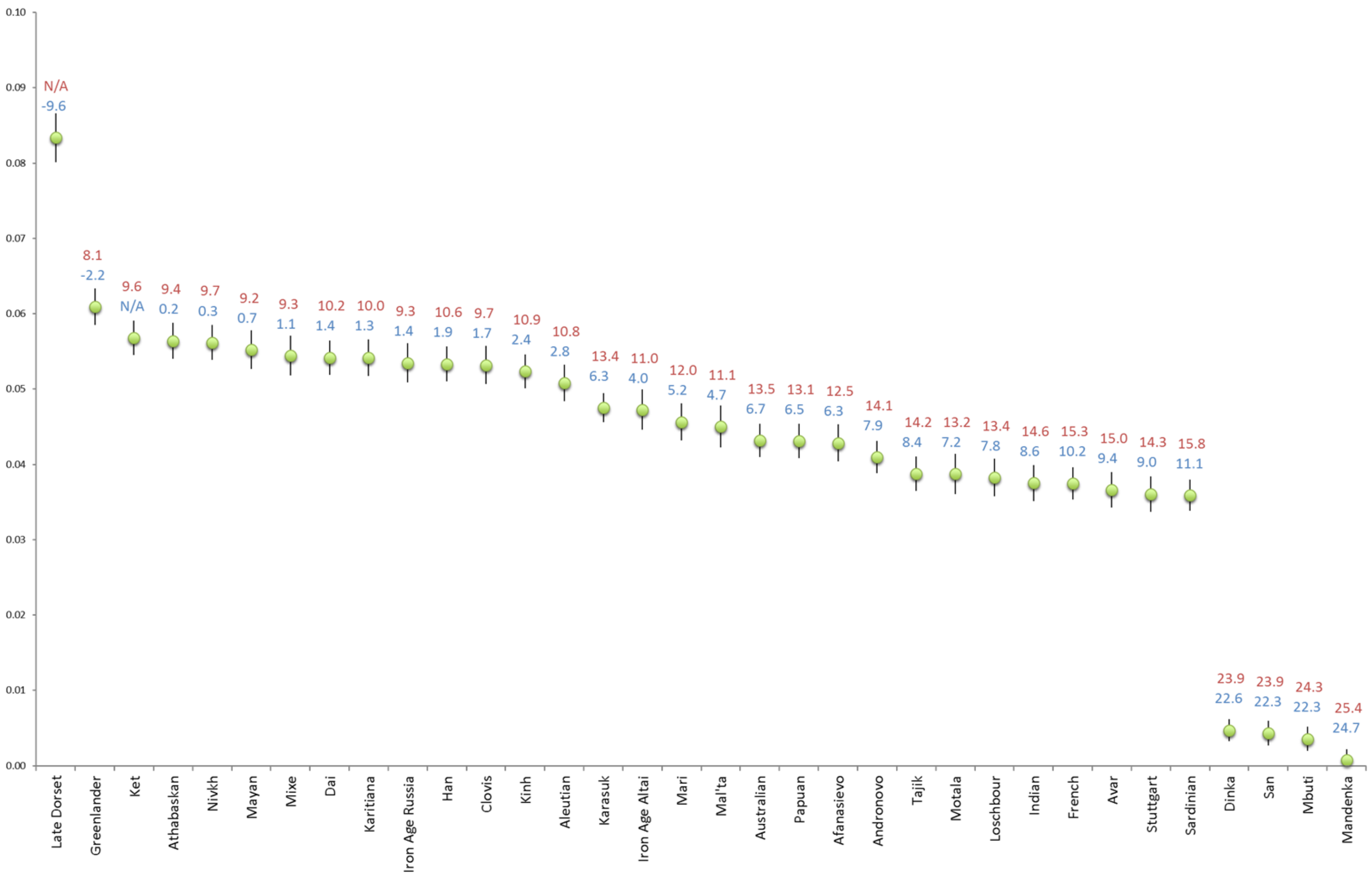

8. *f_4 statistic*

Mal'ta (ancient North Eurasian) ancestry in Kets

Statistic of the form f_4(X, Chimp; Mal'ta, Stuttgart), reproducing the approach used in Lazaridis et al. (2014), tests whether a population X has more drift shared with Mal'ta (ANE) or with Stuttgart, an early European farmer (EEF, Lazaridis et al. 2014). The value of f_4(Ket884+891, Chimp; Mal'ta, Stuttgart) on the dataset 'Ket genomes + HumanOrigins array' fell within the range of North American and Beringian populations (Suppl. Fig. 8.1), and Z=score for f_4(Ket, Chimp; Mal'ta, Stuttgart) equaled 3.4. A similar result was produced on two versions of the genome-based dataset (with and without transitions), where Kets demonstrated f_4 statistics similar to those of Greenlanders, Saqqaq and Late Dorset ancient genomes (Raghavan et al. 2014b) (Suppl. Figs. 8.2, 8.3, Yoruba was used as an outgroup instead of Chimp). And this result was produced again with a different f_4 set-up (X, Papuan; Sardinian, Mal'ta) (Seguin-Orlando et al. 2014), on all datasets tested (Suppl. Figs. 8.4-8.6). Kets were significantly closer ($|Z| > 3$) to Mal'ta as compared to Stuttgart or Sardinians in all cases, except for f_4(X, Papuan; Sardinian, Mal'ta) calculated on the HumanOrigins-based dataset and on the genome-based dataset without transitions (Suppl. Figs. 8.4, 8.6).

Position of Kets in the 'gradients' between Mal'ta and west European hunter-gatherers (WHG, Lazaridis et al. 2014) and between WHG and EEF was determined with the following f_4 statistics: f_4(X, Chimp or Yoruba; Mal'ta, Loschbour) and f_4(X, Chimp or Yoruba; Loschbour, Stuttgart), respectively (Suppl. Figs. 8.7-8.12). Kets were equidistant from Mal'ta and Loschbour in the HumanOrigins-based dataset, which was manifested by a Z-score of 0.62 for (Ket, Chimp; Mal'ta, Loschbour) (Suppl. Fig. 8.7). However, a Z-score of 3.4 for f_4(Ket, Yoruba; Mal'ta, Loschbour) calculated on the original genome-based dataset (Suppl. Fig. 8.8) suggested that Kets were significantly closer to ANE than to WHG, and emphasized differences between the array-based and the genome-based datasets.

All possible population pairs (X,Y) were tested with f_4(Mal'ta, Yoruba; Y, X) on the genome-based datasets (Fig. 5A, Suppl. Fig. 8.15). Most Z-scores for f_4(Mal'ta, Yoruba; Ket, X) were non-significant ($|Z| < 2.9$ under the multiple-testing correction, Fig. 5A), except for Avar, Dai, Han, Kinh, Nivkh, Sardinian, Stuttgart, and Tajik, i.e. populations with a large proportion of EEF or East Asian ancestry. There were no Z-scores < -2.9 (and even < -2), consistent with any population being significantly closer to Mal'ta. Similarly, in the case of the genome-based dataset without transitions there were no Z-scores < -2 for f_4(Mal'ta, Yoruba; Ket, X), and scores for Clovis, Greenlander, Iron Age Russia, Karasuk, Loschbour, and Saqqaq ranged from -0.5 to 0.5 (Suppl. Fig. 8.15), which was consistent with Kets forming a robust clade with any of these populations relative to Mal'ta.

Statistics f_4(Ket, Yoruba; Mal'ta, X) on the genome-based dataset (Fig. 5B) were compatible with Kets being equidistant from Mal'ta and Aleutian, Dai, Han, Iron Age Altai, Iron Age Russia, Karasuk, Kinh, Mari, Motala12, and Nivkh ($|Z| < 2$), whereas Kets were significantly closer to Mal'ta ($Z > 2.9$) as compared to all other members of the European clade, namely Avar, French, Indian, Loschbour, Sardinians, Stuttgart, and Tajik (Fig. 5A), except for Afanasievo, Andronovo, Mari, and Motala12.

Afanasievo and Andronovo (Allentoft et al. 2015) and Motala12 (Lazaridis et al. 2014) are known to have high levels of Mal'ta ancestry. As compared to Mal'ta, Kets were significantly closer to: Athabaskans, Greenlanders, Late Dorset, Mayans, Mixe, and especially Saqqaq (Z < -2.9, Fig. 5B). The dataset without transitions showed a similar picture, but with generally lower Z-scores (Suppl. Fig. 8.16). Overall, these results are consistent with topologies observed for Kets and Mal'ta in the TreeMix analysis on both dataset versions (Suppl. Information, Section 9): Kets formed a sister-clade for East Asians and Native Americans (as in the trees obtained by Raghavan et al. 2015), with Mal'ta located in the European clade (Fig. 3A). The proximity of Kets and Bronze Age Karasuk culture (see f_3 statistics in Suppl. Figs. 7.5 and 7.6 and a TreeMix tree in Suppl. Figs. 9.1) was supported by f_4 statistics f_4(Karasuk, Yoruba; Ket, X) (Suppl. Fig. 8.17A,B). Karasuk was significantly closer to Kets with the Z-score cut-off of 2.9, as compared to all populations in the dataset except for Aleutian, Greenlanders, Iron Age Russia, Mal'ta, Mayans, Mixe, and Saqqaq, and none of the non-significant scores were negative, which means that Kets was probably the closest population for Karasuk in that dataset.

Taking into account the tree topologies and migration edges discussed in Suppl. Information, Section 9, we can tentatively model Kets as a two-way mixture of Siberians (related to East Asians) and ancient North Eurasians (represented by the Mal'ta genome). The Loschbour and Mal'ta individuals form a clade relative to East Asians (Fig. 3A), which is supported by |Z| < 2 for most statistics f_4(East Asian, outgroup; Loschbour, Mal'ta) (Suppl. Table 6, Suppl. Figs. 8.7-8.9; see also Lazaridis et al. 2014). Hence the proportion of ancient North Eurasian ancestry in Kets can be calculated with the following f_4-ratios (Patterson et al. 2012):

$(i)\ \frac{f4(\text{Loschbour, San; Siberian population, East Asian population})}{f4(\text{Loschbour, San; Mal'ta, East Asian population})}$ on the genome-based dataset without transitions, 190K SNPs. Using this approach, the Mal'ta ancestry in Kets can be roughly estimated at 27% – 30%, i.e. the range of f_4-ratios given two East Asian populations, Han or Kinh. A smaller dataset of 105K SNPs, the genome-based dataset without transitions merged with genomes from Raghavan et al. (2015), demonstrated a higher proportion of Mal'ta ancestry in Kets (43%), a bit lower proportion in Altaians (41%), and much lower proportions in Buryats and Nivkhs (9% and 15%, respectively, see Suppl. Table 6). If we modelled Mal'ta ancestry in Athabaskans, Clovis, Karitiana, Mayans, and Mixe with the same approach (similar to that used by Lazaridis et al. 2014: $\frac{f4(\text{Loschbour, Stuttgart; Karitiana, Onge})}{f4(\text{Loschbour, Stuttgart; Mal'ta, Onge})}$), f_4-ratios ranged from 25% to 45% (Suppl. Table 6), consistent with previous estimates of 30 to 40% Mal'ta ancestry in various Native Americans (Lazaridis et al. 2014, Raghavan et al. 2014a). Dataset 'Ket genomes + Raghavan et al. 2015' demonstrated a similar result: f_4-ratios ranged from 31% to 53% for Athabaskans, Clovis, Huichol, Karitiana, Mayans, Mixe, and Tsimshian. In summary, the level of Mal'ta ancestry in Kets is similar to but not higher than that in Native Americans, and this conclusion is compatible with results obtained with f_3(Yoruba, Mal'ta, X) (Suppl. Figs. 7.13-7.16).

Kets and ancient populations of South Siberia

According to statistics f_4(Karasuk, Yoruba; Ket, X) on the genome-based dataset (Suppl. Fig. 8.17A), Kets are significantly closer to Karasuk (Z > 2.9) as compared to most populations in the dataset: Afanasievo, Aleutian, Andronovo, Athabaskan, Avar, Clovis, Dai, French, Greenlander, Han, Indian, Iron Age Altai, Iron Age Russia, Karitiana, Kinh, Late Dorset, Loschbour, Malta, Mari, Mayan, Mixe, Motala12, Nivkh, Saqqaq, Sardinian, Stuttgart, and Tajik. Kets are closer to Karasuk, but without statistically significant Z-scores (Z < 2.9), as compared to the following few populations: Aleutian, Greenlander, Iron Age Russia, Malta, Mayan, Mixe, Saqqaq. No population had a negative Z-score.

Kets and Saqqaq

Nganasans emerged as the top hit for Kets in dataset 'Ket genomes + HumanOrigins array' analyzed with statistic f_4(Ket, Chimp; Saqqaq, X) with a Z-score of -7.4, and Selkups, Chukchi, Koryaks, Itelmens, and Dolgans had significant Z-scores <-3.35 (Bonferroni correction for multiple testing given 126 independent tests corresponds to a threshold *p*-value of 0.0004). Statistic f_4(Saqqaq, Chimp; Ket, X) on dataset 'Ket genomes + HumanOrigins array' gave significantly negative Z-scores for the following populations: Tuvinian, Japanese, Korean, Daur, Yakut, Dolgan, Hezhen, Oroqen, Yukaghir, Ulchi, Nganasan, Itelmen, Koryak, Chukchi, Eskimo (populations are sorted in the order of decreasing Z-score, from -3.6 for Tuvinian to -13.9 for Eskimo). In the genome-based dataset lacking Nganasans and closely related populations, according to statistic f_4(Ket, Yoruba; Saqqaq, X) Kets were significantly closer to Saqqaq (threshold Z-score of 2.9), as compared to all other populations except for Athabaskans, Mayans, and Mixe (Fig. 5B). Late Dorset Paleo-Eskimo was not counted as the closest relative of Saqqaq. The same statistic on the dataset without transitions gave positive non-significant Z-scores < 2.9 for Athabaskans, Clovis, Greenlanders, Iron Age Russia, Mayans, and Mixe (Suppl. Fig. 8.16). Statistic f_4(Saqqaq, Yoruba; Ket, X) had significantly negative Z-scores of -4.3 and -12.8 only for Greenland Inuits and Late Dorset, respectively, with other negative scores >-2 on both versions of the genome-based dataset (Suppl. Fig. 8.18).

Let us consider a topology with high bootstrap support, where Beringian and Native American populations form a clade, Siberian populations represent its sister-clades, and Saqqaq receives genetic input from both Siberian and Beringian populations (see, for example, Suppl. Fig. 9.1). Hence the following f_4-ratios can be applied to estimate the percentage of Beringian ancestry in Saqqaq (on the genome-based dataset without transitions):

$$\alpha = \frac{f4(\text{Native American population, outgroup; Saqqaq, Ket})}{f4(\text{Native American population, outgroup; Greenlander, Ket})}$$

And the Siberian ancestry in Saqqaq equals 1 – α, ranging from 38% to 57% given various Native American populations: Athabaskans, Clovis, Karitiana, Mayans, and Mixe (Suppl. Table 6). A similar ratio on the dataset 'Ket genome + Raghavan et al. 2015' was calculated as follows:

$$\alpha = \frac{f4(\text{Mayan, outgroup; Saqqaq, Siberian population})}{f4(\text{Mayan, outgroup; Eskimo (Yupik), Siberian population})}$$

For various Siberian populations (Altaians, Buryats, Kets, Yakuts), $1 - \alpha$ ranges from 31% to 44% (Suppl. Table 6). This proportion of 'core Siberian' ancestry in Saqqaq is similar to that modelled with ADMIXTURE (Fig. 1A).

Kets and Na-Dene speakers

f_3 and f_4 statistics and TreeMix did not show any specific link between Kets and Athabaskans, Chipewyans, or Tlingit. Outgroup f_3 statistic f_3(Yoruba; Na-Dene-speaking population, X) produced a mixture of South and North American populations as top five hits on all five datasets analyzed (Suppl. Figs. 7.7-7.12). Statistics f_4(Ket, Chimp; Chipewyan, X) on dataset 'Ket genomes + HumanOrigins array' showed that Kets are significantly closer (threshold Z-score of -3.35) to a number of Siberian and Beringian populations, as compared to Chipewyans: Oroqen, Ulchi, Mansi, Yakuts, Dolgans, Itelmens, Yukaghirs, Eskimo, Koryaks, Chukchi, Selkups, Nganasans (populations are sorted in the order of decreasing Z-score, from -3.5 for Oroqen to -12.8 for Nganasans). According to statistic f_4(Ket, Yoruba; Athabaskan, X) calculated on the genome-based dataset, Kets were probably closer only to Saqqaq (however, with a Z-score of -2.7, below the significance threshold of 2.9), as compared to Athabaskans (Fig. 5B). Similar Z-scores were obtained for the genome-based dataset without transitions (-2.4, Suppl. Fig. 8.16).

Statistics f_4(Athabaskan, Yoruba; Ket, X) on the genome-based datasets and f_4(Chipewyan, Chimp; Ket884+891, X) on the dataset 'Ket genomes + HumanOrigins array' showed that these Na-Dene speakers shared significantly more drift ($Z < -2.9$ and <-3.35, respectively) with all First Americans, with all Beringian and some Siberian populations (e.g., Nganasans), as compared to Kets (Suppl. Fig. 8.19). For example, f_4(Athabaskan, Yoruba; Ket, X) produced highly negative Z-scores below -7.8 for Clovis, Greenland Inuits, Karitiana, Mayans, and Mixe; and a moderately negative score for Saqqaq ($Z = -1.9$) (Suppl. Fig. 8.19A). On the dataset without transitions Saqqaq demonstrated a similar Z-score of -2.1 (Suppl. Fig. 8.19B). Similarly, on the dataset 'Ket genomes + HumanOrigins array' statistics f_4(Chipewyan, Chimp; Ket, X) demonstrated highly negative Z-scores <-10.8 for all First Americans, and significant scores (threshold Z-score of -3.35) for the following East Eurasian populations (sorted in the order of decreasing Z-score, from -4.1 for Ulchi to -13.2 for Eskimo): Ulchi, Yukaghir, Nganasan, Itelmen, Koryak, Chukchi. TreeMix consistently placed Athabaskans as the sister-clade for First Americans, i.e. Clovis, Karitiana, Mayans, and Mixe, with very high bootstrap supports from 94 to 99 at *m* from 6 to 8 (Fig. 3A, Suppl. Figs. 9.1-9.5), in agreement with TreeMix results in Raghavan et al. (2014, 2015) and with the f_4 statistic results shown above. Overall, we can conclude that Siberian ancestry in Chipewyans and Athabaskans is more closely related to Nganasans rather than to Kets, and the same is true for Saqqaq.

According to a published admixture graph analysis, Chipewyans were modeled as a mixture of 84% First Americans and 16% Saqqaq (Reich et al. 2012). Given the topology suggested in our study, i.e.

Saqqaq forming a clade with certain Asian populations, rather than with First Americans (due to its 31% to 57% of Siberian ancestry, see above), we can estimate the percentage of Saqqaq ancestry in Chipewyans and Athabaskans on the genome-based dataset without transitions and on the HumanOrigins-based datasets. The topology 'Native American, (Saqqaq, Siberian)' was supported by low Z-scores (|Z| < 2) for a number of American-Siberian population combinations in the following f_4 statistic set-up: f_4(Clovis or Mayan, Yoruba or San; Siberian population, Saqqaq) (Suppl. Table 6). In the case of Athabaskans, included into the genome-based dataset, the f_4-ratios equaled:

$$\frac{f4(\text{Ket or Nivkh, San; Athabaskan, Clovis or Mayan})}{f4(\text{Ket or Nivkh, San; Saqqaq, Clovis or Mayan})}$$

The Saqqaq ancestry in Athabaskans estimated using this approach was close to 0, judging by the f_4-ratios ranging from -0.006 to 0.089. Similarly, the following f_4-ratios were used to estimate the Saqqaq ancestry in Chipewyans, included into datasets 'Ket genomes + HumanOrigins array' and 'Ket genomes + HumanOrigins array + Verdu et al. 2014':

$$\frac{f4(\text{or Tuvinian, Yoruba; Chipewyan, Mayan})}{f4(\text{or Tuvinian, Yoruba; Saqqaq, Mayan})}$$

Thus, the Saqqaq ancestry in Chipewyans can be estimated at 3.6-15.2% on dataset 'Ket genomes + HumanOrigins array' and at 6.8%-11.1% on dataset 'Ket genomes + HumanOrigins array + Verdu et al. 2014', roughly similar to the estimate of 16% published previously (Reich et al. 2012)

References

Allentoft, M. E. *et al*. Population genomics of Bronze Age Eurasia. Nature. **522,** 167–172 (2015).

Lazaridis, I. *et al.* Ancient human genomes suggest three ancestral populations for present-day Europeans. *Nature* **513,** 409–413 (2014).

Patterson, N. J. *et al.* Ancient admixture in human history. *Genetics* **192,** 1065–1093 (2012).

Raghavan, M. *et al.* Upper Palaeolithic Siberian genome reveals dual ancestry of Native Americans. *Nature* **505,** 87–91 (2014a).

Raghavan, M. *et al.* The genetic prehistory of the New World Arctic. *Science* **345,** 1255832 (2014b).

Raghavan, M. *et al.* Genomic evidence for the Pleistocene and recent population history of Native Americans. *Science* doi: 10.1126/science.aab3884 (2015).

Reich, D. *et al.* Reconstructing Native American population history. *Nature* **488,** 370–374 (2012).

Seguin-Orlando, A. *et al.* Genomic structure in Europeans dating back at least 36,200 years. *Science* **346,** 1113–1118 (2014).

Suppl. Table 6. Various f_4-ratios, calculated in most cases on datasets 'Ket genomes + reference genomes' (abbreviated as NGS1) and 'Ket genomes + Raghavan et al. 2015' (abbreviated as NGS2), both without transitions. Only 'C' populations with |Z| < 2 for f_4(C,O;A,B) are shown.

	f_4-ratios	f_4(Loschbour, outgroup; Siberian, East Asian) / f_4(Loschbour, outgroup; Mal'ta, East Asian)				=	f_4(A, O; X, C) / f_4(A, O; B, C)			
dataset	A	B ->	X	<- C	O	f_4 (X)	f_4 (B)	f_4-ratio	average f_4-ratio for X	Z(C,O;A,B)
NGS1 / no transitions	Loschbour	Mal'ta	Ket	Han	San	0.0024	0.0092	0.266	0.283	-0.24
NGS1 / no transitions	Loschbour	Mal'ta	Ket	Kinh	San	0.0029	0.0096	0.301		-1.25
NGS2 / no transitions	Loschbour	Mal'ta	Altaian	Han	San	0.0029	0.0071	0.406	0.405	0.70
NGS2 / no transitions	Loschbour	Mal'ta	Altaian	Kinh	San	0.0029	0.0071	0.405		-0.44
NGS2 / no transitions	Loschbour	Mal'ta	Buryat	Han	San	0.0007	0.0071	0.092	0.091	0.70
NGS2 / no transitions	Loschbour	Mal'ta	Buryat	Kinh	San	0.0006	0.0071	0.090		-0.44
NGS2 / no transitions	Loschbour	Mal'ta	Ket	Han	San	0.0030	0.0071	0.427	0.426	0.70
NGS2 / no transitions	Loschbour	Mal'ta	Ket	Kinh	San	0.0030	0.0071	0.425		-0.44
NGS2 / no transitions	Loschbour	Mal'ta	Nivkh	Han	San	0.0011	0.0071	0.150	0.149	0.70
NGS2 / no transitions	Loschbour	Mal'ta	Nivkh	Kinh	San	0.0010	0.0071	0.147		-0.44
NGS2 / no transitions	Loschbour	Mal'ta	Yakut	Han	San	-0.0007	0.0071	-0.098	-0.100	0.70
NGS2 / no transitions	Loschbour	Mal'ta	Yakut	Kinh	San	-0.0007	0.0071	-0.101		-0.44
		f_4(Loschbour, outgroup; Native American, East Asian) / f_4(Loschbour, outgroup; Mal'ta, East Asian)					=	f_4(A, O; X, C) / f_4(A, O; B, C)		
NGS1 / no transitions	Loschbour	Mal'ta	Athabaskan	Han	San	0.0025	0.0092	0.272	0.289	-0.24
NGS1 / no transitions	Loschbour	Mal'ta	Athabaskan	Kinh	San	0.0030	0.0096	0.307		-1.25
NGS1 / no transitions	Loschbour	Mal'ta	Clovis	Han	San	0.0023	0.0092	0.254	0.272	-0.24
NGS1 / no transitions	Loschbour	Mal'ta	Clovis	Kinh	San	0.0028	0.0096	0.290		-1.25
NGS1 / no transitions	Loschbour	Mal'ta	Karitiana	Han	San	0.0026	0.0092	0.280	0.298	-0.24
NGS1 / no transitions	Loschbour	Mal'ta	Karitiana	Kinh	San	0.0030	0.0096	0.315		-1.25
NGS1 / no transitions	Loschbour	Mal'ta	Mayan	Han	San	0.0039	0.0092	0.420	0.434	-0.24
NGS1 / no transitions	Loschbour	Mal'ta	Mayan	Kinh	San	0.0043	0.0096	0.448		-1.25
NGS1 / no transitions	Loschbour	Mal'ta	Mixe	Han	San	0.0034	0.0092	0.367	0.382	-0.24
NGS1 / no transitions	Loschbour	Mal'ta	Mixe	Kinh	San	0.0038	0.0096	0.398		-1.25
NGS2 / no transitions	Loschbour	Mal'ta	Athabaskan	Han	San	0.0028	0.0088	0.324	0.325	0.70
NGS2 / no transitions	Loschbour	Mal'ta	Athabaskan	Kinh	San	0.0029	0.0088	0.325		-0.44
NGS2 / no transitions	Loschbour	Mal'ta	Clovis	Han	San	0.0030	0.0088	0.343	0.343	0.70
NGS2 / no transitions	Loschbour	Mal'ta	Clovis	Kinh	San	0.0030	0.0088	0.344		-0.44
NGS2 / no transitions	Loschbour	Mal'ta	Huichol	Han	San	0.0027	0.0088	0.312	0.313	0.70
NGS2 / no transitions	Loschbour	Mal'ta	Huichol	Kinh	San	0.0028	0.0088	0.314		-0.44
NGS2 / no transitions	Loschbour	Mal'ta	Karitiana	Han	San	0.0047	0.0088	0.532	0.533	0.70
NGS2 / no transitions	Loschbour	Mal'ta	Karitiana	Kinh	San	0.0047	0.0088	0.534		-0.44

NGS2 / no transitions	Loschbour	Mal'ta	Mayan	Han	San	0.0030	0.0088	0.341	0.342	0.70
NGS2 / no transitions	Loschbour	Mal'ta	Mayan	Kinh	San	0.0030	0.0088	0.343		-0.44
NGS2 / no transitions	Loschbour	Mal'ta	Mixe	Han	San	0.0028	0.0088	0.314	0.315	0.70
NGS2 / no transitions	Loschbour	Mal'ta	Mixe	Kinh	San	0.0028	0.0088	0.316		-0.44
NGS2 / no transitions	Loschbour	Mal'ta	Tsimshian	Han	San	0.0038	0.0088	0.436	0.437	0.70
NGS2 / no transitions	Loschbour	Mal'ta	Tsimshian	Kinh	San	0.0039	0.0088	0.438		-0.44
	1 –	f_4(Native American, outgroup; Saqqaq, Siberian)					= 1 –	f_4(A, O; X, C)		
		f_4(Native American, outgroup; Beringian, Siberian)						f_4(A, O; B, C)		
NGS1 / no transitions	Athabaskan	Greenlander	Saqqaq	Ket	San	0.0059	0.0111	0.469	0.474	-0.47
NGS1 / no transitions	Clovis	Greenlander	Saqqaq	Ket	San	0.0042	0.0068	0.379		-0.66
NGS1 / no transitions	Karitiana	Greenlander	Saqqaq	Ket	San	0.0051	0.0102	0.498		-0.20
NGS1 / no transitions	Mayan	Greenlander	Saqqaq	Ket	San	0.0050	0.0090	0.452		-0.35
NGS1 / no transitions	Mixe	Greenlander	Saqqaq	Ket	San	0.0042	0.0099	0.571		0.65
NGS2 / no transitions	Mayan	Eskimo (Yupik)	Saqqaq	Altaian	San	0.0076	0.0111	0.312	0.370	-0.71
NGS2 / no transitions	Mayan	Eskimo (Yupik)	Saqqaq	Buryat	San	0.0058	0.0092	0.375		-1.90
NGS2 / no transitions	Mayan	Eskimo (Yupik)	Saqqaq	Ket	San	0.0045	0.0079	0.435		-0.67
NGS2 / no transitions	Mayan	Eskimo (Yupik)	Saqqaq	Yakut	San	0.0061	0.0096	0.360		-1.52
		f_4(Siberian, outgroup; Na-Dene, First wave Native Americans)						=	f_4(A, O; X, C)	
		f_4(Siberian, outgroup; Saqqaq, First wave Native Americans)							f_4(A, O; B, C)	
NGS1 / no transitions	Ket	Saqqaq	Athabaskan	Clovis	San	0.0005	0.0061	0.089	0.038	-1.82
NGS1 / no transitions	Nivkh	Saqqaq	Athabaskan	Clovis	San	0.0002	0.0053	0.031		-0.51
NGS1 / no transitions	Nivkh	Saqqaq	Athabaskan	Mayan	San	0.0000	0.0051	-0.006		-1.00
Ket genomes + HumanOrigins array	Dolgan	Saqqaq	Chipewyan	Mayan	Yoruba	0.0003	0.0020	0.152	0.092	1.33
Ket genomes + HumanOrigins array	Tuvinian	Saqqaq	Chipewyan	Mayan	Yoruba	0.0000	0.0014	0.036		1.02
Ket genomes + HumanOrigins array + Verdu et al. 2014	Dolgan	Saqqaq	Chipewyan	Mayan	Yoruba	0.0003	0.0028	0.111		-0.40
Ket genomes + HumanOrigins array + Verdu et al. 2014	Tuvinian	Saqqaq	Chipewyan	Mayan	Yoruba	0.0002	0.0027	0.068		-1.11

8.1. Statistics f_4(X, Chimp; Mal'ta, Stuttgart) computed on the dataset 'Ket genomes + HumanOrigins array'. Top positive f_4 values (green circles) sorted in descending order with their standard errors (single SE interval) shown by vertical lines and Z-scores shown above. |Z| > 3.35 demonstrates that the statistic is significantly different from zero using Bonferroni correction for 126 independent tests (threshold p-value of 0.0004). Populations of America/Chukotka/Kamchatka and Eurasia are underlined by solid red and blue lines, respectively.

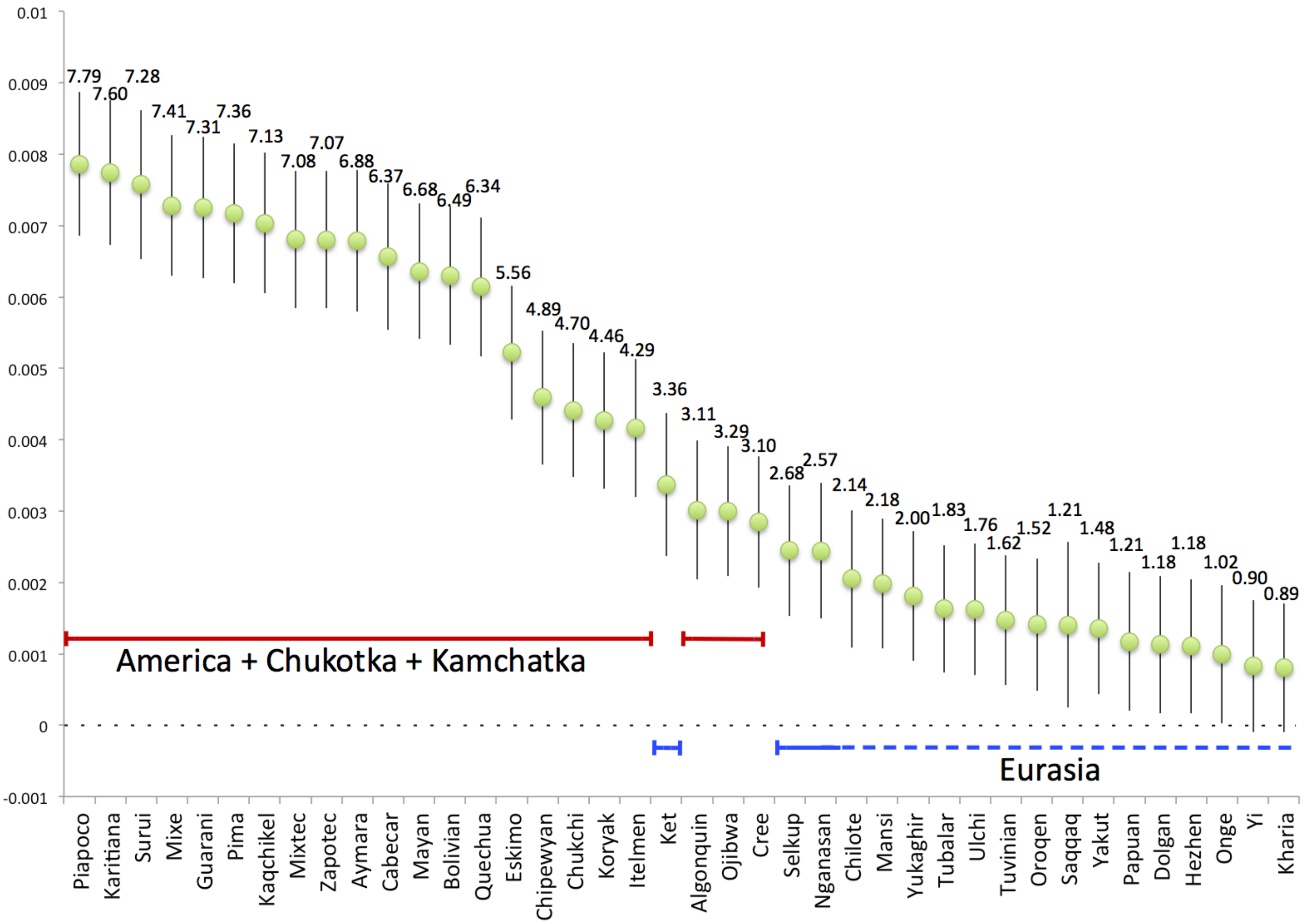

8.2. Statistics f_4(X, Yoruba; Mal'ta, Stuttgart) computed on the genome-based dataset. All f_4 values (green circles) are sorted in descending order with their standard errors (single SE interval) shown by vertical lines and Z-scores shown above. |Z| > 3.0 demonstrates that the statistic is significantly different from zero using Bonferroni correction for 33 independent tests (threshold p-value of 0.0015).

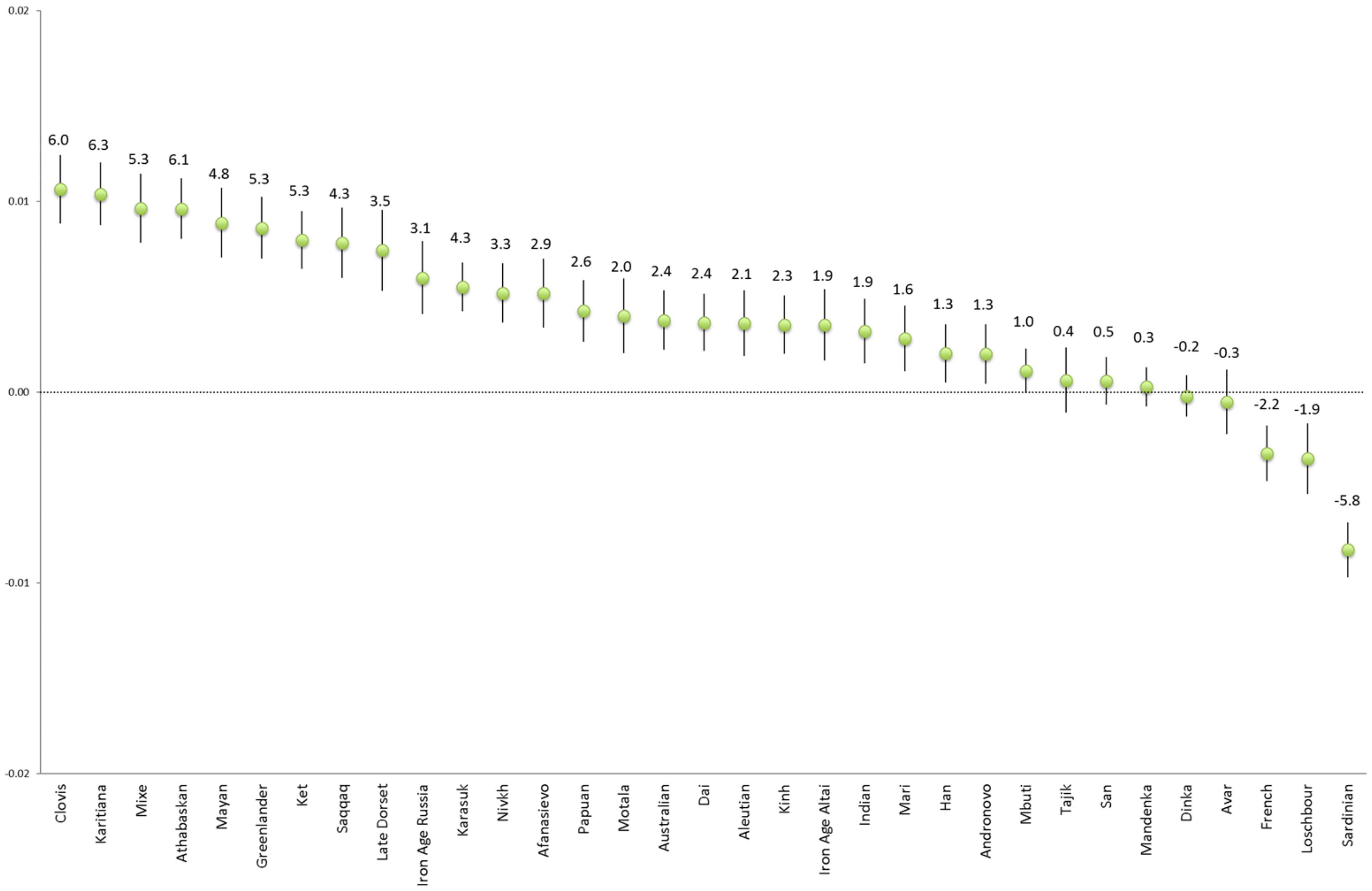

8.3. Statistics f_4(X, Yoruba; Mal'ta, Stuttgart) computed on the genome-based dataset without transitions. All f_4 values (green circles) are sorted in descending order with their standard errors (single SE interval) shown by vertical lines and Z-scores shown above. |Z| > 3.0 demonstrates that the statistic is significantly different from zero using Bonferroni correction for 33 independent tests (threshold p-value of 0.0015).

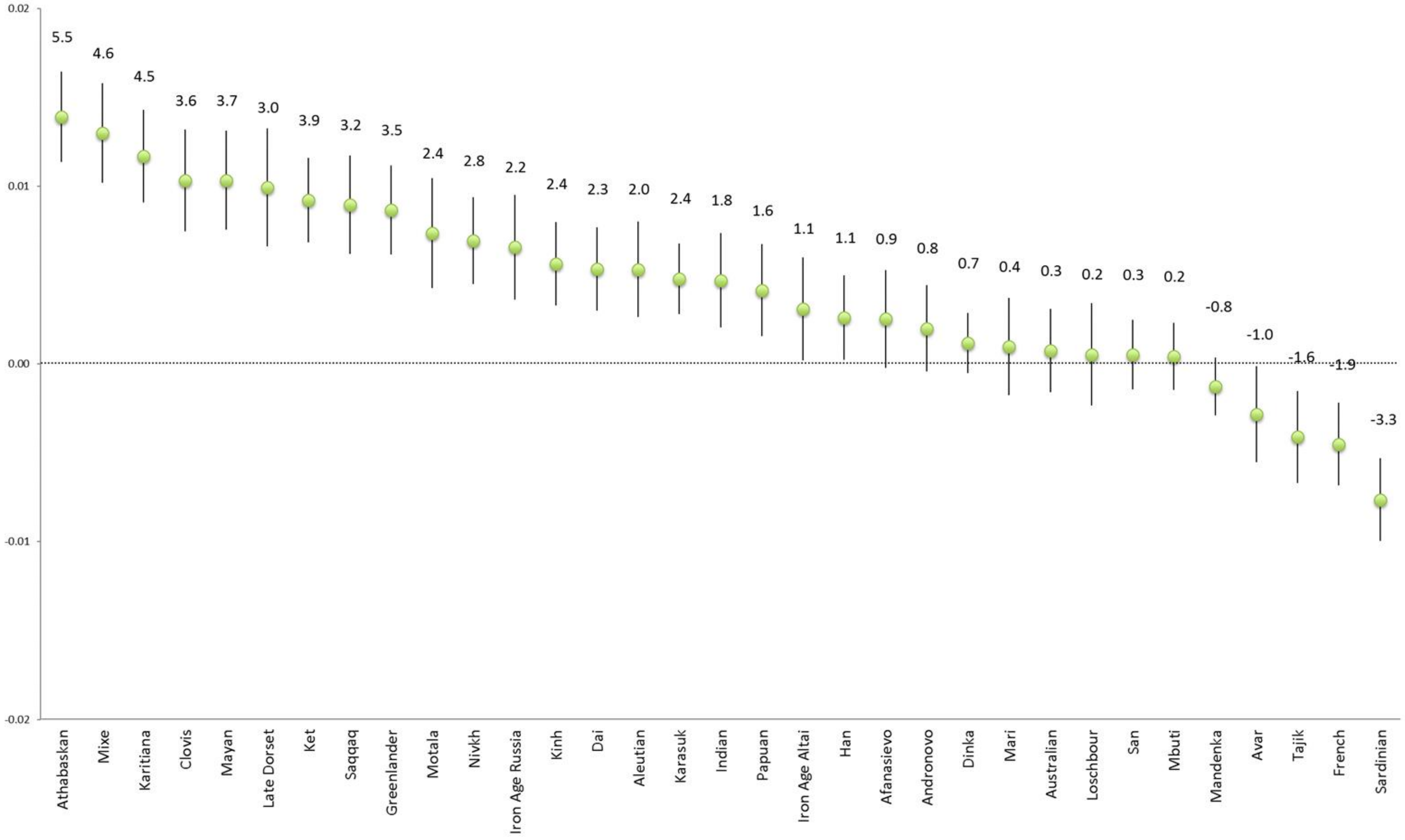

8.4. Statistics f_4(X, Papuan; Sardinian, Mal'ta) computed on the dataset 'Ket genomes + HumanOrigins array'. Top negative f_4 values (green circles) are sorted in descending order with their standard errors (single SE interval) shown by vertical lines and Z-scores shown above. |Z| > 3.35 demonstrates that the statistic is significantly different from zero using Bonferroni correction for 126 independent tests (threshold p-value of 0.0004).

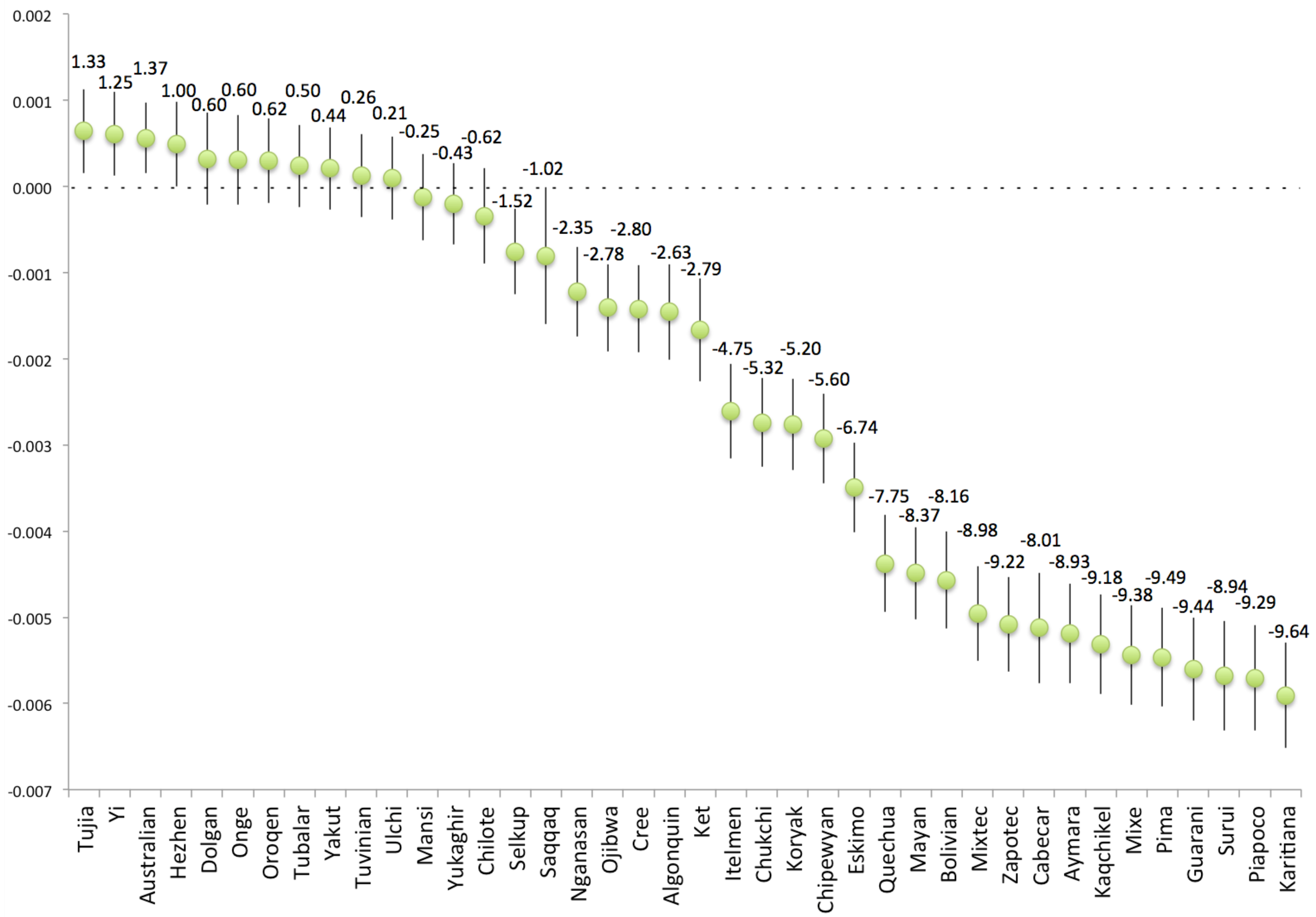

8.5. Statistics f_4(X, Papuan; Sardinian, Mal'ta) computed on the genome-based dataset. All f_4 values (green circles) are sorted in descending order with their standard errors (single SE interval) shown by vertical lines and Z-scores shown above. |Z| > 3.0 demonstrates that the statistic is significantly different from zero using Bonferroni correction for 33 independent tests (threshold *p*-value of 0.0015).

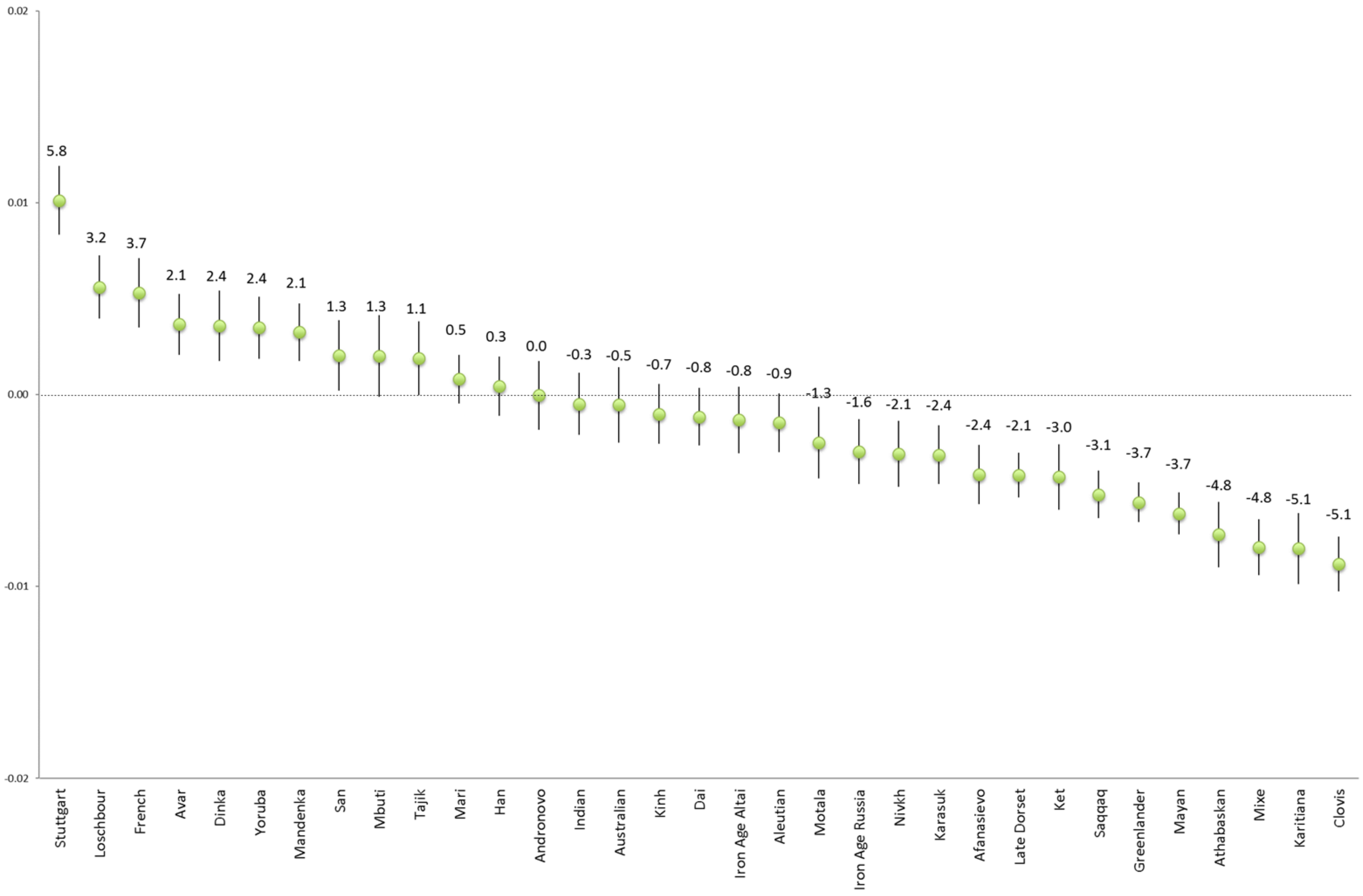

8.6. Statistics f_4(X, Papuan; Sardinian, Mal'ta) computed on the genome-based dataset without transitions. All f_4 values (green circles) are sorted in descending order with their standard errors (single SE interval) shown by vertical lines and Z-scores shown above. |Z| > 3.0 demonstrates that the statistic is significantly different from zero using Bonferroni correction for 33 independent tests (threshold p-value of 0.0015).

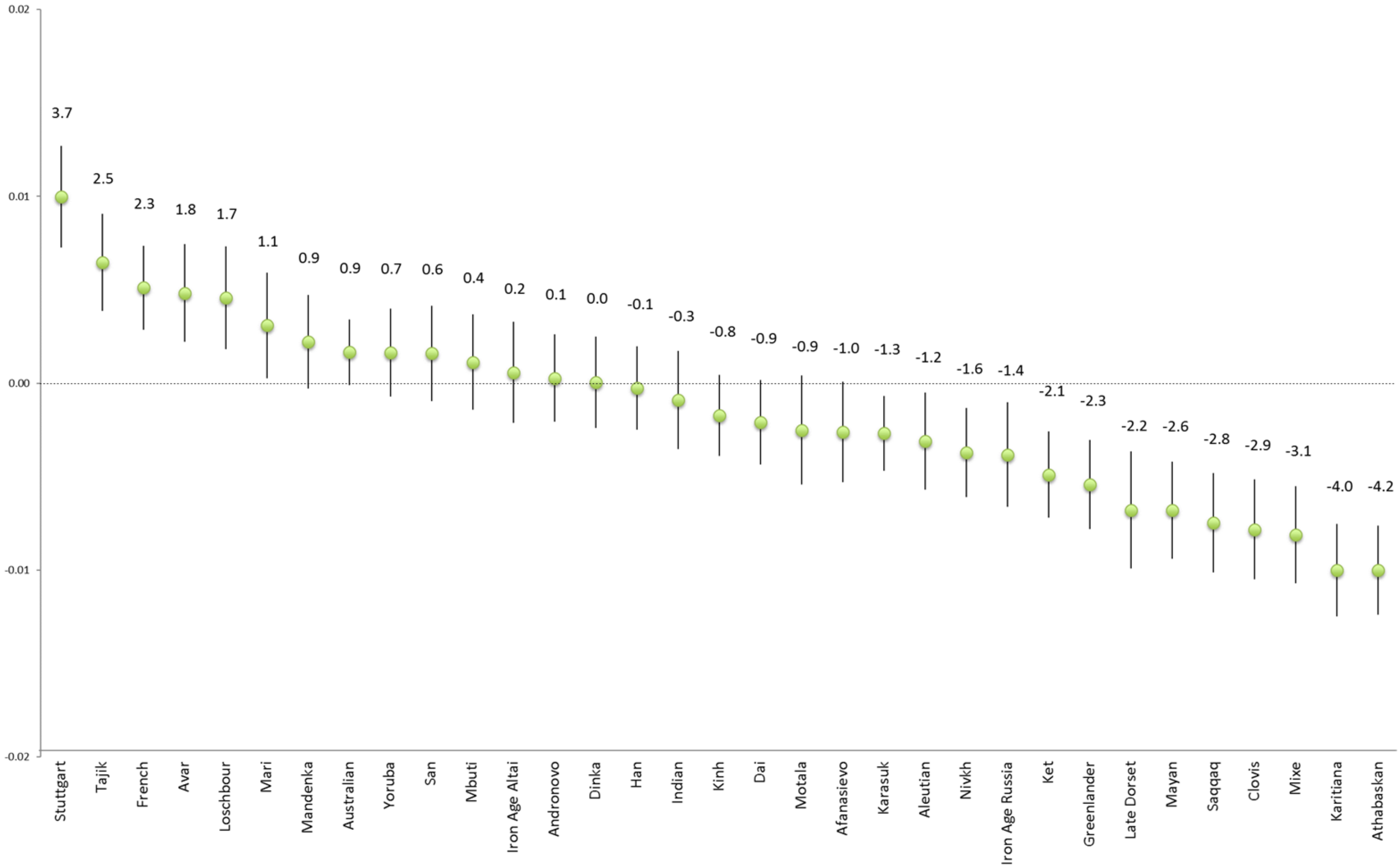

8.7. Statistics f_4(X, Chimp; Mal'ta, Loschbour) computed on the dataset 'Ket genomes + HumanOrigins array'. Top positive f_4 values (green circles) are sorted in descending order with their standard errors (single SE interval) shown by vertical lines and Z-scores shown above. |Z| > 3.35 demonstrates that the statistic is significantly different from zero using Bonferroni correction for 126 independent tests (threshold p-value of 0.0004).

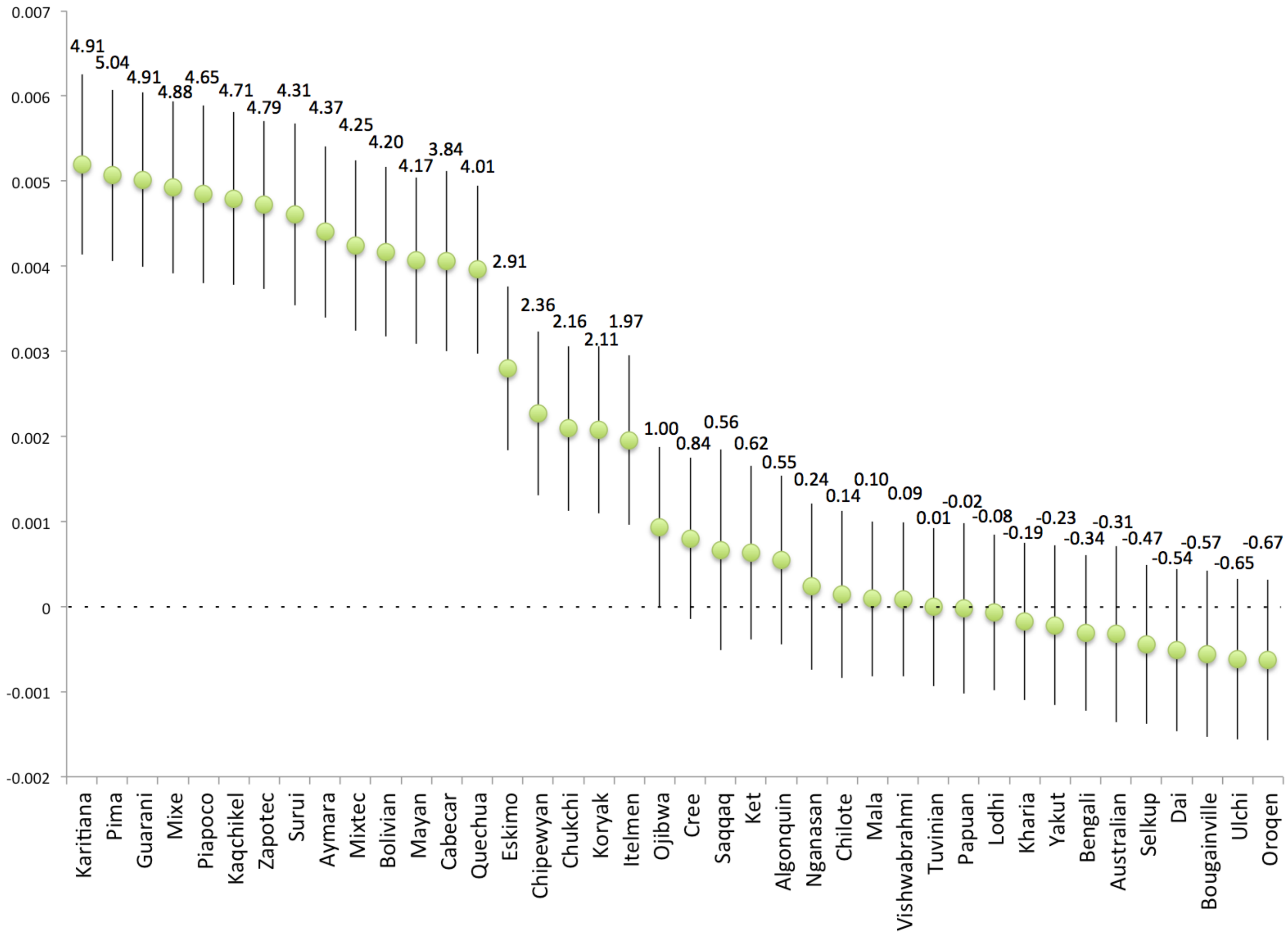

8.8. Statistics f_4(X, Yoruba; Mal'ta, Loschbour) computed on the genome-based dataset. All f_4 values (green circles) are sorted in descending order with their standard errors (single SE interval) shown by vertical lines and Z-scores shown above. |Z| > 3.0 demonstrates that the statistic is significantly different from zero using Bonferroni correction for 33 independent tests (threshold p-value of 0.0015).

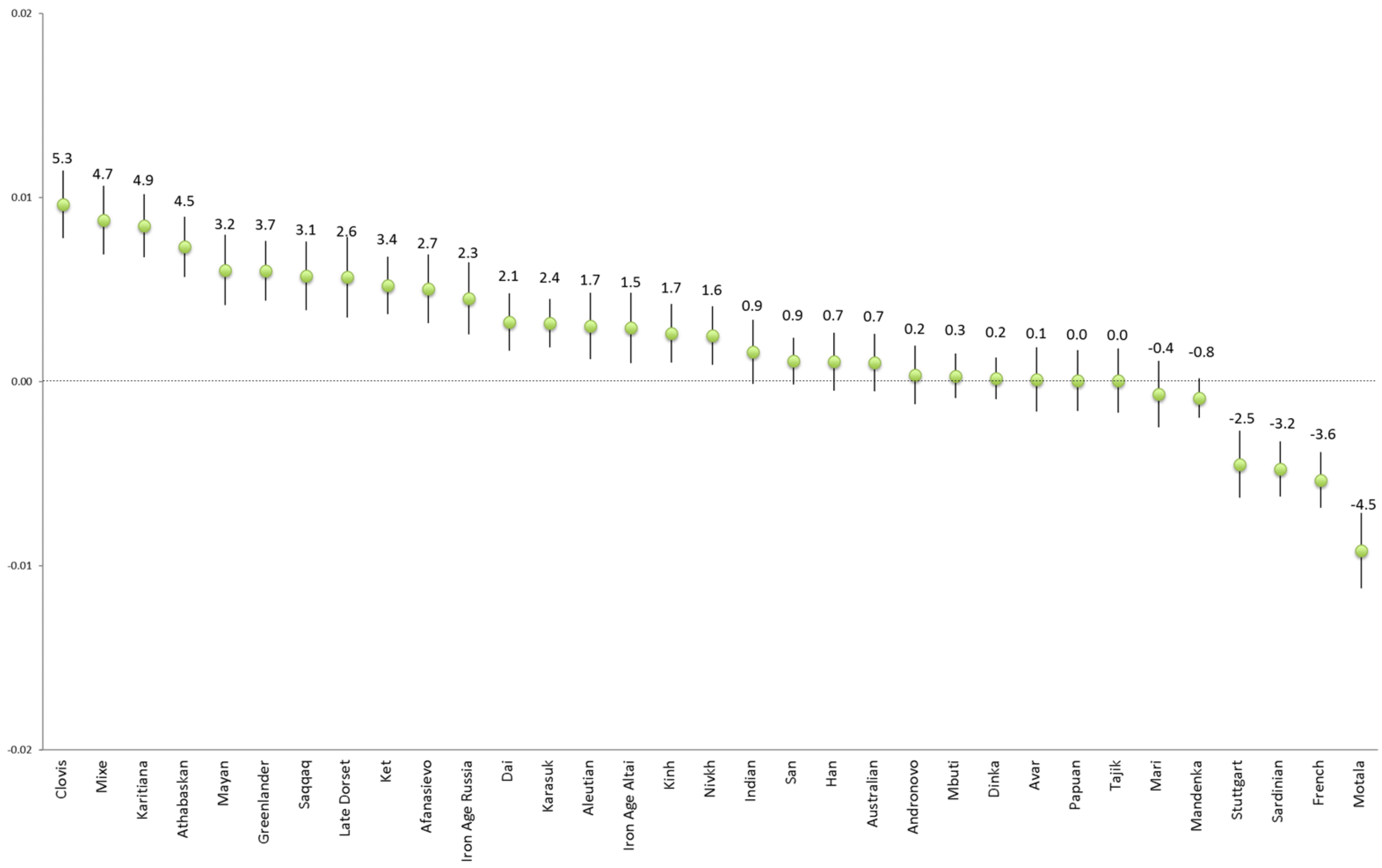

8.9. Statistics f_4(X, Yoruba; Mal'ta, Loschbour) computed on the genome-based dataset without transitions. All f_4 values (green circles) are sorted in descending order with their standard errors (single SE interval) shown by vertical lines and Z-scores shown above. |Z| > 3.0 demonstrates that the statistic is significantly different from zero using Bonferroni correction for 33 independent tests (threshold p-value of 0.0015).

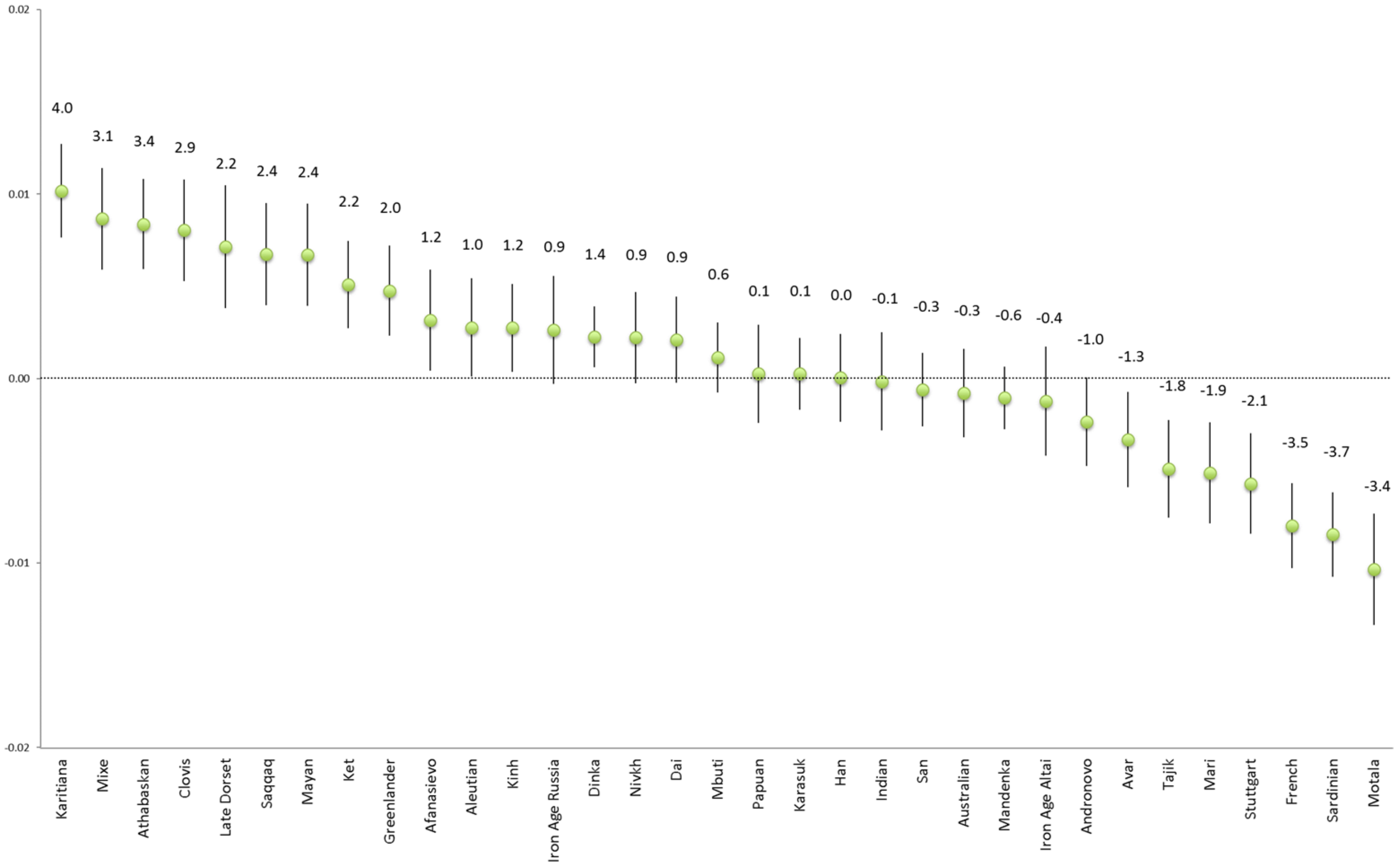

8.10. Statistics f_4(X, Chimp; Loschbour, Stuttgart) computed on the dataset 'Ket genomes + HumanOrigins array'. Top positive f_4 values (green circles) are sorted in descending order with their standard errors (single SE interval) shown by vertical lines and Z-scores shown above. |Z| > 3.35 demonstrates that the statistic is significantly different from zero using Bonferroni correction for 126 independent tests (threshold p-value of 0.0004).

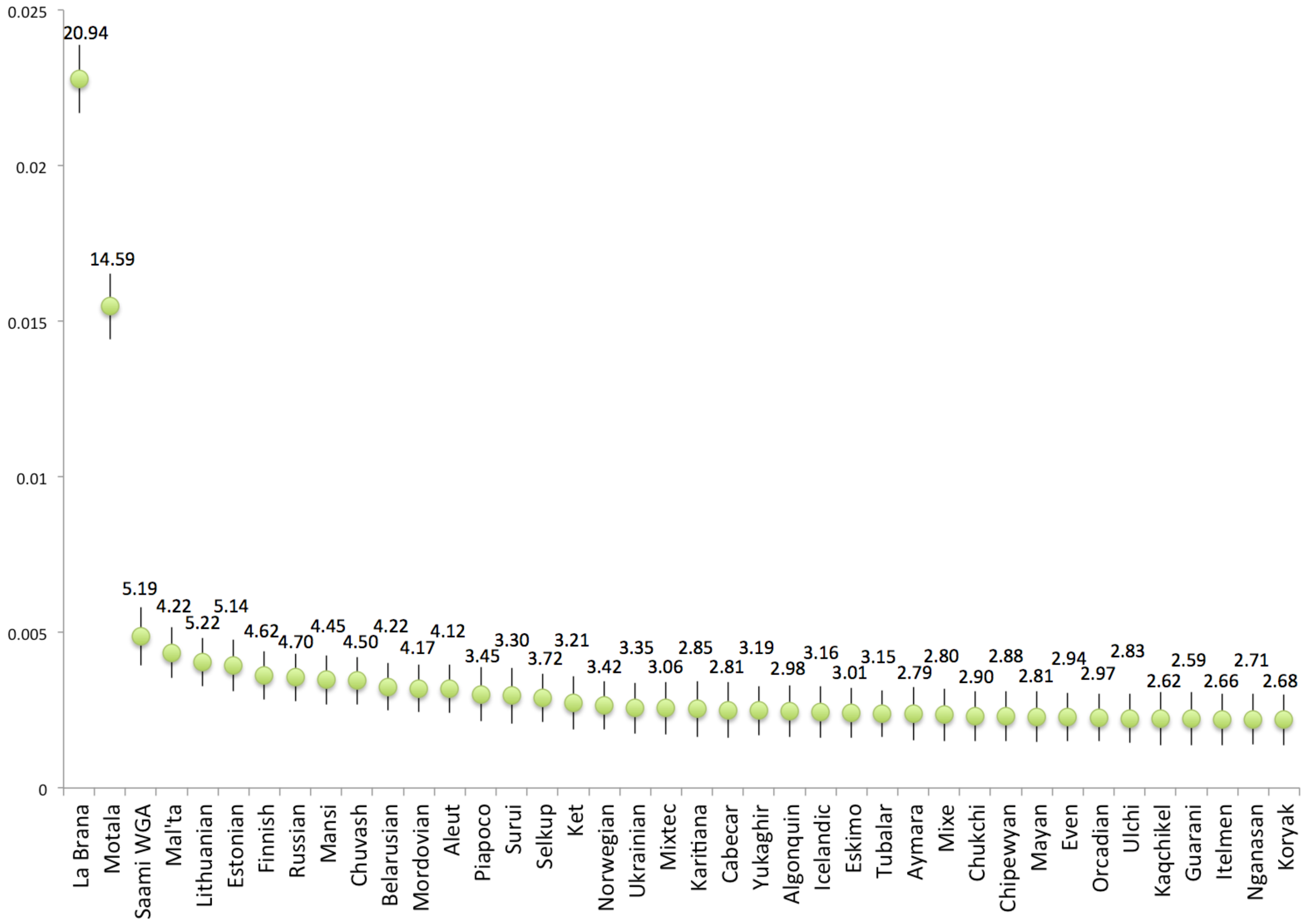

8.11. Statistics f_4(X, Yoruba; Loschbour, Stuttgart) computed on the genome-based dataset. All f_4 values (green circles) are sorted in descending order with their standard errors (single SE interval) shown by vertical lines and Z-scores shown above. |Z| > 3.0 demonstrates that the statistic is significantly different from zero using Bonferroni correction for 33 independent tests (threshold p-value of 0.0015).

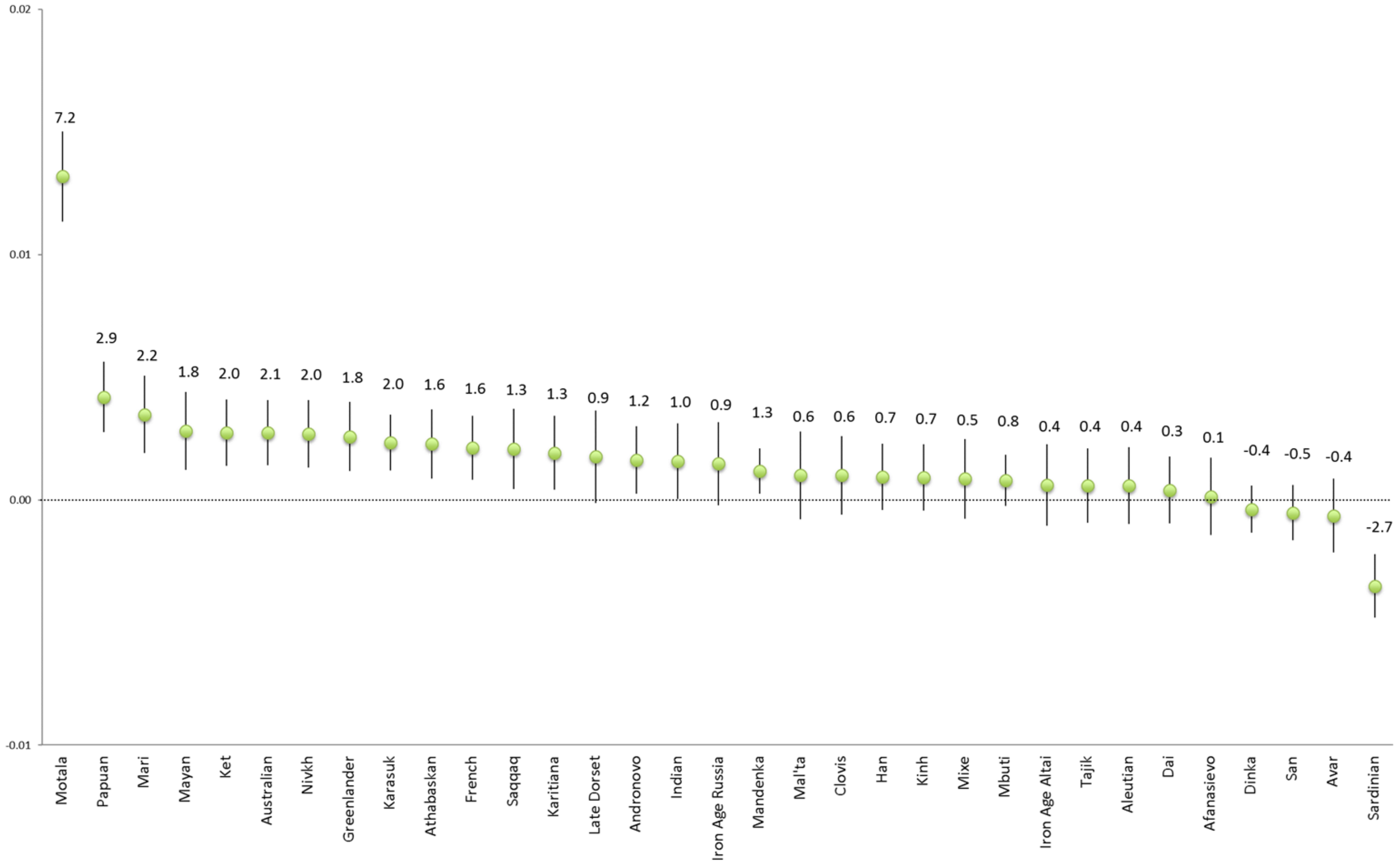

8.12. Statistics f_4(X, Yoruba; Loschbour, Stuttgart) computed on the genome-based dataset without transitions. All f_4 values (green circles) are sorted in descending order with their standard errors (single SE interval) shown by vertical lines and Z-scores shown above. |Z| > 3.0 demonstrates that the statistic is significantly different from zero using Bonferroni correction for 33 independent tests (threshold p-value of 0.0015).

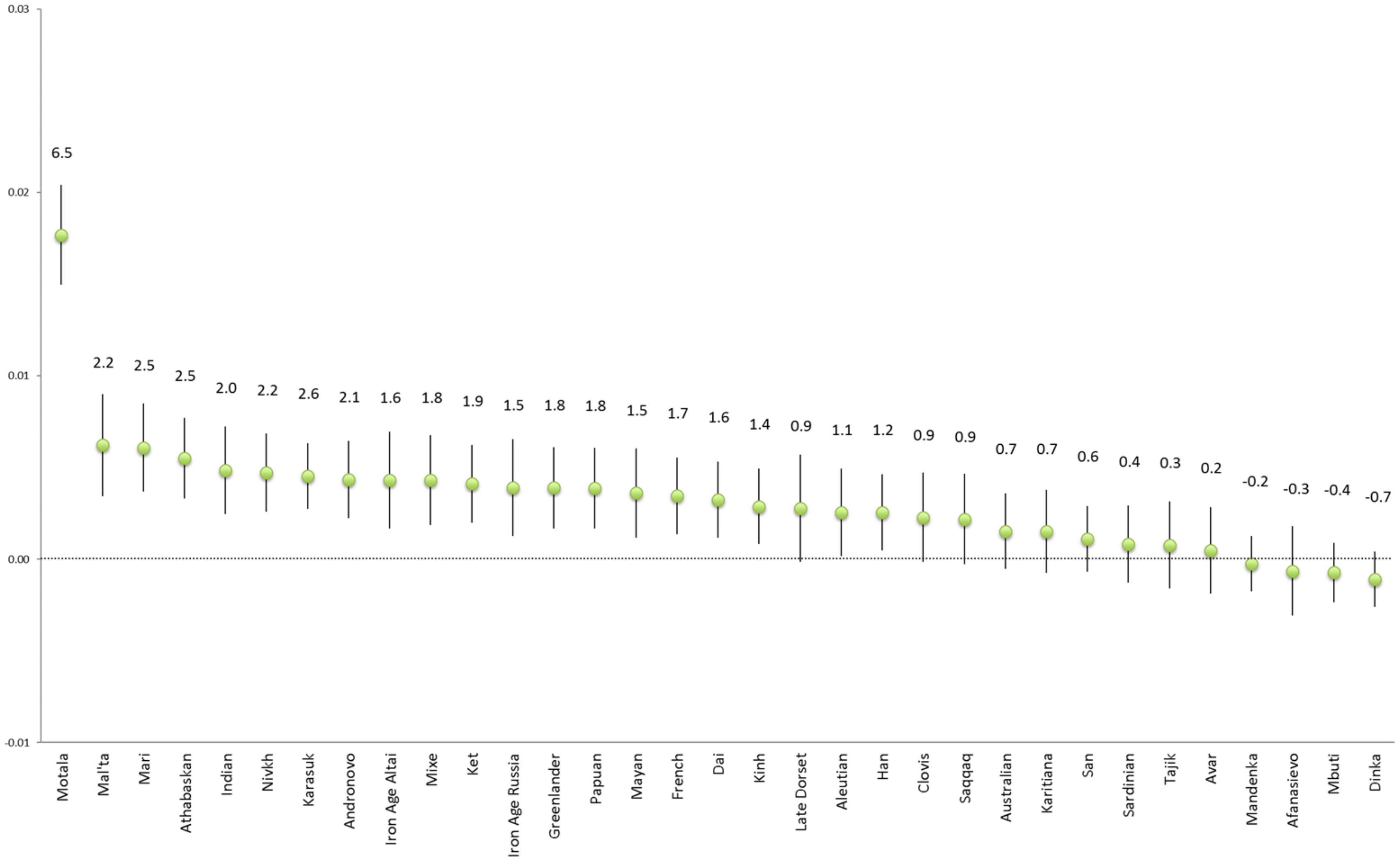

8.13. Statistics f_4(Haida, Chimp; Ket, X) computed on the dataset 'Ket genomes + HumanOrigins array + Verdu et al. 2014'. Top negative f_4 values (green circles) are sorted in descending order with their standard errors (single SE interval) shown by vertical lines and Z-scores shown above. |Z| > 3.37 demonstrates that the statistic is significantly different from zero using Bonferroni correction for 132 independent tests (threshold p-value of 0.00038).

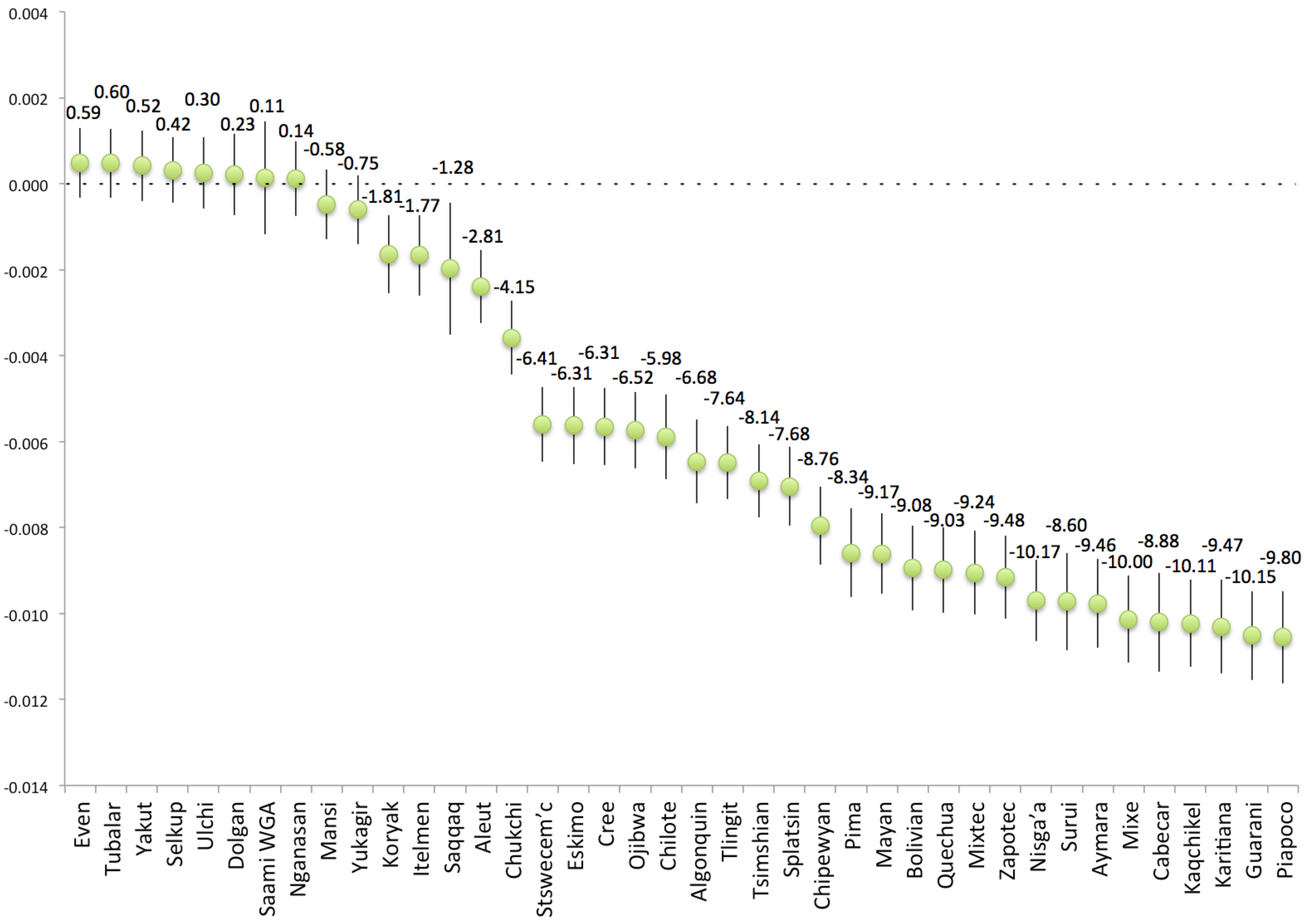

8.14. Statistics f_4(Ket, Chimp; Haida, X) computed on the dataset 'Ket genomes + HumanOrigins array + Verdu et al. 2014'. Top negative f_4 values (green circles) are sorted in descending order with their standard errors (single SE interval) shown by vertical lines and Z-scores shown above. |Z| > 3.37 demonstrates that the statistic is significantly different from zero using Bonferroni correction for 132 independent tests (threshold p-value of 0.00038).

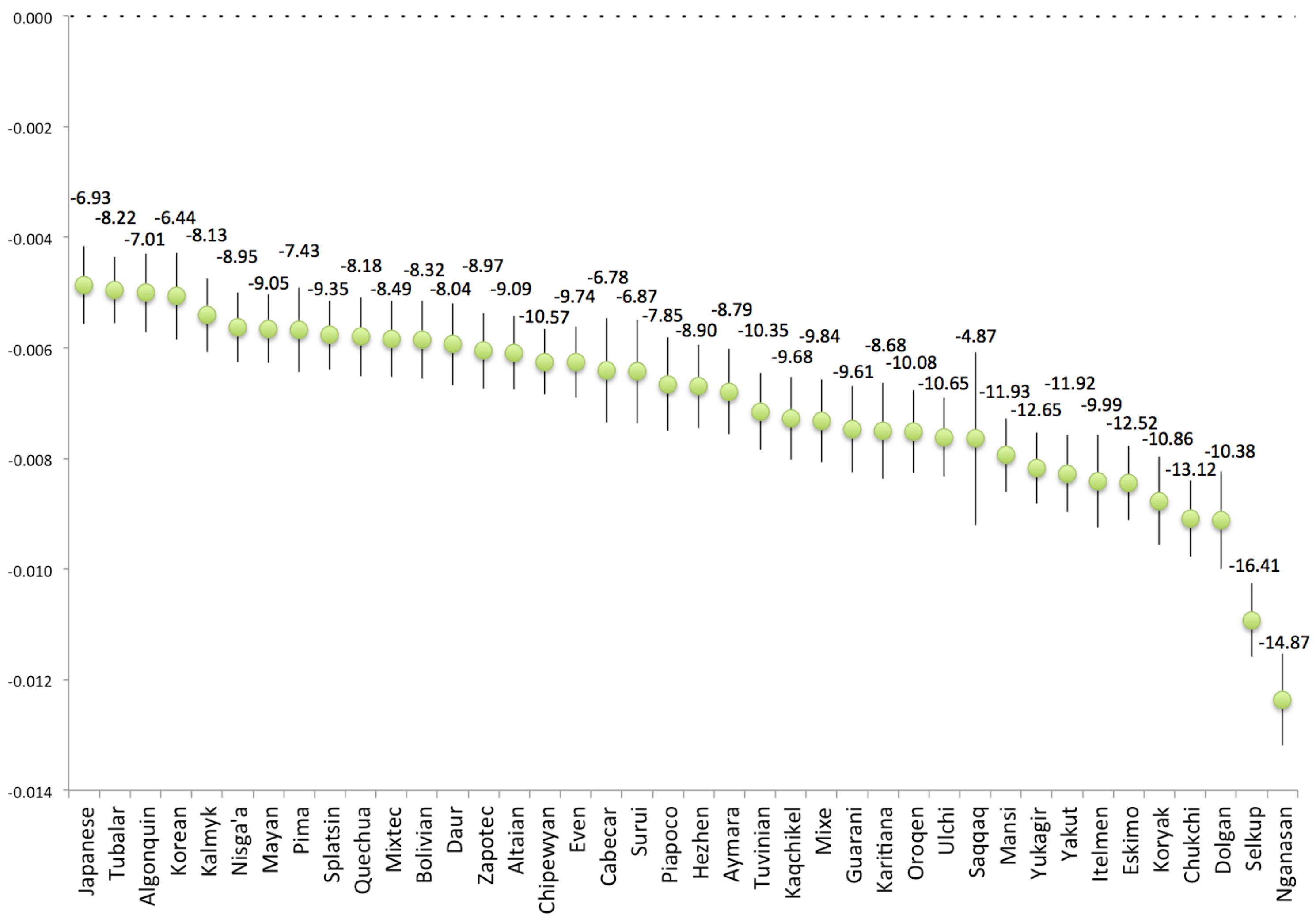

8.15. Statistics f_4(Mal'ta, Yoruba; Y, X) computed on the genome-based dataset without transitions, with African, Australian and Papuan populations removed. A matrix of color-coded Z-scores is shown, ancient genomes are marked with asterisks. Z-score equals the number of standard errors by which the statistic differs from zero, and |Z| > 2.9 demonstrates that the statistic is significantly different from zero using Bonferroni correction for 27 independent tests (threshold *p*-value of 0.001852). Rows show Z-scores for f_4(Mal'ta, Yoruba; row, column), *vice versa* for columns.

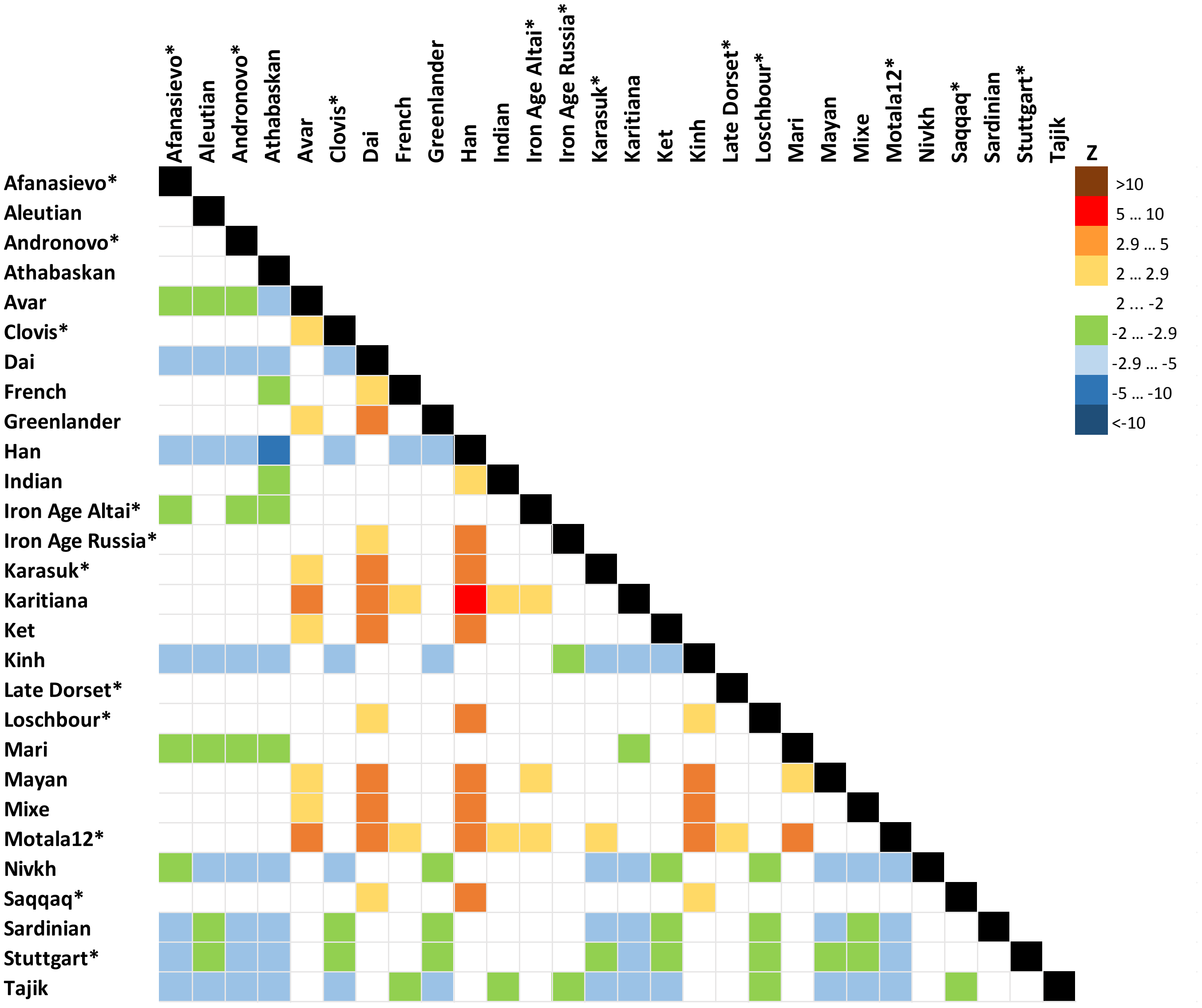

8.16. Statistics f_4(Ket, Yoruba; Y, X) computed on the genome-based dataset without transitions, with African, Australian and Papuan populations removed. A matrix of color-coded Z-scores is shown, ancient genomes are marked with asterisks. Z-score equals the number of standard errors by which the statistic differs from zero, and |Z| > 2.9 demonstrates that the statistic is significantly different from zero using Bonferroni correction for 27 independent tests (threshold *p*-value of 0.001852). Rows show Z-scores for f_4(Ket, Yoruba; row, column), *vice versa* for columns.

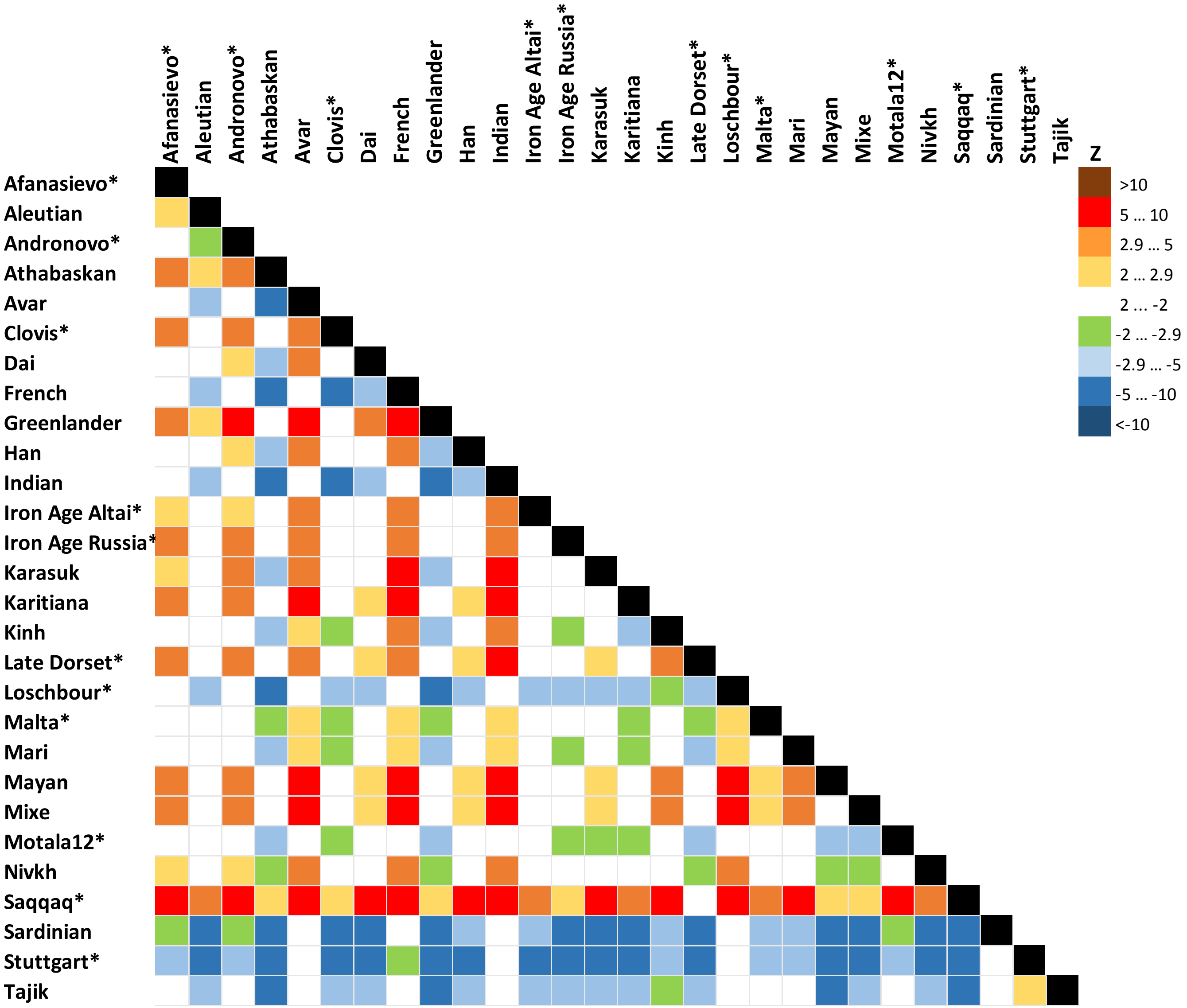

8.17. Statistics f_4(Karasuk, Yoruba; Y, X) computed on the genome-based dataset with African, Australian and Papuan populations removed, on the original version (**A**) and on the dataset with transitions excluded (**B**). A matrix of color-coded Z-scores is shown, ancient genomes are marked with asterisks. Z-score equals the number of standard errors by which the statistic differs from zero, and |Z| > 2.9 demonstrates that the statistic is significantly different from zero using Bonferroni correction for 27 independent tests (threshold *p*-value of 0.001852). Rows show Z-scores for f_4(Karasuk, Yoruba; row, column), *vice versa* for columns. **A**

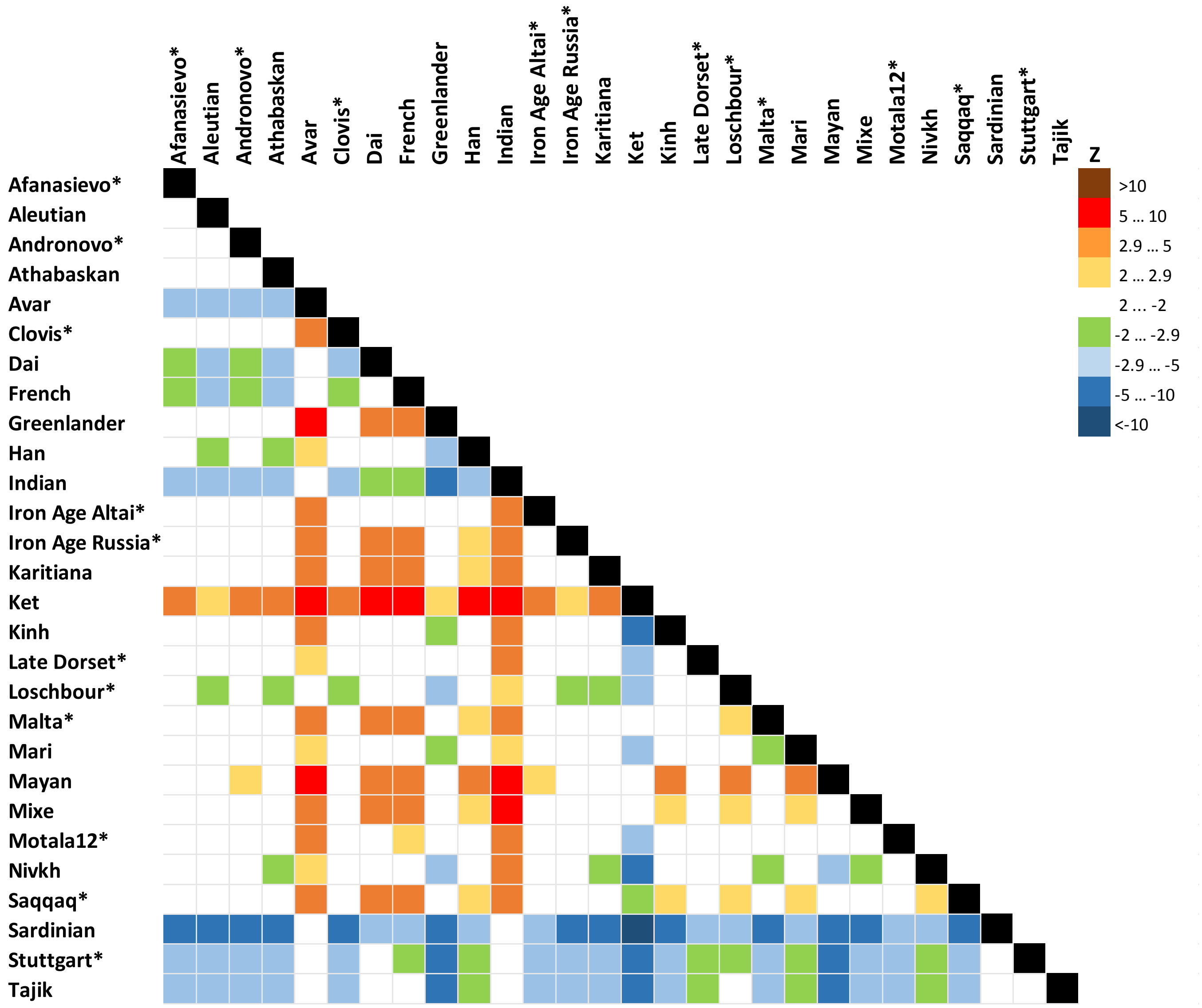

B

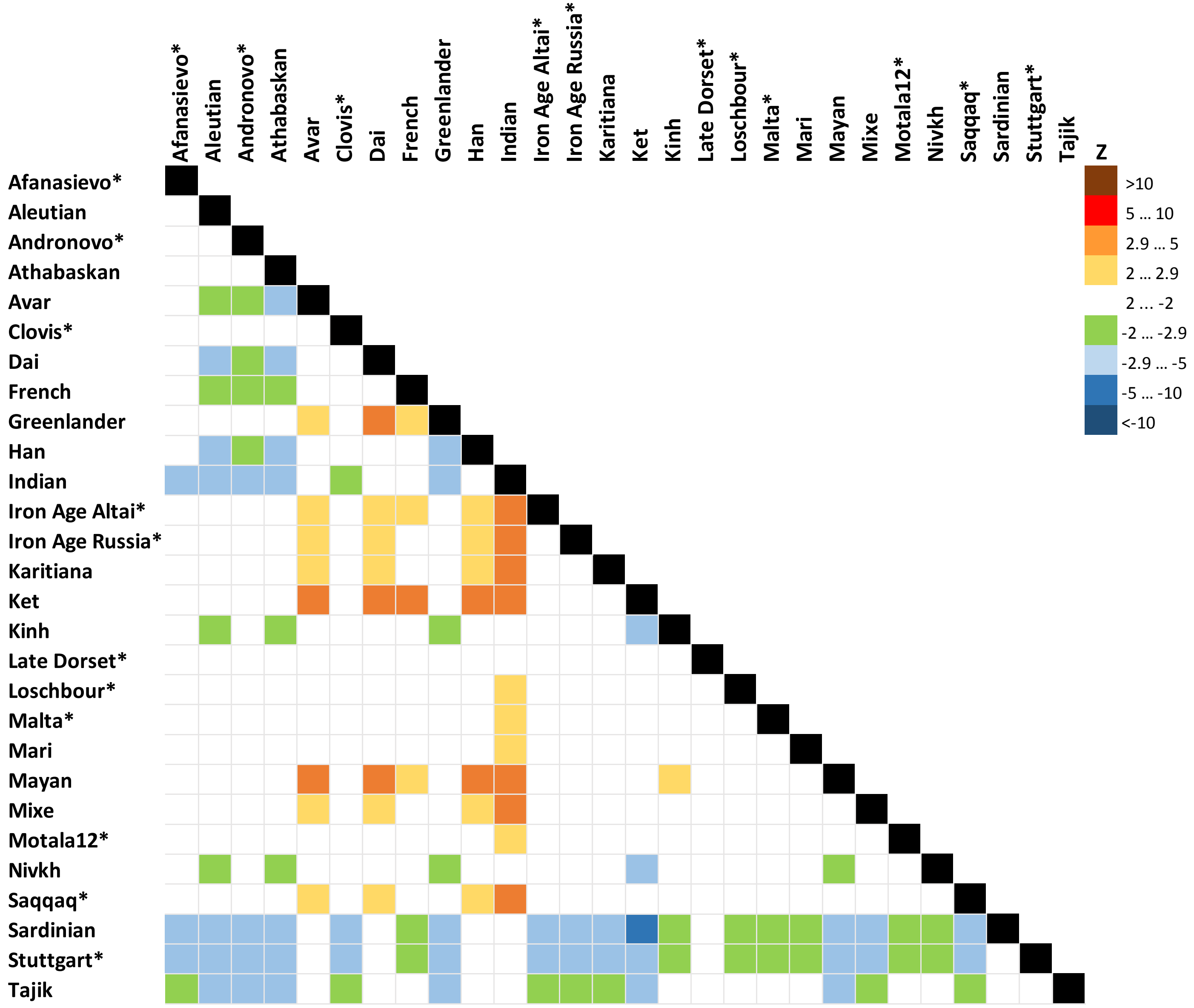
Afanasievo*
Aleutian
Andronovo*
Athabaskan
Avar
Clovis*
Dai
French
Greenlander
Han
Indian
Iron Age Altai*
Iron Age Russia*
Karitiana
Ket
Kinh
Late Dorset*
Loschbour*
Malta*
Mari
Mayan
Mixe
Motala12*
Nivkh
Saqqaq*
Sardinian
Stuttgart*
Tajik
Z
>10
5 ... 10
2.9 ... 5
2 ... 2.9
2 ... -2
-2 ... -2.9
-2.9 ... -5
-5 ... -10
<-10

8.18. Statistics f_4(Saqqaq, Yoruba; Y, X) computed on the genome-based dataset with African, Australian and Papuan populations removed, on the original version (**A**) and on the dataset with transitions excluded (**B**). A matrix of color-coded Z-scores is shown, ancient genomes are marked with asterisks. Z-score equals the number of standard errors by which the statistic differs from zero, and |Z| > 2.9 demonstrates that the statistic is significantly different from zero using Bonferroni correction for 27 independent tests (threshold *p*-value of 0.001852). Rows show Z-scores for f_4(Saqqaq, Yoruba; row, column), *vice versa* for columns. **A**

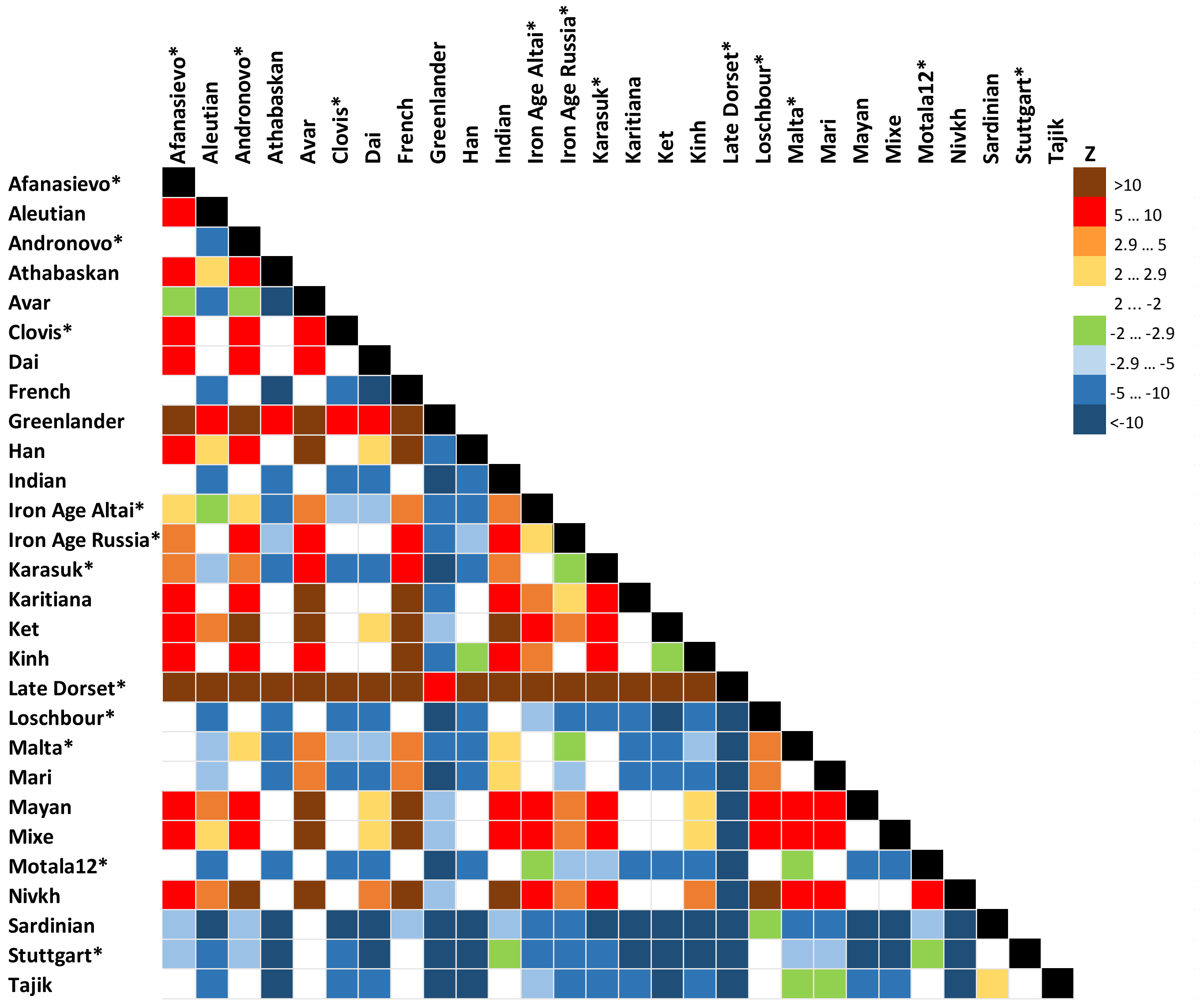

B

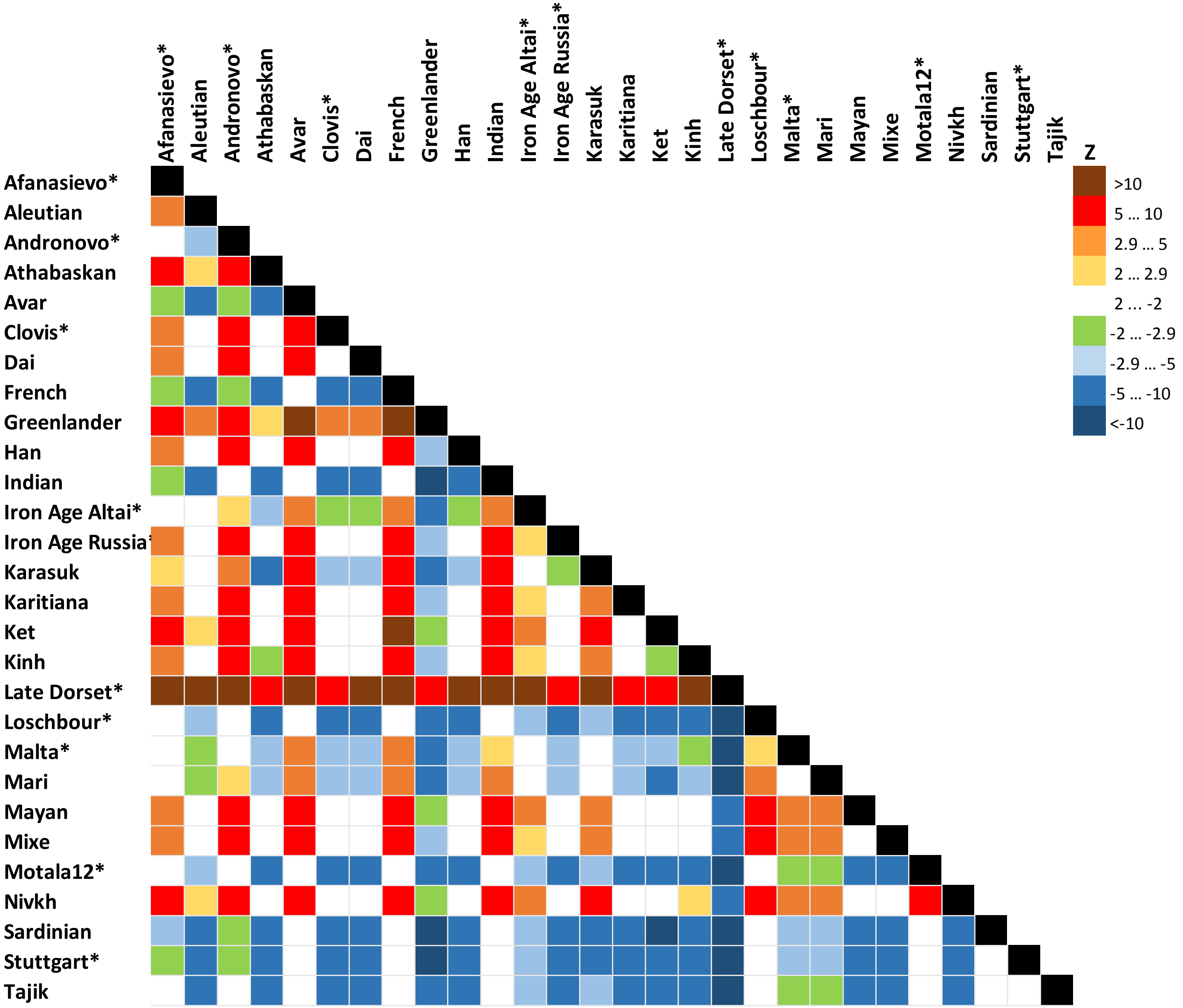

Afanasievo*
Aleutian
Andronovo*
Athabaskan
Avar
Clovis*
Dai
French
Greenlander
Han
Indian
Iron Age Altai*
Iron Age Russia*
Karasuk
Karitiana
Ket
Kinh
Late Dorset*
Loschbour*
Malta*
Mari
Mayan
Mixe
Motala12*
Nivkh
Sardinian
Stuttgart*
Tajik
Z
>10
5 ... 10
2.9 ... 5
2 ... 2.9
2 ... -2
-2 ... -2.9
-2.9 ... -5
-5 ... -10
<-10

8.19. Statistics f_4(Athabaskan, Yoruba; Y, X) computed on the genome-based dataset with African, Australian and Papuan populations removed, on the original version (**A**) and on the dataset with transitions excluded (**B**). A matrix of color-coded Z-scores is shown, ancient genomes are marked with asterisks. Z-score equals the number of standard errors by which the statistic differs from zero, and |Z| > 2.9 demonstrates that the statistic is significantly different from zero using Bonferroni correction for 27 independent tests (threshold p-value of 0.001852). Rows show Z-scores for f_4(Athabaskan, Yoruba; row, column), *vice versa* for columns. **A**

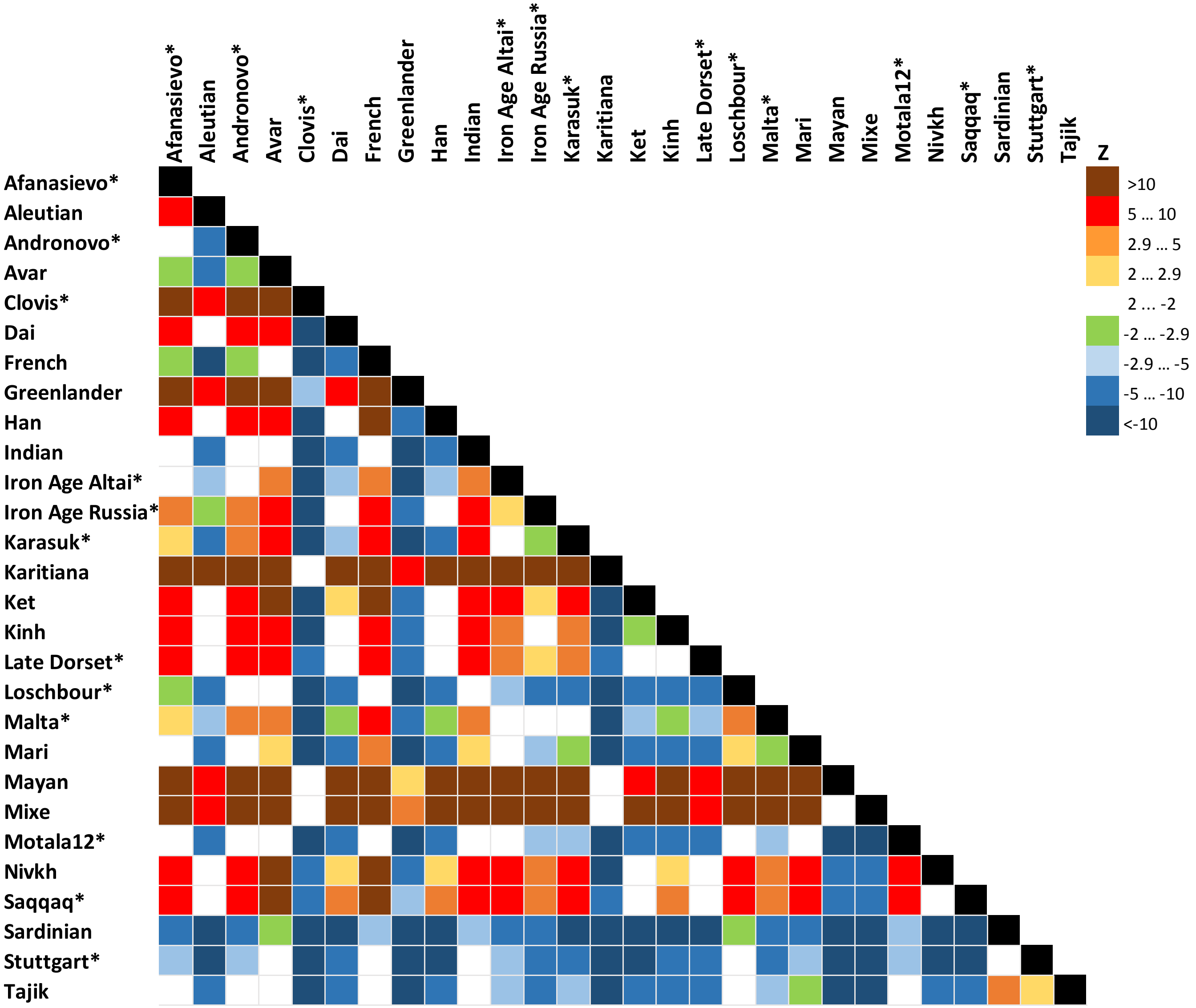

B

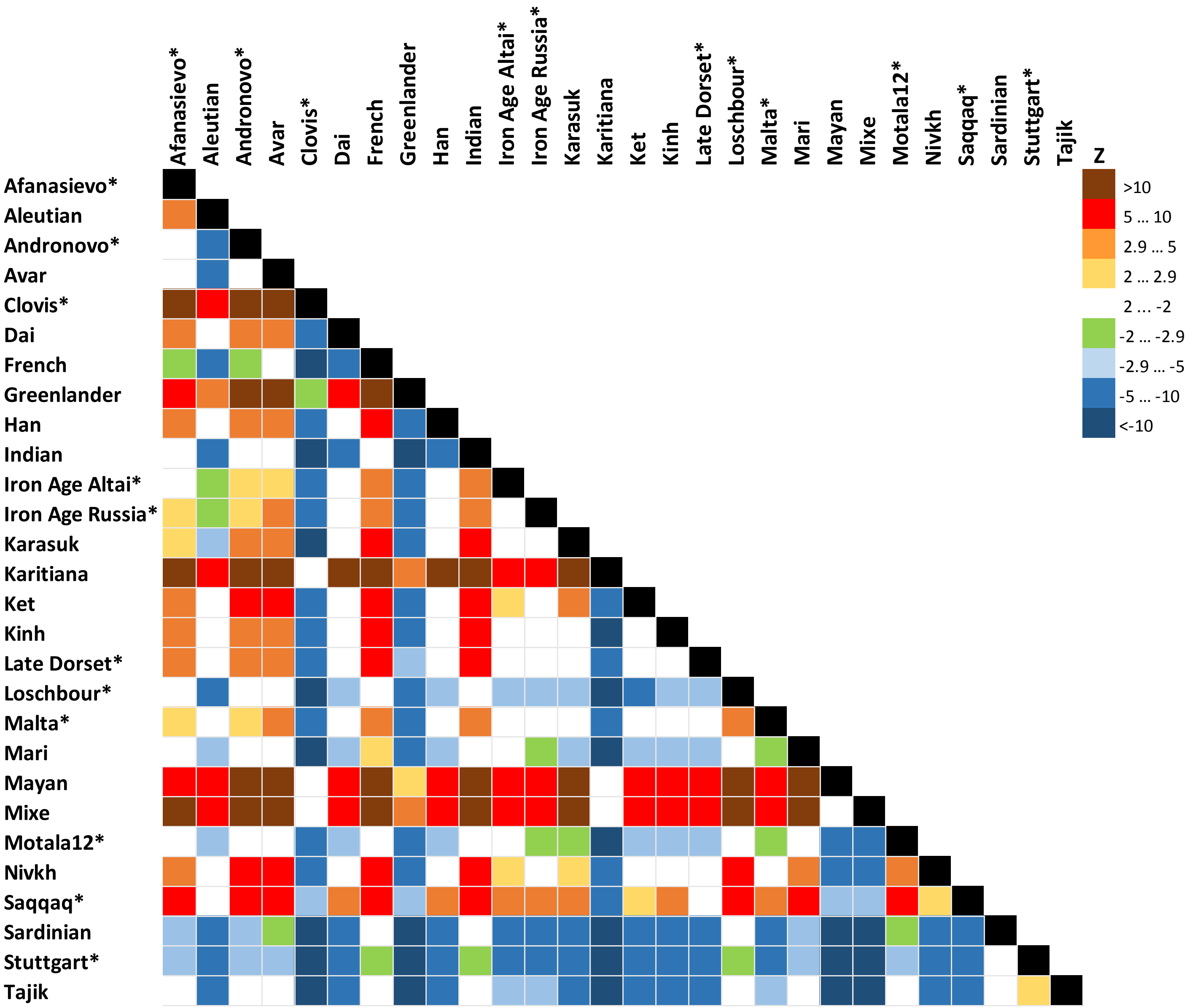
Afanasievo*
Aleutian
Andronovo*
Avar
Clovis*
Dai
French
Greenlander
Han
Indian
Iron Age Altai*
Iron Age Russia*
Karasuk
Karitiana
Ket
Kinh
Late Dorset*
Loschbour*
Malta*
Mari
Mayan
Mixe
Motala12*
Nivkh
Saqqaq*
Sardinian
Stuttgart*
Tajik
Z
>10
5 ... 10
2.9 ... 5
2 ... 2.9
2 ... -2
-2 ... -2.9
-2.9 ... -5
-5 ... -10
<-10

9. TreeMix analysis

TreeMix v.1.12 (Pickrell and Pritchard 2012) was applied to the genome-based dataset (36 populations, final SNP count 398,163) and on its version without transitions (final SNP count 189,964) with a window of 5 SNPs (option -k 5) and the root set to the San population. For each *m* (number of migration edges) from 0 to 8, 100 iterations were performed, and a tree with the highest likelihood (and with the highest explained variance percentage among trees with identical likelihoods) was selected. For a complete list of options see Methods. One hundred bootstrap replicates were calculated for trees with *m* from 6 to 8. Statistics for all edges modelled in the best trees at *m* from 1 to 8 are presented in Suppl. Table 7. Trees, percentage of explained variance, and residuals are shown below for the following counts of migration edges: 6, 7, and 8 for the original dataset and 6 and 8 for its version without transitions. The tree with 7 migration edges for the dataset without transitions is shown in Fig. 3A. Direction of an edge inferred by TreeMix was not considered critical as it was usually the least stable feature, depending on various dataset parameters (see, e.g., the Beringian - Paleo-Eskimo edges in Suppl. Table 7).

Migration edges from Paleo-Eskimos to Siberian populations appear at *m* from 4 to 8 in case of the original dataset, and at *m* 7 and 8 in case of the dataset without transitions. These edges connect the (Saqqaq, Late Dorset) or Saqqaq clades with various clades including Kets: Ket, (Ket, Karasuk), (Ket, Iron Age Russia), (Ket, Mari) (Suppl. Table 7). Migration weight ranges from 8 to 22.7% depending on the dataset and on the number of migration edges, and bootstrap support reaches 52 (Suppl. Table 7). Remarkably, migration weights calculated on the dataset without transitions (8 and 8.9%) are similar to the proportion of the Ket-Uralic admixture component modelled in Saqqaq: 7.2% (Fig. 1C) or 6.3% (Suppl. Fig. 5.6). However, Siberian ancestry in Paleo-Eskimos modelled by these migration edges is much lower than the sum of all three Siberian admixture components in Saqqaq (32% at K=19 in Fig. 1A and 28% at K=23 in Suppl. Fig. 5.4), or the Siberian admixture component at K=5 in the original Saqqaq paper (~25%, Rasmussen et al. 2010), or Siberian ancestry in Saqqaq calculated using f_4-ratios (ranging from 31% to 57% depending on dataset and reference populations, Suppl. Table 6).

In trees made on the original dataset with *m*=5 and 6 Kets form a clade with the Karasuk culture ancient population from the Bronze Age of the Altai region (Suppl. Fig. 9.1). The low bootstrap support of this clade (46 at *m*=6) is explained by the fact that Kets alternatively form a clade with another ancient genome from the Altai, Iron Age Russia (Suppl. Fig. 9.2), or with Mari (Suppl. Fig. 9.3), or form a paraphyletic assemblage with the Karasuk, Iron Age Altai and Iron Age Russia samples (Fig. 3A, Suppl. Figs. 9.4, 9.5). Genetic proximity of the Karasuk culture to Kets was also demonstrated by f_3 statistic (Yoruba; Karasuk, X) on the genome-based dataset (Suppl. Figs. 7.5, 7.6), and the Karasuk culture has been tentatively associated with Yeniseian-speaking people using hydronymic data (Chlenova 1975).

Other edge groups (Suppl. Table 7) correlating with published data or reaching bootstrap support >50 are briefly discussed below. European, or more specifically, ANE ancestry in Karasuk, connected to earlier Andronovo and Afanasievo cultures (Allentoft et al. 2015), is manifested by edges from Andronovo, Afanasievo or Mal'ta to Karasuk or the (Karasuk, Ket) clade: migration weight from 39.9

to 45.9%, bootstrap support up to 66 (Suppl. Table 7). ANE ancestry in the Afanasievo culture (Allentoft et al. 2015) is manifested by the Afanasievo-Mal'ta edges (Suppl. Table 7), and ANE ancestry in Native Americans (Raghavan et al. 2014a) is well modelled on our dataset: migration weight ranges from 28.3 to 42.6%, bootstrap support up to 72 (cf. 30-40% of Mal'ta ancestry estimated in Native Americans (Raghavan et al. 2014a, Lazaridis et al. 2014)). Another group of edges connecting Greenlander and the (Saqqaq, Late Dorset) clade apparently reflects Beringian ancestry or admixture in Paleo-Eskimos (see Results and Discussion): migration weight from 33.8 to 39.7%, bootstrap support up to 78. The Beringian admixture component reaches ~57% in the Saqqaq genome (K=5, Rasmussen et al. 2010).

Robust edges connect the Kinh (Vietnamese) branch (or a wider East Asian clade) to the basal node of Papuans and Australians: migration weight from 25.4 to 49.6%, bootstrap support up to 77 (Suppl. Table 7). This group of edges may represent Australian and Melanesian admixture in South-East Asian populations (Rasmussen et al. 2011). European ancestry in the Aleutian genome (39% at K=10 according to Raghavan et al. 2014b) was modelled by the Andronovo-Aleut in the dataset without transitions: migration weight from 43.5 to 44.4%, bootstrap support up to 62 (Suppl. Table 7). Beringian ancestry in Athabaskans (Raghavan et al. 2015) was modelled by the Athabaskan-Greenlander migration edges at *m*=4 and 5 on the dataset without transitions: migration weight from 36.9 to 37%

References

Allentoft, M. E. *et al*. Population genomics of Bronze Age Eurasia. Nature. **522,** 167–172 (2015).

Chlenova, N. L. Sootnoshenie kul'tur karasukskogo tipa i ketskikh toponimov na territorii Sibiri [The correlation between Karasuk-type cultures and Ket toponyms in Siberia]. *Etnogenez i Etnicheskaya Istoriya Narodov Severa [Ethnogenesis and History of the Peoples of the North]*. Moscow: Nauka, 223–230 (1975).

Lazaridis, I. *et al.* Ancient human genomes suggest three ancestral populations for present-day Europeans. *Nature* **513,** 409–413 (2014).

Pickrell, J. K., Pritchard, J. K. Inference of population splits and mixtures from genome-wide allele frequency data. *PLoS Genet.* **8,** e1002967 (2012).

Raghavan, M. *et al.* Upper Palaeolithic Siberian genome reveals dual ancestry of Native Americans. *Nature* **505,** 87–91 (2014a).

Raghavan, M. *et al.* The genetic prehistory of the New World Arctic. *Science* **345,** 1255832 (2014b).

Raghavan, M. *et al.* Genomic evidence for the Pleistocene and recent population history of Native Americans. *Science* doi: 10.1126/science.aab3884 (2015).

Rasmussen, M. *et al.* Ancient human genome sequence of an extinct Palaeo-Eskimo. *Nature* **463,** 757–62 (2010).

Rasmussen, M. *et al.* An Aboriginal Australian genome reveals separate human dispersals into Asia. *Science* **334,** 94–98 (2011).

Suppl. Table 7. Migration edges in TreeMix trees with 1 to 8 migration edges, grouped according to published data on population relationships. The following abbreviations are used: b., branch; n., node; ANE, ancient North Eurasians; WHG, West European hunter-gatherers. In the names for the edge groups hypothetical sources of ancestry are placed in the beginning. Bootstrap support values ≥50 are considered reliable and are highlighted in bold.

edge group	dataset	*m*	from	to	weight, %	bootstrap support
African - East Asian	original	3	San b.	Australian, Papuan, East Asian n.	8.3	N/A
African - East Asian	original	4	San b.	Australian, Papuan, East Asian n.	9.7	N/A
African - East Asian	original	5	Yoruba, Mandenka, Dinka b.	Saqqaq, Late Dorset, Nivkh, Han, Dai, Kinh n.	8.9	N/A
African - East Asian	original	6	Yoruba, Mandenka, Dinka b.	Saqqaq, Late Dorset, Nivkh, Han, Dai, Kinh n.	8.7	14
African - East Asian	original	7	Yoruba, Mandenka, Dinka b.	Saqqaq, Late Dorset, Nivkh, Han, Dai, Kinh n.	8.6	24
African - East Asian	original	8	Yoruba, Mandenka, Dinka b.	Saqqaq, Late Dorset, Nivkh, Han, Dai, Kinh n.	8.3	36
African - East Asian	without transitions	3	Yoruba, Mandenka, Dinka b.	Nivkh, Han, Dai, Kinh n.	11.1	N/A
African - East Asian	without transitions	4	Yoruba, Mandenka, Dinka b.	Nivkh, Han, Dai, Kinh n.	10.6	N/A
African - East Asian	without transitions	5	Yoruba, Mandenka, Dinka b.	Nivkh, Han, Dai, Kinh n.	10.4	N/A
African - East Asian	without transitions	6	Yoruba, Mandenka, Dinka b.	Nivkh, Han, Dai, Kinh n.	9.9	26
African - East Asian	without transitions	7	Yoruba, Mandenka, Dinka b.	Nivkh, Han, Dai, Kinh n.	9.8	30
African - East Asian	without transitions	8	Yoruba, Mandenka, Dinka b.	Nivkh, Han, Dai, Kinh n.	10.1	33
ANE - Karasuk	original	5	Andronovo b.	Karasuk	44.7	N/A
ANE - Karasuk	original	6	Afanasievo, Mal'ta b.	Karasuk	45.9	**65**
ANE - Karasuk	original	6	Ket, Karasuk b.	Mal'ta	42.9	12
ANE - Karasuk	original	7	Afanasievo b.	Karasuk	42.3	**62**
ANE - Karasuk	original	8	Afanasievo b.	Karasuk	39.9	**66**
ANE - Karasuk	without transitions	3	Andronovo b.	Karasuk	43.1	N/A
ANE - Karasuk	without transitions	4	Andronovo b.	Karasuk	43	N/A
ANE - Karasuk	without transitions	5	Avar, Afanasievo b.	Karasuk	49	N/A
ANE - Karasuk	without transitions	6	Andronovo b.	Karasuk	45.4	23
ANE - Karasuk	without transitions	7	Andronovo b.	Karasuk	43.8	24
ANE - Karasuk	without transitions	8	Andronovo b.	Karasuk	43.2	29
ANE - Native Americans	original	7	Mal'ta b.	Greenlander, Native American n.	35.6	**72**
ANE - Native Americans	original	8	Mal'ta b.	Greenlander, Native American n.	42.6	**70**
ANE - Native Americans	without transitions	5	Athabaskan b.	Mal'ta	28.3	N/A
ANE - Native Americans	without transitions	6	Native American b.	Mal'ta	30.5	**50**
ANE - Native Americans	without transitions	7	Mayan, Clovis, Karitiana, Mixe b.	Mal'ta	30.6	48
ANE - Native Americans	without transitions	8	Native American b.	Mal'ta	30.3	**66**
ANE - post-Yamnaya cultures	original	7	Afanasievo b.	Mal'ta	68.4	25
ANE - post-Yamnaya cultures	original	8	Afanasievo b.	Mal'ta	65.7	33
Australian/Papuan - Kinh	original	1	Kinh b.	Australian, Papuan n.	49.7	N/A
Australian/Papuan - Kinh	original	5	Kinh b.	Australian, Papuan n.	49.6	N/A
Australian/Papuan - Kinh	original	6	Kinh b.	Australian, Papuan n.	49.2	**66**

Australian/Papuan - Kinh	original	7	Kinh b.	Australian, Papuan n.	48.9	**69**
Australian/Papuan - Kinh	original	8	Kinh b.	Australian, Papuan n.	48.4	**66**
Australian/Papuan - Kinh	without transitions	1	Australian, Papuan b.	Han, Dai, Kinh n.	28.6	N/A
Australian/Papuan - Kinh	without transitions	2	Australian b.	Nivkh, Han, Dai, Kinh n.	25.4	N/A
Australian/Papuan - Kinh	without transitions	3	Kinh b.	Australian, Papuan n.	46.4	N/A
Australian/Papuan - Kinh	without transitions	4	Kinh b.	Australian, Papuan n.	46.5	N/A
Australian/Papuan - Kinh	without transitions	5	Kinh b.	Australian, Papuan n.	46	N/A
Australian/Papuan - Kinh	without transitions	6	Kinh b.	Australian, Papuan n.	45.4	**73**
Australian/Papuan - Kinh	without transitions	7	Kinh b.	Australian, Papuan n.	45.5	**77**
Australian/Papuan - Kinh	without transitions	8	Kinh b.	Australian, Papuan n.	45.6	**76**
Beringian - Athabaskan	without transitions	4	Athabaskan b.	Greenlander	36.9	N/A
Beringian - Athabaskan	without transitions	5	Athabaskan b.	Greenlander	37	N/A
Beringian - Paleo-Eskimo	original	3	Greenlander b.	Saqqaq, Late Dorset n.	38.4	N/A
Beringian - Paleo-Eskimo	original	4	Greenlander b.	Saqqaq, Late Dorset n.	35.3	N/A
Beringian - Paleo-Eskimo	original	5	Greenlander b.	Saqqaq, Late Dorset n.	35.8	N/A
Beringian - Paleo-Eskimo	original	6	Greenlander b.	Saqqaq, Late Dorset n.	36.1	**76**
Beringian - Paleo-Eskimo	original	7	Greenlander b.	Saqqaq, Late Dorset n.	38.4	**75**
Beringian - Paleo-Eskimo	original	8	Greenlander b.	Saqqaq, Late Dorset n.	39.7	**78**
Beringian - Paleo-Eskimo	without transitions	6	Saqqaq, Late Dorset b.	Greenlander	35.2	42
Beringian - Paleo-Eskimo	without transitions	7	Saqqaq, Late Dorset b.	Greenlander	33.8	46
Beringian - Paleo-Eskimo	without transitions	8	Saqqaq, Late Dorset b.	Greenlander	34.4	**52**
East Asian - Iron Age Russia	without transitions	8	Iron Age Russia b.	Nivkh, Han, Dai, Kinh n.	4	24
European - Aleut	without transitions	6	Andronovo b.	Aleut	44.4	46
European - Aleut	without transitions	7	Andronovo b.	Aleut	43.9	**58**
European - Aleut	without transitions	8	Andronovo b.	Aleut	43.5	**62**
Siberian - Paleo-Eskimo	original	4	Saqqaq, Late Dorset b.	Ket, Iron Age Russia n.	22.7	N/A
Siberian - Paleo-Eskimo	original	5	Saqqaq, Late Dorset b.	Ket, Karasuk n.	10.6	N/A
Siberian - Paleo-Eskimo	original	6	Saqqaq, Late Dorset b.	Ket, Karasuk n.	10.5	35
Siberian - Paleo-Eskimo	original	7	Saqqaq b.	Ket, Iron Age Russia n.	12.4	49
Siberian - Paleo-Eskimo	original	8	Saqqaq b.	Ket, Mari n.	15.1	**52**
Siberian - Paleo-Eskimo	without transitions	7	Saqqaq b.	Ket	8	43
Siberian - Paleo-Eskimo	without transitions	8	Saqqaq b.	Ket	8.9	48
WHG - Mari	original	8	Loschbour, Motala12 b.	Mari	62.1	21
?	original	2	Saqqaq, Late Dorset b.	Nivkh, Han, Dai, Kinh n.	66.3	N/A
?	original	2	Saqqaq, Late Dorset b.	Australian, Papuan, Nivkh, Han, Dai, Kinh n.	49.7	N/A
?	original	3	Eurasian b.	Australian, Papuan n.	49.6	N/A
?	original	4	Eurasian b.	Australian, Papuan n.	49.7	N/A
?	without transitions	2	Native American b.	Australian, Papuan n.	37.1	N/A

9.1. A. TreeMix tree, the genome-based dataset, 6 migration edges. Drift parameter is shown on the x-axis, migration weight is color-coded. Migration edges are numbered according to their order of appearance in the sequence of trees from *m*=0 to *m*=8, with edge weight and bootstrap support values shown in the table. Note to the figure: as migration edges and tree topology are inter-dependent in bootstrapped trees, bootstrap support for the edges in the original tree was calculated by summing up support for closely similar edges in bootstrapped trees. Below these edge groups are listed for edges #1-6: 1/ Australian and/or Papuan ⇔ the (Nivkh, Han, Dai, Kinh) clade or any of its members; 2/ Greenlander Inuit or the (Greenlander, Aleutian) clade ⇔ Saqqaq and/or Late Dorset (optionally a wider clade with Nivkh); 3/ any clade containing African populations ⇔ any clade composed of Nivkh/Han/Dai/Kinh (optionally a wider clade with Late Dorset and/or Saqqaq and/or Iron Age Altai); 4/ Ket (optionally a wider clade with Karasuk and/or Iron Age Altai and/or Iron Age Russia) ⇔ Saqqaq and/or Late Dorset; 5/ any clade composed of Mal'ta/Afanasievo/Andronovo (optionally a wider clade with Aleutian and/or Mari) ⇔ Karasuk; 6/ any clade composed of Mal'ta/Afanasievo/Andronovo (optionally a wider clade with Aleutian and/or Mari) ⇔ Ket, or the (Ket, Karasuk) clade. **B.** Residuals from the fit of the model to the data visualized. 97.18% variance is explained by the tree.

A

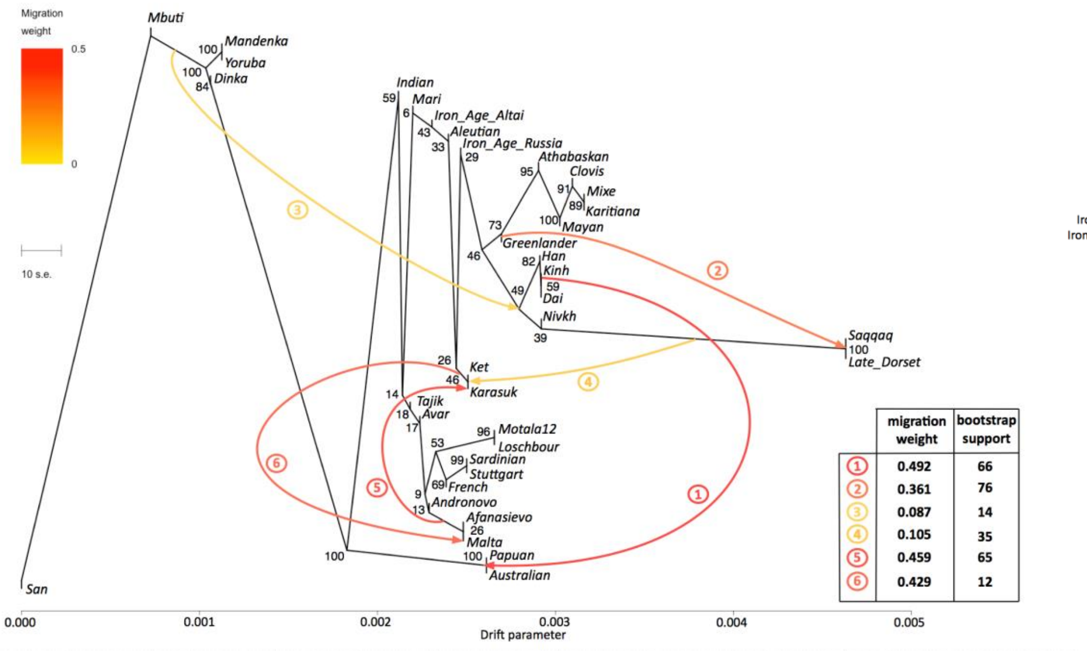

B

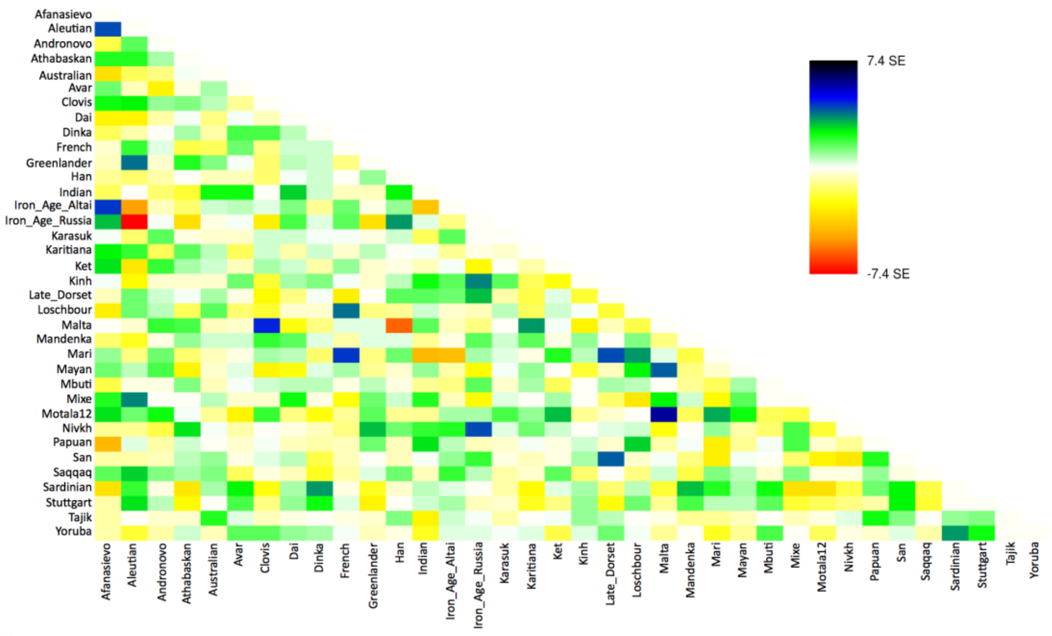

9.2. A. TreeMix tree, the genome-based dataset, 7 migration edges. Drift parameter is shown on the x-axis, migration weight is color-coded. Migration edges are numbered according to their order of appearance in the sequence of trees from *m*=0 to *m*=8, with edge weight and bootstrap support values shown in the table. Note to the figure: as migration edges and tree topology are inter-dependent in bootstrapped trees, bootstrap support for the edges in the original tree was calculated by summing up support for closely similar edges in bootstrapped trees. Below these edge groups are listed for edges #1-7: 1/ Australian and/or Papuan ⇔ the (Nivkh, Han, Dai, Kinh) clade or any of its members; 2/ Greenlander Inuit or the (Greenlander, Aleutian) clade ⇔ Saqqaq and/or Late Dorset (optionally a wider clade with Nivkh); 3/ any clade containing African populations ⇔ any clade composed of Nivkh/Han/Dai/Kinh (optionally a wider clade with Late Dorset and/or Saqqaq and/or Iron Age Altai); 4/ Ket (optionally a wider clade with Karasuk and/or Iron Age Altai and/or Iron Age Russia) ⇔ Saqqaq and/or Late Dorset; 5/ any clade composed of Mal'ta/Afanasievo/Andronovo (optionally a wider clade with Aleutian and/or Mari) ⇔ Karasuk; 6/ Mal'ta ⇔ any clade composed of Afanasievo/Andronovo (optionally a wider clade with Avar); 7/ Mal'ta (optionally a wider clade with Motala12/Afanasievo/Andronovo/Aleut) ⇔ any clade composed exclusively of Native Americans and/or Greenlander. **B.** Residuals from the fit of the model to the data visualized. 97.51% variance is explained by the tree.

A

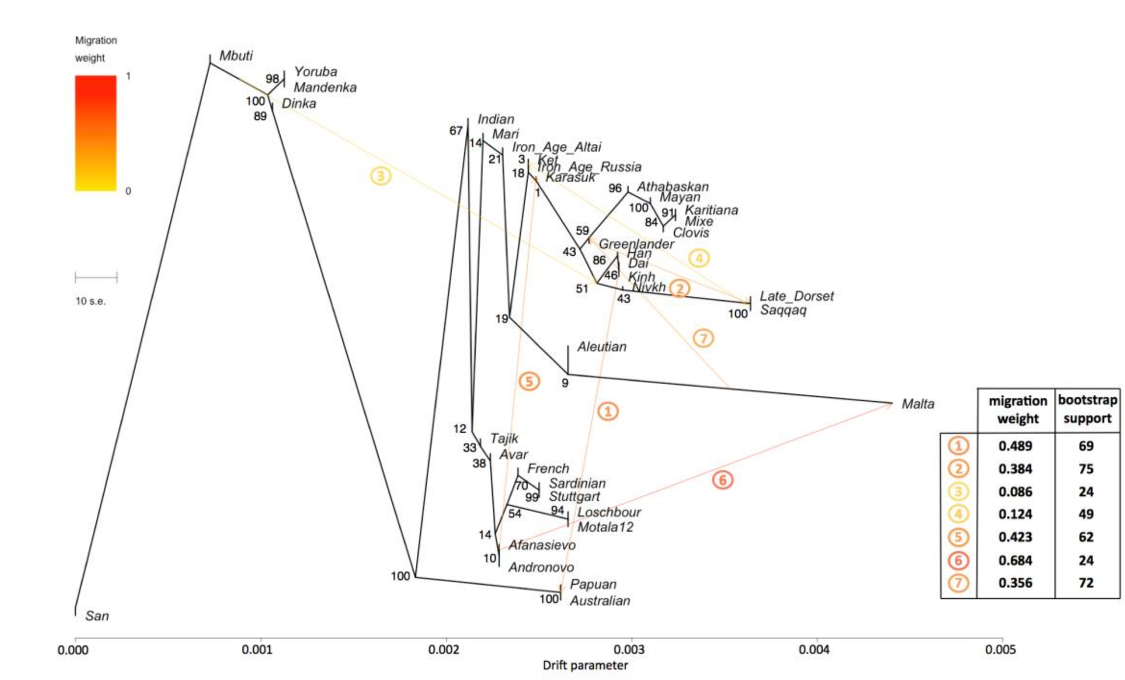

	migration weight	bootstrap support
1	0.489	69
2	0.384	75
3	0.086	24
4	0.124	49
5	0.423	62
6	0.684	24
7	0.356	72

B

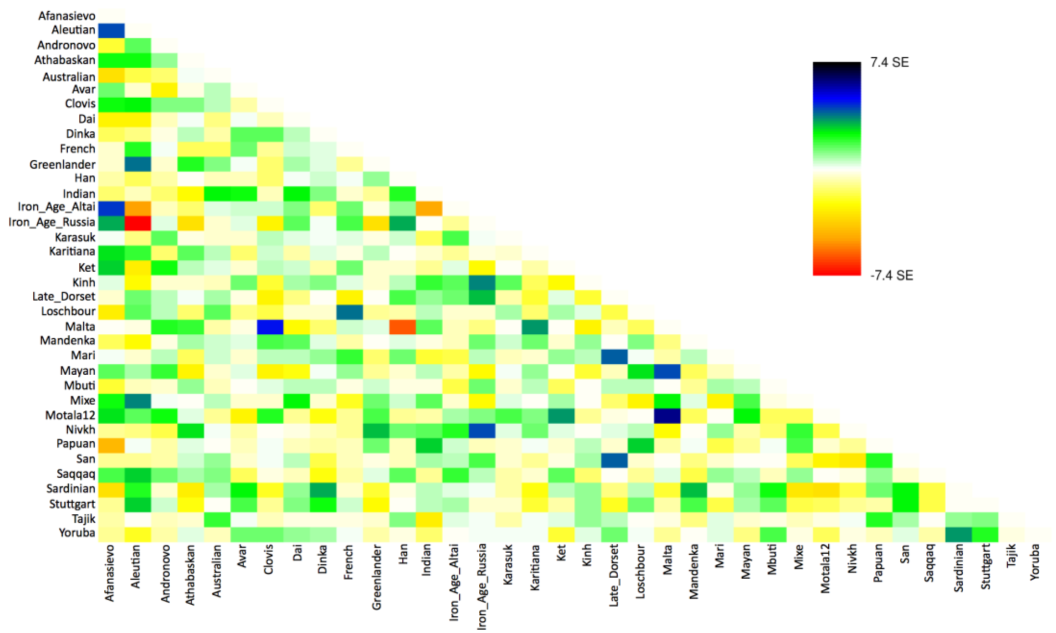

9.3. A. TreeMix tree, the genome-based dataset, 8 migration edges. Drift parameter is shown on the x-axis, migration weight is color-coded. Migration edges are numbered according to their order of appearance in the sequence of trees from *m*=0 to *m*=8, with edge weight and bootstrap support values shown in the table. Note to the figure: as migration edges and tree topology are inter-dependent in bootstrapped trees, bootstrap support for the edges in the original tree was calculated by summing up support for closely similar edges in bootstrapped trees. Below these edge groups are listed for edges #1-8: 1/ Australian and/or Papuan ⇔ the (Nivkh, Han, Dai, Kinh) clade or any of its members; 2/ Greenlander Inuit or the (Greenlander, Aleutian) clade ⇔ Saqqaq and/or Late Dorset (optionally a wider clade with Nivkh); 3/ any clade containing African populations ⇔ any clade composed of Nivkh/Han/Dai/Kinh (optionally a wider clade with Late Dorset and/or Saqqaq and/or Iron Age Altai); 4/ Ket (optionally a wider clade with Karasuk and/or Iron Age Altai and/or Iron Age Russia) ⇔ Saqqaq and/or Late Dorset; 5/ any clade composed of Mal'ta/Afanasievo/Andronovo (optionally a wider clade with Aleutian and/or Mari) ⇔ Karasuk (optionally a wider clade with Ket and/or Iron Age Russia); 6/ Mal'ta ⇔ any clade composed of Afanasievo/Andronovo (optionally a wider clade with Avar); 7/ Mal'ta (optionally a wider clade with Motala12/Afanasievo/Andronovo/Aleut) ⇔ any clade composed exclusively of Native Americans and/or Greenlander; 8/ any clade composed exclusively of populations with European ancestry ⇔ Mari. **B.** Residuals from the fit of the model to the data visualized. 97.79% variance is explained by the tree.

A

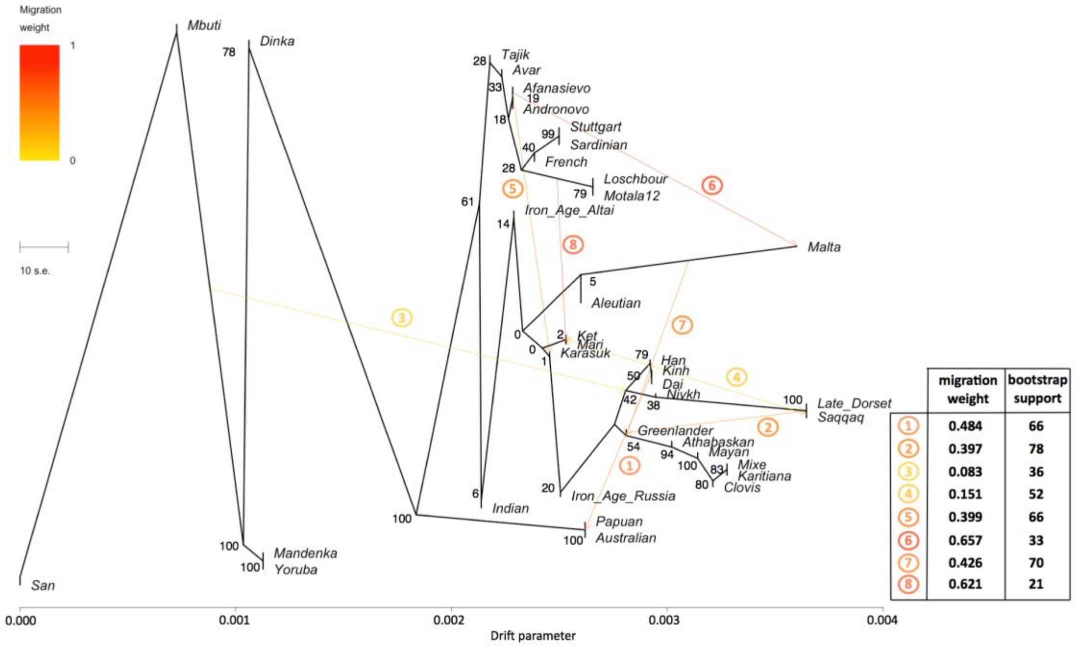

	migration weight	bootstrap support
1	0.484	66
2	0.397	78
3	0.083	36
4	0.151	52
5	0.399	66
6	0.657	33
7	0.426	70
8	0.621	21

B

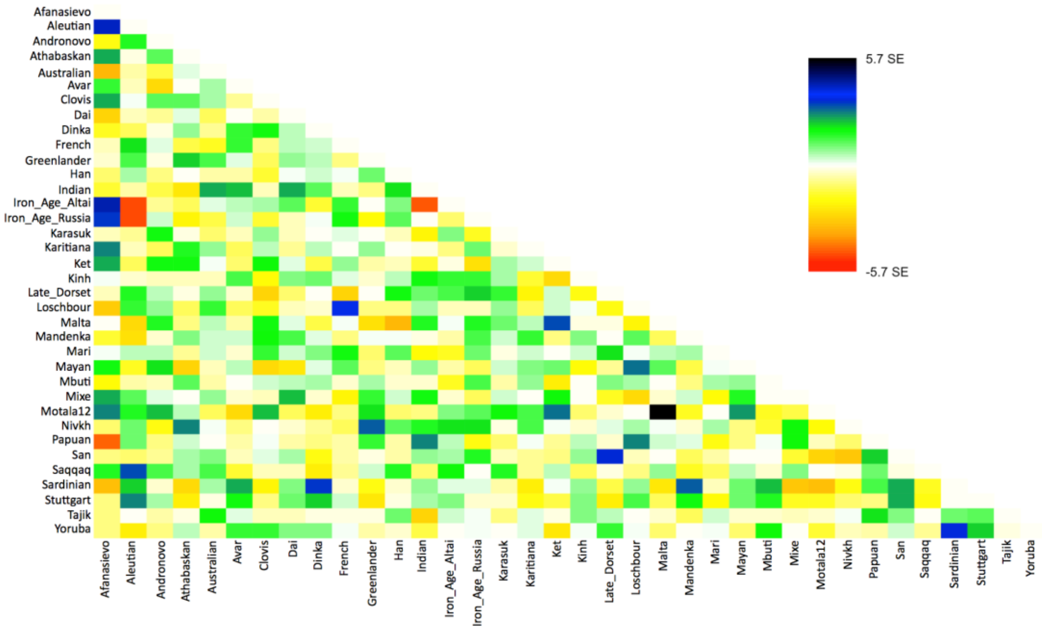

9.4. A. TreeMix tree, the dataset without transitions, 6 migration edges. Drift parameter is shown on the x-axis, migration weight is color-coded. Migration edges are numbered according to their order of appearance in the sequence of trees from *m*=0 to *m*=8, with edge weight and bootstrap support values shown in the table. Note to the figure: as migration edges and tree topology are inter-dependent in bootstrapped trees, bootstrap support for the edges in the original tree was calculated by summing up support for closely similar edges in bootstrapped trees. Below these edge groups are listed for edges #1-6: 1/ Australian and/or Papuan ⇔ the (Nivkh, Han, Dai, Kinh) clade or any of its members; 2/ Greenlander Inuit or the (Greenlander, Aleutian) clade ⇔ Saqqaq and/or Late Dorset (optionally a wider clade with Nivkh); 3/ any clade containing African populations ⇔ any clade composed of Nivkh/Han/Dai/Kinh (optionally a wider clade with Late Dorset and/or Saqqaq and/or Iron Age Altai); 4/ any clade composed of Mal'ta/Afanasievo/Andronovo (optionally a wider clade with Aleutian and/or Mari) ⇔ Karasuk; 5/ Mal'ta (optionally a wider clade with Motala12/Afanasievo/Andronovo/Aleut) ⇔ any clade composed exclusively of Native Americans and/or Greenlander; 6/ any clade composed exclusively of populations with European ancestry ⇔ Aleutian. **B.** Residuals from the fit of the model to the data visualized. 96.63% variance is explained by the tree.

A

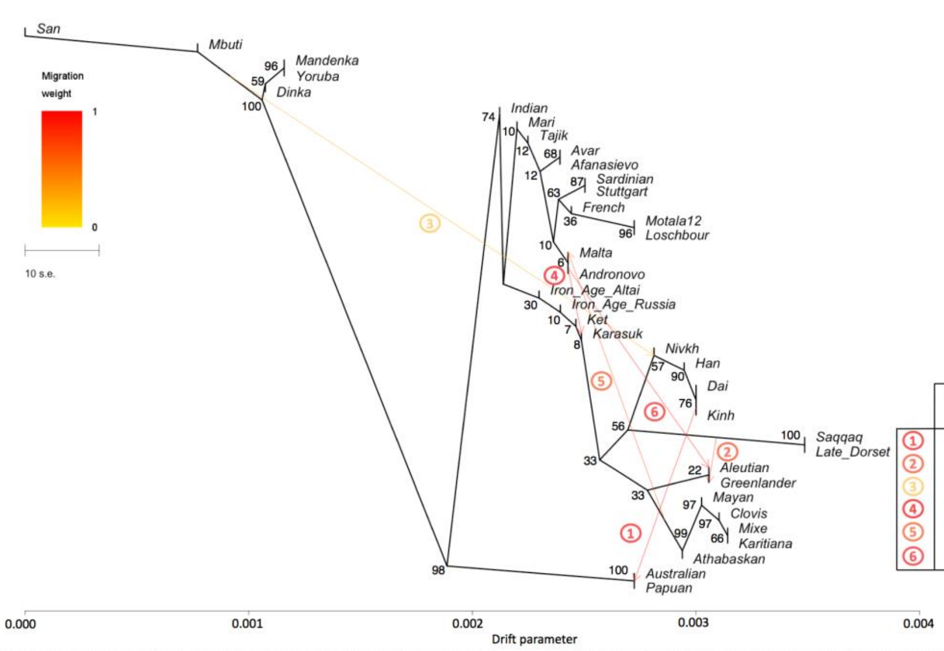

	migration weight	bootstrap support
1	0.454	73
2	0.352	42
3	0.099	26
4	0.454	23
5	0.305	50
6	0.444	46

B

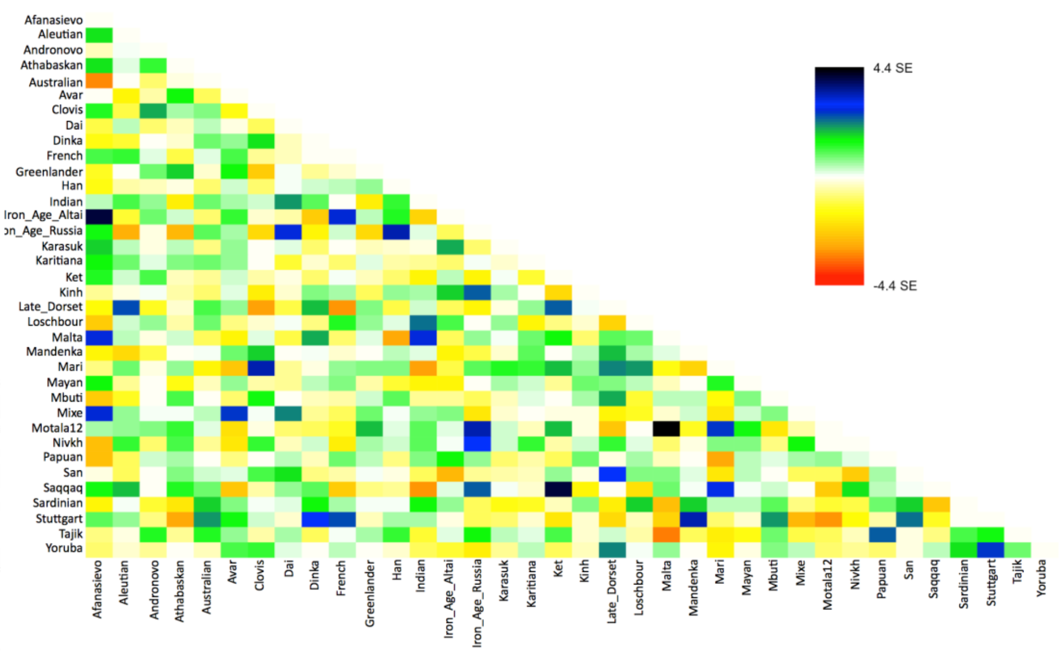

9.5. A. TreeMix tree, the dataset without transitions, 8 migration edges. Drift parameter is shown on the x-axis. Migration edges are numbered according to their order of appearance in the sequence of trees from *m*=0 to *m*=8, with edge weight and bootstrap support values shown in the table. Note to the figure: as migration edges and tree topology are inter-dependent in bootstrapped trees, bootstrap support for the edges in the original tree was calculated by summing up support for closely similar edges in bootstrapped trees. Below these edge groups are listed for edges #1-8: 1/ Australian and/or Papuan ⇔ the (Nivkh, Han, Dai, Kinh) clade or any of its members; 2/ Greenlander Inuit or the (Greenlander, Aleutian) clade ⇔ Saqqaq and/or Late Dorset (optionally a wider clade with Nivkh); 3/ any clade containing African populations ⇔ any clade composed of Nivkh/Han/Dai/Kinh (optionally a wider clade with Late Dorset and/or Saqqaq and/or Iron Age Altai); 4/ any clade composed of Mal'ta/Afanasievo/Andronovo (optionally a wider clade with Aleutian and/or Mari) ⇔ Karasuk; 5/ Mal'ta (optionally a wider clade with Motala12/Afanasievo/Andronovo/Aleut) ⇔ any clade composed exclusively of Native Americans and/or Greenlander; 6/ any clade composed exclusively of populations with European ancestry ⇔ Aleutian; 7/ Ket (optionally a wider clade with Karasuk and/or Iron Age Altai and/or Iron Age Russia) ⇔ Saqqaq and/or Late Dorset; 8/ any clade composed of Nivkh/Han/Dai/Kinh (optionally a wider clade with Late Dorset and/or Saqqaq) ⇔ Iron Age Russia. **B.** Residuals from the fit of the model to the data visualized. 96.93% variance is explained by the tree.

A

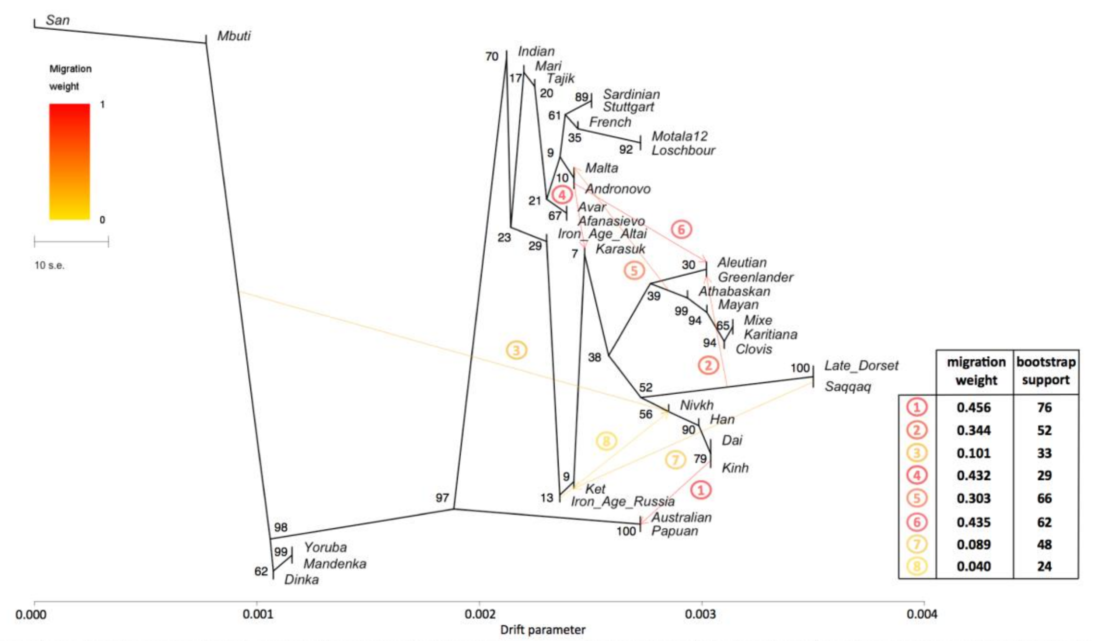

	migration weight	bootstrap support
1	0.456	76
2	0.344	52
3	0.101	33
4	0.432	29
5	0.303	66
6	0.435	62
7	0.089	48
8	0.040	24

B

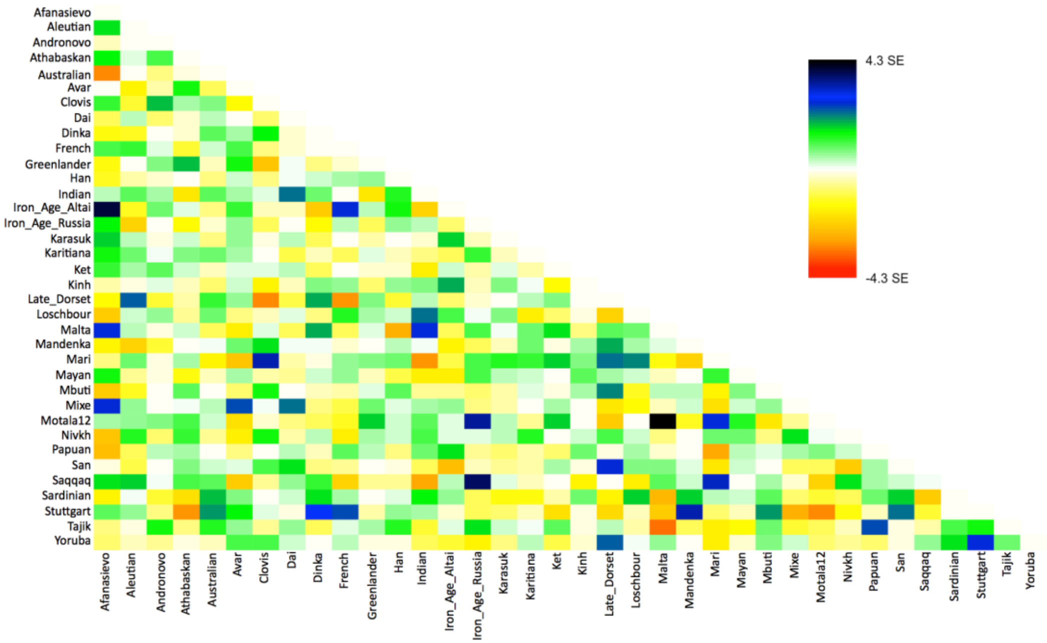

10. Mitochondrial and Y-chromosomal haplogroups

Methods

Mitochondrial genome SNPs (approximately 3,300) were genotyped with the GenoChip array in 158 individuals. SNP loci heterozygous in more than 15 samples or those with missing data in more than 15 samples were removed completely, and remaining heterozygous genotypes were filtered out in particular individuals. Mitochondrial DNA haplogroups were predicted using the MitoTool software (http://www.mitotool.org/).

SNPs typed on Y chromosome with the GenoChip array were checked and low-quality SNPs with genotyping rate <95% were removed for all 53 male individuals genotyped with GenoChip in this study. One sample (sample ID GRC14460103) was removed due to poor genotyping rate (18.7% missing markers on Y chromosome). After this quality control step, 11,883 high-quality Y-chromosomal SNPs remained for the downstream analysis. Genotyping data were transformed into a list of mutations and haplogroup prediction was performed using the Y-SNP Subclade Predictor online tool at MorleyDNA.com (http://ytree.morleydna.com/).

Mitochondrial haplogroups

We have determined mitochondrial and Y-chromosomal haplogroups based on SNP data from GenoChip: approximately 3,300 mitochondrial and 12,000 Y-chromosomal SNPs (see Methods for details). The frequencies of mitochondrial haplogroups in 46 putatively unrelated Kets in this study (Suppl. file S4) were similar to those reported previously for 38 Ket individuals (Derbeneva et al. 2002). There are just few differences between this study and the previous one: i/ two times higher proportion of haplogroup F according to Derbeneva et al. (2002), 23.7% vs 10.9% in this study; ii/ approximately two times lower proportion of haplogroup U5 according to Derbeneva et al. (2002), 5.3% vs 13% in this study (Suppl. file S4). These discrepancies are not surprising given relatively low sample sizes in both studies. Both studies show U4 as a dominant haplogroup in the Ket population, with very much similar frequency: 28.9% and 30.4%. In Nganasans, the same frequency of U4 is obtained in this study and in Derbeneva et al. 2002: 20.8% and 24 individuals in each study. However, haplogroups C (in both studies) and D (in Derbeneva et al. 2002) are dominant in Nganasans. The small Selkup sample of 15 individuals in this study had U4 as a dominant haplogroup with a frequency of 40%.

Notably, the frequency of mitochondrial haplogroup U4, predominant in Kets, correlated with proportion of the Ket-Uralic admixture component: Pearson’s correlation coefficient reached up to 0.81 (p-value 6.9×10^{-8}) in three datasets analyzed (Suppl. Table 8, Suppl. files S5-S7). The Ket-Uralic admixture component did not significantly correlate with any other major mitochondrial haplogroup, and haplogroup U4 also correlated with the 'South Asian 2' (Pearson's r 0.43, p-value 1.5×10^{-2}) and 'Siberian 1' (Pearson's r 0.57, p-value 3.7×10^{-3}) components in datasets Ket genomes + Illumina arrays and Ket genomes + HumanOrigins array, respectively (Suppl. Table 8, Suppl. files S5-S7). To provide a wider context for the observed correlation of the U4 haplogroup frequency and the Ket-Uralic admixture component proportion, we calculated Pearson's correlation coefficients for all possible pairs of major mitochondrial haplogroups and admixture components in three datasets (Suppl. files S5-S7).

Pairs with r > 0.8 and *p*-value < 0.05 are shown in Suppl. Table 8. Remarkably, in the GenoChip-based dataset, the U4/Ket-Uralic component pair had the second lowest *p*-value among all pairs. In the other two datasets, this pair was not found among the best, but had a significant *p*-value in all cases.

Ancient European hunter-gatherers had haplogroup U with >80% frequency (Malmström et al. 2009, Bramanti et al. 2009, Fu et al. 2013), and the Mal'ta individual also belonged to a basal branch of haplogroup U without affiliation to known subclades (Raghavan 2014a). Therefore, haplogroup U, especially its U4 and U5 branches (Brandt et al. 2013), may be considered as a marker of West European hunter-gatherer (WHG) and Ancient North Eurasian (ANE) ancestry. In this light, high prevalence of haplogroup U4 in Kets and Selkups (Suppl. file S4) correlates well with large degrees of ANE ancestry in these populations. Haplogroup U4 was previously suggested as a marker of Uralic-speaking ethnic groups within Russia (Malyarchuk 2004, Flegontova et al. 2009). According to a comparative haplogroup frequency table including reference populations from Brandt et al. (2013) and other studies, haplogroup U4 reaches the highest frequency (42.1%, 19 individuals) in the Pitted Ware culture of Scandinavian hunter-gatherers (3200 BC – 2300 BC). It was also frequent in ancient hunter-gatherers from Siberia, Eastern and Central Europe (Brandt et al. 2013), and in modern populations it has a peculiar geographic distribution essentially matching that of the Ket-Uralic admixture component (Suppl. file S4).

Y-chromosomal haplogroups

The frequencies of Y-chromosomal haplogroups in 20 Ket males in this study were also similar to those reported previously for 48 Ket individuals (Tambets et al. 2004): more than 90% of Kets had haplogroup Q1a (of subclade Q1a2a1 as shown in our study) (Suppl. Table 9), while haplogroups I1a2 and I2a1b3a occurred in just two Ket individuals. Using full Y-chromosome sequences, Karmin et al. (2015) determined haplogroups in two Kets and one Selkup individual as Q1a2a1c according to the ISOGG nomenclature (Q1c according to the revised nomenclature introduced in the paper cited). Despite small sample sizes, Y-haplogroup frequencies for Selkups and Nganasans (Suppl. file S7) were similar to previously reported ones. Haplogroup Q1a2a1 was predominant in Selkups: 71.4% vs. 66.4% in Tambets et al. (2004), sample sizes 7 vs. 131. Haplogroup N1c2b2 was clearly predominant in Nganasans: 93.3% vs. 92.1% in Tambets et al. (2004), sample sizes 15 vs. 38. Frequencies of other minor haplogroups in the populations studied are shown in Suppl. Table 9.

Haplogroup Q1a reaches the highest frequency in all Native American populations, a well-established fact (Lell et al. 2002, Starikovskaya et al. 2005, Dulik et al. 2012), and is also common (>30%) in few scattered Asian populations: Chelkans (Siberia, Altai region), Akha (South-East Asia), Turkmens (Central Asia), Tubalars (Siberia, Altai region), Yukaghirs (East Siberia) (Suppl. file S7). For Eurasian samples, frequency of haplogroup Q correlates well with proportion of the Ket-Uralic admixture component: Pearson's correlation coefficient reached up to 0.91 (*p*-value 2.4×10^{-7}) in three datasets analyzed (Suppl. Table 8, Suppl. files S8-S10). However, the correlation becomes much weaker if Native American and Beringian populations are included (data not shown) as they demonstrate close to 0% of the Ket-Uralic component, but up to 100% frequency of haplogroup Q1a. The other major Y-chromosomal haplogroups demonstrated a weaker correlation with the Ket-Uralic admixture

component: only haplogroup N showed a significant *p*-value in the case of dataset Ket genome + Illumina arrays (Suppl. Table 8). Haplogroup Q did not correlate significantly with any other admixture component except for Ket-Uralic (Suppl. Table 8, Suppl. files S8-S10). We also calculated Pearson's correlation coefficients for all possible pairs of major Y-chromosomal haplogroups and admixture components in three datasets (Suppl. files S8-S10). Pairs with $r > 0.8$ and p-value < 0.05 are shown in Suppl. Table 8. In the GenoChip-based dataset, the Q/Ket-Uralic component pair had the third lowest *p*-value among all pairs. In the other two datasets, this pair was not found among the best, but had a significant *p*-value in all cases.

The Mal'ta individual had a Y-haplogroup classified as a branch basal to the modern R haplogroup, and the modern haplogroup Q forms another sister-branch of haplogroup R (Raghavan 2014a). It is tempting to hypothesize that haplogroup Q1a correlates with ANE ancestry on a global scale: both reach their maxima in America and in few Siberian populations including Kets. Moreover, haplogroup Q1a has been found in 1 out of 4 male individuals of the Bronze Age Karasuk archaeological culture (3,400-2,900 YBP), and in 2 out of 3 Iron Age individuals from the Altai region (Allentoft et al. 2015). Altai's modern populations, as demonstrated in this study, have a rather large proportion of the Ket-Uralic admixture component. Importantly, the Karasuk culture has been tentatively associated with Yeniseian-speaking people (Chlenova 1975), and the Altai region is covered by hydronyms of Yeniseian origin (Dul'zon 1959, Vajda 2001). In this study we also demonstrated close genetic proximity of the Karasuk culture individuals and Kets (Suppl. Figs. 7.5, 7.6, 9.1).

In summary, correlation coefficients for both mitochondrial haplogroup U4 and Y-chromosomal haplogroup Q in Eurasia showed similar trends: the GenoChip-based dataset demonstrated the best correlation (Pearson's correlation coefficients about 0.8-0.9), while the other datasets showed correlation coefficients about 0.5 (Suppl. Table 8).

References

Allentoft, M. E. *et al*. Population genomics of Bronze Age Eurasia. Nature. **522,** 167–172 (2015).

Bramanti, B. *et al.* Genetic discontinuity between local hunter-gatherers and central Europe's first farmers. *Science* **326,** 137–140 (2009).

Brandt, G. *et al.* Ancient DNA reveals key stages in the formation of central European mitochondrial genetic diversity. *Science* **342,** 257–261 (2013).

Chlenova, N. L. Sootnoshenie kul'tur karasukskogo tipa i ketskikh toponimov na territorii Sibiri [The correlation between Karasuk-type cultures and Ket toponyms in Siberia]. *Etnogenez i Etnicheskaya Istoriya Narodov Severa [Ethnogenesis and History of the Peoples of the North]*. Moscow: Nauka, 223–230 (1975).

Derbeneva, O. A., Starikovskaya, E. B., Volodko, N. V., Wallace, D. C., Sukernik, R. I. Mitochondrial DNA variation in the Kets and Nganasans and its implications for the initial peopling of Northern Eurasia. *Russ. J. Genet.* **38,** 1316–1321 (2002).

Dulik, M. C. *et al.* Mitochondrial DNA and Y chromosome variation provides evidence for a recent common ancestry between Native Americans and Indigenous Altaians. *Am. J. Hum. Genet.* **90,** 229–246 (2012).

Dul'zon, A. P. Ketskie toponimy Zapadnoy Sibiri [Ket toponyms of Western Siberia]. *Uchenye Zapisky Tomskogo Gosudarstvennogo Pedagogicheskogo Instituta [Scholarly Proceedings of Tomsk State Pedagogical Institute]* **18,** 91–111 (1959).

Fedorova, S. A. *et al.* Autosomal and uniparental portraits of the native populations of Sakha (Yakutia): implications for the peopling of Northeast Eurasia. *BMC Evol. Biol.* **13,** 127 (2013).

Flegontova, O. V. *et al.* Haplotype frequencies at the DRD2 locus in populations of the East European Plain. *BMC Genet.* **10,** 62 (2009).

Fu, Q. *et al.* DNA analysis of an early modern human from Tianyuan Cave, China. *Proc. Natl. Acad. Sci. USA.* **110,** 2223–2227 (2013).
Karmin, M. *et al.* A recent bottleneck of Y chromosome diversity coincides with a global change in culture. *Genome Res.* **25,** 459–66 (2015).
Lell, J. T. *et al.* The dual origin and Siberian affinities of Native American Y chromosomes. *Am. J. Hum. Genet.* **70,** 192–206 (2002).
Malmström, H. *et al.* Ancient DNA reveals lack of continuity between neolithic hunter-gatherers and contemporary Scandinavians. *Curr. Biol.* **19,** 1758–1762 (2009).
Malyarchuk, B. A. Differentiation of the mitochondrial subhaplogroup U4 in the populations of Eastern Europe, Ural and West Siberia: implication for the genetic history of the Uralic populations. *Russ. J. Genet.* **40,** 1549–1556 (2004).
Raghavan, M. *et al.* Upper Palaeolithic Siberian genome reveals dual ancestry of Native Americans. *Nature* **505,** 87–91 (2014a).
Starikovskaya, E. B. *et al.* Mitochondrial DNA diversity in indigenous populations of the southern extent of Siberia, and the origins of Native American haplogroups. *Ann. Hum. Genet.* **69,** 67–89 (2005).
Tambets, T. *et al.* The Western and Eastern roots of the Saami – the story of genetic “outliers” told by mitochondrial DNA and Y chromosomes. *Am. J. Hum. Genet.* **74,** 661–682 (2004).
Vajda, E. J. *Yeniseian Peoples and Languages: a History of Their Study with an Annotated Bibliography and a Source Guide.* Surrey, England: Curzon Press, 389 p. (2001).
Yunusbayev, B. *et al.* The genetic legacy of the expansion of Turkic-speaking nomads across Eurasia. *PLoS Genet.* **11,** e1005068 (2015).

Suppl. Table 8. Pairs of mitochondrial or Y-chromosomal haplogroups and admixture components with Pearson correlation coefficient > 0.8 and *p*-value < 0.05. Three datasets were investigated. For each dataset, two pairs with the lowest *p*-values are highlighted in bold. For the Ket-Uralic admixture component, mitochondrial haplogroup U4, and Y-chromosomal haplogroup Q, all correlation coefficients with *p*-value < 0.05 are shown.

		**GenoChip + Illumina arrays**		**Ket genomes + Illumina arrays**		**Ket genomes + HumanOrigins array**	
**haplogroup**	**admixture component**	**r**	***p*-value**	**r**	***p*-value**	**r**	***p*-value**
			**mitochondrial (worldwide datasets)**				
M*	South Asian	0.96	4.2×10^{-5}				
M*	South Asian 1			**0.95**	$\mathbf{1.6\times10^{-10}}$		
M*	South Asian 2					**0.84**	$\mathbf{1.8\times10^{-4}}$
M7	South-East Asian	0.80	1.1×10^{-3}	0.84	1.4×10^{-3}	0.84	2.5×10^{-3}
M9	South-East Asian	0.87	1.2×10^{-3}	0.87	1.0×10^{-3}	0.86	7.4×10^{-4}
G1	Eskimo 2	0.86	3.5×10^{-2}				
G2	Siberian 2					**0.81**	$\mathbf{2.8\times10^{-4}}$
J2	South Asian 2			0.80	3.4×10^{-3}		
B*	South American 4			0.88	2.5×10^{-2}		
B5	South-East Asian	0.87	2.4×10^{-3}	0.82	1.4×10^{-2}	0.93	3.1×10^{-4}
pre-HV	South Asian 2			**0.96**	$\mathbf{6.0\times10^{-4}}$		
V	North European	0.83	2.1×10^{-5}				
H*	Middle Eastern	**0.83**	$\mathbf{2.2\times10^{-10}}$				
U3	Caucasian	0.83	4.0×10^{-7}				

U7	South Asian	0.85	1.0×10^{-3}				
U4	Ket-Uralic	**0.81**	**6.9×10^{-8}**	0.49	7.4×10^{-3}	0.42	3.8×10^{-2}
U4	South Asian 2			0.43	1.5×10^{-2}		
U4	Siberian 1					0.57	3.7×10^{-3}
**Y-chromosomal (datasets without American and Beringian populations)**							
C	Siberian 2	0.80	5.6×10^{-7}				
D	South Asian 1					0.94	1.9×10^{-2}
E	African			**0.86**	**7.0×10^{-3}**		
E	African 2	0.82	1.4×10^{-2}				
I	North European	**0.80**	**5.4×10^{-9}**			**0.81**	**6.3×10^{-10}**
O	South-East Asian	**0.91**	**3.4×10^{-8}**	**0.93**	**2.4×10^{-11}**	**0.96**	**1.1×10^{-16}**
Q	Ket-Uralic	0.91	2.4×10^{-7}	0.47	3.2×10^{-2}	0.47	4.2×10^{-2}
N	Ket-Uralic			0.46	1.5×10^{-2}		

Suppl. Table 9. Y-chromosomal haplogroup counts and frequencies in populations sampled in this study.

population	individuals	C3c1	I1a2	I2a1b3a	N1c1a1	N1c2b2	Q1a2a1	R1b1a
Enets	4				2 (50%)	1 (25%)	1 (25%)	
Ket	20		1 (5%)	1 (5%)			18 (90%)	
Nganasan	15	1 (6.7%)				14 (93.3%)		
Selkup	7						5 (71.4%)	2 (28.6%)

11. Neanderthal contribution to the Ket genomes

Methods

To estimate the Neanderthal gene flow influence we performed D-statistic analysis as described in Green et al. (2010). Reads for two Yoruba and two Kinh (Vietnamese) individuals were downloaded from the 1000 Genome Project database (McVean et al. 2012). We chose Yoruba samples NA19238 and NA19239, and Kinh Vietnamese samples HG01873 and HG02522 as they had read coverage similar to the Ket samples, and were not genetically related to each other. Ket, Yoruba, and Vietnamese reads were used for calling SNPs with GATK HaplotypeCaller, emitting both reference and non-reference sites, about 1 billion sites per individual. This procedure ensured that genotype calls for each individual were made in exactly the same way. Altai Neanderthal and chimpanzee genotypes were processed as described in Khrameeva et al. (2014). Coordinates of the chimpanzee genome were mapped to the human genome hg19 using UCSC liftOver tool (Rosenbloom et al. 2015).

In further analysis, we considered only homozygous sites different between the chimpanzee (A) and Neanderthal (B) genomes. Then we matched a randomly selected modern human allele to these sites. All sites where a Ket allele matched a Neanderthal allele and a Yoruba allele matched a chimpanzee allele were counted and referred to as #ABBA (termed Neanderthal-like sites). All sites where a Ket allele matched a chimpanzee allele and a Yoruba allele matched a Neanderthal allele were counted and referred to as #BABA. *D*-statistic = (#ABBA – #BABA)/(#ABBA + #BABA) was calculated and averaged for all possible pairs of Yoruba and Ket samples. As a control, the same analysis was repeated for Vietnamese genotypes instead of Ket genotypes.

Ket and Vietnamese sites used in the *D*-statistic analysis were assigned to human genes according to coordinates of the longest transcript retrieved from UCSC Genome Browser (Rosenbloom et al. 2015) plus 1,000 nucleotides upstream to include potential regulatory regions. The gene set enrichment analysis (GSEA) algorithm (Subramanian et al. 2005) ranked genes according to difference between #ABBA and #BABA, while four pairs of samples were treated as replicates. We used the MSigDB collection of 825 gene ontology (GO) biological processes (c5.bp.v3.0.symbols.gmt) (Subramanian et al. 2005) to assign genes to functional groups. GO terms with less than 15 or more than 500 genes per term were excluded. The mean and median false discovery rates (the mean FDR and median FDR) were used to estimate the significance of Neanderthal sites enrichment in the functional groups. In GSEA, the mean FDR was obtained by using the mean of the estimated number of false positives in each of 3000 permutations of the sample labels, while the median FDR was calculated as the median of the estimated number of false positives in the same permutations.

Results

To estimate the Neanderthal gene flow influence, we performed D-statistic analysis as described in Green et al. (2010). Given two Ket and two Yoruba individuals, we calculated the statistic *D*(Neanderthal, Chimp; Ket, Yoruba) for four different pairs of individuals. The mean *D*-statistic value, 3.85±0.15%, was in good agreement with other studies (Green et al. 2010, Khrameeva et al. 2014). As a control, we replaced the Ket genotypes with Vietnamese genotypes processed using the same

procedure. The control *D*-statistic value was 3.95±0.19% (Suppl. Table 10). Positive *D*-statistic values reflect higher similarity of Ket rather than Yoruba genotypes to Neanderthal genotypes, as expected for any non-African individuals.

In order to find Ket functional gene groups enriched in Neanderthal alleles we applied the GSEA algorithm to 'biological process' gene ontology (GO) terms (Khrameeva et al. 2014) (Suppl. file S11). No functional groups had mean FDR < 0.05, however two groups had median FDR < 0.05: 'amino acid catabolic process' with 24 genes (mean FDR = 0.6, median FDR = 0); and 'nitrogen compound catabolic process' with 28 genes (mean FDR = 0.7, median FDR = 0). It should be noted that the mean FDR was previously reported to overestimate the true false discovery rate when the sample size was small, while the median FDR was almost unbiased (Hirakawa et al. 2008). As the second functional group includes all genes from the first group, only the top group, 'amino acid catabolic process', is discussed further. ABBA and BABA site counts and D-statistics for individual genes in this group are shown in Suppl. file S11. Detailed inspection of site counts in individual genes showed the following genes with D-statistic > 50%: ASL, argininosuccinate lyase, a urea cycle enzyme crucial for ammonia removal; FAH, fumarylacetoacetate hydrolase, involved in tyrosine catabolism; GAD2, glutamate decarboxylase 2, involved in the synthesis of an important neurotransmitter γ-aminobutirate in the brain; GOT1, cytoplasmic isoform of glutamate-oxaloacetate transaminase, playing a role in amino acid metabolism and the urea and tricarboxylic acid cycles; GSTZ1, glutathione S-transferase zeta 1, involved in tyrosine and phenylalanine catabolism. The observed enrichment of Neanderthal-like sites in catabolic pathways associated with a protein-rich diet suggests that Kets and Neanderthals (Sistiaga et al. 2014) had similar dietary preferences. Indeed, the diet of Kets until today includes a large proportion of meat and fish, and Neanderthals were previously reported to predominantly consume meat (Sistiaga et al. 2014). We suggest that Kets, who abandoned the nomadic hunting lifestyle only in the middle of the 20th century, are a good model of genetic adaptation to protein-rich diets. However, we note that our results were obtained with a very small sample of Ket genomes, and the analysis has to be repeated when a much larger sample of Ket genomes becomes available.

We found one gene group potentially enriched in Neanderthal-like sites in control Vietnamese samples with median FDR < 0.05 ('response to nutrient' with 17 genes, mean FDR = 1, median FDR = 0). In a previous work (Khrameeva et al. 2014), genes involved in lipid catabolism (GO process 'lipid catabolic process') were shown to be significantly enriched in Neanderthal alleles in populations of European descent only. This effect was not observed for the Kets (FDR-corrected *p*-value = 1, see also site counts for individual genes in Suppl. file S11).

References

Green, R. E. *et al.* A draft sequence of the Neanderthal genome. *Science* **328,** 710–722 (2010).

Hirakawa, A., Sato, Y., Hamada, C., Yoshimura I. A new test statistic based on shrunken sample variance for identifying differentially expressed genes in small microarray experiments. *Bioinform. Biol. Insights* **2,** 145–156 (2008).

Khrameeva, E. E. *et al.* Neanderthal ancestry drives evolution of lipid catabolism in contemporary Europeans. *Nat. Commun.* **5** (2014).

McVean, G. A. *et al.* An integrated map of genetic variation from 1,092 human genomes. *Nature* **491,** 56–65 (2012).

Rosenbloom, K. R. *et al.* The UCSC Genome Browser database: 2015 update. *Nucl. Acids Res.* **43,** 670–681 (2015).

Sistiaga, A., Mallol, C., Galván, B., Summons, R. E. The Neanderthal meal: a new perspective using faecal biomarkers. *PLoS One* **9,** e101045 (2014).

Subramanian, A. *et al.* Gene set enrichment analysis: a knowledge-based approach for interpreting genome-wide expression profiles. *Proc. Natl. Acad. Sci. USA* **102,** 15545–15550 (2005).

Suppl. Table 10. *D*-statistics estimated for all possible pairs of Yoruba and Ket or Yoruba and Vietnamese samples.

**pair**	**#ABBA sites**	**#BABA sites**	***D*-statistic, %**	***D*-statistic average ± SD, %**
Ket1-Yoruba1	209,768	193,622	4.00	
Ket1-Yoruba2	204,774	189,615	3.84	
Ket2-Yoruba1	208,509	192,869	3.90	
Ket2-Yoruba2	202,379	188,133	3.65	3.85 ± 0,15
Vietnamese1-Yoruba1	209,155	192,351	4.19	
Vietnamese1-Yoruba2	203,379	187,689	4.01	
Vietnamese2-Yoruba1	208,851	193,389	3.84	
Vietnamese2-Yoruba2	204,916	190,029	3.77	3.95 ± 0,19
